# Cluster analysis of networks generated through homology: automatic identification of important protein communities involved in cancer metastasis

**DOI:** 10.1186/1471-2105-7-2

**Published:** 2006-01-06

**Authors:** Pall F Jonsson, Tamara Cavanna, Daniel Zicha, Paul A Bates

**Affiliations:** 1 Biomolecular Modelling Laboratory, Cancer Research UK London Research Institute, 44 Lincoln's Inn Fields, London WC2A 3PX, UK; 2 Light Microscopy Laboratory, Cancer Research UK London Research Institute, 44 Lincoln's Inn Fields, London WC2A 3PX, UK

## Abstract

**Background:**

Protein-protein interactions have traditionally been studied on a small scale, using classical biochemical methods to investigate the proteins of interest. More recently large-scale methods, such as two-hybrid screens, have been utilised to survey extensive portions of genomes. Current high-throughput approaches have a relatively high rate of errors, whereas in-depth biochemical studies are too expensive and time-consuming to be practical for extensive studies. As a result, there are gaps in our knowledge of many key biological networks, for which computational approaches are particularly suitable.

**Results:**

We constructed networks, or 'interactomes', of putative protein-protein interactions in the rat proteome – the rat being an organism extensively used for cancer studies. This was achieved by integrating experimental protein-protein interaction data from many species and translating this data into the reference frame of the rat. The putative rat protein interactions were given confidence scores based on their homology to proteins that have been experimentally observed to interact. The confidence score was furthermore weighted according to the extent of the experimental evidence, giving a higher weight to more frequently observed interactions. The scoring function was subsequently validated and networks constructed around key proteins, identified as being highly up- or down-regulated in rat cell lines of high metastatic potential. Using clustering methods on the networks, we have identified key protein communities involved in cancer metastasis.

**Conclusion:**

The protein network generation and subsequent network analysis used here, were shown to be useful for highlighting key proteins involved in metastasis. This approach, in conjunction with microarray expression data, can be extended to other species, thereby suggesting possible pathways around proteins of interest.

## Background

Microarray experiments provide information about gene expression within the cells under study.

Expression patterns can be uncovered from large-scale microarray data by systematically grouping genes with the help of clustering methods. Co-clustering of genes can indicate that the genes in question have a similar function or that they participate in the same cellular process [1,2]. Nevertheless, microarray experiments typically yield hundreds of significantly differentially-expressed genes, making it difficult to draw biological conclusions. Furthermore, although microarray experiments can show correlations between the expression of genes, they do not reveal the exact protein interaction mechanism.

Protein network analysis is dependent on a reliable assignment of protein-protein interactions. Protein-protein interactions are commonly studied using biochemical methods, and several different experimental methods are currently in use. Two-hybrid screens have, to date, yielded the bulk of available data [3,4]; however their level of accuracy is not particularly high and should be supported by additional evidence [5,6]. Advances in other techniques, such as tandem-affinity purification and mass spectroscopy, have also made large-scale studies increasingly feasible [7,8].

A number of computational methods, either based on sequence or structural features, have been developed to complement experimental approaches to predicting protein-protein interactions [9,10]. An increasing emphasis has been on deducing and exploring the protein-protein interaction networks that are reflected in expression data; gene networks have been inferred from gene expression data using mathematical analysis such as Bayesian regression [11-14]. Moreover, networks have been derived by complementing gene expression data with data from different sources, such as gene ontologies, phenotypic profiling and functional similarities [15-18].

Alternative techniques to network construction have also been taken, see e.g. Cabusora *et al*. [19], where a protein interaction map was created based upon the principle that interacting protein modules in one organism may be fused into a single chain in another, and Calvano *et al*. [20] who constructed the network by literature searches for information pertaining to interacting protein pairs from closely related organisms. These methods do not utilise gene expression explicitly in the network generation, rather the expression data is used as a tool to focus on the network.

Previous studies have mapped expression data of different systems onto experimentally-based networks. Ideker *et al*. [21] used gene expression changes in response to perturbation to highlight clusters within a yeast network, and Sohler *et al*. [22] made use of statistical analysis to highlight significant sub-clusters, also within a yeast network. Moreover, the dynamic aspect of yeast networks have been highlighted by de Lichtenberg and coworkers [23], who combined temporal cell cycle expression data with protein-protein interaction networks.

Here we have taken an extensive multi-genome approach, utilising a homology-based method for predicting interacting proteins [24] and further extended it by developing a scoring function, based upon sequence similarity and the amount of experimental data supporting each interaction. This scoring function has subsequently been extensively validated. In contrast to the above methodologies we go beyond data integration by considering orthologous relationships and are therefore able to create a more extensive protein interaction network – or 'interactome' – for a higher eukaryote, the rat.

In order to demonstrate the utility of our predicted interactions, expression data on tumour progression resulting in rat sarcomas with high metastatic potential were mapped onto our interactome, creating protein networks around key proteins involved in the metastatic process.

## Results and Discussion

**Figure 1:**
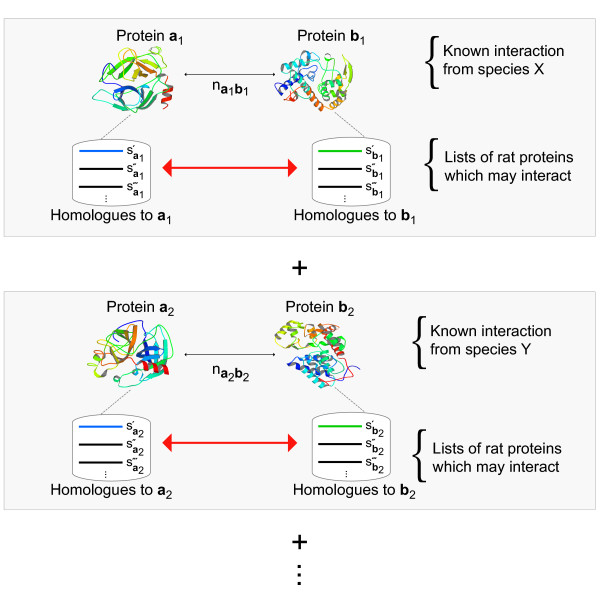
**Inferring interactions by homology**. Each interaction is inferred from homology to experimentally observed interactions. In this schematic, proteins **a**_**1 **_and **b**_**1 **_have been shown experimentally to interact in one organism, here labelled 'species X', and protein **a**_**2 **_and **b**_**2 **_in another, 'species Y'. Lists of homologues are generated for each of the proteins, ranked by their bit score (sai
 MathType@MTEF@5@5@+=feaafiart1ev1aaatCvAUfKttLearuWrP9MDH5MBPbIqV92AaeXatLxBI9gBaebbnrfifHhDYfgasaacH8akY=wiFfYdH8Gipec8Eeeu0xXdbba9frFj0=OqFfea0dXdd9vqai=hGuQ8kuc9pgc9s8qqaq=dirpe0xb9q8qiLsFr0=vr0=vr0dc8meaabaqaciGacaGaaeqabaqabeGadaaakeaacqWGZbWCdaWgaaWcbaacbeGae8xyae2aaSbaaWqaaiabdMgaPbqabaaaleqaaaaa@312D@, sbi
 MathType@MTEF@5@5@+=feaafiart1ev1aaatCvAUfKttLearuWrP9MDH5MBPbIqV92AaeXatLxBI9gBaebbnrfifHhDYfgasaacH8akY=wiFfYdH8Gipec8Eeeu0xXdbba9frFj0=OqFfea0dXdd9vqai=hGuQ8kuc9pgc9s8qqaq=dirpe0xb9q8qiLsFr0=vr0=vr0dc8meaabaqaciGacaGaaeqabaqabeGadaaakeaacqWGZbWCdaWgaaWcbaacbeGae8Nyai2aaSbaaWqaaiabdMgaPbqabaaaleqaaaaa@312F@, etc.). A protein from one list may interact with a protein from the other (shown by the red arrow) and potential pairwise interactions are scored according to Equation 1, based on homology to the proteins involved in the known interaction. Furthermore, interactions receive a higher score if they are derived from multiple experimental sources (*n *> 1). The score is additive, for instance, in the example here, the blue and green sequences are predicted to interact based on the interactions in 'species X' and 'species Y' and the overall score is the sum of both pairwise scores. This additive process continues over all experimentally determined protein pairs, *N*, (e.g. through 'species Z'), for which the rat sequences, labelled blue and green, are present.

Networks of interacting proteins were constructed automatically for the entire rat (*Rattus norvegicus*) genome using the approach described in the methods section and summarised in Figure 1. The number of individual interactions was reduced from 325,087 to 151,049, when a scoring function was applied to filter out low-quality data, and was further cut down by a clustering method aimed at identifying key interconnected network nodes. The interactome data is available though the PIP (Potential Interactions of Proteins) web server [25].

### Validation of the scoring function

The protein networks are composed of predicted individual interactions, each of which is assigned a score which indicates the strength of the prediction. Before examining the networks in detail it is necessary to assess the quality of the predictions and to validate the method.

#### Selection of cut-off value for the scoring function

**Figure 2:**
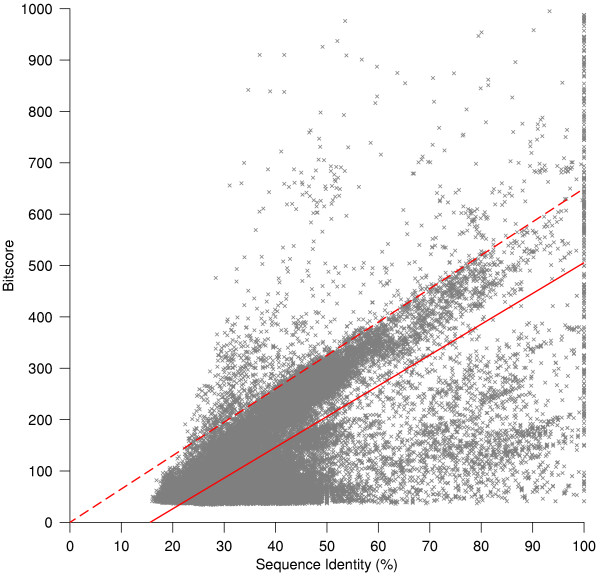
**The distribution of bit scores as a function of sequence identity**. The sequence identity and bit score of each hit when proteins in the interaction data were queried against the rat genome. The solid red line shows the best linear fit to the data and shown in dotted red is a line, starting at the origin, which contains 97% of the data in the area below it. Reading from these lines at 30% sequence identity gives bitscores of 86 and 177, respectively, yielding interaction scores of 9 and 10 when inserted into Equation 1. To ensure a stringent criteria for the minimum interaction score the higher value was selected as a cutoff score.

Our method of constructing networks is based on homology to known interactions. It is therefore imperative to ascertain the minimum level of homology whereby the structural and functional similarity of the interacting proteins is retained. Russell *et al*. [26] have previously examined the relationship between sequence and structural divergence of interacting proteins. They found that pairs of interacting proteins can be considered structurally similar if their sequence identity is no lower than 30%. As we utilise BLAST bitscores as components for our scoring function, we tested the relationship between bitscores and sequence identity. At the 30% sequence identity level, the bitscore ranges linearly from 86–177 (see Figure 2) which, according to Equation 1, yields minimum interaction scores ranging from 9 to 10. We chose to set the minimum score for interactions at 10, to minimise possibilities of false positive results due to low homology.

#### Identification of highly reliable interactions

Many methods for detecting protein-protein interactions can yield either false positive or false negative results, but X-ray crystal structures of complexed proteins can be considered to be a gold standard for proof. To examine the validity of our scoring function we looked at the interaction scores of rat proteins that have either been crystallised together in a complex or have a very high homology to one that has been. These scores were then compared to ones without any crystallographic evidence, i.e. those that do not interact or have not been proven to do so by crystallography.

We found that highly reliable interactions, identified by X-ray crystallography, score higher than those without crystallographic evidence, with median scores 128 and 16 respectively. This difference was significant according to a *χ*^2^-test (*p *≪ 0.0001), indicating that true interactions score higher than those whose association has not been confirmed by crystallography.

Moreover, as shown in Table 1, about 94% of the interactions confirmed by X-ray crystallography score above 10, reaffirming the choice of the cutoff score, whereas just under half of all genome-wide predicted interactions score 10 and lower.

#### Community participation and cellular localisation

Another way of estimating the quality of the scoring function is to look at proteins participating in the same cellular process and compare them with proteins that are not thought to interact directly in a pathway. We used a clique percolation method to identify 'communities' within the network that show high interconnectivity. This yielded 37 communities of tightly interconnected proteins that will be described later. One can assume that interactions within communities are more likely to be true than interactions between communities, i.e. higher scores would be expected for intra-community interactions [27]. We found this to be true; the average score for interactions within a community was 26.2 (*n *= 2038) and the average score for interactions between communities was 13.5 (*n *= 502). This is significant at a 95% confidence level (*p *= 3.1 × 10^-30^).

Lastly, the protein interaction scores were examined in the context of cellular localisation. We assume that for true interactions, interacting proteins would co-localise in the same cellular compartment, at least during the time of interaction, and thus would expect predicted interactions between proteins in separate cellular compartments to be less reliable and receive a lower score. Localisation data from the Gene Ontology Consortium [28] were used, where available, for proteins within the thirty-seven protein communities. Of the protein interactions predicted, 681 (94%) were considered co-localised, with an average score of 25.8 and 41 (6%) were annotated as not sharing cellular localisation, with an average score of 13.1. The score difference is statistically significant (*p *= 0.001 at a 95% confidence level).

### Metastatic network communities identified by cluster analysis

Metastasis is a key event that is associated with a poor prognosis in cancer patients. Metastasising cancer cells have the ability to break away from the primary tumour and move to different organs, making the cancer more difficult to treat. Much is unknown about the molecular biology of metastasis, but it culminates in the cancerous cells acquiring several properties, such as increased motility and invasiveness. This involves a network of cascading protein-protein interactions which have to be unravelled if an effective treatment is to be developed.

As a starting point, we used data from a microarray analysis of cell lines with different metastatic potentials (see Methods). We took the highest up- and down-regulated genes (≥ 4-fold up- or down-expression), and constructed networks around these, extending two generations from the starting point, i.e. initially including proteins that interact directly with the originating protein and then going on to include the proteins that interact with them. This subset of the rat interactome contained 10,628 interactions. We then performed a cluster analysis in order to highlight areas in the protein networks that are involved in the metastatic process. The clustering is based on a clique percolation method [29] that seeks to identify 'communities' of highly interconnected proteins that make up the essential structural units of the networks.

The community definition is based on the observation that a typical member in a community is linked to many, but not necessarily all other nodes in the community. In other words, a community can be regarded as a union of smaller, complete, fully-connected subgraphs that share nodes (see Methods section). Palla *et al*. [27] have shown that clique clustering analysis is a powerful tool to identify communities of proteins participating in the same cellular processes. Furthermore, it has been shown that subnetworks of proteins involved in a defined cellular process are more heavily interconnected by direct protein interaction than would be expected by chance [16]. Highly connected proteins are also more likely to be essential to cellular processes [30].

**Figure 3:**
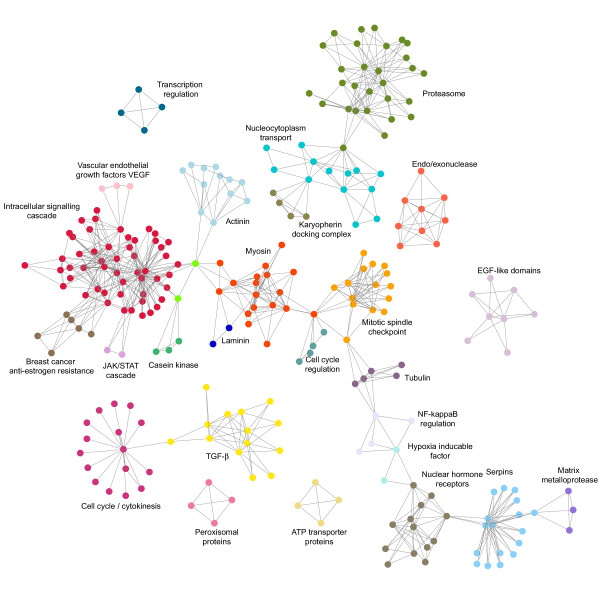
**Identifying protein communities by cluster analysis**. The communities identified by *k*-clique analysis performed on the predicted genome-wide rat protein network. The communities are distinguished by different colours and labelled by the overall function or the dominating protein class. Note that proteins, particularly at community edges, can belong to more than two communities, although this is not shown. A complete list of protein names is included as supplementary material [see Additional file 1]. The graph was created by Graphviz [61].

By applying the clustering method to our rat interactome we automatically identified 37 protein communities of highly interconnected proteins, containing 313 proteins involved in 1,094 interactions (Figure 3). The majority of the communities have been associated with cancer and metastasis. Some show a degree of overlap and are linked, the most prominent link running through the centre of the figure and containing 17 communities linked in a chain-like manner, however others are not linked, for example, the transcription regulation, which consists of only four proteins.

An initial analysis of the structural- and functional composition of the networks was performed using Domain Fishing [31], which assigns structural domains to sequences based on homology to known domains. When comparing the domain composition of the communities to domain frequencies of the whole rat genome we observed a bias towards classes of domains found in proteins involved in cytoskeletal structures, cell motility and cell-signalling (see Table 2). All but one of the most frequent domains are overrepresented when compared to the genome-wide distribution; only immunoglobulin domains appear less frequently. Spectrin repeat domains, which top the table, are found in proteins involved in cytoskeletal structure, such as spectrin, *α*-actinin and dystrophin. They are known to bind to calponin homology domains, which are found in both cytoskeletal and signal transduction proteins. The IQ calmodulin-binding domains work as Ca^2+ ^switches for myosin which are involved in cell motility and chemotaxis. Furthermore, protein kinase domains, SH2 and SH3 domains and protein-tyrosine phosphatase participate in signal transduction and known to interact. These categories of domains, and associated functions and interactions, are all of interest in the context of cancer metastasis.

#### The intracellular signalling cascade

It is not the aim here to explore every member of each community, instead, with the automatic identification of metastatic-related protein communities being the primary focus, we will illustrate the value of our approach by describing a key section of the regulation pathway. The intracellular signalling cascade constitutes the head of a chain of communities (Figure 3), and as such warrants a closer investigation.

**Figure 4:**
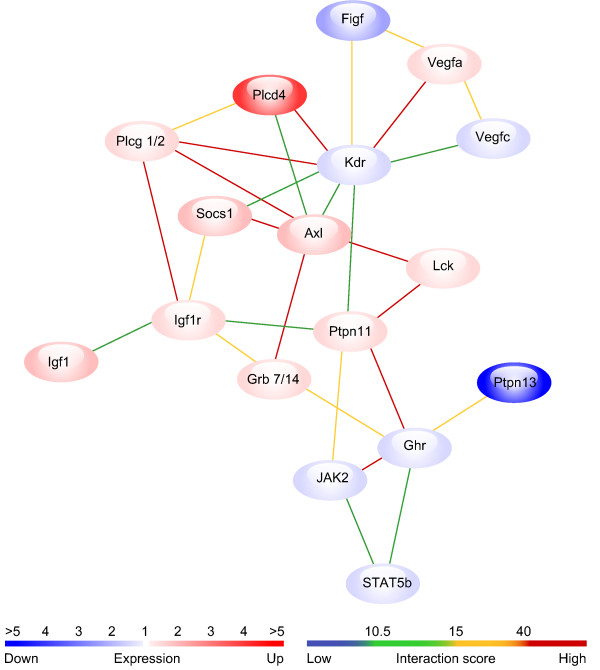
**A closer view of a part of the 'intracellular signalling cascade'**. The figure shows a subsection of the network around the intracellular signalling cascade where it extends to the VEGFs and JAK/STAT protein communities. The confidence of the interactions is shown by colour coding based on the interaction scores ranging from low-scoring blue (10 ≤ *s *< 10.5) to high-scoring red (*s *> 40.0). The metastatic cell line expression levels are also shown; blue for down-regulated genes and red for up-regulated ones.

Figure 4 shows a detailed view of some of the interactions within that community, focused on the intersection with the vascular endothelial growth factors (VEGFs) and the JAK/STAT group. Many of the interactions in this network have been established either in rat or in other species; others have not been previously demonstrated and we propose that these might have a role in the context of the surrounding proteins.

Three separate groups of proteins are distinguishable: vascular endothelial growth factors (Vegfa, Vegfc, Figf) and the receptor (Kdr), which play a principle role in tumour progression and angiogenesis [32] and which have also been associated with tumour metastasis [33]; insulin-like growth factors and receptors (Igf1, Igf1r and Grb 7/14); and JAK/STAT proteins (JAK2, STATSb).

The figure shows the three ligands, Vegfa and Vegfc and Figf, at different levels of expression, all of which can bind to kinase insert domain protein receptor Kdr, a VEGF receptor, which in turn induces mitogenesis and differentation of vascular endothelial cells [34].

The interaction between Kdr and Socs1, an SH2 domain-containing suppressor of cytokine signaling 1, is plausible as Kdr has a tyrosine protein kinase domain which in a mouse homologue has been shown to interact with Socs1 [35]. Furthermore, up-regulation of Socsl has been linked with the suppression of cytokine signalling and the JAK/STAT inflammatory signalling [36-38], which is shown here further down the network; here also, Socs1 is up-regulated and JAK/STAT down-regulated.

The proposed Ptpn11-Lck interaction is based on orthology to an interaction between Ptprc and Lck in mouse. Ptpn11 and Ptprc both have tyrosine specific protein phosphatase activity. Ptpn11 is phosphorylated by tyrosine protein kinases, contains two SH2 domains and therefore could be phosphorylated by Lck.

Higher up the network are the insulin-like growth factor 1 and its receptor (Igf1 and Igf1r, respectively) which are highly implicated in different cancers [39-41]. The insulin-like growth factors are involved in several cellular processes, such as regulation of proliferation, migration, survival, size control, and differentiation [42-45]. Igf1r is overexpressed in most malignant tumours, where it functions as an anti-apoptotic agent by enhancing cell survival. Igf1 has also been shown to enhance adhesion and motility of cancer cells [46,47]; however, the exact role of Igf1r in the metastatic process has not been established. The network shown here suggests a link between the insulin-like growth factor receptor and the vascular endothelial growth factors through the highly up-regulated phospholipase delta 4 (Plcd4) and phospholipase gamma 1/2 (Plcg 1/2). The Plcg 1/2 and Igf1r interaction is based on the fact that the phospholipase has been shown to interact with insulin receptor, a close homologue of the insulin-like receptor.

Another distinguishing feature in the network is the highly down-regulated protein tyrosine phosphatase (Ptpn13). It has been reported that a protein tyrosine phosphatase, Ptp61F, negatively regulates the JAK/STAT pathway in *Drosophila melanogaster *[48]. Our networks suggest that the signalling protein tyrosine phosphatase, Ptpn13, may act on the JAK/STAT pathway similarly, through the dephosphorylation of the growth hormone receptor Ghr.

The few examples shown here illustrate the value of the approach in terms of revealing potential pathways and interactions that play a part in cancer metastasis, but further experimental work will be needed to confirm the validity of these predictions.

### Network view of gene expression

Extracting meaningful information from microarray expression data is often difficult, especially when looking at a complex process involving a large number of genes and unknown mechanisms. Clustering of genes may be of use when trying to find genes in a common pathway and genes with related function, but it often has limitations, such as in identifying negative feedback loops [49]. Furthermore, even if key proteins are highlighted through microarray analysis, the expression data rarely reveals all proteins involved in a particular pathway.

Examining the distribution of up- and down-regulated proteins in the context of their neighbours, shows that this is indeed the case for the protein networks shown in Figure 3. The metastatic expression data was mapped onto the networks and the frequency of the up-, down- and up-/down-regulated genes interacting was examined. The results, in Table 3, indicate that if expression data from the network was randomly redistributed, the probability of observing two up-regulated proteins interacting with each other is about the same as the observed probability. That is, up-regulated proteins do not have a trend of directly interacting with each other, but are interlinked through either neutrally expressed or down-regulated proteins. Moreover, down-regulated proteins are much less likely to interact with each other than expected, demonstrating the benefit of projecting the expression data onto already built networks, as clustering similarly expressed genes and assigning to the same pathway would not be effective.

## Conclusion

Expression data has previously been put into a network context, for example by incorporating gene ontology data [15] and protein interactions [50], but here we generated the networks first, mapped the expression on top, and then performed a clustering. This approach allows us to bypass some of the obstacles involved in traditional microarray analysis, such as clustering of gene expression patterns; as demonstrated here, interactions of up-up and down-down regulated genes are not necessarily co-localised. To focus on the parts of the genome-wide interaction network relevant to metastasis we first selected subnetworks around highly up- and down-regulated genes and then utilised the clique method, which highlights hubs of highly interconnected protein communities. This allows us to examine the most complex parts of the network but as a result simple linear pathways do not get included.

Although we believe the general approach is of value in protein network analysis, there remain some shortcomings. Most importantly, transient protein-protein interactions are unlikely to be captured by our approach. This is a direct consequence of transient not being as well documented as non-transient interactions. Moreover, the method cannot distinguish between true and false positives, for which there is limited experimental data – however, these problems will be alleviated as more high-throughput proteomic studies are completed.

The system-level approach taken here, combining information on how proteins interact with each other and how genes are expressed, is a particularly appealing way to gain understanding of complex biological processes, such as metastasis. Although not discussed here in great detail several interesting groups of interactions, at the domain level, have been highlighted as potentially important players in the metastatic process. Further dissection of these is the subject of ongoing studies and consequently to be confirmed experimentally.

This method of using homologous protein interaction data to infer protein-protein information could be particularly useful for proteins for which there is no definite binding partner information. Moreover the relative expression levels of neighbouring proteins may prove an important consideration, when protein networks are to be subsequently modulated in conjunction with disease analysis, for example by targeting the expression of a particular gene by short interfering RNA (siRNA) [51].

The approaches described in this work are readily transferable to other species and cellular processes.

## Methods

### Protein interaction prediction

In order to identify homologous interaction pairs for which there is experimental data, BLAST searches were run for the rat genome [52] against all proteins in the DIP [53] and MIPS Mammalian Protein-Protein Interaction databases [54].

The experimental data arises from several methods – the most frequent are listed in Table 4. The putative interactions were given confidence scores based on two factors: the level of homology to proteins found experimentally to interact, and the amount of experimental data available (see Figure 1 for an illustrationof the approach).

The score, *S*, was calculated for each putative interaction according to the following:

S=∑i=1Nln⁡(saisbi)n,     (1)
 MathType@MTEF@5@5@+=feaafiart1ev1aaatCvAUfKttLearuWrP9MDH5MBPbIqV92AaeXatLxBI9gBaebbnrfifHhDYfgasaacH8akY=wiFfYdH8Gipec8Eeeu0xXdbba9frFj0=OqFfea0dXdd9vqai=hGuQ8kuc9pgc9s8qqaq=dirpe0xb9q8qiLsFr0=vr0=vr0dc8meaabaqaciGacaGaaeqabaqabeGadaaakeaacqWGtbWucqGH9aqpdaaeWbqaaiGbcYgaSjabc6gaUjabcIcaOiabdohaZnaaBaaaleaaieqacqWFHbqydaWgaaadbaacbiGae4xAaKgabeaaaSqabaGccqWGZbWCdaWgaaWcbaGae8Nyai2aaSbaaWqaaiab+LgaPbqabaaaleqaaOGaeiykaKIaemOBa4MaeiilaWcaleaacqWGPbqAcqGH9aqpcqaIXaqmaeaacqWGobGta0GaeyyeIuoakiaaxMaacaWLjaWaaeWaaeaacqaIXaqmaiaawIcacaGLPaaaaaa@492A@

where sai
 MathType@MTEF@5@5@+=feaafiart1ev1aaatCvAUfKttLearuWrP9MDH5MBPbIqV92AaeXatLxBI9gBaebbnrfifHhDYfgasaacH8akY=wiFfYdH8Gipec8Eeeu0xXdbba9frFj0=OqFfea0dXdd9vqai=hGuQ8kuc9pgc9s8qqaq=dirpe0xb9q8qiLsFr0=vr0=vr0dc8meaabaqaciGacaGaaeqabaqabeGadaaakeaacqWGZbWCdaWgaaWcbaacbeGae8xyae2aaSbaaWqaaiabdMgaPbqabaaaleqaaaaa@312D@ and sbi
 MathType@MTEF@5@5@+=feaafiart1ev1aaatCvAUfKttLearuWrP9MDH5MBPbIqV92AaeXatLxBI9gBaebbnrfifHhDYfgasaacH8akY=wiFfYdH8Gipec8Eeeu0xXdbba9frFj0=OqFfea0dXdd9vqai=hGuQ8kuc9pgc9s8qqaq=dirpe0xb9q8qiLsFr0=vr0=vr0dc8meaabaqaciGacaGaaeqabaqabeGadaaakeaacqWGZbWCdaWgaaWcbaacbeGae8Nyai2aaSbaaWqaaiabdMgaPbqabaaaleqaaaaa@312F@ are sequence similarity bit scores to proteins **a**_*i *_and **b**_*i*_, respectively, which have experimentally been shown to interact; *n *is the number of experiments linking protein **a**_*i *_to protein **b**_*i*_; and *N *is the total number of instances where the same pair of proteins is identified as interacting through different homologues. As mentioned in the Introduction, two-hybrid experiments are prone to giving false-positive results. Although most of the interactions created here are derived through yeast two-hybrid links, it has been shown that confidence is higher for interactions detected in multiple independent yeast two-hybrid experiments [15]. This fact is reflected in the additive nature of the score, where a protein interaction that shows up repeatedly in independent two-hybrid experiments gets a higher score.

### Validation

In order to test the scoring function, we created a subset of data from the RCSB Protein Data Bank [55] that specifically concentrates on stable functional protein interactions, rather than transient. Protein chains with high sequence homology to the Norwegian rat were considered (*e *≤ 1 × 10^-10^). We distinguished biologically functional complexes (where multimeric protein chains are permanently bound and essential for the complex function) from transient ones (where protein chains may be bound to a complex but may also act as a separate functional protein on its own), by applying a method proposed by Ofran and Rost [56]. This yielded 377 binary chain interactions.

Cellular localisation of proteins was obtained from the Gene Ontology Consortium [28]. Each of the proteins identified by the cluster analysis was placed in a basic cellular localisation class as per Table 5. Protein pairs predicted to interact were considered co-localised if they were found in compatible cellular compartments.

### Creation of networks around up/down-regulated genes

Rat genes that were overexpressed or underexpressed more than four-fold were used as starting points (*n *= 100). Networks were expanded two generations out from the starting points using protein-protein interactions whose *S*-score value was 10 or higher. The resulting 10,628 interactions were then analysed using CFinder [27], which locates maximal complete subgraphs (*k*-cliques) in the networks and then identifies 'communities' by carrying out standard component analysis of the clique-clique overlap. In this context, the variable *k *is defined as the number of nodes in the subgraph and a *k*-clique community is defined as the union of all *k*-cliques that can be reached from each other through a series of adjacent *k*-cliques, where cliques sharing *k *- 1 nodes are defined as adjacent. Table 6 shows the number of individual protein communities for different *k*-values. Thirty-seven communities were identified for *k *= 4, i.e. setting the subgraph size threshold to a minimum of four. Selecting the *k*-value is a balancing act; the higher the *k*-value, the smaller and more internally connected the communities become, but less connection is observed between communities. The *k*-value was selected after observing that at *k *= 4, reasonably large communities were formed. Proteins which shared sequence identity higher than 40% within each community, were merged together such that they appeared as a single nodes on the protein map. These merged nodes inherited all the interactions from the individual proteins before the merging process. This was done to correct for any possible redundancies caused by our homology-based method for predicting protein interactions. There was negligible change in the protein networks as a result of this.

### Microarray expression data for metastatic rat cells

To investigate genes that may be important in the development of metastases, we used a rat sarcoma model in which the cell populations K2, T15, A297 and A311 have 0, 40, 90 and 100% incidence of metastasis, respectively. We performed Affymetrix microarray analysis on the four cell populations and the primary tumours that formed when the cells were injected subcutaneously into rats. All experiments were performed in triplicate, using Affymetrix rat 230A GeneChip oligonucleotide arrays [57]. Total RNA was extracted from each sample and used to prepare biotinylated target RNA; 10 *μ*g of RNA was used to generate first-strand cDNA by using a T7-linked oligo(dT) primer. After second-strand synthesis, in vitro transcription was performed with biotinylated UTP and CTP (Enzo Diagnostics), resulting in approximately 100-fold amplification of RNA. A complete description of the procedures is included in The Paterson Institute's Affymetrix GeneChip systems protocols [58].

The target cRNA generated from each sample was processed as per the manufacturer's recommendation using an Affymetrix GeneChip Instrument System [59]. Briefly, spike controls were added to 10 *μ*g fragmented cDNA before overnight hybridisation, arrays were washed and stained with streptavidin-phycoerythrin, and scanned on an Affymetrix GeneChip scanner. The procedure is further described in The Paterson Institute's RNA Hybridisation protocols [60]. The median fluorescence intensity value of each GeneChip was calculated and used to normalise the chips. Gene expression was considered in terms of fold-changes between non-metastatic and the median of the three metastatic samples.

## Authors' contributions

PFJ constructed and analysed the protein networks and drafted the manuscript. TC carried out the microarray analysis on metastatic rat cell lines. PAB initiated the construction of the interactome and DZ its integration with the microarray data. PAB and DZ coordinated and participated in discussions and the preparation of the manuscript. All authors have read and approved the final manuscript.

## Supplementary Material

Additional File 1 Metastasis-related protein communities. List of the proteins identified by the clique analysis. Click here for file

Additional File 2 Microarray data. The microarray expression data used in this study. Click here for file

## Figures and Tables

**Table 1:** Distribution of protein-protein interaction scores. Interaction scores of X-ray crystal structures (*n *= 377) compared to the scores of all (genome-wide) predicted interactions.

	Percentage of interactions	
	Interaction score 0 – 10	Interaction score > 10
X-ray crystal structures	6.4	93.6
Genome-wide	43.2	56.8

**Table 2:** Domain frequency within the clustered communities. The table shows the most frequently observed domains in the metastasis-related cluster communities (observed frequencies) alongside the expected domain frequencies, based on the domain composition of the whole rat genome. The *n*-fold difference was calculated from the frequency percentages (numbers within parentheses).

Domain	Observed frequency (%)	Expected frequency (%)	*n*-fold difference
Spectrin repeat	56 (6.9)	6 (0.7)	8.3
IQ calmodulin-binding motif	54 (6.6)	2 (0.2)	26.5
EGF-like domain	52 (6.4)	16 (2.0)	2.2
Protein kinase domain	47 (5.8)	12 (1.4)	3.0
SH2 domain	27 (3.3)	2 (0.3)	11.7
EF hand	25 (3.1)	7 (0.8)	2.6
Immunoglobulin domain	21 (2.6)	35 (4.3)	-0.4
SH3 domain	20 (2.4)	6 (0.7)	2.6
Calponin homology (CH) domain	13 (1.6)	2 (0.3)	5.4
Proteasome A-type and B-type	12 (1.5)	1 (0.1)	20.0
LIM domain	11 (1.3)	3 (0.4)	2.7
Transforming growth factor *β*-like domain	10 (1.2)	1 (0.1)	11.2

**Table 3:** The connectivity of up- and down-regulated proteins. Observed and expected frequencies of pairwise protein interactions, categorised by their expression: N-N (non-expressed protein interacting with non-expressed protein), U-U (up-regulated protein interacting with up-regulated protein), D-D (down-regulated protein interacting with down-regulated protein) and U-D (up-regulated interacting with down-regulated). For the purpose of the classification, up-regulated proteins are those up-regulated more than 20% and down-regulated proteins down-regulated more than 20%. Expected values were calculated based on a random distribution of the expression data on the network (*p *< 0.001 for a *χ*^2^-test).

	Observed	Expected	*n*-fold difference
N-N	8	5	1.5
U-U	121	109	1.1
D-D	17	41	0.4
U-D	71	67	1.1

**Table 4:** Experimental sources for building the interactome. Summary of the experiments used as a foundation for building the interactome, from most frequent (top) to least frequent (bottom). The percentage of the total is listed after each value.

Method	Frequency (%)
Two hybrid test	35,759 (69.9)
Immunoprecipitation	6,290 (12.3)
Tandem Affinity Purification (TAP)	3,503 (6.85)
Affinity chromatography	1,070 (2.09)
Copurification	572 (1.12)
Cross-linking	518 (1.01)
X-ray crystallography	511 (1.00)
In vitro binding	452 (0.88)
Biochemical/biophysical	327 (0.64)
Gel filtration chromatography	326 (0.64)
In vivo kinase activity assay	185 (0.36)
Competition binding	185 (0.36)
Immunoblotting	140 (0.27)
Cosedimentation	133 (0.26)
Gel retardation assays	106 (0.21)
Native gel electrophoresis	103 (0.20)
	
Other	973 (1.90)

**Table 5:** Gene ontology cellular compartments. A simplified representation of gene ontology cellular compartments. Protein accessibility between compartments is represented by ones and zeros: the former indicates the possibility of interaction between respective compartments and the latter excludes any interactions.

	Extracellular	Intracellular	Cytoplasm	Nucleus	Mitochondrion	Membrane
Extracellular	1	0	0	0	0	1
Intracellular	0	1	1	1	1	1
Cytoplasm	0	1	1	0	0	1
Nucleus	0	1	0	1	0	1
Mitochondrion	0	1	0	0	1	0
Membrane	1	1	1	1	0	1

**Table 6:** The number of protein communities at different clustering threshold values. The number of protein communities vary as the *k*-value for clustering is changed. The table shows the total number of separate protein communities for each *k*-value.

Clustering threshold value	Number of protein communities
*k *= 3	145
*k *= 4	37
*k *= 5	12
*k *= 6	8
*k *= 7	2
*k *= 8	1
*k *= 9	1
*k *= 10	1
*k *= 11	1
