# Supplementary material for: Cluster analysis of networks generated through homology: automatic identification of important protein communities involved in cancer metastasis
Source: BMC Bioinformatics. 2006 Jan 6;7:2. doi: 10.1186/1471-2105-7-2 (PMC1363365; doi:10.1186/1471-2105-7-2)
Supplement: Additional File 1 — Metastasis-related protein communities. List of the proteins identified by the clique analysis. [file 1471-2105-7-2-S1.pdf]

Sequence ID	Gene name	Description
**Community 0: TGF-beta**		
NP_036722	Inha	inhibin alpha
NP_036907	Tgfbr1	transforming growth factor, beta receptor 1
NP_036959	Bmp4	bone morphogenetic protein 4
NP_037234	Fkbp1a	FK506-binding protein 1a
NP_037239	Bmp6	bone morphogenetic protein 6
NP_037306	Tgfb3	transforming growth factor, beta 3
NP_058824	Inhba	inhibin beta A
NP_058874	Bmp2	bone morphogenetic protein 2
NP_058952	Tgfbr3	transforming growth factor, beta receptor 3
NP_058973	Ap1b1	adaptor protein complex AP-1, beta 1 subunit
NP_059052	Hpcal1	neural visinin-like Ca2+-binding protein type 3
NP_067589	Tgfb1	transforming growth factor, beta 1
NP_071886	Acreq	activin A receptor type II-like 1
NP_077342	Freq	neuronal calcium sensor-1
NP_077812	Acvr1	activin type I receptor
NP_110476	Bmpr1a	bone morphogenetic protein receptor, type 1A
NP_112393	Tgfb2	transforming growth factor, beta 2
NP_112394	Tgfbr2	transforming growth factor-b type II receptor
NP_954700	Acvr1b	activin A receptor, type 1B
XP_217147		similar to mmDj4
XP_217297		activin receptor IIB
XP_217409		similar to Bone morphogenetic protein type II receptor
XP_227759		similar to CFK-43a=bone morphogenetic protein binding ser/thr kinase receptor
XP_232168		similar to DNA replication licensing factor MCM2
XP_342432		activin receptor IIA
XP_342592		bone morphogenetic protein 7
XP_345647		similar to growth/differentiation factor 7
**Community 1: Proteasome**		
NP_001005875	Psmd12	proteasome 26S non-ATPase subunit 12
NP_001008218	Psma7	proteasome (prosome, macropain) subunit, alpha type 7
NP_001008282	Psmd3	proteasome 26S non-ATPase subunit 3
NP_001008302	Usp14	ubiquitin specific protease 14
NP_058736	Ppp2cb	protein phosphatase 2a, catalytic subunit, beta isoform
NP_058796	Plk1	polo-like kinase 1
NP_058974	Psma1	proteasome (prosome, macropain) subunit, alpha type 1
NP_058975	Psma2	proteasome (prosome, macropain) subunit, alpha type 2
NP_058976	Psma3	proteasome (prosome, macropain) subunit, alpha type 3
NP_058977	Psma4	proteasome (prosome, macropain) subunit, alpha type 4
NP_058979	Psma6	proteasome (prosome, macropain) subunit, alpha type 6
NP_058980	Psmb2	proteasome (prosome, macropain) subunit, beta type 2
NP_058981	Psmb3	proteasome (prosome, macropain) subunit, beta type 3
NP_112411	Sug1	proteasomal ATPase (SUG1)
NP_112621	Psmd4	proteasome (prosome, macropain) 26S subunit, non-ATPase, 4
NP_113817	Psmb4	proteasome (prosome, macropain) subunit, beta type 4
NP_150239	Psmc2	proteasome (prosome, macropain) 26S subunit, ATPase 2
NP_476440	Psmb6	proteasome (prosome, macropain) subunit, beta type 6
NP_476463	Psmc4	proteasome 26S ATPase subunit 4
NP_942025	MGC72968	proteasome, 26S, non-ATPase regulatory subunit 6
XP_213456		similar to Protein translation factor SUI1 homolog
XP_215745		similar to 26S proteasome-associated pad1 homolog
XP_218424		similar to nucleotide excision repair protein
XP_220754		similar to 26S proteasome non-ATPase regulatory subunit 11
XP_224534		similar to karyopherin beta 3; Ran_GTP binding protein 5; importin beta-3 subunit
XP_226439		similar to 26S proteasome non-ATPase regulatory subunit 7
XP_233467		similar to cytidine 5-triphosphate synthase
XP_341315		proteasome (prosome, macropain) subunit, beta type 5
XP_342114		similar to Periodic tryptophan protein 2 homolog

XP_344650		similar to Proteasome subunit alpha type 7-like
XP_344977		similar to 26S proteasome subunit p40.5

Community 2: Mitotic spindle checkpoint

NP_001008331	Polr1c	RNA polymerase I subunit
NP_036942	Sycp1	synaptonemal complex protein 1
NP_071582	Rad50	RAD50 homolog
NP_071790	Pmpcb	mitochondrial processing peptidase beta
NP_113771	Cspg6	chondroitin sulfate proteoglycan 6
NP_113871	Smc1l1	SMC1 structural maintenance of chromosomes 1-like 1
NP_114009	Plk2	polo-like kinase 2
NP_703201	Rnf40	ring finger protein 40
NP_955795	Cdk2	cyclin-dependent kinase 2
XP_215573		similar to SMC4 protein
XP_218574		similar to BC013491 protein
XP_222860		similar to NUF2R protein
XP_233108		similar to stromal antigen 2
XP_233337		similar to epidermal growth factor receptor pathway substrate 15
XP_235267		similar to pokeweed agglutinin-binding protein
XP_342838		similar to SMC2 protein

Community 3: Myosin

NP_001009268	Actr2	actin-related protein 2
NP_058936	Myh7	myosin heavy chain, polypeptide 7
NP_068640	Cttn	cortactin isoform B
NP_112406	Actb	cytoplasm beta-actin
NP_112408	Arpc1a	suppressor of profilin/p41 of actin-related complex 2/3
NP_446372	Trip10	thyroid hormone receptor interactor 10
NP_446438	Myo1b	myosin Ib
NP_476540	Waspip	Wiskott-Aldrich syndrome protein interacting protein
NP_775124	Myo1e	myosin IE
NP_955795	Cdk2	cyclin-dependent kinase 2
XP_217432		similar to actin related protein 2/3 complex subunit 2; ARP2/3 complex subunit 34
XP_218617		similar to nonmuscle myosin heavy chain
XP_228784		similar to Wiskott-Aldrich Syndrome Protein
XP_233945		similar to mKIAA1256 protein
XP_238365		similar to actin related protein 2/3 complex, subunit 4
XP_239604		similar to GluR-delta2 philic-protein
XP_340818		myosin, heavy polypeptide 4
XP_341113		actin-related protein 3 homolog
XP_343046		similar to Sh3yl1

Community 4: Intracellular signaling cascade

NP_001002289	Fut8	fucosyltransferase 8 (alpha (1,6) fucosyltransferase)
NP_001004081	Mpi	mannose phosphate isomerase
NP_001008725	Il6st	interleukin 6 signal transducer
NP_036645	Bdnf	brain derived neurotrophic factor
NP_036742	Ngfr	nerve growth factor receptor, fast
NP_036863	Ntrk2	neurotrophic tyrosine kinase, receptor, type 2
NP_036887	Fyn	fyn proto-oncogene
NP_036921	Dpp4	dipeptidylpeptidase 4
NP_036978	Fgf1	fibroblast growth factor 1
NP_037101	Irs1	insulin receptor substrate 1
NP_037137	Pik3r	phosphatidylinositol 3-kinase, regulatory subunit, polypeptide 1
NP_037194	Kdr	kinase insert domain protein receptor
NP_037213	Ptk2	PTK2 protein tyrosine kinase 2
NP_037220	Ptpn11	protein tyrosine phosphatase, non-receptor type 11
NP_037319	Plcg1	phospholipase C, gamma 1
NP_058762	Ptn	pleiotrophin
NP_058767	Insr	insulin receptor
NP_058790	Ghr	growth hormone receptor

NP_058864	Plcg2	phospholipase C, gamma 2
NP_059037	Lipf	lipase, gastric
NP_062121	Ntrk3	neural receptor protein-tyrosine kinase
NP_062178	Fgf2	fibroblast growth factor 2
NP_067600	Ntrk1	trk precursor
NP_071549	Pik3r3	phosphatidylinositol 3-kinase p55 subunit
NP_071963	Arf1	ADP-ribosylation factor 1
NP_110483	Lrrn3	leucine rich repeat protein 3, neuronal
NP_110486	Mdk	midkine
NP_112335	Ntf3	neurotrophin 3
NP_112624	Cdh1	cadherin 1
NP_113702	Jak2	Janus kinase 2
NP_113811	Grb14	growth factor receptor bound protein 14
NP_434694	Igf1r	insulin-like growth factor 1 receptor
NP_445775	Degs	degenerative spermatocyte homolog
NP_445809	Catnb	beta-catenin
NP_445855	Grb7	growth factor receptor binding protein GRB7
NP_476547	Ncr1	lymphocyte antigen 94 (mouse) homolog (activating NK-receptor; NK-p46)
NP_542419	Plcd4	phospholipase C, delta 4
NP_604451	Sh2bpsm1	SH2-B PH domain containing signaling mediator 1
NP_612516	Ptprc	protein tyrosine phosphatase, receptor type, C
NP_665725	Cyp3a18	cytochrome P450, 3a18
NP_665886	Socs1	suppressor of cytokine signaling 1
NP_849197	Igf1	insulin-like growth factor 1
XP_213997	Ptpn13	similar to protein Tyr phosphatase, non-receptor type 13
XP_214050	Lap3_pred	leucine aminopeptidase 3 (predicted)
XP_217246	Nck1_pred	similar to non-catalytic region of tyrosine kinase adaptor protein 1
XP_217250	Ephb1	PREDICTED: Eph receptor B1
XP_218346	Axl_pred	now NP_001013165. AXL receptor tyrosine kinase (predicted)
XP_221036	LOC303606	now NP_001013996.hypothetical protein
XP_224344	Dok2_pred	similar to docking protein Dok-R
XP_226503	LOC307845	similar to hypothetical protein BC002770
XP_227525	Ngfb	similar to nerve growth factor beta chain precursor - multimammate rat
XP_231137	Abl1	similar to Abl1 protein Proto-oncogene tyrosine-protein kinase ABL1
XP_232763	Lck	lymphocyte-specific protein tyrosine kinase
XP_233522	LOC298528	similar to Ephrin type-A receptor 10
XP_235164	Frs2_pred	similar to fibroblast growth factor receptor substrate 2
XP_236628	Mst1r_pred	similar to hepatocyte growth factor-like protein receptor
XP_341110	Ptpn4	similar to testis-enriched protein tyrosine phosphatase
XP_342283	Shc1	now NP_445969 SHC (Src homology 2 domain-containing) transforming protein 1
XP_342864	Tek	similar to TIE-2=receptor-like tyrosine kinase
XP_343062	LOC362737	similar to RIKEN cDNA D930036F22 gene
XP_347256		similar to met proto-onco

Community 5: EGF-like domain containing proteins

NP_058928	Ptgs2	prostaglandin-endoperoxide synthase 2
NP_062020	Jag1	jagged 1
NP_077334	Notch2	notch gene homolog 2
NP_446196	Dlk1	delta-like 1 homolog
NP_620192	Pou3f3	POU domain, class 3, transcription factor 3
NP_942048	Rpl3	ribosomal protein L3
XP_232595		similar to Rbpsuh protein
XP_241375		similar to N-terminal aceyltransferase 1
XP_343120		jagged 2

Community 6: Endo/exonuclease

NP_001008383	Cnot8	similar to CCR4-NOT transcription complex, subunit 8 (CAF1-like protein)
NP_001009357	Rqcd1	RCD1 required for cell differentiation1 homolog
XP_216889		similar to CCR4-NOT transcription complex, subunit 2; NOT2 (neg. reg. of transcr)
XP_218062		similar to LATS homolog 1
XP_218187		similar to CCR4-NOT transcription complex, subunit 3

XP_224583		similar to CG31759-PA endo/exonuclease activity
XP_226233		similar to KIAA1007 protein; adrenal gland protein AD-005
XP_231603		similar to potential transcriptional repressor Not4hp
XP_341192		similar to Hypothetical protein 4932442K20Rik

Community 7: Nucleocytoplasm transport

NP_058759	Kpnb1	karyopherin (importin) beta 1
NP_059057	Nup54	nucleoporin 54kDa
NP_445891	Ran	RAN, member RAS oncogene family
NP_446074	Pom121	nucleus pore membrane glycoprotein 121 kD
NP_703206	Krt1-9	keratin complex 1, acidic, gene 9
NP_942021	Kpna1	karyopherin alpha 1 (importin alpha 5)
XP_214639		similar to RIKEN cDNA 2410008G02
XP_218620		similar to nucleus pore glycoprotein p62 (62 kDa nucleoporin)
XP_224534		similar to karyopherin beta 3; Ran_GTP binding protein 5; importin beta-3 subunit
XP_341509		nucleoporin 153kD

Community 8: Cell cycle/cytokinesis

NP_037027	Adk	adenosine kinase
NP_037309	Got2	glutamate oxaloacetate transaminase 2
NP_072138	Sept7	CDC10 (cell division cycle 10, S.cerevisiae, homolog)
NP_076481	Prkaa2	AMP-activated protein kinase alpha 2 catalytic subunit
NP_114025	Sept9	septin 9
NP_446023	Rgpr	regucalcin gene promotor region related protein
NP_446383	Gp1bb	glycoprotein Ib (platelet), beta polypeptide
NP_476489	Sept2	septin 2
NP_620184	Gorasp2	golgi reassembly stacking protein 2
NP_787032	Eef1a1	eukaryotic translation elongation factor 1 alpha 1
XP_213393		similar to CGI-125 protein
XP_213413		similar to H5
XP_213922		similar to Brain protein 44 (0-44 protein)
XP_216170		similar to Hypothetical protein MGC59076
XP_217147		similar to mmDj4
XP_219498		similar to serine/threonine kinase 29
XP_223227		similar to hypothetical protein FLJ10849
XP_231118		similar to kynurenine aminotransferase/glutamine transaminase K
XP_234516		similar to Cyclin K
XP_340794		similar to TBC1 domain, member 8; BUB2-like protein 1; vasc. Rab-GAP/TBC-containing
XP_343156		similar to protein inhibitor of activated STAT gamma
XP_343275		similar to myosin phosphatase targeting subunit 3 MYPT3

Community 9: Nuclear hormone receptors

NP_036787	Sp1	sp1 transcription factor
NP_036804	Thrb	thyroid hormone receptor beta
NP_036937	Rxra	retinoid X receptor alpha
NP_071516	Hnf4a	hepatocyte nucleus factor 4 alpha
NP_077335	Hif1a	hypoxia inducible factor 1, alpha subunit
NP_077364	Nr4a1	nucleus receptor subfamily 4, group A, member 1
NP_112392	Nr2f1	nucleus receptor subfamily 2, group F, member 1
NP_113815	Nr1h3	nucleus receptor subfamily 1, group H, member 3
NP_446294	Mapk1	mitogen activated protein kinase 1
NP_695209	Cops2	COP9 (constitutive photomorphogenic) homolog, subunit 2
XP_216720		similar to Thyroid transcription factor 1 (Thyroid nucleus factor 1) (TTF-1)
XP_226076		similar to putative WDC146
XP_233944		similar to nucleus receptor co-activator

Community 10: Sarcoglycans

NP_001006994	Sgcg	gamma sarcoglycan
NP_036830	Dmd	dystrophin isoform Dp71a
XP_213891		similar to abnormal spindle
XP_220884		similar to alpha-sarcoglycan

XP_223355		similar to beta-sarcoglycan

Community 11: Karyopherins

NP_037347	Akr7a3	aldo-keto reductase family 7, member A3 (aflatoxin aldehyde reductase)
NP_058759	Kpnb1	karyopherin (importin) beta 1
NP_058999	Kcnab1	potassium voltage-gated channel, shaker-related subfamily, beta member 1
NP_059000	Kcnab2	potassium voltage-gated channel, shaker-related subfamily, beta member 2
NP_942021	Kpna1	karyopherin alpha 1 (importin alpha 5)

Community 12: Hypoxia inducable factor

NP_077335	Hif1a	hypoxia inducible factor 1, alpha subunit
NP_077338	Arntl	aryl hydrocarbon receptor nucleus translocator-like
XP_234728		similar to Hspca protein
XP_234791		similar to heat shock protein 84 - mouse

Community 13: Peroxisomal proteins

NP_742060	Pex14	peroxisomal membrane anchor protein
XP_218778		similar to peroxisomal PTS2 receptor
XP_223684		similar to Peroxisomal membrane protein PEX13 (Peroxin-13)
XP_232343		similar to Pex5 protein

Community 14: Cell cycle regulation

NP_001009470	MGC108931	similar to cyclin B2
NP_741990	Cdc20	cell division cycle 20 homolog
NP_955795	Cdk2	cyclin-dependent kinase 2
XP_213222		similar to membrane-associated tyrosine-and threonine-spec. cdc2-inhibitory kinase
XP_214152		similar to cyclin-dependent kinase inhibitor 3; CDK2-assoc. dual spec. phosphatase
XP_235722		similar to cell division cycle 2 homolog 2; cell division cycle 2-like 2

Community 15: VEGF

NP_037194	Kdr	kinase insert domain protein receptor
NP_113949	Figf	c-fos induced growth factor
NP_114024	Vegfa	vascular endothelial growth factor
NP_446047	Pgf	placental growth factor
NP_446105	Vegfc	vascular endothelial growth factor C

Community 16: JAK/STAT cascade

NP_058790	Ghr	growth hormone receptor
NP_071775	Stat5b	signal transducer and activator of transcription 5B
NP_113702	Jak2	Janus kinase 2
NP_116001	Stat1	signal transducer and activator of transcription 1

Community 17: Karyopherin docking complex

NP_942021	Kpna1	karyopherin alpha 1 (importin alpha 5)
XP_215550		similar to polymyositis scleroderma overlap syndrome (PM-SCL) antigen 1 a
XP_216949		similar to putative exosome complex exonuclease RRP41
XP_218343		similar to DNA segment, Chr 7, Wayne State University 180, expressed
XP_233673		similar to P100 polymyositis-scleroderma overlap syndrome assoc. autoantigen homolog

Community 18: NF-kappaB regulation

NP_445807	Ikbkb	inhibitor of kappa light polypeptide gene enhancer in B-cells, kinase beta
NP_954534	Ikbkg	inhibitor of kappa light polypeptide gene enhancer in B-cells, kinase gamma
XP_219857		similar to conserved helix-loop-helix ubiquitous kinase
XP_234728		similar to Hspca protein
XP_234791		similar to heat shock protein 84 - mouse
XP_340919		similar to NF-kappaB inducing kinase

Community 19: Calmodulin

NP_036650	Calm3	calmodulin 3
NP_037220	Ptpn11	protein tyrosine phosphatase, non-receptor type 11
XP_213368		similar to Eukaryotic translation initiation factor 5A (eIF-5A) (eIF-4D) (Rev-binding)

XP_214050		similar to leucine aminopeptidase
XP_341110		similar to testis-enriched protein tyrosine phosphatase

Community 20: Actinins

NP_112267	Actn1	actinin, alpha 1
NP_113863	Actn4	alpha actinin 4
NP_653346	Gpsm1	G-protein signalling modulator 1 (AGS3-like, C. elegans)
XP_214499		similar to actinin, alpha 2
XP_216586		similar to Msx-2 interacting nucleus target protein

Community 21: ATP transporter proteins

NP_036686	Eno1	enolase 1, alpha
NP_445954	Abcg1	ATP-binding cassette, sub-family G (WHITE), member 1
XP_214583		similar to grp75
XP_341267		similar to polynucleotide phosphorylase-like protein

Community 22: Tubulin proteins

NP_036942	Sycp1	synaptonemal complex protein 1
NP_112286	Dbn1	drebrin 1
NP_665721	Tubg1	tubulin, gamma 1
NP_954534	Ikbkg	inhibitor of kappa light polypeptide gene enhancer in B-cells, kinase gamma
XP_215080		similar to RIKEN cDNA 3230401O13
XP_219470		similar to tubulin, gamma complex associated protein 2
XP_225013		similar to Gamma-tubulin complex 3 (GCP-3) (Spindle pole body Spc98 homol.)

Community 23: Actinin/calmodulin

NP_036650	Calm3	calmodulin 3
NP_059015	Grp58	glucose regulated protein, 58 kDa
NP_112267	Actn1	actinin, alpha 1
NP_113863	Actn4	alpha actinin 4
XP_213891		similar to abnormal spindle
XP_214499		similar to actinin, alpha 2
XP_343034		similar to dystrobrevin B (mDTN-B)

Community 24: Proteasome

NP_001005875	Psmd12	proteasome 26S non-ATPase subunit 12
XP_226439		similar to 26S proteasome non-ATPase regulatory subunit 7 (26S proteasome reg. subunit)
XP_230377		similar to TRAF6
XP_343114		similar to CD40 receptor associated factor 1
XP_344977		similar to 26S proteasome subunit p40.5

Community 25: Serine protease inhibitors

NP_001007619	Rchy1	ring finger and CHY zinc finger domain containing 1
NP_001007733	MGC94010	similar to SPI6
NP_036630	Aldr1	aldehyde reductase 1 (low Km aldose reductase) (5.8 kb PstI fragment)
NP_037215	Hspa5	heat shock 70kD protein 5
NP_059036	Acox1	acyl-Coenzyme A oxidase 1, palmitoyl
NP_067728	Serpinb2	serine (or cysteine) proteinase inhibitor, clade B, member 2
NP_075218	Kcnip1	potassium channel interacting protein 1
NP_113815	Nr1h3	nucleus receptor subfamily 1, group H, member 3
NP_446231	Serpini1	serine (or cysteine) proteinase inhibitor, clade I (neuroserpin), member 1
NP_476449	Serpinb5	serine (or cysteine) proteinase inhibitor, clade B, member 5
NP_543169	Pde11a	phosphodiesterase 11A
NP_596897	Agpat4	1-acylglycerol-3-phosphate O-acyltransferase 4
NP_741984	Spna2	alpha-spectrin 2
NP_742005	Canx	calnexin
NP_742026	Eif2b1	eukaryotic translation initiation factor 2B, subunit 1 alpha
NP_954888	Rela	v-rel reticuloendotheliosis viral oncogene homolog A
XP_213076		similar to GTP-binding protein NGB
XP_233568		similar to Zinc finger protein 436
XP_341644		similar to Polymerase (RNA) II (DNA directed) polypeptide C

Community 26: Myosin

NP_036650	Calm3	calmodulin 3
NP_058936	Myh7	myosin heavy chain, polypeptide 7
XP_218617		similar to nonmuscle myosin heavy chain
XP_340818	Myh4_pred	myosin, heavy polypeptide 4; now: XP_340819, similar to Myh4
XP_343512		similar to leucyl-tRNA synthetase

Community 27: Nucleoporin

NP_036922	Dspp	dentin sialophosphoprotein
XP_218620		similar to nucleus pore glycoprotein p62 (62 kDa nucleoporin)
XP_219265		similar to importin 7
XP_341509		nucleoporin 153kD

Community 28: Laminin

NP_112406	Actb	cytoplasm beta-actin
NP_113708	Myh10	myosin heavy chain 10, non-muscle
XP_218617		similar to nonmuscle myosin heavy chain
XP_225550		similar to RIKEN cDNA 2310068O22
XP_228209		similar to Lama4 protein

Community 29: Matrix metalloproteases

NP_037215	Hspa5	heat shock 70kD protein 5
NP_077376	Adamts1	a disintegrin and metalloproteinase with thrombospondin motifs 1
NP_446301	Erp70	protein disulfide isomerase related protein (calcium-binding, intestinal-rel.)
XP_343193		Now NP_001012197 Tra1_predicted tumor rejection antigen gp96 (predicted)

Community 30: Casein kinase

NP_036654	Cbs	cystathionine beta synthase
NP_074046	Csnk1g3	casein kinase 1, gamma 3
XP_213368		similar to Eukaryotic translation initiation factor 5A (eIF-5A, eIF-4D) (Rev-binding)
XP_343107		similar to Vrk1 protein

Community 31: Zinc finger protein

NP_062566	Znf386	zinc finger protein 386 (Kruppel-like)
NP_665887	Lhx1	LIM homeobox protein 1
NP_954525	MGC73008	Unknown (protein for MGC:73008)
XP_342746		hypothetical protein XP_342745

Community 32: Actinin

NP_112267	Actn1	actinin, alpha 1
NP_113863	Actn4	alpha actinin 4
NP_775148	Pdlim7	PDZ and LIM domain 7
XP_214499		similar to actinin, alpha 2
XP_235710		similar to Tenc1 protein

Community 33: Breast cancer anti-estrogen resistance

NP_037063	Bcar1	breast cancer anti-estrogen resistance 1
NP_037213	Ptk2	PTK2 protein tyrosine kinase 2
XP_217250	Ephb1	PREDICTED: Eph receptor B1 (Tyrosine-protein kinase receptor EPH-2)
XP_223781		similar to Vinculin (Metavinculin)
XP_233522		similar to Eph-like receptor tyrosine kinase
XP_341935		transforming growth factor beta 1 induced transcript 1
XP_343143		similar to ring finger protein 41; hypothetical SBBI03 protein

Community 34: Breast cancer anti-estrogen resistance

NP_036890	Syk	spleen tyrosine kinase
NP_037063	Bcar1	breast cancer anti-estrogen resistance 1
NP_067719	Erbb4	v-erb-a erythroblastic leukemia viral oncogene homolog 4
XP_223781		similar to Vinculin (Metavinculin)

XP_341935		transforming growth factor beta 1 induced transcript 1

Community 35: Protein-tyrosine kinases

NP_036887	fyn	fyn proto-oncogene
NP_037137	Pik3r1	phosphatidylinositol 3-kinase, regulatory subunit, polypeptide 1
NP_037213	Ptk2	PTK2 protein tyrosine kinase 2
XP_217250		similar to Ephrin type-B receptor 1 precursor (Tyrosine-protein kinase receptor EPH-2)
XP_342283		SHC (Src homology 2 domain-containing) transforming protein 1

Community 36: Fyn proto-oncogene

NP_036887	fyn	fyn proto-oncogene
NP_569089	Khdrbs1	src associated in mitosis, 68 kDa
XP_232763	Lck	lymphocyte-specific protein tyrosine kinase
XP_343333		similar to SMARCD1 protein
