# Supplementary material for: Cluster analysis of networks generated through homology: automatic identification of important protein communities involved in cancer metastasis
Source: BMC Bioinformatics. 2006 Jan 6;7:2. doi: 10.1186/1471-2105-7-2 (PMC1363365; doi:10.1186/1471-2105-7-2)
Supplement: Additional File 2 — Microarray data. The microarray expression data used in this study. [file 1471-2105-7-2-S2.pdf]

Supplementary material – Microarray data

Pall F. Jonsson, Tamara Cavanna, Daniel Zicha and Paul A. Bates: Cluster analysis of networks generated through homology: automatic identification of important protein communities involved in cancer metastasis. BMC Bioinformatics, 2005.

RefSeq sequence ID	Expression Fold
NP_001000100	-1
NP_001000284	1.032130484362528
NP_001000387	-1
NP_001000583	1.225547584056809
NP_001000716	1.05583672789378
NP_001000723	0.9391161759167254
NP_001000942	1.2352992097103224
NP_001000983	-1
NP_001001515	0.7271615816290821
NP_001001516	1.207633294311901
NP_001001718	1.2062492814227743
NP_001002016	0.9299693526401964
NP_001002023	0.40473335097489477
NP_001002289	1.0066218430707103
NP_001002802	-1
NP_001002807	1.4000012014078367
NP_001002809	1.023046934624517
NP_001002813	2.1504231635059234
NP_001002827	1.0717381436542013
NP_001002831	1.2733332966724527
NP_001002851	-1
NP_001002855	-1
NP_001003401	-1
NP_001003673	0.8639216742206198
NP_001003708	1.0565792331569879
NP_001003975	-1
NP_001003978	0.8684366883679582
NP_001004021	-1
NP_001004022	1.1457632542161185
NP_001004078	0.9147065029314764
NP_001004080	0.9941397426883285
NP_001004081	0.7754775203031948
NP_001004084	1.2401554762053506
NP_001004085	0.7810476582376338
NP_001004086	0.726932481672724
NP_001004089	0.7655280876635647
NP_001004090	-1
NP_001004091	0.8942064496043248
NP_001004199	1.1394324920964143
NP_001004200	0.8752596714907356
NP_001004201	-1
NP_001004202	1.579327109177539
NP_001004203	1.2194189242792164
NP_001004204	1.0262890502809865
NP_001004206	0.9669321291943462
NP_001004207	1.1709069628193476
NP_001004209	1.6251681331310086
NP_001004210	1.06332026696191
NP_001004211	0.7877674367116082
NP_001004214	-1
NP_001004215	0.2179147945468516
NP_001004218	0.9416187410379493
NP_001004219	1.222088358394957
NP_001004220	0.47738310324019056
NP_001004222	1.0239973270686784

NP_001004224	1.05817151841425
NP_001004225	0.8020383685484512
NP_001004226	0.8936733905171667
NP_001004227	0.927812547903179
NP_001004228	0.8178848156605549
NP_001004230	0.8956154285212367
NP_001004231	0.5126196743326804
NP_001004233	1.3650588585293628
NP_001004234	0.8626621798055859
NP_001004235	0.9188403478419869
NP_001004237	0.9584247765562678
NP_001004239	1.0427409712494673
NP_001004241	0.9194484037886043
NP_001004242	-1
NP_001004243	0.9116494435918829
NP_001004244	0.8460068191469773
NP_001004245	0.8035140144482419
NP_001004247	0.9172991567433361
NP_001004248	1.0796219166107417
NP_001004249	0.4234021437308996
NP_001004250	1.0232952117440395
NP_001004252	0.9451208171651985
NP_001004258	0.8492060122022229
NP_001004260	-1
NP_001004275	1.211269984888257
NP_001004279	1.2172026917633163
NP_001004280	0.9323810262876835
NP_001004283	0.9223973827137512
NP_001004424	1.1118579477398682
NP_001004442	0.7745777742758886
NP_001004446	1.1857598794418098
NP_001005246	-1
NP_001005383	1.110655871184064
NP_001005529	1.1194731959546107
NP_001005533	0.8879186659005591
NP_001005537	1.0596972115029473
NP_001005538	1.3151882696768242
NP_001005539	2.036410471223044
NP_001005540	-1
NP_001005541	1.0998861975642882
NP_001005543	0.7872589316029087
NP_001005545	1.00408261365185
NP_001005546	1.0517020492897513
NP_001005547	0.9458535621491881
NP_001005548	0.8703269958117619
NP_001005550	1.030550111819295
NP_001005551	3.693647930087434
NP_001005552	0.9935870022290351
NP_001005553	1.2254742821791198
NP_001005554	0.9513986407922072
NP_001005555	1.2305822169021605
NP_001005558	-1
NP_001005560	0.96402550186352
NP_001005561	1.318542736036963
NP_001005563	0.9648001435568769
NP_001005564	0.9910412860786063
NP_001005765	1.0153970170101936
NP_001005871	1.4318686030930818
NP_001005872	1.1227444850077852
NP_001005876	0.8603454661799516
NP_001005878	1.405519258344737
NP_001005885	-1

NP_001005891	1.8849271273486654
NP_001005902	1.1070588308456224
NP_001005903	0.9770933480533822
NP_001005905	1.2601827594589727
NP_001005906	0.6392983629501648
NP_001005908	1.1023469219760633
NP_001006610	-1
NP_001006953	0.7753195066170172
NP_001006955	1.1660830054652183
NP_001006956	1.3715775199339681
NP_001006959	1.2017772183661697
NP_001006961	0.7868720235116041
NP_001006964	0.9652847857909697
NP_001006965	0.7887987412514862
NP_001006966	0.7213011760909795
NP_001006967	0.8449580711427251
NP_001006968	1.0908404121182083
NP_001006969	1.039563906496282
NP_001006970	0.9763745726762615
NP_001006971	1.0265680122129106
NP_001006973	0.9487305216266696
NP_001006974	0.8734901388683545
NP_001006975	1.153120150393184
NP_001006977	0.7778543087985965
NP_001006979	0.6378928777122892
NP_001006982	1.0774273499531057
NP_001006983	1.054899169405582
NP_001006984	0.8386553073500597
NP_001006985	1.4373800583798404
NP_001006986	0.8638614914729227
NP_001006987	1.1119904669565928
NP_001006992	1.3929643298498566
NP_001006993	0.4347489964031958
NP_001006996	1.0425598019868663
NP_001006998	0.9687437136420073
NP_001007001	0.7624172208197781
NP_001007004	1.2589938599042088
NP_001007005	0.7366497663824947
NP_001007008	0.9842481962576293
NP_001007009	-1
NP_001007145	1.1630704718669185
NP_001007146	0.9690916135600879
NP_001007149	0.8728646525439278
NP_001007150	0.7571332794523906
NP_001007236	0.9290204510469491
NP_001007600	1.1450653795591628
NP_001007601	1.2057156325500502
NP_001007602	-1
NP_001007603	1.0586607293317076
NP_001007605	1.0818919873970627
NP_001007606	1.408629550571323
NP_001007609	0.9271772021232617
NP_001007611	0.8993389136054272
NP_001007614	1.090072447869259
NP_001007616	0.9465430606055422
NP_001007621	1.0175964721188557
NP_001007622	0.7008966811596334
NP_001007623	0.7116345003021115
NP_001007625	1.2344396026065112
NP_001007629	0.9290750027310765
NP_001007630	0.9654704871943515
NP_001007634	0.9004541143508704

NP_001007635	0.9870124512094982
NP_001007637	1.9174563292389404
NP_001007638	0.7856657426530864
NP_001007648	1.039880585517269
NP_001007649	1.1041799750683738
NP_001007650	1.0622641000741224
NP_001007652	0.9417354830553939
NP_001007654	0.7676278871222396
NP_001007655	1.0609542371574603
NP_001007656	0.9494114173616199
NP_001007660	0.8202443962112689
NP_001007664	0.8408496022995865
NP_001007666	0.8748500601305967
NP_001007667	0.9502276335318934
NP_001007668	0.9464165665928973
NP_001007671	-1
NP_001007676	0.5705590041410159
NP_001007677	0.9113992185721265
NP_001007678	-1
NP_001007681	1.610349207963374
NP_001007684	0.5453114616700651
NP_001007685	1.0822813325955085
NP_001007690	0.7989697479427571
NP_001007692	0.9073395247267336
NP_001007695	1.4907533177796244
NP_001007696	-1
NP_001007697	0.6825953547072178
NP_001007702	1.132852502333679
NP_001007709	1.2663692586312634
NP_001007710	1.0614579817785823
NP_001007715	0.9864664690545039
NP_001007716	1.3174774317777898
NP_001007724	0.8037015277924769
NP_001007726	0.9557789676244033
NP_001007730	1.5125819055240683
NP_001007733	1.9105645445816481
NP_001007734	0.7370989422885263
NP_001007735	0.9068543806932688
NP_001007736	0.8900970014661388
NP_001007740	0.9507139633995841
NP_001007741	1.3096197479625078
NP_001007743	0.846616564418811
NP_001007744	-1
NP_001007750	1.0710534816338566
NP_001007754	0.7471386062630286
NP_001007757	0.8915505535896794
NP_001007759	0.8118460758364406
NP_001007800	1.297498652915413
NP_001007802	0.402208030312879
NP_001007804	0.8515296721848675
NP_001007805	0.9323266534373408
NP_001008218	0.9037654481831482
NP_001008278	0.9601756985586091
NP_001008279	1.5254224490139114
NP_001008280	1.13912472214294
NP_001008282	0.9889030226311663
NP_001008283	0.5144369059441162
NP_001008287	0.7768966480787709
NP_001008289	1.0825547362883787
NP_001008290	1.0306119851694346
NP_001008291	0.8734431220212723
NP_001008293	0.9682230892557122

NP_001008300	0.9470288073602248
NP_001008303	1.2284880013484747
NP_001008304	0.9754252552446433
NP_001008305	0.937352683552746
NP_001008306	0.8710853183536096
NP_001008308	0.6837373394544012
NP_001008309	1.255782451850499
NP_001008310	0.9453736344765685
NP_001008313	1.282557802983389
NP_001008314	0.8464352697024684
NP_001008315	1.0723908954021362
NP_001008319	0.9364669651750769
NP_001008322	1.4335424573751192
NP_001008323	0.9167846543260271
NP_001008324	0.8101467262257273
NP_001008325	1.054251621393351
NP_001008326	0.9883307361421432
NP_001008327	0.6168403420114749
NP_001008328	0.8942953795350682
NP_001008330	1.085505638210098
NP_001008331	0.8166202905190323
NP_001008334	1.4613174011718149
NP_001008336	1.067568735577406
NP_001008340	0.9366041456859256
NP_001008342	-1
NP_001008343	0.9191736771726218
NP_001008344	0.9571310179937434
NP_001008350	1.3527134068149245
NP_001008351	0.9878020072178922
NP_001008353	0.9527891231384719
NP_001008359	1.6829890918346624
NP_001008361	0.846093180981136
NP_001008362	1.1668667990869677
NP_001008368	0.8958210572679763
NP_001008369	0.9330836518638758
NP_001008370	1.048640807126965
NP_001008371	0.6701486475455035
NP_001008373	1.157175944848835
NP_001008377	0.7932102779139297
NP_001008381	1.0481159637142745
NP_001008385	1.5769570857344652
NP_001008507	0.9654801830464219
NP_001008510	1.3967300214458893
NP_001008514	-1
NP_001008515	1.1676864626721775
NP_001008519	0.8313109948165057
NP_001008520	0.5606146417916564
NP_001008524	1.1549014796759407
NP_001008525	0.8243907934243951
NP_001008526	1.2319776957239816
NP_001008553	0.8184778249571103
NP_001008557	-1
NP_001008558	-1
NP_001008562	0.8148974799608947
NP_001008694	0.55665112727911
NP_001008721	1.0215530765283067
NP_001008724	-1
NP_001008725	1.0016431784829418
NP_001008751	-1
NP_001008759	-1
NP_001008764	1.0530961595729285
NP_001008766	0.8302617748693842

NP_001008767	0.2997500484046876
NP_001008768	1.0291988093211557
NP_001008770	3.1308360267630992
NP_001008775	1.1531695165923739
NP_001008776	-1
NP_001008802	-1
NP_001008804	1.12244586447785
NP_001008817	-1
NP_001008823	-1
NP_001008826	1.1624466963648703
NP_001008829	1.1523434346858348
NP_001008831	1.5887244886797722
NP_001008835	0.9102111581851379
NP_001008836	1.301007104374525
NP_001008838	1.3138703166926609
NP_001008839	1.4019898830785877
NP_001008843	1.3522669178886488
NP_001008845	1.0811508647003274
NP_001008847	1.1895088366682318
NP_001008855	1.2184891573222583
NP_001008879	1.1184112292508497
NP_001008884	1.1135798105418315
NP_001008887	1.0156790050519644
NP_001008888	0.9719144404793699
NP_001008890	-1
NP_001008893	1.0119589126853241
NP_001009180	1.807316163564239
NP_001009239	1.15719148057267
NP_001009246	1.044636890661804
NP_001009255	0.8745199738943814
NP_001009268	1.1500404528701103
NP_001009271	0.47945804228176797
NP_001009275	0.7837630470569503
NP_001009283	-1
NP_001009290	1.2109648208160244
NP_001009316	1.0416517511647867
NP_001009344	0.6643725012883381
NP_001009369	0.9554864072495679
NP_001009416	1.0715878216533834
NP_001009419	0.9777988467674027
NP_001009422	0.5415055384686058
NP_001009430	0.5188157977851201
NP_001009455	1.2371666185934178
NP_001009470	0.9931330424694501
NP_001009480	0.7778071146630503
NP_001009494	1.404455821963027
NP_007225	1.542237636404722
NP_007226	1.0706192694181027
NP_007227	0.9625522312739442
NP_007228	1.1532631483452234
NP_007232	1.1059290584205101
NP_007234	1.3004018446973813
NP_007237	1.19358175349372
NP_036620	-1
NP_036621	0.6942723389794754
NP_036622	1.1642062939911277
NP_036623	-1
NP_036624	1.5837569167549124
NP_036625	-1
NP_036626	1.3644124222817668
NP_036627	1.066509402348176
NP_036628	-1

NP_036629	0.4023412572273299
NP_036630	1.0738067529550677
NP_036631	1.226133616378822
NP_036632	1.1918084839164667
NP_036633	-1
NP_036634	1.1127387500822605
NP_036635	1.0381386861508386
NP_036636	1.5437918605512664
NP_036637	1.1010370503379088
NP_036638	1.1822888376234388
NP_036639	-1
NP_036640	1.149057601150624
NP_036642	-1
NP_036643	1.1820009728284406
NP_036644	1.246863824374277
NP_036645	0.5355420303265356
NP_036646	1.3268772941844997
NP_036647	0.9799538991539355
NP_036648	-1
NP_036649	1.209176072769881
NP_036651	1.291013634410233
NP_036652	1.1188393700188304
NP_036653	-1
NP_036654	1.0594525952966478
NP_036655	1.8190339527104011
NP_036656	-1
NP_036658	0.8094934304614871
NP_036659	3.0600615353889453
NP_036660	-1
NP_036661	0.8703882095392784
NP_036662	1.1333061018335906
NP_036663	0.8629919204930006
NP_036664	0.6187177075740025
NP_036665	-1
NP_036666	-1
NP_036667	-1
NP_036668	1.7285197010102162
NP_036670	-1
NP_036672	-1
NP_036673	-1
NP_036674	-1
NP_036675	1.4450068399153049
NP_036676	-1
NP_036677	1.0157726032172867
NP_036678	-1
NP_036679	0.8966834734074247
NP_036680	0.9833420648095714
NP_036681	-1
NP_036682	1.5851727278409076
NP_036683	0.4662807926857402
NP_036684	-1
NP_036685	-1
NP_036686	1.0191011754739536
NP_036687	0.9983316999254165
NP_036688	-1
NP_036689	1.0810695761859441
NP_036690	-1
NP_036691	0.5773254059354876
NP_036692	-1
NP_036693	0.4161126215297359
NP_036694	0.8406844967831844
NP_036695	-1

NP_036696	1.0648099354440044
NP_036697	1.0536213534710825
NP_036698	-1
NP_036699	1.0769321908131515
NP_036700	-1
NP_036701	0.7927115006637498
NP_036702	1.4413473850660359
NP_036703	1.048838454262486
NP_036704	-1
NP_036705	1.2093249832848671
NP_036706	1.448203907216065
NP_036707	1.1037136188912544
NP_036708	1.1013183814923952
NP_036709	1.530724096774328
NP_036710	0.3183354947823467
NP_036711	-1
NP_036712	1.791011552014
NP_036713	1.237273829269442
NP_036714	-1
NP_036715	0.865882239104344
NP_036716	1.2368695476367875
NP_036717	-1
NP_036718	-1
NP_036719	-1
NP_036720	0.9980912100802406
NP_036721	0.3575521226031837
NP_036722	0.9593478654674426
NP_036723	1.1901308750644117
NP_036724	0.5192122872550047
NP_036725	-1
NP_036726	-1
NP_036727	0.4014752577118021
NP_036728	0.6195264192002462
NP_036729	-1
NP_036730	1.4797119634158218
NP_036731	-1
NP_036732	0.90616405660986
NP_036733	0.5931411440480243
NP_036735	0.9659456568247675
NP_036736	-1
NP_036737	0.6180444911069239
NP_036738	0.5230738181050695
NP_036739	-1
NP_036740	0.7828753776872821
NP_036741	1.102933970537723
NP_036742	-1
NP_036743	1.5822795208108515
NP_036744	-1
NP_036745	-1
NP_036746	0.941759752840872
NP_036747	0.8015671664858456
NP_036748	2.10955886512318
NP_036749	-1
NP_036750	1.149879626834695
NP_036751	-1
NP_036752	0.8845264953278473
NP_036753	0.7988206674554664
NP_036755	1.3289640455631802
NP_036756	-1
NP_036757	1.1815070666560528
NP_036758	-1
NP_036759	1.1964998866400267

NP_036760	1.1755501017121117
NP_036761	-1
NP_036762	1.0864392843289132
NP_036763	2.159030788084101
NP_036764	0.7527164789468608
NP_036765	1.0663267147910707
NP_036766	1.0907858325595623
NP_036767	-1
NP_036768	1.618195499992548
NP_036769	1.025051655485252
NP_036771	1.2178534163838273
NP_036772	0.28003154949409426
NP_036773	1.047624264095437
NP_036774	1.0898758188201632
NP_036775	-1
NP_036777	1.1105855690184312
NP_036778	3.5623211712043896
NP_036779	-1
NP_036780	1.1372696536189264
NP_036781	1.3631099271689326
NP_036782	-1
NP_036783	1.3939771974504573
NP_036784	1.0637158251081171
NP_036785	-1
NP_036786	1.00679010884026
NP_036787	1.1834187135375391
NP_036788	0.7152917813815958
NP_036789	-1
NP_036791	-1
NP_036792	-1
NP_036793	1.1100008183966377
NP_036794	-1
NP_036795	1.4691321795751024
NP_036796	1.0938685059829443
NP_036797	-1
NP_036798	-1
NP_036799	1.1575387975437568
NP_036800	-1
NP_036801	-1
NP_036802	1.252686796691534
NP_036803	3.014263461038766
NP_036804	1.1893764597743361
NP_036805	0.34367061552678707
NP_036806	1.212805591463844
NP_036807	-1
NP_036808	-1
NP_036809	-1
NP_036810	1.1837751691965264
NP_036812	1.214830370976249
NP_036813	1.2275198404439225
NP_036814	-1
NP_036816	1.2336622508416328
NP_036817	0.9151596938593836
NP_036818	1.2590395697263443
NP_036819	1.3348040947615163
NP_036820	-1
NP_036821	-1
NP_036822	1.4098862187787529
NP_036824	0.9834778732052932
NP_036825	-1
NP_036826	1.0900183901514064
NP_036827	0.711436741627487

NP_036828	-1
NP_036829	-1
NP_036831	1.674166750549155
NP_036832	-1
NP_036833	1.9355908939279878
NP_036834	-1
NP_036835	1.2429748122609388
NP_036836	-1
NP_036837	1.1676965661461653
NP_036838	1.0577378939957598
NP_036839	-1
NP_036840	1.8073828496504512
NP_036842	-1
NP_036843	-1
NP_036845	1.5093703763221589
NP_036846	-1
NP_036847	1.291045972032225
NP_036848	1.031751970677334
NP_036849	0.6652445605815466
NP_036850	-1
NP_036851	1.1103368265611315
NP_036852	-1
NP_036853	1.1684803227411251
NP_036855	-1
NP_036856	0.8274060780364584
NP_036857	-1
NP_036858	1.1327675219741353
NP_036859	1.081558063202127
NP_036860	-1
NP_036861	-1
NP_036862	-1
NP_036863	-1
NP_036864	0.8755483959124831
NP_036865	0.7703123545779551
NP_036866	1.2164491585050987
NP_036867	1.4178078901342532
NP_036868	0.7917035600857303
NP_036869	-1
NP_036870	-1
NP_036871	-1
NP_036872	1.1722741091602469
NP_036873	-1
NP_036874	-1
NP_036875	1.3143820540560591
NP_036876	1.2296168903984588
NP_036877	1.277280730774837
NP_036878	-1
NP_036879	0.7959560269400665
NP_036880	1.0224873058024573
NP_036881	2.4894776713224305
NP_036882	1.1577988961226593
NP_036883	0.824841206741062
NP_036884	2.478914178927263
NP_036885	-1
NP_036886	1.421107648833491
NP_036887	1.8474185668462009
NP_036888	0.995887944013541
NP_036889	-1
NP_036890	1.303353222184901
NP_036891	1.7460014550096927
NP_036892	-1
NP_036893	-1

NP_036894	1.9176857014476443
NP_036895	0.9505722384094478
NP_036896	1.1457494222083293
NP_036897	1.0034839993697908
NP_036898	0.786228423988281
NP_036899	1.0526890437525143
NP_036900	-1
NP_036901	1.0918170075897553
NP_036902	-1
NP_036903	1.534140862930338
NP_036906	1.334777439753534
NP_036907	2.7256537659051223
NP_036908	1.153250196216786
NP_036909	0.21958158329955957
NP_036910	2.59135941998107
NP_036911	-1
NP_036912	0.7225834589780794
NP_036913	0.7751488561252878
NP_036915	0.7940468198440871
NP_036916	1.0000776556584923
NP_036917	1.2886265550918248
NP_036918	1.073586379123433
NP_036920	0.7965615402524833
NP_036921	1.0593168555024415
NP_036922	1.2217674705772168
NP_036923	1.0613362937825588
NP_036924	-1
NP_036925	0.6811352549179431
NP_036926	-1
NP_036927	-1
NP_036928	1.1099953725140892
NP_036929	1.197390845122878
NP_036930	-1
NP_036931	-1
NP_036932	-1
NP_036933	1.3278210486361164
NP_036935	-1
NP_036936	0.9865932588426604
NP_036937	-1
NP_036938	1.1215287125836582
NP_036939	0.5375410383414729
NP_036942	1.6121900135516387
NP_036943	1.566358846235942
NP_036944	3.261781398762175
NP_036945	-1
NP_036947	1.5210951531279253
NP_036948	1.0955456460366182
NP_036949	-1
NP_036950	-1
NP_036951	0.8767786655848279
NP_036952	1.3578548654358455
NP_036953	1.151065899954549
NP_036954	1.0998663487834965
NP_036955	1.4104722376888852
NP_036956	0.774975100006189
NP_036957	-1
NP_036958	1.1347267189016064
NP_036959	1.025795821962699
NP_036960	0.8701425676030521
NP_036961	1.0481716003669879
NP_036962	1.6130097379167174
NP_036964	-1

NP_036965	1.3255096354788574
NP_036966	0.6544811725057648
NP_036967	-1
NP_036968	0.8421518401200951
NP_036969	1.077591829827537
NP_036970	0.928320128439629
NP_036971	0.9557341374148081
NP_036972	-1
NP_036973	-1
NP_036974	0.9791572905383571
NP_036975	1.5866858914879973
NP_036976	0.43305826018656024
NP_036977	0.9718115560324128
NP_036978	1.0245827877008253
NP_036979	1.2319693734040036
NP_036980	1.3638840761443347
NP_036981	-1
NP_036982	1.055063768599446
NP_036983	1.1175901782052358
NP_036984	-1
NP_036985	1.6880547489202848
NP_036986	-1
NP_036987	0.6198193721984225
NP_036988	-1
NP_036989	1.0721312396084466
NP_036990	0.9844757622098408
NP_036991	1.8740017111103093
NP_036992	-1
NP_036993	0.5221871620182938
NP_036994	0.7194480351904554
NP_036995	-1
NP_036996	-1
NP_036997	1.125462932481018
NP_036998	1.2686373410537888
NP_036999	1.1600730219060493
NP_037000	-1
NP_037001	1.2006202363098242
NP_037002	-1
NP_037003	-1
NP_037004	-1
NP_037005	-1
NP_037006	0.8107537984525516
NP_037007	1.0837575152082015
NP_037008	1.1133236416003924
NP_037009	-1
NP_037010	-1
NP_037011	-1
NP_037012	0.7671654706197963
NP_037013	4.517390823930934
NP_037014	-1
NP_037015	-1
NP_037016	1.1334984574524452
NP_037018	1.4831973954081357
NP_037019	1.2416026399522746
NP_037020	-1
NP_037021	0.5802015994572206
NP_037022	1.153064125240893
NP_037023	0.7996974963140132
NP_037024	1.0597259702258748
NP_037025	1.289055766276666
NP_037026	1.4309054915233197
NP_037027	0.878196301974886

NP_037028	-1
NP_037029	-1
NP_037030	0.9235649561025857
NP_037031	0.7066903302540544
NP_037032	-1
NP_037033	-1
NP_037034	-1
NP_037035	1.2153148514427308
NP_037036	1.2554161662366863
NP_037039	1.4570714586249052
NP_037040	1.139559780492073
NP_037041	-1
NP_037042	1.9018364440392290
NP_037043	1.848100763531219
NP_037044	0.9690293791167498
NP_037046	0.924902997352182
NP_037047	1.1242762898912788
NP_037048	-1
NP_037050	2.608617521093768
NP_037051	1.3106244413213388
NP_037052	-1
NP_037053	-1
NP_037054	0.9268112471414981
NP_037055	1.1202133997124422
NP_037056	1.890988423305731
NP_037057	1.782797223320035
NP_037058	-1
NP_037059	-1
NP_037060	-1
NP_037061	-1
NP_037062	-1
NP_037063	1.3404139561249968
NP_037064	-1
NP_037066	0.7258036982610013
NP_037067	0.6997289806015615
NP_037068	-1
NP_037069	-1
NP_037070	1.246272112321617
NP_037071	0.3741195728906712
NP_037072	0.6305912358667898
NP_037073	1.3283958622817655
NP_037074	1.0149255607671908
NP_037075	1.2625541160390323
NP_037076	-1
NP_037077	1.2286710013364763
NP_037078	0.8562977891686988
NP_037079	1.3873495810553127
NP_037080	0.9750738040594138
NP_037081	0.9553121162413846
NP_037082	1.218425962816564
NP_037083	1.7568778837452654
NP_037084	-1
NP_037085	1.7464189453684429
NP_037086	0.8607398663666316
NP_037087	1.2195405027854986
NP_037088	-1
NP_037089	-1
NP_037090	-1
NP_037091	-1
NP_037092	1.1324038326162318
NP_037093	-1
NP_037094	1.1571786363927463

NP_037095	0.9338137408432028
NP_037096	1.2068183721843981
NP_037097	-1
NP_037098	0.7881769539703498
NP_037099	0.7104311533706366
NP_037100	1.6299340659476036
NP_037101	1.2602934304608144
NP_037102	-1
NP_037103	-1
NP_037104	-1
NP_037105	-1
NP_037106	1.1421532995966759
NP_037107	1.032489104801833
NP_037108	-1
NP_037109	0.595716599643625
NP_037110	1.0631268621648329
NP_037112	0.8154030067975158
NP_037113	1.160173131881039
NP_037114	-1
NP_037115	1.3619772903119103
NP_037116	1.0710536084608542
NP_037117	0.9053761221564124
NP_037119	0.9539518457311953
NP_037120	0.5108879963488897
NP_037123	0.8296252942199632
NP_037124	0.8949069817801015
NP_037125	1.1560341193013177
NP_037126	1.1564248822082999
NP_037127	26.86382740569403
NP_037128	-1
NP_037129	-1
NP_037130	0.8590353300640152
NP_037131	1.1144630389624126
NP_037132	1.0457299243600624
NP_037133	1.3160702322375062
NP_037134	2.876828484392806
NP_037135	0.6263818339763777
NP_037136	1.4862221459420233
NP_037137	-1
NP_037138	1.2204172335967365
NP_037139	-1
NP_037140	-1
NP_037142	0.8507850279199634
NP_037143	1.345689549248618
NP_037144	-1
NP_037145	1.3388720861782601
NP_037146	1.1567382344315682
NP_037147	-1
NP_037148	1.6756122979955865
NP_037150	0.9731546566850513
NP_037151	1.013329358212485
NP_037153	0.4158033932065679
NP_037154	1.768046998497524
NP_037155	-1
NP_037156	2.7511849091745724
NP_037157	1.8874315448698846
NP_037158	1.8158028129946997
NP_037159	0.5360052135736179
NP_037160	1.6692837304963748
NP_037161	1.0711212319558756
NP_037162	0.9880130277016369
NP_037163	1.0958207154305728

NP_037164	1.0498989525632008
NP_037165	-1
NP_037166	1.5483776132909446
NP_037168	-1
NP_037169	0.6194042008783669
NP_037170	1.684111165402506
NP_037171	1.0273691216335101
NP_037172	1.0093704503114351
NP_037173	1.3608076203094273
NP_037174	0.8855175613149407
NP_037176	8.962689231553645
NP_037177	2.0105367140652692
NP_037178	1.142704213334554
NP_037179	0.748089429807781
NP_037180	-1
NP_037181	1.0360871765658617
NP_037182	0.9853932568509028
NP_037183	-1
NP_037184	1.1708390329611804
NP_037185	1.1315719861134295
NP_037187	1.1148881528222587
NP_037189	3.0490338157895067
NP_037190	1.1306391906569442
NP_037191	-1
NP_037192	1.138820589580204
NP_037193	-1
NP_037194	0.7385522041699217
NP_037195	1.0211536767880993
NP_037196	-1
NP_037197	1.0078770984387668
NP_037198	-1
NP_037199	1.0087668442213638
NP_037200	-1
NP_037201	1.3064388658937842
NP_037202	1.8901833382889255
NP_037203	0.4951616970524911
NP_037205	1.024323348047179
NP_037206	1.1272625458025776
NP_037207	0.9432283206993688
NP_037208	-1
NP_037209	-1
NP_037210	0.9928833093408468
NP_037211	0.3953410372403401
NP_037212	1.313383781501454
NP_037213	0.9652254699837087
NP_037214	2.1334881361528466
NP_037215	1.0206831073769667
NP_037216	1.4750853904537233
NP_037217	2.8640885329353587
NP_037218	-1
NP_037219	1.180237533157636
NP_037220	1.3825336177202543
NP_037221	-1
NP_037222	1.2157341006395908
NP_037223	0.6286929634677192
NP_037224	0.6630836471620877
NP_037226	-1
NP_037227	1.130245106414632
NP_037228	0.98813098806936
NP_037229	-1
NP_037230	1.3462567108470258
NP_037231	-1

NP_037232	1.0757695102558018
NP_037233	1.116584723154712
NP_037234	0.9830431947810448
NP_037235	-1
NP_037236	4.412991136634996
NP_037238	1.1399275273007603
NP_037239	-1
NP_037240	1.2268052147759965
NP_037241	-1
NP_037242	2.222369989157486
NP_037243	1.1630618376736288
NP_037244	-1
NP_037245	0.9395152343711282
NP_037246	1.1872126400356475
NP_037247	0.2957341356768329
NP_037248	1.1078871069821519
NP_037250	-1
NP_037251	-1
NP_037252	-1
NP_037253	-1
NP_037254	1.0334778329478795
NP_037255	1.0435056628286121
NP_037256	1.0395449233739853
NP_037257	0.7792677380350693
NP_037258	1.0593900562729985
NP_037259	0.8751766813957151
NP_037260	15.312815833649323
NP_037261	0.546301906149037
NP_037262	1.4750585095720457
NP_037263	1.8989805424538126
NP_037264	1.2173846292414894
NP_037265	1.0604700800420523
NP_037266	1.370125488577572
NP_037267	1.4718026059393925
NP_037268	-1
NP_037269	1.4652908867279308
NP_037270	1.8313200013316169
NP_037271	-1
NP_037273	1.543985805295004
NP_037275	0.9163211938974385
NP_037276	-1
NP_037277	5.469033868811965
NP_037279	1.001309187261877
NP_037280	1.0773958092056182
NP_037281	1.1734705337893765
NP_037282	-1
NP_037283	1.025667080633131
NP_037284	-1
NP_037285	0.7873191009220687
NP_037286	0.38107662331743886
NP_037287	1.2175723895454278
NP_037288	0.9637176059300313
NP_037289	1.0336025334121604
NP_037290	-1
NP_037291	1.529631115893964
NP_037292	2.730760418467367
NP_037293	-1
NP_037295	0.8865595624122192
NP_037297	-1
NP_037298	1.4799490046134425
NP_037299	-1
NP_037300	0.8854533494054286

NP_037301	1.136111023191222
NP_037302	-1
NP_037304	-1
NP_037305	0.9490186470144146
NP_037306	1.9541901794185517
NP_037307	0.877449296071377
NP_037308	1.3357154507378939
NP_037309	0.9835628628831445
NP_037310	-1
NP_037311	-1
NP_037312	1.282570189294237
NP_037313	1.0117076171026285
NP_037314	-1
NP_037315	-1
NP_037316	-1
NP_037317	1.486957704811113
NP_037318	-1
NP_037319	1.2175836569004306
NP_037321	-1
NP_037322	1.02545573834688
NP_037323	0.6510993581269708
NP_037324	1.1403511429383313
NP_037326	1.5238090147510914
NP_037327	1.0976830615482418
NP_037328	-1
NP_037329	0.7906204966311744
NP_037330	0.9267250124107632
NP_037331	-1
NP_037332	1.0230185472160436
NP_037346	1.0802782528659076
NP_037347	-1
NP_037348	1.423631526360042
NP_037349	2.279471756495757
NP_037351	1.1602957782654826
NP_037352	0.8499349286007548
NP_037353	0.9348956037562819
NP_037354	0.9210041399413899
NP_037355	0.9903118326639558
NP_037356	0.9849155242751088
NP_037357	-1
NP_037358	0.8876375220599333
NP_038199	1.0080414667510882
NP_038200	-1
NP_058682	1.1274030997621696
NP_058683	0.8042242588231657
NP_058684	0.7634618066103932
NP_058685	-1
NP_058686	1.144247712571926
NP_058687	0.9334418045411268
NP_058688	-1
NP_058689	1.3204732037170495
NP_058690	1.5490549388343766
NP_058691	1.0215199840336944
NP_058692	0.761092293608284
NP_058693	-1
NP_058694	0.502827889978736
NP_058695	0.5578541592558709
NP_058696	2.214571519901533
NP_058697	-1
NP_058698	0.8871776102399443
NP_058699	1.2322388629872827
NP_058701	0.9289061871518576

NP_058702	1.3572979059346022
NP_058703	0.9290698380496893
NP_058704	1.1153641654279849
NP_058705	1.0602566641262379
NP_058706	-1
NP_058707	0.8940919805952368
NP_058708	1.4828561535428626
NP_058709	4.504285909616941
NP_058710	1.3132681409597684
NP_058711	0.48296658781380697
NP_058712	0.7449327327767509
NP_058713	1.2183252610417357
NP_058714	1.0402379709970795
NP_058715	1.3745761455847112
NP_058716	0.8024315731420947
NP_058717	1.20152378250811
NP_058718	0.8759020989163557
NP_058719	-1
NP_058720	1.8377247128341416
NP_058721	1.0872592066929148
NP_058722	1.3974352407799413
NP_058723	-1
NP_058725	-1
NP_058726	1.0335885056475635
NP_058727	0.10651081107064027
NP_058728	0.5998884865729301
NP_058729	0.8699427958918544
NP_058730	1.3181732842751652
NP_058731	1.2313174927255497
NP_058732	-1
NP_058733	1.3208277826876307
NP_058734	1.165816386651444
NP_058735	1.1111390516898885
NP_058736	1.2550478908056537
NP_058737	1.5406698421915805
NP_058738	1.0677170205357147
NP_058739	0.3228501272951441
NP_058740	-1
NP_058742	1.2680742121661666
NP_058743	-1
NP_058744	3.116569774243507
NP_058745	1.2373584015208043
NP_058746	1.2015603891227817
NP_058747	2.302135830138906
NP_058748	0.8063535246021836
NP_058749	0.8456598432568998
NP_058750	1.5559972221229572
NP_058751	2.341192869570476
NP_058752	-1
NP_058753	0.8615818934897964
NP_058754	1.2267645650990873
NP_058755	0.9077010187562943
NP_058756	1.403871231696067
NP_058757	0.3866103587759433
NP_058758	4.001237338259901
NP_058759	1.2348892160779958
NP_058760	0.7718775532667999
NP_058761	-1
NP_058762	0.7195276242153171
NP_058763	1.095570479612707
NP_058764	0.8681288091614011
NP_058765	-1

NP_058766	0.9904099108666209
NP_058767	1.0745000949565884
NP_058768	0.9225227597065117
NP_058769	1.2613694449234938
NP_058770	0.7418250719416178
NP_058771	0.6665221309611151
NP_058772	0.6852453788590035
NP_058773	-1
NP_058774	-1
NP_058775	-1
NP_058776	0.09948638469522308
NP_058777	-1
NP_058778	-1
NP_058779	1.5234452052171568
NP_058780	-1
NP_058781	-1
NP_058782	1.076249020520814
NP_058783	0.7300482308552433
NP_058784	1.0777092252533593
NP_058785	2.0566098135544633
NP_058786	0.9541669185995506
NP_058787	1.2619985708615142
NP_058788	-1
NP_058789	0.9562844157417041
NP_058790	0.9298760926005768
NP_058791	1.2944061130357778
NP_058792	-1
NP_058793	1.594691280569867
NP_058794	-1
NP_058795	1.0034921175847085
NP_058796	1.1395274570267855
NP_058797	1.138699704577846
NP_058798	0.6799644850117083
NP_058799	0.964272208591316
NP_058800	1.228312451384684
NP_058801	1.1808686692432877
NP_058802	0.7999624770068539
NP_058803	0.9398603984497429
NP_058804	1.1610573757652112
NP_058805	1.195179266913342
NP_058806	-1
NP_058807	1.2525175025302246
NP_058808	1.1017422230872358
NP_058809	0.9587229090543592
NP_058811	1.2597968422234531
NP_058812	1.1683545612738728
NP_058813	-1
NP_058815	1.0130380732527013
NP_058816	1.0341169285286813
NP_058817	-1
NP_058818	-1
NP_058819	1.90204951559965
NP_058820	2.9567067607827386
NP_058821	1.0428852251899403
NP_058822	0.6853664035719115
NP_058823	0.7984656496037178
NP_058824	0.6363445981758766
NP_058825	0.8969111481052328
NP_058826	1.0526526201194688
NP_058827	1.025854880505817
NP_058828	0.8417197709149394
NP_058830	0.6670163566538704

NP_058831	-1
NP_058832	1.20622282424054
NP_058833	-1
NP_058834	0.9171373773807296
NP_058835	1.0590058918724314
NP_058836	1.1833331995760386
NP_058837	1.7858449035905426
NP_058838	0.9329354758310058
NP_058839	-1
NP_058840	-1
NP_058841	0.9027837165400047
NP_058844	0.911280655270799
NP_058845	-1
NP_058846	0.9708183260644945
NP_058847	0.9990057913460549
NP_058848	1.1137932685779448
NP_058849	1.1175532518656464
NP_058850	1.7060742188996303
NP_058851	-1
NP_058852	-1
NP_058854	1.1919749341809205
NP_058855	-1
NP_058856	1.1494682639642704
NP_058857	0.872057463424421
NP_058858	-1
NP_058859	-1
NP_058860	-1
NP_058861	0.9326454569907137
NP_058862	1.2611524011493709
NP_058863	1.1811781462596953
NP_058864	-1
NP_058865	0.9365794782791967
NP_058866	0.9868715493120145
NP_058867	1.1201460788769257
NP_058868	0.9762860245246344
NP_058869	0.7126743434928099
NP_058870	1.5563902255624367
NP_058871	1.1808792262743335
NP_058872	1.1193478212700618
NP_058873	1.214200085192625
NP_058874	2.651666249906258
NP_058875	0.9988488109023843
NP_058876	1.8681725055226461
NP_058877	0.9275747196019385
NP_058878	1.265008601301457
NP_058879	-1
NP_058880	-1
NP_058881	-1
NP_058882	-1
NP_058884	0.8285740166491019
NP_058885	1.2900466802310395
NP_058886	1.1402753044334308
NP_058887	1.1592007873136072
NP_058888	0.7930026844008897
NP_058889	1.1694817718734245
NP_058890	1.1381651695006343
NP_058891	-1
NP_058892	1.5734962014096607
NP_058893	2.9272226279119993
NP_058894	0.8540825207669434
NP_058895	1.061162102216635
NP_058896	0.6414625483414521

NP_058897	1.3215183692281662
NP_058898	1.0472449520944689
NP_058900	1.7557736572972908
NP_058901	1.067031635430064
NP_058902	1.0365725547328464
NP_058903	0.64384669354499
NP_058904	0.5411904185200787
NP_058905	1.070659820120501
NP_058906	0.999057070187841
NP_058907	1.070717228414119
NP_058909	1.3357130438847893
NP_058910	0.8588944936515626
NP_058911	-1
NP_058912	1.8898643263916979
NP_058913	0.6952442191717623
NP_058914	-1
NP_058916	0.6271565585822031
NP_058917	-1
NP_058918	1.2991770926112058
NP_058919	1.658864747586889
NP_058920	-1
NP_058921	0.7290989662246862
NP_058922	1.323061900378176
NP_058923	1.0991104985808213
NP_058924	1.0983180854384853
NP_058925	0.5593698306177578
NP_058926	-1
NP_058927	1.032383173139798
NP_058928	0.03785276396028246
NP_058929	-1
NP_058930	0.8406792426723987
NP_058931	1.100415254725308
NP_058932	0.8898007066582453
NP_058933	-1
NP_058934	-1
NP_058935	1.0954323687473475
NP_058936	-1
NP_058937	-1
NP_058938	-1
NP_058939	1.1989725284157076
NP_058940	1.4168186921006385
NP_058941	1.0886413972826585
NP_058942	1.0887543257465413
NP_058943	1.2741619406884634
NP_058944	1.0012153804245394
NP_058945	0.9356103833157778
NP_058946	0.9536372820792076
NP_058947	-1
NP_058948	-1
NP_058949	1.0045320592537177
NP_058950	1.2174002428938617
NP_058951	1.3064164702482517
NP_058952	1.107087731163098
NP_058953	0.9239422851411563
NP_058954	1.3196239274095432
NP_058955	0.4615969002838204
NP_058956	1.5876430117451792
NP_058957	-1
NP_058958	-1
NP_058959	1.5382902448462452
NP_058960	0.850236738060886
NP_058962	-1

NP_058963	0.7578337689127208
NP_058964	0.9796474834197421
NP_058965	1.082956897675038
NP_058966	-1
NP_058967	0.9699351215143251
NP_058968	1.2383804922072235
NP_058970	1.2376266457567289
NP_058971	-1
NP_058972	0.9686767614681772
NP_058973	1.1583528826600122
NP_058974	1.0195354116445494
NP_058975	0.8861395798363656
NP_058976	0.8519536865002026
NP_058977	0.7919409153826903
NP_058978	0.8032837636870745
NP_058979	0.961958738457161
NP_058980	0.7809919188636044
NP_058981	1.1076529239199668
NP_058982	-1
NP_058984	23.119584997176016
NP_058985	1.1750229399188048
NP_058986	1.0711888244470757
NP_058987	1.1117922910019729
NP_058988	-1
NP_058989	1.257396320828874
NP_058990	0.9001252589854571
NP_058991	1.0615293491584656
NP_058992	-1
NP_058993	0.9939699839893672
NP_058994	-1
NP_058995	0.6641851211537672
NP_058996	-1
NP_058997	0.8463838585953556
NP_058999	-1
NP_059000	-1
NP_059001	1.8829608274625542
NP_059002	0.8677690334297766
NP_059003	0.6245902385144867
NP_059004	1.0251966360634726
NP_059005	1.4717397090209265
NP_059006	3.244005908367542
NP_059007	0.8803685376377439
NP_059008	1.3482320252906972
NP_059009	1.0265923449366858
NP_059010	1.1433092398341547
NP_059011	1.1396456908924908
NP_059012	2.192923866503719
NP_059013	-1
NP_059014	-1
NP_059015	0.7235631437237449
NP_059016	1.0928700551175416
NP_059017	0.896348112426268
NP_059018	1.6606370952679723
NP_059019	-1
NP_059021	1.7720545693482457
NP_059022	1.116210157779474
NP_059023	1.1011093790594866
NP_059024	-1
NP_059025	0.9844873552308159
NP_059026	-1
NP_059028	1.0345197537590596
NP_059029	0.8992625528858049

NP_059030	0.6029930198609973
NP_059031	1.4589611727944585
NP_059032	1.1063278911652976
NP_059033	1.1240544339313496
NP_059034	-1
NP_059035	-1
NP_059036	1.329708792519023
NP_059037	-1
NP_059038	-1
NP_059039	0.730956879454462
NP_059040	1.0759543589901743
NP_059041	-1
NP_059042	-1
NP_059043	1.2663207372114258
NP_059044	1.3138493223891796
NP_059046	1.1847142043643268
NP_059047	-1
NP_059048	1.96970087761938
NP_059049	0.8200955800693173
NP_059050	-1
NP_059051	1.0765639986950568
NP_059052	1.6082186920723245
NP_059053	-1
NP_059054	1.0408848678470475
NP_059055	1.4315161889223649
NP_059056	1.0593289092195493
NP_059057	1.2953317731359164
NP_059058	0.7004238737746454
NP_059059	1.263932595282028
NP_059060	0.9552011709412433
NP_059061	1.2809651267320628
NP_061995	-1
NP_061996	0.9648959461058848
NP_061997	0.9506110838866804
NP_061998	-1
NP_061999	-1
NP_062000	-1
NP_062001	1.0117812157866197
NP_062002	1.0655930482852989
NP_062003	-1
NP_062004	0.9019767224845112
NP_062005	1.1148685400053524
NP_062006	1.0567132778896302
NP_062007	0.7264850037312905
NP_062008	-1
NP_062009	-1
NP_062010	1.9041464409614584
NP_062012	-1
NP_062013	1.4595100384716464
NP_062014	1.0965848981994966
NP_062015	1.1737759190699577
NP_062016	1.2403862104034389
NP_062017	1.4443319068362626
NP_062018	-1
NP_062019	1.4296612782323344
NP_062020	1.298717524066112
NP_062021	-1
NP_062022	0.9270384160288881
NP_062023	0.984552065245711
NP_062024	-1
NP_062025	1.223377256721159
NP_062026	0.6064655001672362

NP_062027	1.133590158566102
NP_062028	-1
NP_062029	1.583520541057285
NP_062030	0.9477151695216443
NP_062031	-1
NP_062032	-1
NP_062033	-1
NP_062034	1.0231660481693903
NP_062035	-1
NP_062036	1.19559670784239
NP_062037	-1
NP_062038	2.666401144921458
NP_062039	2.3968171939703558
NP_062040	1.0283355575916089
NP_062041	1.2950669531949675
NP_062042	-1
NP_062043	0.743146112217519
NP_062044	-1
NP_062045	1.5609744982519527
NP_062046	-1
NP_062047	-1
NP_062048	1.2329016061921607
NP_062050	-1
NP_062051	1.8426853219700456
NP_062052	0.7461417216372889
NP_062053	1.4425414087818471
NP_062055	1.179209082442323
NP_062057	0.8913410889433914
NP_062058	1.4172389754640813
NP_062059	1.7964296928239785
NP_062060	0.7869859912569138
NP_062061	-1
NP_062062	1.7282992323559594
NP_062063	0.9919499972376989
NP_062064	1.1350889990191833
NP_062065	0.6960754224106797
NP_062066	-1
NP_062067	1.4109199945531923
NP_062068	0.9725316286185444
NP_062069	0.744468328537556
NP_062071	-1
NP_062072	-1
NP_062074	1.0431702289094569
NP_062075	-1
NP_062076	0.9131731137372817
NP_062077	1.7990371471487148
NP_062078	1.1596757221425167
NP_062080	-1
NP_062081	1.0501390151736707
NP_062083	-1
NP_062084	1.5001000836208622
NP_062085	0.7707900326403095
NP_062086	0.9943154833840796
NP_062087	-1
NP_062089	0.9486298445914008
NP_062091	1.1992458057171718
NP_062092	1.2066278185696062
NP_062093	0.9694536124512532
NP_062094	-1
NP_062095	1.0006131816632016
NP_062096	0.5823302224226071
NP_062097	0.972731788103424

NP_062098	0.7606548485604941
NP_062099	1.1053872086414183
NP_062100	1.3636481504748046
NP_062102	1.2176516991684163
NP_062103	-1
NP_062104	-1
NP_062105	0.7212816543708712
NP_062106	0.7721147791685717
NP_062107	1.128653577162929
NP_062108	-1
NP_062109	-1
NP_062110	0.8477169555323627
NP_062111	1.1577172269134053
NP_062112	-1
NP_062113	-1
NP_062114	-1
NP_062115	1.769538719145599
NP_062116	0.4883882505256269
NP_062117	1.0584016140951278
NP_062119	0.9958256483011395
NP_062120	1.269126363909587
NP_062121	1.0893450239410707
NP_062122	0.7147198906822253
NP_062123	1.164852788076545
NP_062124	0.9952690642021519
NP_062125	0.949075642219341
NP_062126	-1
NP_062128	-1
NP_062129	1.108423203416104
NP_062131	1.1558212244262653
NP_062132	0.9660149793400225
NP_062134	-1
NP_062135	1.046835009941941
NP_062137	1.2278769160876577
NP_062138	-1
NP_062139	0.9485835485924676
NP_062141	1.160617076102736
NP_062142	1.2421761213361298
NP_062143	0.9189323496736937
NP_062144	0.9578987404638036
NP_062145	1.084624377058343
NP_062146	-1
NP_062147	1.8333823688503095
NP_062148	1.1902508842357236
NP_062149	1.0236728397824448
NP_062150	2.376187361543166
NP_062151	-1
NP_062152	2.095862505036701
NP_062153	0.8167475704221457
NP_062154	-1
NP_062155	0.015347900629285717
NP_062156	1.2257030347262772
NP_062158	1.1204185567757916
NP_062159	0.6261966230145685
NP_062161	1.1378939976050655
NP_062162	0.7645356437997745
NP_062163	1.171747196996647
NP_062164	1.263138157411974
NP_062165	1.085191422975123
NP_062166	-1
NP_062167	1.0575811780984152
NP_062168	1.1941881188525487

NP_062169	1.2184683415506072
NP_062170	-1
NP_062171	1.1999186546047544
NP_062172	0.9509049390197017
NP_062174	0.6854672938502998
NP_062175	1.1014805308847262
NP_062176	-1
NP_062177	1.069804242365383
NP_062178	1.0698756856939233
NP_062179	1.018428375709723
NP_062181	-1
NP_062182	-1
NP_062183	-1
NP_062184	1.3168824741927005
NP_062185	1.3418897510850651
NP_062186	-1
NP_062187	0.6716078880464469
NP_062188	-1
NP_062189	1.3027186569529856
NP_062190	-1
NP_062191	1.5050564587083386
NP_062194	-1
NP_062195	0.9403728155584802
NP_062196	0.7753687933012547
NP_062199	1.0940253072694155
NP_062201	0.9815684505105308
NP_062202	1.1957051655977706
NP_062204	0.9730309761572842
NP_062205	0.9432291601376256
NP_062206	-1
NP_062207	-1
NP_062208	0.8104163005326912
NP_062212	1.3426198873507682
NP_062213	0.8782691761269712
NP_062214	1.1217059781707055
NP_062216	1.057429688583567
NP_062217	-1
NP_062218	-1
NP_062219	1.315100862269563
NP_062220	1.0509998769488131
NP_062221	-1
NP_062222	1.0110795528270051
NP_062223	-1
NP_062224	0.9554188846623334
NP_062225	0.7880710075484769
NP_062226	-1
NP_062227	1.018798559479446
NP_062229	0.8911480041890291
NP_062230	2.4720953156681156
NP_062231	0.6843956988832187
NP_062232	1.0742268252817884
NP_062233	1.2096555605619992
NP_062234	-1
NP_062235	0.9379342145597734
NP_062236	1.079943480462135
NP_062237	0.8841151665780015
NP_062238	0.895874335029153
NP_062240	0.945936199767139
NP_062241	0.9118046491713699
NP_062242	1.0855732794113813
NP_062243	0.8020798534769652
NP_062244	1.3448657806122006

NP_062245	1.3212631092823413
NP_062246	1.193760793743878
NP_062247	-1
NP_062248	-1
NP_062249	1.0672225502781032
NP_062250	1.146068237819341
NP_062251	-1
NP_062252	0.6385195048429476
NP_062253	0.9327753310085983
NP_062256	0.8818456449736342
NP_062257	1.1060400514133504
NP_062258	0.6739594580055551
NP_062259	1.109962921804528
NP_062260	1.3132774590345235
NP_062566	1.2786812954011686
NP_062567	1.1093640674026446
NP_062568	1.1132309928345285
NP_062569	-1
NP_062604	-1
NP_063969	1.1096773306339642
NP_063970	1.2020684708048563
NP_063971	1.1196719087395754
NP_063972	1.009751794570721
NP_064456	0.9131149266015611
NP_064457	0.7002977179242968
NP_064458	0.9747654428365602
NP_064459	1.3839853054395261
NP_064460	1.3878996149340246
NP_064461	-1
NP_064462	1.2633071170811598
NP_064463	1.074368870171142
NP_064464	1.1658785348095466
NP_064465	1.325887618687996
NP_064466	1.4465754383325653
NP_064467	0.2461527100805876
NP_064469	1.0710457067860053
NP_064471	0.7945660273575719
NP_064472	1.3366955975845236
NP_064473	1.8965396168381616
NP_064474	1.0024098797404173
NP_064475	-1
NP_064476	1.8299918145082492
NP_064477	1.2124448779648704
NP_064478	1.4708283256613364
NP_064480	-1
NP_064481	1.030254532841736
NP_064482	1.7962722368148405
NP_064483	0.9074925500717744
NP_064484	0.9279280090869906
NP_064485	0.9724854286485209
NP_064486	1.0591533177788794
NP_064487	-1
NP_064488	1.5519895701596897
NP_064489	0.9323478265273614
NP_064491	1.23634140767408
NP_064697	-1
NP_064698	-1
NP_064702	0.8440662686032973
NP_064704	1.2454691743503152
NP_065204	0.9368767239984362
NP_065412	-1
NP_065413	1.0221387858316255

NP_065415	-1
NP_065417	1.2853904991310754
NP_065418	-1
NP_065707	1.2420934980202605
NP_066125	-1
NP_067087	0.9156030592794829
NP_067088	1.143469890345421
NP_067089	0.8964991101666492
NP_067587	0.9966648251984788
NP_067588	0.818423171499049
NP_067589	14.256271132100004
NP_067590	1.013161477134868
NP_067591	3.36822051729742
NP_067592	0.7611986543602186
NP_067593	1.1042671559300101
NP_067594	1.8846245830356478
NP_067596	1.2488260939044653
NP_067597	2.7555404391246707
NP_067598	1.0129730873761793
NP_067599	-1
NP_067600	1.4167988571455399
NP_067601	1.3348157437100754
NP_067603	-1
NP_067604	-1
NP_067605	0.7603230904110335
NP_067606	-1
NP_067607	0.9894677562341196
NP_067608	0.9342308506282351
NP_067609	-1
NP_067685	1.189230220677789
NP_067686	2.3311206851818542
NP_067687	-1
NP_067688	-1
NP_067689	1.1215538695604603
NP_067690	0.8468212330675513
NP_067691	-1
NP_067693	0.9528115522329205
NP_067694	1.0619913322475372
NP_067695	1.0974884203410715
NP_067696	-1
NP_067698	1.1750960951637273
NP_067700	0.9736307160713791
NP_067701	-1
NP_067702	-1
NP_067703	1.0300730838824361
NP_067704	1.2086219295007241
NP_067705	-1
NP_067708	1.1233377612463826
NP_067709	1.718938560484631
NP_067710	-1
NP_067711	1.194775024293602
NP_067712	1.2160683492518891
NP_067713	1.3824922177910386
NP_067714	-1
NP_067716	-1
NP_067718	1.6630233808691635
NP_067719	-1
NP_067720	1.2725169052389778
NP_067721	0.996841066216772
NP_067722	0.9637295055195613
NP_067723	0.572008476846251
NP_067724	1.254106865958247

NP_067725	1.3832179604990187
NP_067726	0.9831507498973496
NP_067727	-1
NP_067728	8.60670276323207
NP_067729	-1
NP_067730	1.1376277131166592
NP_067731	1.539015954437969
NP_067732	-1
NP_067733	-1
NP_067734	-1
NP_067735	1.0985879814713753
NP_068507	-1
NP_068508	1.0205357859067534
NP_068509	1.0079591389322429
NP_068510	-1
NP_068512	1.7248430607267538
NP_068513	-1
NP_068514	1.4303391515821413
NP_068515	1.2749377996644689
NP_068516	1.1148977788315297
NP_068518	0.6993829138377726
NP_068519	1.3011716020693906
NP_068520	1.1669516881078796
NP_068521	-1
NP_068522	0.9912764377178314
NP_068523	1.110746898404818
NP_068525	0.900579664850529
NP_068526	-1
NP_068527	1.0323409919269073
NP_068528	0.7944209316976345
NP_068530	0.9801550855327291
NP_068531	1.0048546541829773
NP_068532	1.0696533459573712
NP_068533	0.8012969698774777
NP_068534	0.8988158585220964
NP_068535	-1
NP_068537	-1
NP_068538	0.8406444865904216
NP_068539	-1
NP_068540	-1
NP_068542	1.1622158558162476
NP_068543	-1
NP_068544	-1
NP_068606	-1
NP_068607	1.9591027524354572
NP_068608	0.9213787119987651
NP_068609	-1
NP_068610	0.9351391108883114
NP_068611	0.7661794231305096
NP_068612	-1
NP_068613	1.1335748731561195
NP_068614	1.124392907781331
NP_068615	1.3775422899194352
NP_068618	1.059312599340079
NP_068619	1.0523133625539653
NP_068620	-1
NP_068621	0.9536383345239715
NP_068622	-1
NP_068623	1.1237973744229308
NP_068624	1.3669991780298494
NP_068625	-1
NP_068626	-1

NP_068627	-1
NP_068628	1.2730310523658073
NP_068629	1.0913348071016298
NP_068630	-1
NP_068631	1.7187032650297693
NP_068632	0.9829437481244749
NP_068635	1.172120092246285
NP_068637	1.1201088440615194
NP_068638	1.4997666377488137
NP_068639	1.215533284746379
NP_068640	1.2911698094475526
NP_068641	0.8641383652134355
NP_068709	1.7563788011107955
NP_068824	1.0548987573965072
NP_068837	0.8077973225986943
NP_071288	-1
NP_071291	1.487287698823659
NP_071511	0.6880859377330765
NP_071513	1.012484883274979
NP_071514	1.184100991315906
NP_071515	0.905799221295072
NP_071516	-1
NP_071517	1.1925724176078385
NP_071518	0.8140381142044163
NP_071519	0.9243191141586192
NP_071520	0.11295580289141159
NP_071521	0.697213059254414
NP_071522	0.9175624597002378
NP_071524	1.7954627105869998
NP_071525	1.1378238202699464
NP_071526	1.1400370594688656
NP_071527	0.9196572067339864
NP_071528	-1
NP_071529	0.9488095821655479
NP_071530	-1
NP_071531	-1
NP_071532	0.9796376662821258
NP_071534	-1
NP_071535	1.012746374347177
NP_071538	-1
NP_071539	-1
NP_071540	0.9453328641091558
NP_071541	1.1517568965829468
NP_071542	-1
NP_071543	2.6972905990096763
NP_071544	1.5301676464923677
NP_071545	-1
NP_071546	1.0167724569424537
NP_071547	1.335330295020089
NP_071549	-1
NP_071550	-1
NP_071551	-1
NP_071552	-1
NP_071553	0.788985357525443
NP_071554	-1
NP_071555	0.7746840950237038
NP_071556	-1
NP_071557	1.407190204446432
NP_071558	1.2225455226734974
NP_071559	1.1133486780635966
NP_071560	1.7682182011361627
NP_071561	-1

NP_071562	0.6742403900572395
NP_071563	-1
NP_071564	1.0935140618723422
NP_071565	0.9558859675637379
NP_071566	-1
NP_071567	1.0896949497349993
NP_071568	0.6931210833590562
NP_071570	-1
NP_071571	0.8807665917104677
NP_071572	0.7059918782121728
NP_071574	-1
NP_071575	-1
NP_071576	-1
NP_071578	1.0826293594513174
NP_071580	1.2803792930862243
NP_071581	1.5125811921900507
NP_071582	1.300164307392365
NP_071583	1.3492240071338768
NP_071584	1.0403383693454737
NP_071585	0.7154207340154617
NP_071586	1.1559829976376932
NP_071587	0.9486706562988881
NP_071588	1.1231201670517126
NP_071589	-1
NP_071590	0.6904189887386183
NP_071591	-1
NP_071592	0.9683367550095879
NP_071593	0.6526418061138713
NP_071594	-1
NP_071595	0.9637797358818401
NP_071596	1.1852478148877177
NP_071597	-1
NP_071598	0.25368937884518483
NP_071600	-1
NP_071601	0.8982257029977974
NP_071602	0.6593120461234901
NP_071604	0.3773641484479702
NP_071605	2.042131519092767
NP_071606	-1
NP_071607	1.0921308873665885
NP_071608	1.2620095318647109
NP_071609	0.9791467555566192
NP_071610	0.9306260269684258
NP_071611	-1
NP_071612	1.308101203221465
NP_071613	1.2740364962817952
NP_071614	1.5984541137629045
NP_071615	0.9774533134421138
NP_071616	-1
NP_071617	1.6851759778852735
NP_071618	-1
NP_071620	-1
NP_071622	1.158431267488904
NP_071623	0.8787690507783309
NP_071624	1.3516029343625577
NP_071625	1.3351106289986134
NP_071626	0.9210003436280795
NP_071627	1.0741729172525158
NP_071628	-1
NP_071629	-1
NP_071630	1.075666493967247
NP_071632	0.8968106796518175

NP_071633	0.5219540243794882
NP_071636	0.16613688470662888
NP_071637	0.9082773661708073
NP_071638	1.0004793178231843
NP_071639	-1
NP_071775	0.8586661636918599
NP_071776	1.4771385131598687
NP_071777	1.2661311502262809
NP_071778	1.3117549062856755
NP_071779	1.0074593330779986
NP_071780	0.9344337977439006
NP_071781	2.4839063287161864
NP_071782	0.9611114709742057
NP_071783	-1
NP_071784	0.8417635786524539
NP_071785	0.8280526547853392
NP_071786	1.25729153642095
NP_071787	2.021070550141917
NP_071788	1.454870536306091
NP_071789	1.01017807277447
NP_071790	1.211982125114465
NP_071791	0.7025044637394403
NP_071792	0.9878573347967193
NP_071793	1.1933294620922588
NP_071794	0.8258225914482007
NP_071795	0.7724566271343972
NP_071796	1.63832042089843
NP_071798	-1
NP_071799	1.0503119076847027
NP_071852	-1
NP_071886	0.7091409589622929
NP_071943	0.8568164288217774
NP_071944	-1
NP_071946	2.239812557961733
NP_071947	0.7311350934928532
NP_071948	0.9628936657360229
NP_071950	1.0138371890259827
NP_071951	1.0558903018226986
NP_071952	-1
NP_071953	1.1313545374966316
NP_071954	1.4388191568802084
NP_071956	1.070813584516099
NP_071957	0.7596576077929718
NP_071958	1.8684611903697357
NP_071959	1.0992257770554106
NP_071960	1.0584897282720795
NP_071961	1.0633885107362853
NP_071963	0.9676130533186948
NP_071964	-1
NP_071966	1.2984827742562304
NP_071967	1.1450587671326893
NP_071968	2.1875395559806754
NP_071969	0.8238991647432542
NP_071970	1.2684676354662632
NP_071971	1.5833955968067779
NP_071973	1.0585411772908586
NP_071974	0.8505927607444895
NP_071975	4.06891338134279
NP_071976	0.9552620323123606
NP_071977	1.1208723624089614
NP_071978	-1
NP_071979	0.48133872546238776

NP_071980	0.776114246794957
NP_071981	0.7192126225175829
NP_071982	1.0035092578739506
NP_071983	1.1402862569702157
NP_071984	1.0913514970221032
NP_071985	0.720838109469744
NP_071986	0.7387328208884679
NP_071987	0.618617261380806
NP_071989	0.8305757257644695
NP_071990	0.8925456575126137
NP_071991	1.054169472543282
NP_071992	1.0195995807684348
NP_071993	0.8969422717896433
NP_072104	-1
NP_072105	-1
NP_072106	1.5934855615185823
NP_072107	1.6734524447376007
NP_072108	1.1323048496392676
NP_072109	-1
NP_072110	0.9669366660336745
NP_072111	1.2053063034038334
NP_072112	1.0273918744562325
NP_072113	0.9444935301253105
NP_072114	1.6202823963099537
NP_072115	0.8507991500206775
NP_072116	0.8281624153351297
NP_072117	0.8776912329231003
NP_072118	0.8401333711558675
NP_072119	1.1192556454305242
NP_072120	1.0179901260393098
NP_072121	1.0347550573195603
NP_072122	1.1558573842772393
NP_072123	0.96525118862083
NP_072124	1.6926578398726164
NP_072125	-1
NP_072126	13.017777928333361
NP_072127	-1
NP_072128	1.074559258339622
NP_072129	0.9046244631815427
NP_072131	-1
NP_072132	1.9585046362649599
NP_072133	0.9111100065128221
NP_072134	-1
NP_072135	0.9930213442490698
NP_072136	-1
NP_072137	1.3344923902678594
NP_072138	1.1892409368295824
NP_072139	0.9293147087370475
NP_072140	1.135570631680597
NP_072141	0.9635391084952576
NP_072142	-1
NP_072143	1.0380882968638685
NP_072144	1.0741321851362633
NP_072145	1.0072687237728655
NP_072147	-1
NP_072148	1.2442454667944487
NP_072149	1.10705681786245
NP_072150	1.0513020862245155
NP_072151	0.5775455263876913
NP_072152	1.1388300972428482
NP_072153	0.2699161793219213
NP_072155	-1

NP_072156	1.6482704806314539
NP_072157	1.34276267097547
NP_072158	0.9824008871396358
NP_072159	1.021394416598315
NP_072160	-1
NP_072161	-1
NP_072169	-1
NP_073156	0.9674204955439598
NP_073157	1.290011209497469
NP_073158	1.398392501814416
NP_073159	0.6932326170782829
NP_073160	0.7664787760808177
NP_073161	1.1277923151413607
NP_073162	-1
NP_073163	0.9456828425759277
NP_073164	1.1568546052749087
NP_073165	1.0469967412102754
NP_073166	-1
NP_073167	1.0857577264467728
NP_073168	-1
NP_073169	1.6113728155388756
NP_073170	0.9788172453522207
NP_073171	-1
NP_073172	1.4265010379700884
NP_073173	-1
NP_073174	0.8070217037701353
NP_073175	1.3463795649472547
NP_073176	0.8736426054462169
NP_073178	-1
NP_073179	1.1936142482033412
NP_073180	0.6560485085810752
NP_073181	0.9664187803146244
NP_073182	1.0676336583082966
NP_073183	1.2705922355107118
NP_073184	2.690215128840928
NP_073185	1.1480851769258378
NP_073186	0.9105539710721441
NP_073187	0.7666671292947922
NP_073188	1.140448903014257
NP_073189	0.8877164153778571
NP_073190	1.076644603410183
NP_073191	1.2375232894382366
NP_073192	1.0809362666023103
NP_073193	1.1687785402396338
NP_073194	1.0308543175574372
NP_073195	-1
NP_073196	1.5712572205168052
NP_073197	0.8874780427520944
NP_073198	1.2692209955991922
NP_073199	-1
NP_073200	1.1349904659917223
NP_073201	-1
NP_073202	1.059556060817629
NP_073204	1.2668176178830985
NP_073205	1.008340917427397
NP_073206	1.052418364302677
NP_073634	0.9338647117073314
NP_073635	-1
NP_073636	1.0035455094476347
NP_073637	1.0770849348845457
NP_074037	-1
NP_074038	0.9991464939352009

NP_074039	1.0596316422365757
NP_074041	-1
NP_074043	-1
NP_074044	1.313818616681968
NP_074045	-1
NP_074046	1.351993288325324
NP_074047	0.9546762538924742
NP_074048	0.9988231554750104
NP_074049	0.7036439294876643
NP_074050	-1
NP_074051	-1
NP_074052	-1
NP_074053	1.0319138560517678
NP_074054	1.1811695756584137
NP_074055	1.0316829139451595
NP_074056	0.5998033164020207
NP_074057	-1
NP_074058	0.9480103552229499
NP_074059	1.0480624101059874
NP_074060	1.2612974374681902
NP_075209	1.1728127358335017
NP_075210	1.1643509508822936
NP_075211	0.8568241707532401
NP_075212	-1
NP_075213	1.1462362812776938
NP_075214	-1
NP_075215	1.2236670081170393
NP_075216	0.4820837210277766
NP_075217	1.2609562856439742
NP_075218	-1
NP_075219	-1
NP_075220	1.0923033493624774
NP_075222	1.1760762991416933
NP_075223	1.1140887276938098
NP_075224	0.9099228492891834
NP_075225	-1
NP_075227	1.0794800693385336
NP_075228	1.2183880794700033
NP_075229	1.245480135331879
NP_075230	-1
NP_075231	1.171486070183548
NP_075232	-1
NP_075233	1.3809149505692628
NP_075234	-1
NP_075235	1.337269652132473
NP_075236	1.220195469062369
NP_075237	1.3167026127856774
NP_075238	1.0579405840790708
NP_075239	1.397762467171899
NP_075240	1.0159661438682397
NP_075241	0.6969058079262477
NP_075242	-1
NP_075243	-1
NP_075244	1.076011620097201
NP_075245	-1
NP_075246	-1
NP_075247	0.9464990600022246
NP_075248	1.595939553357533
NP_075249	0.5736539209487826
NP_075250	1.3362993594198393
NP_075251	1.4042695501739286
NP_075409	-1

NP_075410	2.319329506144786
NP_075411	-1
NP_075412	-1
NP_075413	1.152733170331237
NP_075414	1.0708269988750672
NP_075415	-1
NP_075416	1.066014808288852
NP_075578	1.3957310923709794
NP_075579	1.4607870013069852
NP_075580	1.0748537005697072
NP_075581	0.8959763727001259
NP_075582	2.2582099177149364
NP_075583	-1
NP_075584	-1
NP_075587	1.2872613920282852
NP_075588	-1
NP_075589	1.4055268973194142
NP_075590	0.8993944690503032
NP_075591	0.9309562471023741
NP_075592	0.9123712262485327
NP_076440	1.0695308035827762
NP_076441	0.9526182639139175
NP_076442	-1
NP_076443	1.2699273754495937
NP_076445	1.0019950718467723
NP_076446	1.0012266305113608
NP_076447	-1
NP_076448	-1
NP_076450	1.292846961505518
NP_076451	-1
NP_076452	-1
NP_076453	0.9661195898227332
NP_076454	0.9384498439580864
NP_076455	1.3597287296806473
NP_076456	-1
NP_076457	-1
NP_076458	-1
NP_076459	1.0586687851159717
NP_076460	-1
NP_076461	1.074934596479329
NP_076462	0.7247526819459448
NP_076463	-1
NP_076464	1.2292781465984393
NP_076465	1.352280966668528
NP_076466	1.097478781047602
NP_076467	1.140932672320235
NP_076468	1.3406292329990588
NP_076469	1.395598976688649
NP_076470	-1
NP_076471	1.071502360635546
NP_076472	1.3398385261335148
NP_076473	0.6900154386569085
NP_076477	1.1113747984020492
NP_076478	0.9874625520306743
NP_076479	1.4299357942355106
NP_076480	-1
NP_076481	-1
NP_076482	-1
NP_076483	-1
NP_076484	1.0039042113001422
NP_076485	-1
NP_076486	-1

NP_076487	-1
NP_076488	-1
NP_076489	-1
NP_076490	1.131887360712445
NP_076491	0.8995018268025483
NP_076492	1.2226050794738688
NP_077039	0.9616109128573814
NP_077040	-1
NP_077041	1.574855177829807
NP_077042	1.1243224726874077
NP_077043	0.9098145813067507
NP_077044	0.8557832882096628
NP_077045	0.8912897305095907
NP_077046	-1
NP_077048	2.189427143416688
NP_077049	0.8697239263060313
NP_077050	-1
NP_077051	1.2395012169869504
NP_077052	-1
NP_077053	1.4699008971639005
NP_077054	-1
NP_077055	1.4779161572850505
NP_077056	-1
NP_077057	1.1599913081775444
NP_077058	0.882482030602172
NP_077059	1.862974955829779
NP_077060	5.779654032416249
NP_077061	0.9906526451328705
NP_077062	0.9213305281075522
NP_077063	0.749967461837358
NP_077064	1.3296572291222846
NP_077065	0.7504071775561592
NP_077066	1.5202687066501452
NP_077067	1.7446091556911014
NP_077068	1.0070520716523845
NP_077069	1.2414277204600146
NP_077070	0.6428983155545828
NP_077071	0.9078980812178893
NP_077072	1.3188238809650226
NP_077073	1.5581408193117696
NP_077074	1.1551400812183343
NP_077075	1.098687603172575
NP_077076	1.069279671632746
NP_077077	1.1428650183650761
NP_077322	0.7390156188875939
NP_077325	1.3512341881602143
NP_077328	-1
NP_077329	1.3201555390777584
NP_077330	-1
NP_077331	2.0079320439222084
NP_077332	2.0311004259411654
NP_077334	1.0724884588761492
NP_077335	0.8245363943899013
NP_077336	0.7752452300423247
NP_077337	0.8865419564954032
NP_077338	0.8042196374112797
NP_077339	0.7922044782658298
NP_077340	1.3528616034211982
NP_077341	1.2214436007567298
NP_077342	0.838102620698184
NP_077344	1.4811403170618533
NP_077345	0.7693260728260586

NP_077346	1.1850111707953823
NP_077347	3.993305035089847
NP_077348	-1
NP_077349	0.7952675966856745
NP_077350	1.0211546413043597
NP_077351	-1
NP_077352	-1
NP_077353	1.1159621187051427
NP_077354	-1
NP_077356	-1
NP_077357	1.3637210123908041
NP_077358	-1
NP_077359	0.7831063754663324
NP_077360	1.0079595420771046
NP_077361	0.9523782624940896
NP_077362	0.781201210082767
NP_077363	1.0222955336041317
NP_077364	0.16976536886681778
NP_077366	0.8688459460886934
NP_077367	-1
NP_077368	1.0159460364546928
NP_077370	1.145476732676381
NP_077372	-1
NP_077373	1.1163159283232973
NP_077374	0.888160690295911
NP_077375	0.7295663660061761
NP_077376	0.1874650735709682
NP_077377	1.5598750733343654
NP_077378	0.868227502217711
NP_077379	1.1415890616080129
NP_077380	1.2822174936360533
NP_077381	1.271368822168087
NP_077809	-1
NP_077810	0.341615207400991
NP_077811	-1
NP_077812	0.9783877340919362
NP_077813	0.803428144465718
NP_077814	0.7926092783932148
NP_077815	0.8439244751787481
NP_085073	-1
NP_085074	0.9850995146110204
NP_085075	0.6617362099262631
NP_085914	-1
NP_110453	0.9473468544656861
NP_110454	1.259023360276875
NP_110455	1.7268730910202337
NP_110456	1.4168428656039669
NP_110457	0.8392829129641145
NP_110458	-1
NP_110459	-1
NP_110460	0.645794220955547
NP_110461	0.2005933842487515
NP_110462	1.000273435898752
NP_110463	0.9870906016288531
NP_110465	-1
NP_110466	-1
NP_110467	-1
NP_110468	0.45956285467308416
NP_110471	1.1214740764650686
NP_110472	1.0851804442201047
NP_110473	1.2315294123199632
NP_110474	1.3573157903783308

NP_110475	0.015917882616225305
NP_110476	0.9998366582250279
NP_110477	-1
NP_110478	1.1506629721375516
NP_110479	1.3168434700487477
NP_110480	1.0588908066369642
NP_110481	1.193040048251387
NP_110482	1.3452062865986154
NP_110483	8.266512529824345
NP_110484	1.0323237373398992
NP_110485	2.2543173147668405
NP_110486	-1
NP_110487	0.9284061256451696
NP_110488	0.7142865035236098
NP_110489	1.1890249715372672
NP_110490	1.4676542756581903
NP_110491	-1
NP_110492	0.6112219027027039
NP_110494	1.0680743197195228
NP_110495	1.1110779465501837
NP_110496	1.229231720384767
NP_110497	0.7882980127001051
NP_110498	-1
NP_110499	0.8895681448500895
NP_110500	1.00361594053621
NP_110502	-1
NP_112247	0.9747039682797403
NP_112248	0.6664629994813539
NP_112249	0.9384326728184746
NP_112250	0.9920362974179323
NP_112251	1.0791089935206435
NP_112252	-1
NP_112253	-1
NP_112254	0.68482278207515
NP_112255	-1
NP_112256	1.6162403294526084
NP_112257	1.1816485864151807
NP_112258	0.6340834817607313
NP_112259	1.0944627729691538
NP_112260	-1
NP_112261	-1
NP_112262	0.9680341669259797
NP_112263	-1
NP_112264	0.7452726950551651
NP_112265	-1
NP_112268	0.8850554793328911
NP_112269	1.0691119545086447
NP_112270	0.9976437303662641
NP_112271	1.074366402743421
NP_112272	-1
NP_112273	1.2883537197046329
NP_112274	0.20351067265750541
NP_112275	1.0170027935424615
NP_112276	1.0586865470953806
NP_112277	-1
NP_112278	1.338153902153082
NP_112279	1.1451912775361537
NP_112280	2.022740344729137
NP_112281	-1
NP_112282	1.1429207726846093
NP_112283	1.0375325754585683
NP_112284	39.694844608366196

NP_112285	0.7908601424434796
NP_112286	1.0077545654444604
NP_112288	1.4795970158403229
NP_112289	1.0280847112773228
NP_112290	1.2632007696141665
NP_112291	1.127765854889024
NP_112292	1.1529109211615716
NP_112293	1.165608876187377
NP_112294	0.908616512657383
NP_112295	0.9455866162840061
NP_112296	1.3299217640397405
NP_112297	1.1714953714903273
NP_112298	1.3325857809291317
NP_112299	1.3860641978318258
NP_112300	-1
NP_112301	-1
NP_112302	1.2392517480814342
NP_112303	0.9362912651889702
NP_112304	0.9061199481177625
NP_112305	1.0248117957573386
NP_112306	1.143698368673886
NP_112307	1.2191394135253746
NP_112308	0.9593684863033853
NP_112309	-1
NP_112310	0.9510167899227957
NP_112312	0.9758094909359293
NP_112313	1.043481124070742
NP_112314	1.2441939955900494
NP_112315	1.0501635775712908
NP_112316	0.6118091235649543
NP_112317	6.200930666092654
NP_112318	0.5700800087821062
NP_112319	1.1937952070376983
NP_112320	1.2504455556213891
NP_112321	0.7329116895133577
NP_112323	1.3512315980260432
NP_112324	0.8036417807155875
NP_112325	0.9709949957308108
NP_112326	0.8662153756291321
NP_112327	1.0811672137320847
NP_112328	0.4150506115475073
NP_112331	-1
NP_112332	-1
NP_112335	-1
NP_112336	1.4597390654201954
NP_112337	0.9856307688738165
NP_112339	0.9062113285731772
NP_112340	1.2044922883882274
NP_112341	0.9385606218009853
NP_112343	1.149068606838407
NP_112344	1.0980683117088295
NP_112345	1.0695689166273246
NP_112347	1.0254140242425107
NP_112349	-1
NP_112350	-1
NP_112351	2.030005614704884
NP_112352	1.0391575715929633
NP_112353	-1
NP_112354	0.509706726921444
NP_112355	1.2441923165837236
NP_112356	1.0154490846301907
NP_112357	0.8987424397829984

NP_112358	-1
NP_112359	0.7153242445172764
NP_112360	1.326034255950174
NP_112361	1.138385046755997
NP_112362	1.0995976580222204
NP_112363	1.0181917834692789
NP_112364	1.0075593198200055
NP_112365	1.0974790347882721
NP_112366	0.9627492464003403
NP_112367	0.9181259923090713
NP_112368	1.0268627803934982
NP_112369	1.2424375652297455
NP_112370	1.0618233081427204
NP_112371	1.1458174485148886
NP_112372	1.1397331104729773
NP_112373	1.124950625201721
NP_112374	0.808071057431207
NP_112376	1.2991615193533688
NP_112377	1.1569665459397032
NP_112378	1.3685060720270872
NP_112379	1.071641532718541
NP_112380	15.197056039670494
NP_112381	0.9796320546726197
NP_112382	1.450711332576227
NP_112383	0.990495245732027
NP_112384	1.121457470800084
NP_112385	-1
NP_112386	-1
NP_112387	1.2030306070630952
NP_112388	1.0846462606341096
NP_112389	-1
NP_112391	0.6640006192755146
NP_112392	1.3324269564818854
NP_112393	0.9119122139280273
NP_112394	1.2190380723014405
NP_112395	1.0965434543440014
NP_112396	0.9934485349238671
NP_112397	1.048057921657707
NP_112398	1.2212025967413058
NP_112399	1.1420892791478472
NP_112400	1.174388728085244
NP_112401	1.232671854501472
NP_112402	1.2484453417561514
NP_112403	1.3179498053537662
NP_112404	1.1789173947100628
NP_112405	0.6062341308509148
NP_112406	1.2225289779786281
NP_112407	1.5982498949815487
NP_112408	0.902070631178117
NP_112409	1.2525912641579737
NP_112410	1.655938051725632
NP_112411	0.8588999617535721
NP_112412	-1
NP_112413	0.8171404597846976
NP_112414	0.7354954703170761
NP_112415	0.9498173526361654
NP_112416	0.8073126339937486
NP_112514	1.300557548518612
NP_112515	1.4440497017189324
NP_112516	1.1401483103832524
NP_112517	-1
NP_112518	0.7980179450318875

NP_112520	-1
NP_112521	-1
NP_112605	-1
NP_112606	0.9453582687902382
NP_112607	1.097369406444234
NP_112608	1.1719874031693143
NP_112610	1.015540556530986
NP_112611	0.8538902726507974
NP_112612	1.0179129067441213
NP_112614	1.000650668525555
NP_112615	0.9896417373899417
NP_112616	1.0350626503655196
NP_112617	0.7323414146871542
NP_112618	1.3295575585781885
NP_112619	-1
NP_112620	0.9864869872818596
NP_112621	0.9724379846903166
NP_112622	-1
NP_112624	-1
NP_112625	0.7191152041686145
NP_112626	-1
NP_112627	0.9222525760424832
NP_112628	1.015900323021457
NP_112629	1.0300330375505249
NP_112630	1.2276224694006734
NP_112631	1.629554093379805
NP_112632	0.9193413036170758
NP_112633	-1
NP_112634	2.141018451390157
NP_112635	0.9369955970987125
NP_112636	1.0414467501798714
NP_112637	1.1433749776352797
NP_112638	1.2117313291890115
NP_112639	-1
NP_112640	0.8853870984852008
NP_112641	1.1566271989658947
NP_112642	1.005624008021255
NP_112643	0.9167546298281446
NP_112644	1.0772639685230583
NP_112645	1.5775153423621682
NP_112646	0.9044905116736369
NP_112647	1.1397900914698393
NP_112648	1.2086643537911232
NP_112649	0.823925020137464
NP_112650	1.136398950212415
NP_113690	-1
NP_113691	-1
NP_113692	0.8812529004218402
NP_113695	1.0209343787292948
NP_113697	0.8942736345664636
NP_113698	0.9750941165236475
NP_113699	0.7672584185185968
NP_113700	1.9034938247012405
NP_113701	-1
NP_113702	0.9361961407828544
NP_113703	1.4275001736772985
NP_113705	1.2986344511746977
NP_113706	0.6880048915452228
NP_113708	0.026781469140044773
NP_113709	1.6688041245771406
NP_113710	1.2997300332825965
NP_113711	-1

NP_113715	0.8866261255703963
NP_113716	0.7387727368627223
NP_113718	1.4159660408294465
NP_113719	0.9982130474115442
NP_113721	-1
NP_113722	-1
NP_113723	1.0837911962204236
NP_113724	1.0658441322915593
NP_113725	1.4726168043696142
NP_113726	1.2565469003771044
NP_113727	-1
NP_113729	0.6931058300249439
NP_113730	1.343903670379257
NP_113731	1.0795742454868391
NP_113732	7.0607428650023385
NP_113733	2.064008426292349
NP_113734	-1
NP_113736	0.8767308988626202
NP_113737	1.3404672152487962
NP_113738	-1
NP_113739	0.8390417683108894
NP_113741	0.8133993007761869
NP_113744	0.7203026871461008
NP_113745	-1
NP_113746	4.860703728648509
NP_113747	1.6463925986072376
NP_113748	0.5174617294072409
NP_113749	1.2793254072133111
NP_113750	1.1630068521479753
NP_113751	1.0152846251398293
NP_113752	-1
NP_113753	-1
NP_113756	1.195154685261105
NP_113757	1.0674669896866753
NP_113760	-1
NP_113761	1.0355029753290284
NP_113763	1.24021500150896
NP_113764	0.9853878153923485
NP_113765	-1
NP_113766	1.0227471014696896
NP_113767	0.740884696314864
NP_113769	1.630092424437564
NP_113771	1.1942620097412002
NP_113772	-1
NP_113773	-1
NP_113774	1.2188658174146614
NP_113775	1.2207560525123349
NP_113776	1.2359611976034874
NP_113777	1.046502890361262
NP_113778	0.39820544985782497
NP_113781	0.788233395743872
NP_113782	0.7555751429961867
NP_113783	0.8504028369650501
NP_113784	1.1378550936832161
NP_113785	1.147594199187851
NP_113786	187.20324426285126
NP_113787	0.9760586246505981
NP_113788	1.0701638935219784
NP_113789	1.0968175616120108
NP_113790	-1
NP_113791	1.097184618362965
NP_113792	1.3407704396465545

NP_113793	1.275259940780652
NP_113794	0.754637240855453
NP_113796	-1
NP_113797	1.977312297652107
NP_113798	1.2572920898363757
NP_113800	1.2882993541009966
NP_113801	-1
NP_113802	1.4456901830467737
NP_113803	0.993468918783763
NP_113804	0.9538867261427821
NP_113805	1.1868274362100557
NP_113807	-1
NP_113808	-1
NP_113809	0.8575943091273084
NP_113810	1.9026137650367694
NP_113811	1.3821402357409147
NP_113812	0.5097852470463279
NP_113814	1.4302619464493984
NP_113815	1.15806718812781
NP_113816	0.6583254011860012
NP_113817	0.7844406053327336
NP_113818	-1
NP_113819	1.091762865716681
NP_113820	0.9502886815867253
NP_113821	1.3623139993942688
NP_113822	-1
NP_113823	1.0469149007496608
NP_113827	1.0637616411248627
NP_113828	1.028369419197822
NP_113829	-1
NP_113830	1.4053051905457252
NP_113831	1.0831623733285143
NP_113832	0.9352202140295313
NP_113833	1.0847497815766014
NP_113834	1.323583601325223
NP_113835	0.6238013824171579
NP_113836	4.0430284884685666
NP_113837	-1
NP_113838	0.996644321228472
NP_113839	-1
NP_113840	-1
NP_113841	0.8114676502080149
NP_113842	1.0794671532465276
NP_113843	0.7369753161954598
NP_113844	1.0623437595863188
NP_113845	1.6773958607272226
NP_113846	1.3244978950104984
NP_113847	0.8828671631397856
NP_113848	0.7703386717136911
NP_113850	1.0955622378477983
NP_113851	-1
NP_113852	2.816837233240966
NP_113853	0.9550786978463236
NP_113854	-1
NP_113855	1.1903083835727408
NP_113856	0.8572547401830284
NP_113857	-1
NP_113858	-1
NP_113860	-1
NP_113861	1.1450853902959999
NP_113862	1.0778510138160333
NP_113863	1.5112121839831203

NP_113864	1.3051455399408447
NP_113865	1.1706314768143469
NP_113866	1.222743077164275
NP_113868	-1
NP_113870	0.5993771289689552
NP_113871	1.0921734021896106
NP_113872	1.6406441766331847
NP_113873	0.7410167573928779
NP_113874	0.9387359852873512
NP_113875	0.8537021146749233
NP_113876	1.5675994420426071
NP_113877	-1
NP_113878	1.5736137761684097
NP_113879	-1
NP_113880	1.0876400113453952
NP_113881	-1
NP_113882	1.0988245509940076
NP_113883	1.3090985031154527
NP_113884	1.1318715021146601
NP_113885	1.0351374603283445
NP_113886	1.0613906248799099
NP_113887	1.1726880557198716
NP_113888	1.1162893863980992
NP_113891	0.9635073235867881
NP_113892	1.014798798747134
NP_113893	0.7480901074526356
NP_113894	1.0990059245518025
NP_113895	0.9122103754008961
NP_113896	1.3191243092637663
NP_113897	0.8754485653582843
NP_113898	-1
NP_113899	1.0825565190704176
NP_113900	-1
NP_113901	1.5021066113137866
NP_113902	1.0359333128993704
NP_113903	0.4113527535207819
NP_113904	0.2939592991340459
NP_113906	0.9558418818690998
NP_113907	1.1177216586417893
NP_113908	1.1299176388385634
NP_113909	1.3248846384250765
NP_113910	1.0542575862178527
NP_113911	1.056999292370669
NP_113913	0.7889674627772489
NP_113914	-1
NP_113915	0.9978389349571937
NP_113916	-1
NP_113917	0.9891089290544275
NP_113918	-1
NP_113919	1.222756620054802
NP_113920	-1
NP_113921	-1
NP_113923	0.9873477978501312
NP_113924	1.0933930907037799
NP_113925	-1
NP_113926	0.9918837769066017
NP_113927	-1
NP_113928	1.1049768767252417
NP_113929	0.8115844210678432
NP_113930	1.2353187202685085
NP_113931	1.2508518089202496
NP_113932	-1

NP_113933	1.3790038722200038
NP_113934	1.71290399879329
NP_113935	-1
NP_113937	1.0477894127983947
NP_113938	-1
NP_113939	1.1008958734511325
NP_113940	1.0079975043772862
NP_113941	2.532406461695014
NP_113943	1.7632313442364513
NP_113944	0.989467355038616
NP_113945	1.124856565859668
NP_113946	-1
NP_113947	-1
NP_113948	0.96794225165107
NP_113949	0.49574385282031547
NP_113950	1.1163049710085453
NP_113951	0.8899307518635647
NP_113954	-1
NP_113956	1.220587665421708
NP_113957	0.7673180599044911
NP_113958	1.561826023259998
NP_113959	1.234675220126119
NP_113960	0.9823702632160325
NP_113961	0.874359064558464
NP_113962	1.01712438668312
NP_113963	2.0508617058264487
NP_113964	1.2428703921809323
NP_113965	0.8161493603157287
NP_113966	1.0513463573163297
NP_113967	1.5111630740275652
NP_113968	-1
NP_113969	0.8591085247650532
NP_113970	0.9847487256415807
NP_113971	1.1511919167053872
NP_113972	0.8996063399431561
NP_113973	0.8493049485716054
NP_113974	1.1179553486134912
NP_113975	1.3390552688944837
NP_113976	2.454545815853058
NP_113977	1.3014342696614607
NP_113978	1.0173845379448572
NP_113980	1.0594359482788838
NP_113981	1.4573338286845718
NP_113983	0.9054007214499272
NP_113984	-1
NP_113985	1.7424942904347411
NP_113986	1.0166711559968222
NP_113987	-1
NP_113988	0.8380100022127782
NP_113989	1.167986281089966
NP_113990	5.635057298655865
NP_113991	1.0898800477414896
NP_113994	-1
NP_113995	0.6069687655548555
NP_113996	0.02003155002313344
NP_113997	1.1491163594655986
NP_113998	-1
NP_113999	1.9462312613992243
NP_114000	1.18838091539782
NP_114001	-1
NP_114002	1.1855802152388761
NP_114003	1.3626147213938788

NP_114004	0.9709896712667996
NP_114005	0.6905288127895144
NP_114006	1.4274381084122976
NP_114007	1.4150597758640624
NP_114008	1.3553589598940985
NP_114009	0.1523332565003053
NP_114010	-1
NP_114011	1.2323070511524232
NP_114012	1.2818892233477808
NP_114013	0.9801372740983525
NP_114014	1.0162339200518287
NP_114015	0.6746222168025898
NP_114016	-1
NP_114018	1.0985575239539669
NP_114019	1.435861029765311
NP_114020	1.1011895691511266
NP_114021	0.8536465945337275
NP_114022	0.4469216357779799
NP_114023	-1
NP_114024	1.569261604384448
NP_114026	1.1295437027927318
NP_114027	0.858226252491634
NP_114028	0.9569687732907927
NP_114029	1.1065612567552123
NP_114039	0.8846708641162946
NP_114061	1.321501772645555
NP_114173	0.5024867920456658
NP_114176	1.8451369825857027
NP_114178	1.6930290889444872
NP_114179	0.8839002960892177
NP_114180	1.0180789171398132
NP_114181	1.010773027879629
NP_114182	0.8658494945010536
NP_114183	-1
NP_114184	1.0439920359970094
NP_114185	1.1723738739878795
NP_114186	1.614723432415676
NP_114187	1.129584885969357
NP_114188	-1
NP_114189	0.8651281357488089
NP_114190	-1
NP_114191	1.0444762286113642
NP_114192	1.125143599464212
NP_114193	1.1408013134017507
NP_114444	1.7603371265351209
NP_114445	1.5432577143870232
NP_114446	0.9149031792753077
NP_114447	0.9978613826162722
NP_114449	1.619492292064725
NP_114450	-1
NP_114451	1.0845461065354556
NP_114452	0.9296975917945128
NP_114454	-1
NP_114455	0.9307986427848696
NP_114456	1.5867857853013616
NP_114458	1.7990810207934018
NP_114459	2.455340195473998
NP_114460	1.5666126492980357
NP_114461	1.2514611907510917
NP_114462	-1
NP_114463	1.3084653392381633
NP_114464	1.0472091054369284

NP_114465	-1
NP_114466	-1
NP_114468	1.0222041556222732
NP_114469	0.9639475856511055
NP_114470	-1
NP_114471	1.4689976318005646
NP_114472	0.7183578570907695
NP_114473	-1
NP_114474	0.28830781345885087
NP_115792	1.008869231367531
NP_115851	-1
NP_116001	0.9638036231889218
NP_116002	0.7516186061115344
NP_116003	0.9480640021100748
NP_116004	1.2486442436862732
NP_116005	1.1011156277680718
NP_116006	1.1820930305350388
NP_116785	-1
NP_148981	0.9636050570130922
NP_149086	-1
NP_149087	1.2641146432741992
NP_149088	1.3681750284942025
NP_149089	0.8915095327524752
NP_149090	0.7238741516518911
NP_150233	0.9876740836956486
NP_150236	1.0041564380951418
NP_150237	1.079249995079325
NP_150238	1.021053002789482
NP_150239	0.937180316957734
NP_150240	-1
NP_150641	1.868415128871531
NP_203500	1.172775963278209
NP_203502	0.9596157558004074
NP_203503	1.086826511040905
NP_203523	-1
NP_203694	0.7756649764512402
NP_254276	1.0842517081067804
NP_254277	1.1506347503540324
NP_277020	1.0970376143099896
NP_277034	1.0449265904446967
NP_284925	1.1318926212755238
NP_414541	1.456267778954658
NP_434685	1.0020792392462512
NP_434686	1.1821575010975438
NP_434688	0.8054747975207045
NP_434689	-1
NP_434690	-1
NP_434692	-1
NP_434693	-1
NP_434694	1.5241909504747608
NP_434695	1.0440303007199903
NP_434696	1.1645717068311197
NP_439894	6.623323790918965
NP_443211	1.0092138714191856
NP_443212	-1
NP_443213	0.9988264316508988
NP_443215	1.1745089547434833
NP_444178	-1
NP_444180	0.2918608879401945
NP_445740	-1
NP_445741	-1
NP_445743	1.0513759723655847

NP_445745	-1
NP_445746	-1
NP_445747	1.686071361474359
NP_445748	-1
NP_445749	0.9790022813288367
NP_445751	-1
NP_445753	0.7982527985293464
NP_445754	-1
NP_445755	-1
NP_445758	1.1474153085272305
NP_445759	1.0140618530634649
NP_445761	1.0188280612775769
NP_445762	0.68475974905759
NP_445763	1.2864157229066933
NP_445765	1.1689692968934042
NP_445766	1.8217752167178503
NP_445768	0.9634276781220755
NP_445769	-1
NP_445770	-1
NP_445771	1.2013960656108484
NP_445773	3.579680515147286
NP_445774	0.9501339463912406
NP_445775	1.3007609997084202
NP_445776	1.1807230903397519
NP_445777	-1
NP_445778	1.7745936505470028
NP_445779	-1
NP_445780	1.6096960647477287
NP_445781	-1
NP_445783	0.8536562065145366
NP_445784	0.6936386168016466
NP_445785	-1
NP_445786	1.0116321903448446
NP_445787	1.4286317100382318
NP_445788	-1
NP_445789	0.9830543276864963
NP_445790	-1
NP_445791	0.9465915347329161
NP_445792	1.025578164500632
NP_445793	0.6687006079070849
NP_445794	-1
NP_445795	0.8527609443460351
NP_445796	-1
NP_445797	0.8353890686085231
NP_445798	2.781598211548266
NP_445799	0.9796948290970088
NP_445800	-1
NP_445801	0.9254875457213796
NP_445802	0.7245630327815564
NP_445803	1.9129009893795257
NP_445804	2.6269460670423386
NP_445805	-1
NP_445806	1.1193294119330859
NP_445807	1.7146726194957014
NP_445808	0.8191975498690206
NP_445809	1.100740105946928
NP_445810	0.6442545735048486
NP_445811	0.779773484890533
NP_445812	1.5383637514942343
NP_445814	1.0563863269069873
NP_445816	-1
NP_445817	0.2109741508107057

NP_445821	0.8874233524822808
NP_445822	0.6952883103658255
NP_445823	0.8076191956629896
NP_445824	0.9045250626977879
NP_445825	0.5034859361755427
NP_445826	0.896085723330537
NP_445827	1.0848908941864128
NP_445831	-1
NP_445832	1.0715309924103158
NP_445833	-1
NP_445835	1.0344805266743509
NP_445837	3.0334652611286876
NP_445840	-1
NP_445841	1.0731960908300875
NP_445842	1.4627558675528107
NP_445843	2.585125440111266
NP_445846	2.4834983841006752
NP_445847	-1
NP_445851	-1
NP_445852	1.0408008567664064
NP_445853	0.25618730949287943
NP_445854	-1
NP_445855	1.269478085818673
NP_445856	1.0517693830033172
NP_445857	-1
NP_445858	1.241157905612445
NP_445859	1.2543166634888676
NP_445860	-1
NP_445861	0.5303528305766382
NP_445862	0.9514755621773755
NP_445863	0.956677308755518
NP_445864	1.129325894647019
NP_445867	1.1031436088938102
NP_445868	-1
NP_445869	-1
NP_445870	1.0882371656691552
NP_445872	1.035697116823637
NP_445873	0.6874982718133584
NP_445875	-1
NP_445876	-1
NP_445877	0.8612383424434427
NP_445879	0.755450924511074
NP_445880	1.5040970847287465
NP_445881	-1
NP_445882	1.2204292089231383
NP_445883	-1
NP_445885	0.8609058650726208
NP_445887	1.0047732657407031
NP_445888	1.1685706312325843
NP_445889	0.7610114395912763
NP_445890	1.1708398318298154
NP_445891	1.012592010286918
NP_445892	1.1225124750130795
NP_445893	-1
NP_445897	1.9755820545150222
NP_445900	0.7411504827755788
NP_445905	1.1919637888374788
NP_445907	1.1653466327680764
NP_445908	0.32117905720692136
NP_445909	-1
NP_445910	0.7552425284910234
NP_445911	0.9302030999834313

NP_445912	-1
NP_445914	1.2151039693994152
NP_445915	1.0289932687771832
NP_445916	0.6967403839580094
NP_445917	-1
NP_445919	0.9706816791593299
NP_445921	1.1917613586294482
NP_445923	0.9037694801881124
NP_445924	0.8670521736484811
NP_445925	-1
NP_445926	1.061242235690795
NP_445927	1.1074359744016604
NP_445928	1.0291879250606146
NP_445929	1.093207398069001
NP_445930	-1
NP_445932	1.5659903318623547
NP_445933	1.6502358465584601
NP_445934	2.8580396381280004
NP_445935	1.1503963914614141
NP_445936	1.27925948722335
NP_445937	1.1560805286987457
NP_445938	1.6372294445979865
NP_445939	1.3267967642766625
NP_445942	1.4423636304689154
NP_445943	-1
NP_445944	0.9802744307477249
NP_445945	0.8438464240803785
NP_445946	0.7133021903070059
NP_445947	-1
NP_445948	-1
NP_445949	-1
NP_445950	-1
NP_445951	1.1497126193609228
NP_445952	1.1601169658388932
NP_445954	3.530567650454394
NP_445955	0.7839250159959527
NP_445958	1.5702497155157051
NP_445959	0.5746030892136751
NP_445960	-1
NP_445961	0.9576960923436622
NP_445963	-1
NP_445964	1.048465855528952
NP_445966	-1
NP_445970	1.842085464861226
NP_445972	1.18134840115618
NP_445973	1.244772606613467
NP_445974	0.8052485112204006
NP_445975	0.8348443914611893
NP_445976	1.0913520480636172
NP_445977	0.978315115933793
NP_445978	-1
NP_445979	1.1151181636031484
NP_445980	1.0153503466454763
NP_445981	1.1953579395443885
NP_445982	2.4246620306207887
NP_445983	1.3404537290065148
NP_445984	0.7711034798470916
NP_445986	0.9710690499520425
NP_445987	1.0530202994795348
NP_445988	0.7954078901762484
NP_445989	1.4302561540923662
NP_445990	1.3139926955684103

NP_445991	1.1022920402245644
NP_445992	1.406130740068824
NP_445993	1.0006599192182273
NP_445994	1.078017980043141
NP_445995	1.0775577350674908
NP_445996	2.4011420738576548
NP_446000	0.8858492197999798
NP_446002	1.5950776188426243
NP_446003	1.1086025151646495
NP_446004	1.0226337433408916
NP_446005	0.9486764550144066
NP_446006	1.3029457200102452
NP_446007	2.01040849479329
NP_446008	0.8591286189696856
NP_446009	1.1608130662559526
NP_446010	1.2467686693446587
NP_446011	2.257235073848403
NP_446013	1.1492229740229787
NP_446014	-1
NP_446015	1.399842737585244
NP_446017	1.4407549836676616
NP_446019	1.6958915799493868
NP_446020	1.156289961810794
NP_446021	0.9062969944316496
NP_446023	0.5930750135203106
NP_446024	0.3289930404592872
NP_446025	1.426705111436458
NP_446026	1.0495457480174706
NP_446027	1.0673808687080597
NP_446028	0.9107585624330921
NP_446029	-1
NP_446030	0.943956472147074
NP_446032	1.0420910302711297
NP_446033	0.9996376747679937
NP_446034	0.7999057667831271
NP_446035	-1
NP_446036	1.1470810904726114
NP_446037	0.887106306818444
NP_446038	0.8291965592983801
NP_446039	1.554008834485755
NP_446040	0.850127324372203
NP_446041	1.2454808020735881
NP_446042	1.0381173696391424
NP_446043	0.39020875977294134
NP_446044	0.9369143637785728
NP_446045	0.9958100387483931
NP_446046	3.5176971144475737
NP_446047	1.1860153211082134
NP_446048	0.6579098812932399
NP_446049	1.0467297754659262
NP_446050	0.9468673471777824
NP_446051	1.1703025502609765
NP_446052	1.4161599795779505
NP_446053	1.2972802469000997
NP_446054	0.8299222613368104
NP_446055	-1
NP_446056	0.7334404197112002
NP_446057	1.0035730673636964
NP_446058	0.9619476395341788
NP_446059	0.9865435515803421
NP_446060	1.1939790434373774
NP_446061	1.2998099765305

NP_446062	1.7202042761836938
NP_446063	0.6712772139565625
NP_446064	2.07793742703282
NP_446065	1.1875968646056931
NP_446066	-1
NP_446067	0.683328928131658
NP_446068	0.8547706998784791
NP_446069	-1
NP_446070	1.2215268675212492
NP_446071	1.9449363747631596
NP_446072	1.3266408229132034
NP_446073	0.9956589777429832
NP_446074	1.3895313332141663
NP_446075	1.248507896889525
NP_446076	-1
NP_446077	0.9474346080069569
NP_446078	-1
NP_446081	-1
NP_446082	1.3591813334666245
NP_446083	1.112390236099232
NP_446085	1.6478143275414627
NP_446086	1.579759297179069
NP_446087	1.0824937909294894
NP_446089	1.1868087765633022
NP_446090	0.8784207222502936
NP_446091	0.8579271718980949
NP_446092	-1
NP_446093	1.520028911783298
NP_446094	1.5449709389445185
NP_446095	-1
NP_446096	-1
NP_446097	-1
NP_446098	1.174321737661115
NP_446099	1.313630976507661
NP_446100	-1
NP_446101	1.2129256198419796
NP_446102	1.0491964863611212
NP_446103	-1
NP_446104	-1
NP_446105	0.7870590627226319
NP_446106	0.6670083664059262
NP_446107	1.2412558793904764
NP_446108	-1
NP_446109	1.1797240870136674
NP_446114	1.49260490514955
NP_446116	0.5033231701098416
NP_446117	1.604623547185012
NP_446118	-1
NP_446119	0.3912824410000659
NP_446121	0.8314954082166904
NP_446122	1.0863708683008473
NP_446123	1.4253473959421339
NP_446126	2.170733607860614
NP_446127	1.1259191226659995
NP_446129	0.8678579376452235
NP_446130	-1
NP_446131	-1
NP_446132	-1
NP_446133	1.0606984428590092
NP_446134	0.7970740959102679
NP_446135	1.137960548240747
NP_446136	0.8129448674317147

NP_446137	1.1953137091485144
NP_446138	-1
NP_446139	0.9902139784304946
NP_446140	-1
NP_446141	1.0361832547722565
NP_446142	1.0776632232978403
NP_446150	0.8819683645587869
NP_446151	-1
NP_446152	-1
NP_446153	-1
NP_446155	0.654569751057837
NP_446156	1.8895142763464887
NP_446158	-1
NP_446159	0.9493065390223725
NP_446165	2.1779875960494026
NP_446166	3.9563235058766093
NP_446168	-1
NP_446170	0.8351106178032989
NP_446171	0.3514846715451471
NP_446172	1.0194626883229305
NP_446173	-1
NP_446174	0.9713450027632352
NP_446175	1.108172359330707
NP_446176	0.9751189805331686
NP_446178	-1
NP_446179	0.695383101044332
NP_446181	0.8878112529896616
NP_446182	1.4600670365978985
NP_446183	1.0189689033312375
NP_446185	1.2267751046901714
NP_446186	1.5807957326042663
NP_446187	1.2912636776267792
NP_446188	2.2816061340881117
NP_446190	1.6090480672576437
NP_446191	1.6398706632373319
NP_446192	1.2526153347739017
NP_446193	0.8975896783433095
NP_446194	0.9421176571238116
NP_446195	0.9073800302181146
NP_446196	-1
NP_446198	-1
NP_446199	1.1569281374127562
NP_446200	0.8890715127466192
NP_446201	1.00482744638838
NP_446202	-1
NP_446203	0.8960368033089172
NP_446204	0.8723317673012625
NP_446205	1.1079078250359555
NP_446206	1.227848371430549
NP_446207	1.268678921239928
NP_446208	0.8797472234519187
NP_446210	1.329209833049295
NP_446214	0.8214142751931034
NP_446215	1.159381843968839
NP_446216	1.4095952582948457
NP_446217	0.9441442519253735
NP_446220	0.8168212803821572
NP_446221	1.4932327657064208
NP_446222	2.4127070308767284
NP_446223	-1
NP_446224	0.6112171360230988
NP_446226	1.1434250359864817

NP_446229	0.6067266400071463
NP_446230	1.064823933510332
NP_446231	0.8993609395654711
NP_446233	-1
NP_446235	0.6243198188040301
NP_446237	0.9852884593040424
NP_446238	0.7682152427640832
NP_446239	1.1078276835687084
NP_446240	-1
NP_446243	-1
NP_446244	0.9872198295255371
NP_446246	1.171110339097488
NP_446247	1.048003943051987
NP_446248	1.1482009063476997
NP_446249	1.2221064777240913
NP_446250	1.0449442397884396
NP_446251	0.8502136032874438
NP_446252	1.0890017970858141
NP_446253	1.2852515495920112
NP_446256	-1
NP_446258	-1
NP_446259	0.8664913763919898
NP_446260	-1
NP_446261	-1
NP_446262	0.9839334489055447
NP_446263	0.8246966623406901
NP_446264	0.787607988274471
NP_446266	1.1043090251655647
NP_446268	-1
NP_446269	-1
NP_446270	1.3625091288565343
NP_446271	0.9348933614258631
NP_446272	-1
NP_446273	0.7752431316424521
NP_446274	1.5599273428011902
NP_446276	1.3205629486130883
NP_446277	0.7520591752385942
NP_446278	0.7673538980415443
NP_446279	0.5436825555041559
NP_446280	-1
NP_446282	1.3807770882640515
NP_446283	-1
NP_446284	-1
NP_446286	-1
NP_446287	0.9697503530252143
NP_446288	-1
NP_446289	0.8700811002455057
NP_446290	1.2760193754215414
NP_446292	4.535825196925836
NP_446294	0.9304672140071752
NP_446295	0.8994480447744055
NP_446296	-1
NP_446297	-1
NP_446298	1.0384157600270865
NP_446299	0.7225103750276519
NP_446300	0.9503890959386587
NP_446301	0.4455933030673403
NP_446302	0.7797591596888588
NP_446303	0.9881371209875502
NP_446305	0.8705971314245828
NP_446306	0.7535944425401174
NP_446308	-1

NP_446309	1.11401247221238
NP_446310	1.7151410240254783
NP_446311	-1
NP_446314	0.9668474584222767
NP_446315	-1
NP_446316	1.1950656133282602
NP_446317	0.7724906517541886
NP_446318	0.9769242275252027
NP_446319	1.0527643154141104
NP_446320	1.084480871069625
NP_446321	1.0705108727147121
NP_446322	1.1215979923035764
NP_446326	-1
NP_446328	0.8268438848191817
NP_446329	0.8511416626207245
NP_446330	1.2764136176176355
NP_446331	-1
NP_446332	0.8373163553001927
NP_446333	0.8604033332050066
NP_446334	0.8272926680105203
NP_446335	1.284093958085822
NP_446336	1.042008271808911
NP_446337	1.0583610820542675
NP_446338	0.8390221813414963
NP_446339	1.1915698199077438
NP_446340	1.2487926454487825
NP_446342	1.112967881798767
NP_446343	-1
NP_446344	1.2706496103226645
NP_446345	-1
NP_446346	1.5550525091585063
NP_446347	1.207237030235377
NP_446348	0.9302569036586026
NP_446349	1.0288895028297775
NP_446351	0.9543884770044282
NP_446353	-1
NP_446354	1.1606585439212893
NP_446355	-1
NP_446356	0.8379999584105041
NP_446357	0.9329233777825716
NP_446358	1.5101186168315952
NP_446359	1.2562268526905243
NP_446360	1.4276344208432687
NP_446361	1.2814914193435054
NP_446362	0.9597990693010292
NP_446363	1.2832176767957468
NP_446364	0.7622749987235742
NP_446369	0.9467373966293833
NP_446370	-1
NP_446371	1.0477948381736106
NP_446372	0.8212078110736446
NP_446373	1.3300991974234402
NP_446374	-1
NP_446375	1.3172297122078698
NP_446376	1.2707928360706084
NP_446377	1.0120384101327031
NP_446378	1.6290996142184373
NP_446379	0.2958165623394425
NP_446380	1.0620723123045097
NP_446381	0.5701277503664012
NP_446383	1.0015927898209482
NP_446385	1.346920772093885

NP_446386	0.6602823070815956
NP_446388	1.7778717374371193
NP_446389	1.4463629299160896
NP_446397	-1
NP_446398	1.1223956976005038
NP_446399	-1
NP_446400	1.0646843524477614
NP_446401	2.3580109202492303
NP_446402	0.943654326314892
NP_446403	1.3599268446450843
NP_446404	1.5455268995082927
NP_446405	0.5598647852980461
NP_446406	-1
NP_446407	1.1438022700277084
NP_446409	1.0855696932238719
NP_446410	-1
NP_446411	0.936277629037801
NP_446412	2.2366937850372395
NP_446413	0.5707649166134304
NP_446414	-1
NP_446415	2.204331414542519
NP_446416	-1
NP_446417	0.6605673231869148
NP_446419	1.1658211045178473
NP_446420	-1
NP_446421	1.082135995915265
NP_446422	0.9001068305086702
NP_446423	1.0240782547605196
NP_446424	1.0209648559453826
NP_446425	1.057980789576552
NP_446426	1.0591693258134027
NP_446429	-1
NP_446430	1.2292259479227894
NP_446431	1.1268502633414479
NP_446432	0.9625075351833371
NP_446433	1.2397094964212256
NP_446434	1.162525800827203
NP_446435	-1
NP_446436	-1
NP_446437	0.7752083167944143
NP_446438	1.1538948005619094
NP_446440	-1
NP_446442	1.1016183132949433
NP_446444	1.4847036543871364
NP_446446	-1
NP_446447	0.6995751600337075
NP_446448	1.00711275219632
NP_446449	1.0919458765742092
NP_446450	0.9154577943849552
NP_446451	1.0698969344175449
NP_446452	-1
NP_446453	0.6547786684909866
NP_446454	1.5242162844690206
NP_446455	1.2721353591006992
NP_446456	1.0839037139191519
NP_446457	1.20224699283
NP_446458	1.021881107309636
NP_446459	1.224710874869927
NP_446460	1.2879061257453053
NP_446461	-1
NP_446462	-1
NP_446463	1.1092974410735088

NP_476438	1.1553505379545532
NP_476439	0.7604310325143571
NP_476441	1.2595708641667889
NP_476442	0.8625657428456155
NP_476443	1.0297518672984725
NP_476444	2.318122050038637
NP_476445	0.7732890141671723
NP_476446	0.9986323677330842
NP_476448	0.8225048437558649
NP_476449	0.12979724696863798
NP_476450	0.9838584452139334
NP_476455	1.2280021539828323
NP_476456	1.7784010088824713
NP_476457	-1
NP_476459	-1
NP_476461	0.8331057610874358
NP_476462	-1
NP_476463	0.9752584367839855
NP_476464	0.8584248257176096
NP_476465	1.3638284550918223
NP_476466	0.6525691385316028
NP_476468	-1
NP_476470	1.035831873599643
NP_476471	-1
NP_476472	1.2050760078092821
NP_476473	1.1928479577442221
NP_476474	-1
NP_476476	1.3536610631514623
NP_476477	1.1447128482799642
NP_476478	1.127048886330412
NP_476479	-1
NP_476480	0.9601352048368953
NP_476481	1.1943001851141428
NP_476482	1.0128932921257143
NP_476483	1.1381954500349296
NP_476484	0.816089866862508
NP_476485	0.9967682016574848
NP_476487	-1
NP_476488	0.9784486598239567
NP_476489	1.0188130199041592
NP_476490	-1
NP_476492	1.7564759107511534
NP_476493	1.020453638595125
NP_476496	-1
NP_476497	-1
NP_476532	-1
NP_476533	-1
NP_476534	0.3430942388651647
NP_476535	1.1661285261258154
NP_476536	1.378625096517034
NP_476538	1.3353984885012948
NP_476539	1.5272895181878168
NP_476540	0.8167452358003208
NP_476541	1.1355718495591576
NP_476542	0.7427980251596435
NP_476543	1.0302671438171223
NP_476544	1.834954472355103
NP_476545	0.6610750930378559
NP_476546	1.325899358911742
NP_476547	-1
NP_476548	1.1926806093371662
NP_476549	1.0997955588074173

NP_476552	0.954135417391409
NP_476553	0.8482325767489255
NP_476554	-1
NP_476555	1.0620225583030427
NP_476556	1.4192508475071264
NP_476557	1.2504907635254454
NP_476558	0.8749597847132601
NP_476559	0.9153561226038567
NP_476560	1.1627501229649844
NP_476561	0.7060828498150095
NP_478115	-1
NP_478116	-1
NP_478118	1.6673268552492824
NP_478120	0.9165572606496512
NP_511172	1.1330123887537422
NP_511174	1.1189347267845937
NP_511175	1.0635580151259567
NP_511176	-1
NP_511177	1.3781163676986175
NP_511178	0.843314714283118
NP_536319	2.9401700567801257
NP_536321	0.9308485465171277
NP_536322	-1
NP_536324	1.0923532296461786
NP_536325	1.0938929234090407
NP_536335	0.8095947819886766
NP_536336	1.1190950428284048
NP_536725	1.061596772570665
NP_536726	1.124401126750427
NP_536727	0.35320230485930443
NP_536728	1.3438010076467
NP_536729	0.8192809618100436
NP_536730	1.0283365794460622
NP_542143	-1
NP_542144	1.1448388100601894
NP_542147	0.5917883935074361
NP_542148	0.9537179696962502
NP_542149	0.993535090139582
NP_542150	1.2334329977131597
NP_542151	1.3362474013717547
NP_542152	0.903280865735309
NP_542153	-1
NP_542154	-1
NP_542419	4.097866977336842
NP_542420	2.016224741624753
NP_542421	1.5104647441954533
NP_542422	-1
NP_542423	1.1410745708005037
NP_542424	-1
NP_542425	-1
NP_542426	1.104774449131711
NP_542427	0.8671728355318825
NP_542428	0.9336745504929346
NP_542429	3.2228943362245226
NP_542944	1.444643328152891
NP_542945	1.6427525114705832
NP_542946	-1
NP_542947	1.0892641368375469
NP_542951	1.1329511108700776
NP_542953	-1
NP_542954	1.3385805604730268
NP_542955	-1

NP_542956	0.7575583921017024
NP_542958	0.9370944205430269
NP_542959	1.111794183723162
NP_542960	1.837015103835302
NP_542961	0.8494377137083386
NP_542963	1.216787783494487
NP_542964	0.8972713359803545
NP_542965	1.1305838798759684
NP_543161	1.2926511898886979
NP_543162	1.305218623137524
NP_543163	1.0211154462634895
NP_543164	0.8938509342770444
NP_543166	1.3245485755474424
NP_543167	1.2092395984183208
NP_543168	1.0801901734012012
NP_543169	1.3837801848951325
NP_543170	-1
NP_543171	1.0168170981247864
NP_543172	1.2482799527142692
NP_543173	0.863643018401206
NP_543174	-1
NP_543175	0.9301995467217883
NP_543176	0.9283832724341421
NP_543177	-1
NP_543178	1.083251312479822
NP_543179	-1
NP_543180	1.0976986540370344
NP_543181	1.2290427505671193
NP_543182	0.6923893930046651
NP_543183	1.1089346973397207
NP_543184	-1
NP_543186	1.243466172697382
NP_569083	1.0785468114518801
NP_569084	0.9779912477351287
NP_569085	-1
NP_569086	3.041548865982145
NP_569087	-1
NP_569088	0.36256318971865675
NP_569089	1.0245887437861916
NP_569090	1.0762885437734733
NP_569091	0.7236159585213188
NP_569092	-1
NP_569093	1.0282134256226008
NP_569094	-1
NP_569095	2.208958663585127
NP_569096	0.8553660771830095
NP_569097	1.0144835867518036
NP_569098	1.0378792225300737
NP_569099	0.5536433441930064
NP_569100	1.164068148027892
NP_569103	1.8333222676835415
NP_569104	0.9409336706755252
NP_569105	1.8687520491775624
NP_569106	2.1884725005735635
NP_569107	-1
NP_569108	1.109782140398772
NP_569109	0.9148890391359292
NP_569110	1.0480942404464486
NP_569112	0.7538520053738523
NP_569113	4.516152464708735
NP_569114	0.8876425305193809
NP_569115	-1

NP_569116	1.1014019294078965
NP_569117	0.6630910320189208
NP_570090	1.0900291666039355
NP_570091	-1
NP_570092	1.4689319935735823
NP_570093	1.0866341118280713
NP_570094	0.8264109600512787
NP_570095	-1
NP_570096	1.5203068877955994
NP_570097	0.470328767278795
NP_570098	-1
NP_570100	1.0456942323561373
NP_570102	1.526140248143838
NP_570103	-1
NP_570104	0.594977005656538
NP_570105	0.9070044841685746
NP_570106	-1
NP_570107	-1
NP_570108	-1
NP_570109	-1
NP_570110	0.9262444697348858
NP_570111	1.033492179131376
NP_570112	1.244313003402214
NP_570135	0.9665598075725359
NP_570825	-1
NP_570826	1.168249991813138
NP_570827	-1
NP_570829	-1
NP_570830	2.289362693745594
NP_570831	0.9458357300478268
NP_570832	1.2705443729482204
NP_570833	-1
NP_570834	-1
NP_570835	0.8570284311850355
NP_570836	1.0764808982652598
NP_570837	-1
NP_570838	-1
NP_570839	0.9886059026116971
NP_570842	0.8671903487933355
NP_570964	0.9921966868461028
NP_571977	1.2826289314523582
NP_571979	1.3079692187756093
NP_571980	-1
NP_571981	0.9876090177311109
NP_571982	1.3909636642832532
NP_571983	-1
NP_571984	-1
NP_571985	1.2325798121461633
NP_571986	1.188199736673997
NP_571987	-1
NP_571988	0.9368921116860706
NP_571989	1.2304764429176902
NP_579817	0.914391483398877
NP_579818	-1
NP_579819	0.7891969699520923
NP_579820	-1
NP_579823	-1
NP_579824	0.9778551194953208
NP_579825	1.3185674736969668
NP_579827	1.652399076309593
NP_579828	1.0477120130103572
NP_579829	-1

NP_579830	0.94621981111142
NP_579831	1.0643941382497004
NP_579832	1.087835465800172
NP_579833	1.0158307326751481
NP_579834	1.1383393940118178
NP_579835	1.4286972152901114
NP_579836	-1
NP_579837	1.8605888528659804
NP_579838	0.6579870153184791
NP_579839	1.561785470046946
NP_579840	1.058396394783524
NP_579841	1.034923268317367
NP_579842	-1
NP_579843	1.0373436854405587
NP_579844	0.919273950042502
NP_579845	-1
NP_579846	1.2980653255837695
NP_579847	1.0610216005688522
NP_579848	1.4662290421560433
NP_579849	1.0232857068523182
NP_579850	1.1447728964738568
NP_579851	1.3715602235919973
NP_579852	2.1104388630011006
NP_579854	0.9633805580836402
NP_579855	-1
NP_579856	-1
NP_579857	1.355576034311402
NP_579858	1.3175316443082208
NP_596871	0.9655454170160561
NP_596872	1.0854853739755248
NP_596874	1.0725031820580593
NP_596876	-1
NP_596877	1.3845234004114477
NP_596878	0.4256083605210528
NP_596883	1.8139585414815935
NP_596884	1.741484052973893
NP_596885	1.21204693987494
NP_596886	0.8232019791976172
NP_596887	1.4164572148195484
NP_596888	1.0626772554482347
NP_596889	1.2014467112141678
NP_596891	0.9074176908098356
NP_596892	2.042000742336932
NP_596893	1.1043548636200966
NP_596894	1.1041209686286269
NP_596895	1.0865515139677766
NP_596896	-1
NP_596897	1.0367798762141807
NP_596900	1.0794369049524122
NP_596901	1.182360638661975
NP_596903	0.8052106144251212
NP_596904	1.279820810888698
NP_596905	1.3253243626147753
NP_596906	1.090383989067637
NP_596907	2.0836862474128597
NP_596908	1.153304748580415
NP_596909	1.0372888202851667
NP_596910	1.2227324968948006
NP_596911	-1
NP_596912	1.0446378740966449
NP_596913	1.0124909014983865
NP_596914	1.2784869408698163

NP_596915	-1
NP_596916	1.477610891916706
NP_596917	1.0919616442704256
NP_596918	1.0686790470864282
NP_596919	1.4539694545122614
NP_596920	1.1677976901382756
NP_597684	0.5650639054330394
NP_598195	-1
NP_598196	-1
NP_598197	-1
NP_598198	19.321381759363593
NP_598199	-1
NP_598200	-1
NP_598201	-1
NP_598202	1.2775382038885
NP_598203	-1
NP_598205	1.07348223593909
NP_598206	1.1552441187372011
NP_598207	1.8818773727785592
NP_598208	0.6138249158681234
NP_598209	0.7731167023486986
NP_598210	0.9671133141270927
NP_598211	0.8905116531777207
NP_598212	1.2188221491277142
NP_598213	1.2021014014387772
NP_598215	1.008602631035644
NP_598216	-1
NP_598217	-1
NP_598218	1.305275999716683
NP_598219	1.3054208302302284
NP_598220	-1
NP_598221	-1
NP_598222	1.017351101349165
NP_598223	0.9661250489920624
NP_598224	1.2747298228985573
NP_598225	1.2584609948189822
NP_598226	1.5608286530243054
NP_598228	0.9293355529778009
NP_598229	1.2060514951075982
NP_598230	1.2472652627151373
NP_598231	-1
NP_598232	2.142027997900195
NP_598233	0.9581626697262352
NP_598234	-1
NP_598235	0.3602778696279821
NP_598236	0.879549929132029
NP_598237	1.4390473515431221
NP_598238	1.1067063288691372
NP_598239	1.6086438525170645
NP_598240	0.8318497573541513
NP_598241	0.9358619629758036
NP_598242	0.9224671167489573
NP_598243	0.9722858685778525
NP_598244	0.9539531904700729
NP_598246	1.1106504255102514
NP_598247	0.7635601594285844
NP_598250	-1
NP_598251	-1
NP_598252	-1
NP_598253	1.4515128588584176
NP_598254	-1
NP_598255	1.1128608924498837

NP_598256	1.5601249599601121
NP_598257	-1
NP_598258	1.2352415313310867
NP_598259	0.542427132592856
NP_598261	0.7760103985224861
NP_598262	2.72332061144561
NP_598263	0.7788573489184526
NP_598264	1.1425230625749008
NP_598265	-1
NP_598266	1.0479027962215592
NP_598267	1.1769689264804326
NP_598268	0.9609366916525754
NP_598269	1.0352097725337552
NP_598270	0.5802130305309463
NP_598271	-1
NP_598273	0.9429836072072063
NP_598274	0.8610974506129047
NP_598275	1.1283540628312598
NP_598277	0.8584133407855443
NP_598279	1.1192315912790936
NP_598280	0.8638287093943418
NP_598281	-1
NP_598282	1.0254718679539614
NP_598283	20.96799299448381
NP_598284	0.8540078822942468
NP_598285	0.826534313801001
NP_598286	1.1744520016832323
NP_598287	-1
NP_598289	1.0767948055217633
NP_598290	-1
NP_598291	1.3790912139068732
NP_598292	1.1761739736599244
NP_598293	1.2231906231100054
NP_598294	0.633215972507405
NP_598295	1.15470243528595
NP_598298	-1
NP_598299	1.5470542466932038
NP_598300	1.1073373775614548
NP_598301	1.2175441237316431
NP_598302	1.4457974881866285
NP_598303	-1
NP_598304	1.0678244164011599
NP_598305	1.7089025245731364
NP_598306	-1
NP_598307	-1
NP_598308	1.5197619453335445
NP_598413	1.1018341659043083
NP_599153	1.1359925065817724
NP_599155	1.0049362145712903
NP_599156	2.118669552083952
NP_599157	1.4003274849140137
NP_599158	-1
NP_599159	1.036457733451534
NP_599161	1.3505960899485512
NP_599162	1.1316395001370803
NP_599163	1.1449440499593324
NP_599167	0.8150263517134517
NP_599173	1.2138905120792474
NP_599176	1.1419421277076618
NP_599177	0.9760873091052584
NP_599178	0.9814420767266405
NP_599179	2.207127976195466

NP_599180	1.2124126655107732
NP_599188	-1
NP_599190	1.3554467965949009
NP_599191	0.9906212641019132
NP_599192	0.9495169730131755
NP_599193	1.1154095804659658
NP_599196	-1
NP_599197	-1
NP_599198	0.9828434006087856
NP_599199	-1
NP_599200	1.2435536256266095
NP_599201	1.4112206183028175
NP_599203	1.4031882954980766
NP_599204	-1
NP_599205	0.5569719599279078
NP_599206	1.105055139782187
NP_599207	1.2592814656485378
NP_599209	1.1014748275233515
NP_599210	0.5987522054507934
NP_599211	-1
NP_599212	0.9688409173001294
NP_599213	1.1691512397216621
NP_599214	-1
NP_599215	-1
NP_599216	-1
NP_599217	1.679054525068467
NP_599218	0.9860219032911952
NP_599219	0.9363404012389883
NP_599220	0.2802265763767142
NP_599221	1.2223225714881913
NP_599222	1.342283667913187
NP_599223	-1
NP_599225	1.2500511113643162
NP_599227	-1
NP_599228	-1
NP_599229	0.670132660552596
NP_599230	0.7700815467547765
NP_599231	-1
NP_599232	1.1607760792546769
NP_599233	1.2297194798985904
NP_599234	0.6654846565874982
NP_599235	2.9096196475349823
NP_599236	-1
NP_599237	0.5387092510782813
NP_599238	1.0947716536496424
NP_599239	1.3910736274377307
NP_599240	1.2175323412216117
NP_599241	1.2312228496574855
NP_599242	0.978093472520995
NP_599243	1.1854537726125711
NP_599244	2.0458873893401273
NP_599245	1.3680254952642987
NP_599246	1.0313586299407216
NP_602308	-1
NP_604444	1.2942489977005207
NP_604447	0.894902722545134
NP_604448	1.2157463998742981
NP_604450	0.6098911791899843
NP_604451	1.2339022852484833
NP_604452	1.3662359990137811
NP_604453	1.1733095663153577
NP_604454	-1

NP_604456	2.8839203687454797
NP_604457	-1
NP_604460	0.9202965725515596
NP_604461	0.7703919249964213
NP_604462	1.2253863442511237
NP_604463	0.7150133377182158
NP_608302	0.96041504148383
NP_612511	-1
NP_612513	1.6654060009022118
NP_612514	-1
NP_612515	-1
NP_612516	3.500896912069269
NP_612517	0.8390531284654416
NP_612518	1.3850927588147064
NP_612519	0.8777189511432382
NP_612520	1.1107220892178242
NP_612521	-1
NP_612522	-1
NP_612523	-1
NP_612524	0.7563213982254756
NP_612526	3.996148559334898
NP_612527	0.9686845900762341
NP_612528	1.0862210979362161
NP_612529	1.1834986253377568
NP_612531	1.6980673069126708
NP_612532	1.0104550387245739
NP_612533	0.6101221937893849
NP_612534	1.0563894212444802
NP_612536	-1
NP_612537	1.0878475420370604
NP_612539	-1
NP_612540	1.0079730947489058
NP_612541	0.8646969257234317
NP_612542	1.2977084960542316
NP_612544	1.0377864188815606
NP_612545	1.0489495856886615
NP_612546	1.139981073624347
NP_612547	0.949166005789636
NP_612548	1.1648636917699224
NP_612550	-1
NP_612551	-1
NP_612552	-1
NP_612553	0.947528129161157
NP_612556	0.2928958038015068
NP_612558	1.378798691838807
NP_619516	-1
NP_619715	1.211331900182692
NP_619724	1.188694127985367
NP_620178	0.7330177920003067
NP_620181	0.6220457780575952
NP_620182	1.6826379518964834
NP_620183	0.9166903883393979
NP_620184	0.7835045306791838
NP_620186	-1
NP_620187	0.7972701295900618
NP_620188	1.1881940183786717
NP_620190	-1
NP_620191	-1
NP_620193	1.141663609479083
NP_620194	1.3138410011774773
NP_620195	1.065462025432827
NP_620196	-1

NP_620197	0.9914435010341894
NP_620198	0.6553713614202644
NP_620199	-1
NP_620202	0.8810784374928775
NP_620203	0.930496209444287
NP_620204	3.796700142521132
NP_620205	0.9757028808842674
NP_620206	-1
NP_620207	-1
NP_620209	-1
NP_620210	1.0025543778203518
NP_620211	1.522132071560927
NP_620212	1.2336751579555754
NP_620213	2.3854196079194563
NP_620214	1.0903826326474992
NP_620216	-1
NP_620217	2.179990369084634
NP_620218	-1
NP_620219	1.1743498938786905
NP_620220	0.8865164573210085
NP_620221	0.9858967532037317
NP_620222	1.2171600899542987
NP_620225	1.0851703659405003
NP_620226	1.0062133093856678
NP_620227	-1
NP_620228	0.9722696468283932
NP_620229	1.202801754154393
NP_620230	-1
NP_620231	1.08558718390306
NP_620232	1.2627510714482943
NP_620233	0.8566661840704456
NP_620234	1.2300306298452337
NP_620235	-1
NP_620236	1.3576683901099196
NP_620237	1.4856981228102304
NP_620238	0.763087115017436
NP_620239	-1
NP_620240	1.0128395508315116
NP_620241	0.9613047076132767
NP_620242	1.1805709651178207
NP_620243	0.7096567142810213
NP_620244	-1
NP_620245	-1
NP_620246	1.206871301132124
NP_620247	1.1508502796720244
NP_620248	-1
NP_620249	2.9531416199681844
NP_620250	1.116720709499055
NP_620251	1.044798627269554
NP_620252	1.3291498584052663
NP_620253	1.1181302597120164
NP_620254	1.0110148978348235
NP_620255	0.6930181546365359
NP_620256	0.9209360281682594
NP_620257	-1
NP_620259	0.97997981095947
NP_620260	0.4700807522371624
NP_620261	-1
NP_620262	1.4718100432732717
NP_620263	1.3338034472994122
NP_620264	1.4264119528851664
NP_620265	0.8464831873102397

NP_620266	1.127223721800751
NP_620267	-1
NP_620268	1.0115567780871333
NP_620269	1.1071678349173346
NP_620270	-1
NP_620271	2.2498292336027337
NP_620272	0.9501345651939888
NP_620273	0.7704165188006374
NP_620274	0.9036103524744958
NP_620275	1.3871539237532677
NP_620276	-1
NP_620277	-1
NP_620431	1.408633668974305
NP_620432	0.9895218488168833
NP_620433	-1
NP_620434	-1
NP_620605	-1
NP_620606	0.43296955775709695
NP_620607	1.4937385060711827
NP_620608	1.1841491387306706
NP_620609	1.412380591221842
NP_620610	1.2132142010943638
NP_620611	1.2583949659026674
NP_620612	-1
NP_620691	1.1272812473170424
NP_620780	1.0309132304916169
NP_620781	1.0869432940633048
NP_620782	-1
NP_620784	0.9298385492088032
NP_620785	0.9737313101894259
NP_620786	1.0090427623687923
NP_620787	-1
NP_620788	0.7393594760073418
NP_620789	1.9987415716508972
NP_620790	1.0591058304318623
NP_620791	1.106246254206034
NP_620793	1.116375039874181
NP_620794	1.4915720960869665
NP_620795	1.273861470116579
NP_620796	0.8146806591383974
NP_620797	1.1960815894865107
NP_620798	1.0733062961106532
NP_620799	0.9436591228969745
NP_620800	0.9995600548527755
NP_620801	0.9327308358114227
NP_620802	-1
NP_620803	0.8970810227425362
NP_620804	0.9280745838590598
NP_620805	1.333928357421273
NP_620806	0.8057506971386609
NP_620807	0.9938003413921573
NP_620808	1.0764103620987007
NP_620809	1.149862827946345
NP_620810	1.206039472589351
NP_620811	0.8444399870380728
NP_620812	-1
NP_620813	0.711344888133535
NP_620814	1.058717316373729
NP_620815	1.0153771243454401
NP_620816	1.0387484889266052
NP_631922	-1
NP_631923	1.8375407036129783

NP_631924	1.0614756948253263
NP_631925	0.8531700987187075
NP_631926	-1
NP_631928	1.0408205353236144
NP_631929	1.1187022164091494
NP_631931	0.9403261926443665
NP_631932	-1
NP_631933	-1
NP_631962	0.24923733765560982
NP_631963	-1
NP_631976	0.7547163400102171
NP_631977	-1
NP_640347	1.1726432594638168
NP_640348	1.1355383623901338
NP_640349	1.2870393819852188
NP_640350	-1
NP_640351	-1
NP_640352	0.594785169237899
NP_640353	0.9466476250707192
NP_640354	0.9917380244665729
NP_640355	-1
NP_640356	-1
NP_647540	0.8720452061352167
NP_647541	1.0261376338468895
NP_647542	-1
NP_647545	0.961463615332162
NP_647546	1.656743471854986
NP_647547	2.089279469653828
NP_647548	1.267167544284345
NP_647549	1.0442490113028016
NP_647550	-1
NP_647551	0.8654795973588822
NP_647552	0.8991754312769217
NP_647553	-1
NP_647557	1.696362304462797
NP_647558	0.8746783579501413
NP_653331	1.0903028481651806
NP_653341	1.3113273470555626
NP_653343	1.085127692481515
NP_653346	1.1350558128587271
NP_653347	1.162593937306031
NP_653349	1.1020156985995215
NP_653350	-1
NP_653351	-1
NP_653352	-1
NP_653353	-1
NP_653354	1.1570877219196911
NP_653356	1.9282510151069048
NP_653357	-1
NP_653358	1.8372358223848408
NP_653359	1.1204485437337741
NP_653360	1.10259582050045
NP_658907	-1
NP_659549	1.5251080258056968
NP_659550	0.9245479938051581
NP_659551	2.080161725273369
NP_659552	0.8425549147011925
NP_659553	0.9018306330018602
NP_659555	-1
NP_659556	-1
NP_659557	0.9706964989146896
NP_659558	0.8575006074297786

NP_659559	0.9630918585957682
NP_659560	0.8426690894373099
NP_659561	-1
NP_659562	1.1348557060951852
NP_659563	-1
NP_659564	1.411665339224669
NP_659565	1.135358752847021
NP_659566	0.22048558934425888
NP_660185	1.029214093694168
NP_663703	1.09247075517993
NP_663711	1.1397757724967488
NP_663715	-1
NP_663716	1.1493385315598175
NP_663769	1.3441404862719253
NP_663773	-1
NP_663775	1.4651567164958268
NP_663776	1.1060636514344142
NP_665708	1.5207181391256401
NP_665709	1.0155165383736986
NP_665710	-1
NP_665711	-1
NP_665713	0.9747685492707762
NP_665714	-1
NP_665715	1.111480680181814
NP_665716	1.6322945307841863
NP_665717	1.1510314441904497
NP_665718	1.765427297922774
NP_665719	-1
NP_665720	-1
NP_665721	1.1607353670450589
NP_665722	-1
NP_665723	1.843134920950403
NP_665724	0.7398180980953827
NP_665725	-1
NP_665726	1.0629122250715732
NP_665728	1.7384067459212267
NP_665729	-1
NP_665730	0.9215548314256647
NP_665731	1.2106191549520475
NP_665732	0.8408112328555356
NP_665733	0.9842036619182425
NP_665884	0.9401875712404356
NP_665885	2.9980691545620775
NP_665886	1.9044154579112047
NP_665887	1.0120205657171246
NP_671476	-1
NP_671477	1.0977187917686186
NP_671478	-1
NP_671479	0.8687749633231027
NP_671481	0.8204220524721958
NP_671482	1.6540151933784035
NP_671483	1.0795515246886624
NP_671484	-1
NP_671486	3.314968938636138
NP_671487	-1
NP_671694	-1
NP_671706	0.8967680753788138
NP_671738	1.446672126925334
NP_671739	1.2095432410998284
NP_671742	0.8599132690175101
NP_671743	2.1142834724344004
NP_671744	1.0844309712176337

NP_671747	-1
NP_671748	1.0110733829365846
NP_671749	0.9850147830355261
NP_683687	1.2109412643046087
NP_683689	1.0532648523734343
NP_683690	0.8335488371446438
NP_690003	1.6053120977198205
NP_690600	0.9202680689329128
NP_690918	1.0490757965732374
NP_690919	0.9834199896841126
NP_695206	-1
NP_695207	1.1215011740541874
NP_695208	2.0085535920597253
NP_695209	0.9908002744025849
NP_695210	-1
NP_695214	0.857381390788138
NP_695215	0.8169483941508631
NP_695216	0.9938155249171894
NP_695218	0.7967890763684166
NP_695219	1.315380199683566
NP_695220	0.8900072560257632
NP_695222	-1
NP_695223	-1
NP_695224	-1
NP_695225	-1
NP_695226	-1
NP_695227	0.5905570388028358
NP_695228	2.012011465813126
NP_695229	0.8529664057441896
NP_695230	0.8517926686244851
NP_703195	1.1568903336745195
NP_703196	1.2734292344601754
NP_703199	0.9409439130355671
NP_703200	1.2255459209479695
NP_703202	0.8729460541838523
NP_703203	1.036222023379296
NP_705888	-1
NP_705891	0.9976582494237948
NP_705892	0.9907123521505232
NP_705893	0.887362184103722
NP_705894	1.0030994863221174
NP_714942	1.4212023562186125
NP_714943	-1
NP_714944	0.5892699478166444
NP_714947	1.2586213172344263
NP_714951	-1
NP_714953	0.9551610719037088
NP_714954	1.3001237751528056
NP_714956	-1
NP_714957	-1
NP_714958	-1
NP_722543	0.801750110200059
NP_733836	0.7538511664483165
NP_740769	1.171811338554358
NP_740770	1.05330344747198
NP_741984	0.8306142460040723
NP_741985	1.1969705578444443
NP_741987	0.8370899881328082
NP_741988	1.0295092925604765
NP_741989	3.0320456510282963
NP_741990	1.065882482793581
NP_741991	1.0477579822377467

NP_741992	1.2249343592901512
NP_741993	1.107408646716062
NP_742005	1.0609942972372373
NP_742015	0.7488309017595651
NP_742018	0.7062675101188733
NP_742019	0.9090576040106956
NP_742026	0.8122951272290521
NP_742027	-1
NP_742030	1.0900258096446542
NP_742031	0.9654636602093335
NP_742032	0.7316658946520965
NP_742033	0.901594092834716
NP_742035	0.5009776388475468
NP_742036	0.6692969461489556
NP_742037	0.8518086747572617
NP_742039	0.8299079394289732
NP_742041	0.6951012708197947
NP_742042	1.0567276923296436
NP_742045	1.2976623515734909
NP_742052	0.597871071592638
NP_742059	0.8541554510545402
NP_742060	0.9780331195633559
NP_742062	-1
NP_742063	1.0420786624115304
NP_742064	1.1675398328496982
NP_742065	0.9381185337309456
NP_742069	0.9354562895274422
NP_742070	-1
NP_742071	0.8553698965166107
NP_742072	0.9441878187439913
NP_742074	-1
NP_742087	1.6593290707196298
NP_742089	-1
NP_742090	0.9275675728821583
NP_750843	1.235131218666171
NP_750845	1.0009422235203382
NP_757377	1.1160767830551528
NP_757378	0.7579403275104216
NP_758443	0.7493297031398692
NP_758528	-1
NP_758823	-1
NP_758824	-1
NP_758825	0.9213201468477669
NP_758826	0.6994243720272655
NP_758829	0.7554752575701532
NP_758830	1.1436320983625587
NP_758832	1.208179798261438
NP_758835	1.385860741172097
NP_758837	1.1407416128674404
NP_758838	1.8647403207116693
NP_758839	0.7105621099413814
NP_775117	-1
NP_775118	-1
NP_775119	1.3859283177922501
NP_775120	1.2710110995330766
NP_775121	0.8772295448132456
NP_775122	1.0819659482313584
NP_775123	1.5801279892730793
NP_775124	1.191124739627305
NP_775125	1.3009246607202152
NP_775126	0.8834490738991346
NP_775128	-1

NP_775134	1.1334972269014538
NP_775135	1.3763116996823488
NP_775136	-1
NP_775137	0.7604130846436143
NP_775140	0.8853950439290855
NP_775141	1.3916994356743138
NP_775143	0.9496093213167445
NP_775146	1.4365500760992134
NP_775147	0.788722747088567
NP_775148	1.2979093201230782
NP_775149	1.3975269396035686
NP_775150	-1
NP_775151	-1
NP_775152	-1
NP_775153	-1
NP_775155	-1
NP_775156	0.9181029827736169
NP_775158	-1
NP_775159	3.010777074355128
NP_775160	1.594146608666272
NP_775161	0.9737468912564466
NP_775162	-1
NP_775163	-1
NP_775165	0.961700456506382
NP_775166	1.0092119096954693
NP_775167	-1
NP_775168	1.0241722729694756
NP_775169	1.1004649409060847
NP_775170	1.0975634807339805
NP_775171	-1
NP_775174	-1
NP_775175	-1
NP_775176	0.9753966382929057
NP_775411	-1
NP_775412	-1
NP_775413	-1
NP_775414	-1
NP_775415	-1
NP_775416	-1
NP_775417	-1
NP_775418	-1
NP_775419	-1
NP_775420	-1
NP_775421	-1
NP_775422	1.4599275748724463
NP_775423	-1
NP_775425	1.1665913809249207
NP_775426	-1
NP_775427	-1
NP_775435	-1
NP_775436	0.7141886224404755
NP_775437	-1
NP_775438	0.8472515445625609
NP_775439	-1
NP_775440	-1
NP_775441	-1
NP_775442	1.0822005066206135
NP_775443	-1
NP_775444	0.9407692681634555
NP_775445	1.034706754818141
NP_775446	1.1003916170173307
NP_775447	1.287711038325256

NP_775448	-1
NP_775449	1.1963537394414454
NP_775450	0.5483191529787437
NP_775451	-1
NP_775452	1.1243234537312345
NP_775453	-1
NP_775455	-1
NP_775456	1.118292232297406
NP_775457	-1
NP_775458	-1
NP_775459	-1
NP_775460	1.1252227819815324
NP_775461	-1
NP_776209	1.3181633676137634
NP_776210	0.9534563660673285
NP_777353	0.6856865135202573
NP_783167	0.6695967929879627
NP_783168	-1
NP_783169	10.891197250589293
NP_783170	0.8109346964092915
NP_783171	1.2324753066213512
NP_783172	6.225793321496957
NP_783180	-1
NP_783181	1.2454613430575052
NP_783186	1.0605774645891384
NP_783191	0.7827992063021987
NP_783193	0.9700583564341286
NP_783638	0.7697583016729987
NP_783639	0.5864485479950098
NP_786930	1.1249629775511953
NP_786931	0.9356096288085893
NP_786932	1.1752588184363697
NP_786933	3.7819240406461474
NP_786934	0.7250213430801846
NP_786935	-1
NP_786936	-1
NP_786937	1.131963748479959
NP_786938	1.4786529317184927
NP_786939	1.3078840419247824
NP_786940	1.1013343488139022
NP_786942	-1
NP_787037	1.2382756058208888
NP_787038	-1
NP_788264	0.9407377780858927
NP_788265	-1
NP_788266	0.9531131669497754
NP_788267	0.9164059941478732
NP_788268	0.8095969921461799
NP_788269	0.769719566824056
NP_789826	1.044717584274445
NP_789827	1.0604979661686644
NP_803154	0.99609023656502
NP_803156	0.5030697717558049
NP_803174	0.8251206351221945
NP_803175	0.5271693871626097
NP_803434	1.2561530012789
NP_803435	1.138700863807044
NP_808788	0.4431958889166454
NP_808789	0.8517624016376134
NP_808791	1.2194991331003417
NP_808877	1.2040862695361207
NP_821140	1.1103402476112891

NP_835194	1.3076047774743804
NP_835202	0.746388961621741
NP_835205	0.8109925927928673
NP_835280	0.7094167779413487
NP_847900	1.0507669846944325
NP_848017	0.9413975411358955
NP_848035	1.0503225232461726
NP_848036	-1
NP_849178	1.96497416552173
NP_849197	1.5446779542863525
NP_851594	1.179236855019147
NP_851596	1.6645928935449363
NP_851597	1.1246185465430205
NP_851602	0.7823231123840054
NP_851604	0.9160625659341527
NP_851605	0.6838835992564222
NP_851606	-1
NP_852027	1.063274395164552
NP_852028	0.9146232938425977
NP_852029	0.9041896018276336
NP_852030	1.1840636991806586
NP_852031	1.0657650951167763
NP_852033	1.6996791454568123
NP_852034	-1
NP_852035	0.9238106942041211
NP_852036	1.315070695942297
NP_852038	1.11265355901439
NP_852039	1.2494022639859506
NP_852040	0.8596747540103165
NP_852043	1.4877432562401822
NP_852044	1.0185869110201795
NP_852045	1.241016529016377
NP_852046	0.8745733403550617
NP_852053	1.0338470838936407
NP_852098	0.36809033792176776
NP_852105	1.7060757105298892
NP_852140	19.76706793857007
NP_852142	0.6893296872190005
NP_852143	1.3462890721816176
NP_853528	1.3869664035886569
NP_853657	0.7768209337118691
NP_853658	0.7786897266383956
NP_853659	1.1673084832442857
NP_853662	0.8814088590746744
NP_853665	-1
NP_853666	0.9941679399022421
NP_853667	1.162978141760575
NP_853668	1.0079685107980128
NP_853669	2.030098326924446
NP_853670	1.1318696186902908
NP_859044	-1
NP_859045	1.4086517721988738
NP_861434	-1
NP_861435	1.5264209694318878
NP_861543	1.1983641585294844
NP_861544	0.6306524132405503
NP_872279	0.9998566515658877
NP_872608	0.5867302584049685
NP_872609	0.6194459386717621
NP_872613	0.6503728360121693
NP_872614	1.0524211602313067
NP_872615	0.8759776240500377

NP_877401	-1
NP_877966	1.1685218182547812
NP_877968	1.3916161259390087
NP_877969	0.8330431339687211
NP_877971	0.8073087525002886
NP_878262	1.0749805018796905
NP_878263	0.8467397084091965
NP_878264	1.108058888879153
NP_891994	-1
NP_891996	1.2516480097684801
NP_891999	-1
NP_898874	1.8024501716444632
NP_898876	1.1683702783320224
NP_899154	1.2058713135693182
NP_899157	0.9000184971201373
NP_899158	0.6374181016718006
NP_899159	1.6688970245142356
NP_899162	-1
NP_899652	1.2461174776174515
NP_899653	-1
NP_908937	1.0599086142344154
NP_908938	0.7836960727740659
NP_919443	1.8198682717009833
NP_937764	-1
NP_941958	0.9981353586704748
NP_942023	0.8156806565804321
NP_942024	-1
NP_942025	1.0114503911420711
NP_942027	1.028180789837564
NP_942028	1.2170864065938443
NP_942030	1.0354644009792437
NP_942031	1.3819317910977782
NP_942032	1.6031513460550748
NP_942035	1.475155372078554
NP_942036	1.3756444791294917
NP_942037	0.7948940092074454
NP_942039	0.8574010353291324
NP_942040	0.9030328578588401
NP_942041	1.2917709598646903
NP_942042	1.0405762295284837
NP_942044	-1
NP_942045	-1
NP_942046	1.0528391886862412
NP_942048	1.1338098992613654
NP_942049	-1
NP_942051	1.0829042346973368
NP_942052	0.9195847900662631
NP_942053	1.1178632974450275
NP_942054	-1
NP_942056	1.2058513577750472
NP_942057	1.2242007246562205
NP_942060	0.9017116861274213
NP_942062	1.1700538920905585
NP_942067	1.0671865100789446
NP_942069	0.8447597567379789
NP_942072	1.0779092356958009
NP_942075	1.0886418627035808
NP_942080	1.0702562615445457
NP_942082	0.8160422811553922
NP_942083	1.031158236154567
NP_942084	0.9294648282022799
NP_942085	0.6817238250854825

NP_942086	1.2053221778048586
NP_945323	1.1226372579005315
NP_954512	1.2759105017805548
NP_954513	-1
NP_954515	-1
NP_954516	1.0609505392426433
NP_954517	1.0802516370465
NP_954518	-1
NP_954521	0.8252470048531914
NP_954524	0.5074233844363307
NP_954526	1.1075200184175997
NP_954527	1.202310889663616
NP_954528	1.1892671806026607
NP_954530	0.9771951904186813
NP_954531	1.0299535895810823
NP_954533	1.129203776538493
NP_954537	0.9342204898709113
NP_954541	1.1876406101995556
NP_954543	1.8888068489270344
NP_954545	1.7400865550304676
NP_954547	1.1728908938359008
NP_954548	1.3691260385571287
NP_954549	0.7688780195713009
NP_954550	0.9953392754268112
NP_954677	1.0233841628226905
NP_954678	-1
NP_954862	0.8823167710662294
NP_954865	0.801102806543927
NP_954887	-1
NP_954889	1.2593800126484245
NP_954891	1.160729326409317
NP_955402	1.3925343188905461
NP_955404	0.8900786942492563
NP_955407	0.6356902800186398
NP_955408	0.6476720827540773
NP_955409	1.2896872728839304
NP_955410	1.1353430142539274
NP_955411	0.7451842218219823
NP_955412	1.094743904669812
NP_955413	0.7089244555241914
NP_955415	0.9113319025652155
NP_955416	0.9595219357848775
NP_955417	1.0235605890239903
NP_955418	1.1451147028287447
NP_955419	1.2755566547242552
NP_955420	0.8934852062481276
NP_955423	1.0587021793236795
NP_955424	0.9416102873059495
NP_955425	1.0815719946650737
NP_955427	0.839523385567481
NP_955431	0.9339942912359398
NP_955432	1.4735405331555083
NP_955435	0.9756228793566183
NP_955436	0.9515168512376344
NP_955439	1.0841101218959093
NP_955440	0.4681207406497652
NP_955441	1.004690083545184
NP_955442	0.8951089740323823
NP_955444	1.5116093484322037
NP_955750	0.863243516345234
NP_955783	1.116226299146626
NP_955786	-1

NP_955789	0.8665490279801794
NP_955790	1.0491716856000786
NP_955792	-1
NP_955796	1.0559269517084657
NP_958427	-1
NP_958429	-1
NP_958818	1.0812075032064161
NP_958819	1.1915800789098905
NP_958820	0.8689205807118109
NP_958821	0.856742485440714
NP_958822	-1
NP_958824	0.8909350567447153
NP_963854	1.348709200531963
NP_963856	-1
NP_973718	0.8844858489983828
NP_976070	-1
NP_976078	-1
NP_976079	-1
NP_976080	0.77925843088307
NP_976242	1.1872193513296243
NP_981955	0.8501225985895785
NP_996538	0.8553862018923407
NP_996732	-1
NP_997469	1.1144059582492514
NP_997471	1.23642762756792
NP_997472	0.6485259097220865
NP_997474	1.1216772545191127
NP_997475	0.8036737183167105
NP_997476	-1
NP_997477	0.7867405916826278
NP_997478	1.1847855376905994
NP_997480	-1
NP_997481	1.154168675208891
NP_997484	1.148417765546397
NP_997486	1.9565098306714905
NP_997487	1.1758091098859849
NP_997490	0.9295406417644604
NP_997491	1.3656134718132247
NP_997492	-1
NP_997496	1.2089901030510182
NP_997624	1.395587143278558
NP_997627	1.3429138703635608
NP_997628	1.082204724271776
NP_997631	-1
NP_997653	-1
NP_997656	1.1491211489950617
NP_997659	1.1144541469173843
NP_997660	1.577230238761721
NP_997661	1.2365245868427583
NP_997665	1.1176013984830255
NP_997666	0.9701849284215648
NP_997669	2.30260783306833
NP_997670	1.6446561498572512
NP_997671	1.0812325473258075
NP_997675	1.0892341260216847
NP_997676	1.0460300098801656
NP_997679	0.9760038875166175
NP_997680	0.9578217417664937
NP_997681	0.8778247706343176
NP_997682	0.7746799026235056
NP_997686	-1
NP_997690	1.5958732681588994

NP_997696	1.138227457029501
NP_997697	0.7736585580063163
NP_997699	1.4206544174906774
NP_997703	2.192802413058508
NP_997706	-1
NP_997711	-1
NP_997713	1.1814446429014713
NP_997714	1.2599044925680671
NP_998722	1.1359496928445931
NP_998724	1.2785587228286586
NP_998730	0.7144480557165478
NP_998732	1.0814658651987938
NP_998775	2.1073975329388595
NP_998790	1.0956378666668962
XP_212687	1.1172298771061024
XP_212689	1.0790042565899325
XP_212748	1.0920247228172602
XP_212806	-1
XP_212873	0.9562931029203451
XP_212882	1.0227856148092742
XP_212936	-1
XP_212940	1.0312019960999201
XP_212972	1.2859844866191235
XP_213006	1.1388952189840813
XP_213050	1.0948624118452752
XP_213053	1.1572212856534236
XP_213072	-1
XP_213105	1.1260187695152353
XP_213108	1.1733897033910476
XP_213132	1.2194498793831494
XP_213170	1.3319805428438072
XP_213196	0.9876530371149591
XP_213205	0.8508174239327264
XP_213211	1.0678799713227696
XP_213213	1.0893198713323782
XP_213214	0.763549123112397
XP_213215	0.7824945280647225
XP_213217	1.018756514754219
XP_213218	1.1315927540822919
XP_213222	1.5187016542566532
XP_213228	1.004172732133331
XP_213230	0.9297002064391333
XP_213231	-1
XP_213234	0.6981046463298949
XP_213235	0.9171564961457678
XP_213237	0.8351138057541618
XP_213239	0.8887474572384318
XP_213240	0.7363555467350779
XP_213242	0.831160510231454
XP_213244	0.9722141699050572
XP_213250	0.9135819368671289
XP_213253	-1
XP_213257	-1
XP_213261	0.7246770942170114
XP_213263	-1
XP_213267	1.0387903204676752
XP_213270	0.9891154735966813
XP_213271	1.053698959997898
XP_213273	-1
XP_213276	0.871508094904342
XP_213280	-1
XP_213283	1.1466130224800999

XP_213287	1.0878588904590853
XP_213293	0.8899076804884295
XP_213295	0.9975831830619981
XP_213307	0.8550410960973176
XP_213308	0.3815205047055308
XP_213314	0.603141566868655
XP_213318	0.3324272270661444
XP_213321	0.8544461830293816
XP_213324	0.6923781232638849
XP_213328	0.7784017147760532
XP_213329	1.2401168770250224
XP_213330	1.0036241803838493
XP_213333	1.1331409738399016
XP_213334	1.1368826467662905
XP_213336	1.2039348018847633
XP_213338	-1
XP_213342	0.9262795495146422
XP_213343	1.188477105168748
XP_213345	0.4241949459039243
XP_213346	1.0699807840482487
XP_213347	0.6682791914442459
XP_213354	1.1282729235340423
XP_213357	0.8666091464376562
XP_213362	1.1532766873913802
XP_213365	1.1882571528904848
XP_213367	0.6927926937633703
XP_213368	0.917778475635164
XP_213369	1.043957722321731
XP_213370	1.0772193411067243
XP_213372	1.9484478773171026
XP_213376	1.239896568962723
XP_213377	0.9320431672982198
XP_213382	0.8893611915225704
XP_213385	1.0762803016751084
XP_213394	0.8719803120028428
XP_213395	1.175382246586068
XP_213396	0.9233572190343826
XP_213403	1.0217698295584003
XP_213406	0.6989439341027267
XP_213407	1.113883240413331
XP_213410	1.0081694946161648
XP_213411	0.9157961224778178
XP_213413	0.6839403338531382
XP_213415	1.021325503575512
XP_213416	1.6059589321057623
XP_213418	1.100979939276307
XP_213421	1.6733237794410447
XP_213423	1.1115305779542355
XP_213426	1.329101759639851
XP_213427	0.9657410275939582
XP_213429	-1
XP_213430	1.2764500410833417
XP_213433	-1
XP_213437	1.128469376587135
XP_213439	0.796108190018344
XP_213440	0.7089064486689733
XP_213443	0.7936397277824744
XP_213445	0.9831537081561667
XP_213446	0.7844914421187871
XP_213447	0.7770623256929413
XP_213456	1.252840691204387
XP_213459	1.0642438927830902

XP_213461	1.242041168812686
XP_213463	1.1018225390872967
XP_213465	0.878474142062005
XP_213467	1.0711323115585467
XP_213468	-1
XP_213469	1.1545352346822872
XP_213483	1.1149136867452676
XP_213484	-1
XP_213487	1.1041145130691195
XP_213488	-1
XP_213491	-1
XP_213492	1.1482054136178599
XP_213493	0.7890267707931293
XP_213506	1.2569287671404898
XP_213508	1.3015539965912304
XP_213509	-1
XP_213511	1.2479413620607478
XP_213513	0.8022229667754766
XP_213514	1.6342349082602403
XP_213516	0.9685104270103384
XP_213518	1.577110705819545
XP_213526	0.9529675026217466
XP_213532	-1
XP_213534	1.173313025185557
XP_213535	-1
XP_213539	1.4358373229025634
XP_213542	0.9653528248876597
XP_213560	1.2332758755660556
XP_213564	0.960822262400613
XP_213567	0.4100081628886159
XP_213569	0.9188101830267026
XP_213573	1.1030696045550643
XP_213574	0.741750826162812
XP_213578	0.7517195314595589
XP_213581	0.9887168452228674
XP_213584	-1
XP_213585	0.7700330461421906
XP_213586	1.0244750741237263
XP_213598	1.0045129593188646
XP_213600	2.391573629333126
XP_213602	0.6630532733354972
XP_213604	0.9386234293419222
XP_213608	0.9008086787036949
XP_213610	0.7866323547695354
XP_213612	1.1997940279489876
XP_213618	0.796779571689784
XP_213619	0.9118056177091383
XP_213621	-1
XP_213633	-1
XP_213637	0.7184386092836627
XP_213638	0.8844600354609513
XP_213639	1.0779086399411177
XP_213645	0.7643889045527965
XP_213649	0.8486104198942922
XP_213650	1.1238906066278627
XP_213651	0.7680887955554554
XP_213656	0.987726605506213
XP_213658	1.0339382912291468
XP_213664	1.0967777544120578
XP_213670	0.6362404336204944
XP_213672	1.502016521944725
XP_213677	0.6429269352078841

XP_213679	1.2786092727495666
XP_213684	0.9790606186947224
XP_213685	0.9484206439615467
XP_213690	0.8181386439403678
XP_213692	1.005009104104121
XP_213700	0.8577036786477823
XP_213701	1.2842990023164103
XP_213702	1.098701221843535
XP_213706	0.9857433035149513
XP_213710	0.9739787428204466
XP_213712	1.2074905960742675
XP_213713	1.042712006503751
XP_213716	0.6294730535577633
XP_213717	1.0595755707848549
XP_213719	1.1106556625645996
XP_213723	1.4186328683294143
XP_213725	1.1776504745234442
XP_213726	0.873340927156594
XP_213727	2.1164531363811365
XP_213729	1.4085802134587093
XP_213733	0.868604025339006
XP_213736	1.2893321922591987
XP_213738	0.7544042074558462
XP_213742	0.5346427977558738
XP_213746	0.7090385794723884
XP_213751	1.6515819284249302
XP_213758	1.0274346255650646
XP_213761	-1
XP_213762	0.5891933405725343
XP_213765	1.2374560698003234
XP_213769	1.2853124967777678
XP_213773	1.1738937776019138
XP_213774	0.871565467773939
XP_213777	1.3503730740714988
XP_213779	0.9709242846355589
XP_213782	0.9101007018397681
XP_213783	0.8810846187392963
XP_213790	0.8054341861549733
XP_213791	0.3843014447270839
XP_213793	0.8546735382133308
XP_213794	0.819783676433405
XP_213797	1.1528555942625256
XP_213799	1.0263344536063796
XP_213800	1.152837818061451
XP_213807	1.0249258934019398
XP_213811	0.9955445566030874
XP_213821	0.9113470763134223
XP_213823	0.8643211385311792
XP_213824	1.1912477914601818
XP_213831	4.218038471435644
XP_213832	0.9079836118133033
XP_213833	0.6937884123057406
XP_213835	1.0449953830702197
XP_213840	0.9963585333409964
XP_213841	0.9645680792193262
XP_213842	0.7711774194396459
XP_213843	0.9204906674783446
XP_213844	1.152237464337205
XP_213848	0.9844078889289518
XP_213849	1.1143754607218908
XP_213876	0.4834293557462523
XP_213879	1.5202041044929657

XP_213886	1.007057015600237
XP_213890	0.8309519707130498
XP_213893	-1
XP_213895	0.7336467422599199
XP_213896	1.1383827224738043
XP_213898	0.6701421356897218
XP_213900	1.0174646271024277
XP_213902	1.5230142321667082
XP_213903	0.8848066737727142
XP_213907	1.1508188699011388
XP_213910	-1
XP_213912	1.0643816233318644
XP_213919	1.4083871921449447
XP_213920	0.817746979260027
XP_213921	1.6744254918089017
XP_213922	0.6188122948195468
XP_213925	1.0527498214042466
XP_213926	0.9321897795531408
XP_213927	1.0483930299545474
XP_213930	0.9959356006353297
XP_213936	0.9056553166175196
XP_213940	0.8089254186775984
XP_213943	1.039581556692321
XP_213944	0.5860480595700808
XP_213949	0.9208041207128695
XP_213950	1.095788448611806
XP_213951	0.8850176699264516
XP_213954	0.7150037926012952
XP_213960	0.8871375501993984
XP_213963	0.7490967398379795
XP_213964	1.0162058558261755
XP_213967	0.937595125150235
XP_213969	0.9327581263680719
XP_213972	0.755548439619011
XP_213976	1.0853984362214186
XP_213979	1.940080209533661
XP_213982	0.7400887930411594
XP_213983	0.738732982757638
XP_213984	1.1012500128748992
XP_213992	1.0867006530538061
XP_213995	1.5165486363151819
XP_213997	0.19578746365746783
XP_213999	-1
XP_214001	1.0940308687112263
XP_214004	0.7354884441494659
XP_214006	1.2385058172343466
XP_214007	0.997205223427399
XP_214008	1.1253298547002901
XP_214011	1.2316047706986681
XP_214012	1.1639968753865813
XP_214013	1.282175914605258
XP_214014	1.0766998628246798
XP_214017	1.2480711491159355
XP_214021	1.0792155222660773
XP_214030	1.4647668836625864
XP_214031	0.4382501513605546
XP_214035	1.4844992004186164
XP_214042	1.3945104171861509
XP_214043	1.1518043579241715
XP_214047	0.7363451216013336
XP_214048	1.0828403056513005
XP_214050	1.3029260293483513

XP_214053	1.5243793963770442
XP_214059	0.9144607707818705
XP_214061	0.9711657412561049
XP_214063	0.9534450358507733
XP_214067	0.8538511511595394
XP_214069	0.9892819551581415
XP_214071	0.9030529512260695
XP_214072	-1
XP_214074	-1
XP_214078	0.9797689911853248
XP_214079	1.1849027756474055
XP_214085	-1
XP_214087	0.7599569971615786
XP_214090	1.070755990416891
XP_214092	1.0016963833611388
XP_214093	1.1378560306265555
XP_214096	0.9671843178383331
XP_214099	1.0203559490914518
XP_214102	-1
XP_214108	1.149602158738188
XP_214110	1.3307913681464099
XP_214120	1.4787175067406455
XP_214121	1.0158106049796052
XP_214130	1.250262304423371
XP_214132	0.7996638655659559
XP_214137	0.8425005162022533
XP_214148	1.0247008097489487
XP_214152	1.4426893031947328
XP_214153	1.0154450836634141
XP_214155	0.9610718111801968
XP_214157	0.8519134416593076
XP_214160	1.1415554111259603
XP_214163	0.8513031998656803
XP_214169	1.0694834987085216
XP_214173	0.9174253680102757
XP_214175	1.0813434883841253
XP_214181	0.8202491142611281
XP_214182	0.9220600768561448
XP_214185	0.6806928937915119
XP_214189	-1
XP_214196	2.0494166148888735
XP_214197	0.8877270099791884
XP_214199	0.8311250022618828
XP_214202	0.870513945881311
XP_214203	1.0006726020590206
XP_214211	-1
XP_214213	0.8477780832952879
XP_214215	1.2140945942643433
XP_214216	1.7533680151778273
XP_214217	-1
XP_214221	0.7495308348391004
XP_214234	0.9619566861389472
XP_214237	1.0600505492424082
XP_214238	0.6957991580710491
XP_214241	0.775277396964249
XP_214242	0.833622518540814
XP_214245	0.9686497864194195
XP_214250	0.8798052902148633
XP_214253	1.130064247368954
XP_214258	1.1373352887687425
XP_214266	0.6463264810434496
XP_214273	1.2075444533066508

XP_214276	0.8797967492523089
XP_214278	-1
XP_214279	2.4698068701484908
XP_214284	0.9779158034453591
XP_214288	0.9402674407251381
XP_214290	0.7317034090676598
XP_214293	0.8233120034473814
XP_214295	0.5268961939626889
XP_214296	0.5391138612580296
XP_214298	1.1482310367168291
XP_214300	0.9055057415556916
XP_214304	0.7693911630388309
XP_214305	0.6442593504775583
XP_214306	1.1181700844041609
XP_214307	0.8326909154619629
XP_214309	0.6386799261968913
XP_214310	1.2040038469620595
XP_214312	1.17486862019367
XP_214313	1.2551514066215328
XP_214316	0.7490623950740701
XP_214317	1.0172353999420667
XP_214318	0.7681831335012873
XP_214319	-1
XP_214320	0.9498205864885184
XP_214321	0.724993871761807
XP_214322	1.3251466902076554
XP_214331	-1
XP_214332	0.819586689827155
XP_214334	0.7379132294031691
XP_214338	0.9752920816028489
XP_214339	0.8446748705389338
XP_214349	0.8201573966817244
XP_214350	1.5247636063625625
XP_214353	0.8754134249854914
XP_214355	0.3456610861308667
XP_214362	0.9979395171231454
XP_214365	1.1482494922689621
XP_214366	1.0530867954023453
XP_214369	1.8543976360985341
XP_214372	1.2620397496435707
XP_214374	1.2693719152086738
XP_214377	0.9858436003667522
XP_214380	1.5950084913319562
XP_214381	1.2929536227711542
XP_214382	0.8992268761387131
XP_214383	1.386690825965036
XP_214386	-1
XP_214392	1.2260377641465057
XP_214393	0.8099911248605971
XP_214400	0.6556769968150816
XP_214403	0.7047748066622126
XP_214404	1.1984357565294639
XP_214412	-1
XP_214413	-1
XP_214416	0.6416187742343467
XP_214417	0.8283991172178823
XP_214420	1.6782650878252685
XP_214423	1.1342948381803628
XP_214427	1.1514736093817666
XP_214428	0.8662143182366798
XP_214431	0.9141083960576988
XP_214433	0.8899713290370822

XP_214434	1.1323200100083433
XP_214440	1.9194393428144005
XP_214441	0.28613102552284664
XP_214443	1.89808045370572
XP_214446	-1
XP_214448	1.042224070018327
XP_214451	0.8959911735079423
XP_214454	-1
XP_214459	1.0789934427067298
XP_214460	1.2870401077933196
XP_214469	15.519859993501683
XP_214475	1.0360708497865725
XP_214478	1.0865982979004294
XP_214485	0.9952526340132967
XP_214491	0.8024419241445804
XP_214499	0.9704541803972027
XP_214505	0.8697158437751308
XP_214514	0.986319698223666
XP_214518	0.9843166426438977
XP_214522	1.05989737308939
XP_214524	0.9347387880651266
XP_214525	1.0416028644058437
XP_214526	0.9495449321625467
XP_214528	0.9019641088567664
XP_214529	0.9475090912953178
XP_214535	0.9425840764154895
XP_214539	0.9185186767201641
XP_214541	0.7905550465580671
XP_214550	0.9547024763932795
XP_214551	1.1077218441734826
XP_214552	1.0229100616727316
XP_214553	1.1753054095870683
XP_214554	0.7940927251077726
XP_214557	0.778021096151143
XP_214564	0.930957399559695
XP_214566	0.8286650685207079
XP_214569	-1
XP_214570	0.5046170551184752
XP_214574	0.9353255314639438
XP_214583	1.012355927279943
XP_214584	0.8919038157104171
XP_214590	0.9768072323156652
XP_214596	1.4799278110753329
XP_214599	0.7257653668975729
XP_214601	0.8537312665840303
XP_214605	1.07045232080723
XP_214614	0.8492523888309691
XP_214617	1.1987750842382505
XP_214622	0.8991349421462703
XP_214625	0.926217624293553
XP_214626	0.9915264910200916
XP_214630	0.8107024481342363
XP_214633	0.14873770979384957
XP_214635	1.0704162807264466
XP_214639	0.8534584107347497
XP_214640	0.8224204032583845
XP_214646	1.0961164982830014
XP_214655	0.8273200117498801
XP_214656	0.9232040342620156
XP_214659	1.4002463737801618
XP_214662	1.3133348594407392
XP_214663	1.0787155705771347

XP_214664	0.8036796096324847
XP_214665	1.1267245575310267
XP_214666	0.8756098297246092
XP_214667	0.8510685522303305
XP_214668	0.9331696198179055
XP_214669	1.492987017450209
XP_214671	1.0951128775219288
XP_214672	0.9298203333149657
XP_214673	0.9102275074418428
XP_214677	-1
XP_214678	1.7916955181115732
XP_214687	0.8747593310464118
XP_214688	-1
XP_214690	1.1465830601113813
XP_214697	1.0194510784950932
XP_214698	1.3067206664418038
XP_214702	1.0106081425809963
XP_214703	0.9392863799484861
XP_214704	0.8879300768442526
XP_214707	1.1580644111745286
XP_214712	0.49525192575779275
XP_214713	1.2271854866110201
XP_214715	0.8259681549543569
XP_214721	0.8613777443224904
XP_214730	0.9474044865480508
XP_214735	1.1055662897726402
XP_214737	1.2768298119686463
XP_214744	0.6985495141862531
XP_214749	0.9109401647549453
XP_214751	1.0286477345949787
XP_214757	0.9282689620109134
XP_214769	1.0245001904438993
XP_214774	1.0697866355579515
XP_214778	0.7370537926101183
XP_214780	1.0517153182458552
XP_214790	1.7258367634194982
XP_214797	1.0027413254252728
XP_214798	1.6942639361562033
XP_214805	1.2693422006814297
XP_214806	1.1968344449267156
XP_214811	1.2086951683157128
XP_214812	1.1826914563739954
XP_214813	1.999773656013999
XP_214817	1.3560522394309371
XP_214819	0.862608625373508
XP_214825	1.0960475762723279
XP_214831	0.7953382929947918
XP_214833	1.468222786597383
XP_214834	0.8616269764621683
XP_214835	1.2743630353705273
XP_214836	0.933327518307086
XP_214838	1.2805918467276731
XP_214840	-1
XP_214842	1.0378512979122714
XP_214843	0.8656358055387537
XP_214845	1.0926945469951586
XP_214847	0.7128752049688649
XP_214853	-1
XP_214859	1.1724376245822885
XP_214862	0.9980771430838197
XP_214863	0.9655927618603637
XP_214868	0.8089623767068131

XP_214873	1.0998147187674772
XP_214874	1.1229147364387455
XP_214875	1.1207136932973485
XP_214878	1.0032101009408345
XP_214883	-1
XP_214886	0.9169125389012059
XP_214888	0.8766614748285205
XP_214890	0.8581140045115592
XP_214895	0.9720644180402629
XP_214896	1.0737792599382476
XP_214897	0.6504669651183931
XP_214898	1.4945434531913422
XP_214899	1.0826509744686874
XP_214902	1.0587560897176977
XP_214906	-1
XP_214907	0.8801522412757291
XP_214910	1.060190065553714
XP_214911	0.9981692075554083
XP_214912	1.3433536461855153
XP_214914	1.040064171701276
XP_214916	1.0034129380722816
XP_214924	1.1645733303229773
XP_214925	0.9745876302867024
XP_214926	1.1300991987750235
XP_214927	1.1391090717884576
XP_214929	0.5139518346937907
XP_214931	-1
XP_214935	-1
XP_214939	1.5529610107262706
XP_214940	2.3865435595603612
XP_214945	-1
XP_214948	0.9345849976807105
XP_214950	1.0600245394890433
XP_214951	1.025196281004075
XP_214953	-1
XP_214954	0.8260036554163701
XP_214962	1.1372223927887235
XP_214963	0.8510608484666199
XP_214967	0.9234374928458946
XP_214969	1.36227968669906
XP_214972	1.1493844933959079
XP_214973	1.3416720257310326
XP_214975	1.4757230708786861
XP_214979	2.007664502188496
XP_214980	1.091755272218356
XP_214982	1.2356615188129385
XP_214983	1.2715462626357246
XP_214986	1.4302281064151448
XP_214991	1.2198091991800286
XP_214992	2.3875211878673155
XP_214993	-1
XP_214994	1.203394760400056
XP_214995	1.0911033644107888
XP_214996	1.1165247186198974
XP_214998	0.9881584782228044
XP_215000	1.8019012849316942
XP_215001	0.9594095483901026
XP_215003	1.0680440186839926
XP_215005	3.146340265920138
XP_215009	1.0427238168299031
XP_215011	1.3545810301824002
XP_215012	0.8337619040409193

XP_215013	1.1633338768829862
XP_215016	1.155732324258441
XP_215023	1.195408447998837
XP_215026	1.4676948201498892
XP_215027	0.7858864657076364
XP_215028	1.0400207411124878
XP_215033	0.9251711103463642
XP_215034	0.8120000292278563
XP_215037	1.0223147609052894
XP_215038	1.2615617705315392
XP_215041	1.1311224193478557
XP_215043	-1
XP_215044	1.167236660087289
XP_215045	0.9255145114133757
XP_215046	0.7402379740519864
XP_215047	1.2137336324565537
XP_215054	2.50256576191259
XP_215057	1.1519319982146172
XP_215059	-1
XP_215064	1.023848335969114
XP_215069	0.9556508063635157
XP_215070	1.7358800235432732
XP_215071	-1
XP_215074	1.1344966771589875
XP_215075	0.9082106393268906
XP_215076	0.9378675999101342
XP_215080	1.1509407932442421
XP_215089	1.153930127756917
XP_215090	0.9158852199977788
XP_215106	-1
XP_215108	1.1161501102710587
XP_215113	1.0100618211849124
XP_215117	4.58198887976501
XP_215121	0.8414165204150098
XP_215123	1.060914135341087
XP_215127	1.1011519769492741
XP_215128	1.1542051016236494
XP_215130	-1
XP_215134	1.0000737446353638
XP_215136	1.1736904034593068
XP_215139	0.5817665124972079
XP_215142	0.7763306039461031
XP_215145	0.7639303431543092
XP_215147	0.8817546957905944
XP_215148	0.9006081654618943
XP_215154	0.9896647541628282
XP_215159	0.7956202867398058
XP_215165	1.2746723872725823
XP_215167	0.9648450931427138
XP_215168	0.7993345468678309
XP_215174	1.402189587553094
XP_215177	1.1335452868877596
XP_215178	0.8871144224747024
XP_215180	0.9612846616835792
XP_215182	1.1051776819381496
XP_215185	1.0203416273346124
XP_215186	-1
XP_215190	0.8800376577133008
XP_215192	1.1665364788040324
XP_215195	-1
XP_215196	1.216733190547286
XP_215197	0.7955855166962497

XP_215199	0.6202551640540853
XP_215206	0.8170763011762053
XP_215208	0.9924142415177938
XP_215213	1.0446369095499057
XP_215222	0.9354351222907095
XP_215225	0.9733002710499866
XP_215227	0.49998509131468877
XP_215230	-1
XP_215234	-1
XP_215240	-1
XP_215244	-1
XP_215247	0.9992302148164957
XP_215251	1.0142238530851977
XP_215254	3.3360584097363097
XP_215257	-1
XP_215259	1.2209733460355117
XP_215260	0.986506467460678
XP_215262	0.8640500426086001
XP_215264	0.6198707508261804
XP_215270	1.4148237281394966
XP_215273	1.0405363725688532
XP_215278	0.7951194495133693
XP_215285	-1
XP_215286	1.2474802059598415
XP_215293	1.7297081089138662
XP_215296	1.2825605502897277
XP_215303	1.7076813607937247
XP_215320	0.8319573077572091
XP_215323	-1
XP_215324	0.972476983877843
XP_215325	1.161542363373028
XP_215327	1.321522077594125
XP_215342	1.7874842957576156
XP_215344	1.196638517325319
XP_215351	2.2769778296057592
XP_215355	1.9784413643177297
XP_215356	-1
XP_215357	1.7421032509943313
XP_215358	0.9686359285014295
XP_215359	0.7188216217532565
XP_215362	1.1180388663284646
XP_215367	1.2636505983127078
XP_215373	1.1362901074451566
XP_215375	0.8224594223518692
XP_215376	1.0238711467540822
XP_215378	0.9461552011959151
XP_215386	0.8887763463892352
XP_215389	1.1530797332658407
XP_215390	1.0447868461383942
XP_215393	1.1927354112690458
XP_215397	0.962703127800402
XP_215401	1.1742151002590875
XP_215403	1.1976693165492942
XP_215407	1.014651907791265
XP_215413	-1
XP_215416	0.9995930752840351
XP_215419	1.064415551260535
XP_215421	-1
XP_215422	1.045926007115277
XP_215424	1.2883711915759213
XP_215432	1.227557161582418
XP_215434	1.0306111126634792

XP_215438	0.924992780967225
XP_215439	0.7737662237191025
XP_215445	-1
XP_215451	1.1138188510213887
XP_215452	1.0440556887401498
XP_215455	1.1374699976610385
XP_215465	0.9930822079806766
XP_215467	0.6169844682984124
XP_215468	1.0258400599177169
XP_215469	1.1304348332861367
XP_215475	0.9637202907084013
XP_215481	1.1554727351446605
XP_215482	0.9511774429929538
XP_215483	1.1404962134186858
XP_215485	1.1379067783458756
XP_215486	0.7799010666873619
XP_215487	1.077857962344459
XP_215495	0.018444387943722086
XP_215497	1.0358442385117
XP_215501	1.0358485680861766
XP_215503	1.2115299959772106
XP_215504	0.9960972649215236
XP_215505	1.2429947435972875
XP_215510	-1
XP_215512	1.0305794180692398
XP_215513	-1
XP_215515	1.24793526222624
XP_215516	1.3529572492745359
XP_215528	0.8543391075182933
XP_215530	1.2848008280438414
XP_215540	0.9376290152043739
XP_215541	1.007056579292938
XP_215544	0.6535078965158834
XP_215550	1.0537927599626185
XP_215551	0.4953872026232091
XP_215553	0.8686807587863727
XP_215554	0.7782524576279988
XP_215562	1.6507083325534637
XP_215564	1.1703146995852725
XP_215566	1.0219522086587833
XP_215570	1.8156308132069388
XP_215573	1.5367651100760717
XP_215575	1.171441933256824
XP_215576	6.870689140042383
XP_215578	0.8132139875580194
XP_215579	1.6709293680905486
XP_215594	0.9401861714212969
XP_215597	-1
XP_215598	1.155313148716923
XP_215600	0.8369492021700798
XP_215605	-1
XP_215607	0.5631547833014579
XP_215612	0.6962623865860281
XP_215616	0.9241359351935702
XP_215619	0.6298219278452538
XP_215626	0.8371992732880077
XP_215627	0.8159577527475697
XP_215628	0.8246505880578047
XP_215630	0.6926651071401464
XP_215635	0.9604294038921429
XP_215637	0.9625556208200184
XP_215641	1.0435297321400359

XP_215645	1.1198992565425683
XP_215648	1.3922371376361713
XP_215655	1.0535085007861678
XP_215659	1.1369269061040153
XP_215660	-1
XP_215663	1.1951249976214968
XP_215664	1.0749127813914459
XP_215666	1.1031718933790908
XP_215667	1.0376385501065328
XP_215670	0.9317242568361038
XP_215674	0.8770775882407488
XP_215677	1.1513775590647082
XP_215685	0.8513670332165404
XP_215691	0.9695585946515198
XP_215692	-1
XP_215693	1.1906032667392912
XP_215701	0.8771924010248715
XP_215705	0.734999400202914
XP_215706	1.166304103306855
XP_215717	0.9317957265250278
XP_215718	1.3633281576270708
XP_215723	0.9838829746378684
XP_215730	1.0263133400630584
XP_215733	1.1547514488171804
XP_215741	1.18065055474176
XP_215742	1.3161400040624682
XP_215747	1.1125377932376128
XP_215749	1.176256772054836
XP_215751	-1
XP_215754	0.9399565950395873
XP_215757	0.892757639685259
XP_215762	0.45049890615780797
XP_215767	1.0473296602813333
XP_215769	0.9297593077990943
XP_215771	-1
XP_215776	1.2064091908433343
XP_215777	-1
XP_215781	1.0017931145257248
XP_215783	-1
XP_215785	0.9550202458925517
XP_215787	0.916261034852233
XP_215790	0.9239435609221843
XP_215794	1.0701329680110587
XP_215797	0.9112363769605212
XP_215801	1.2321337974383222
XP_215811	0.8651845127784106
XP_215812	1.033919490527902
XP_215821	1.24014207936849
XP_215822	0.9700226071926329
XP_215826	1.2818532184983251
XP_215827	0.9348980570166601
XP_215828	0.8359687097947924
XP_215829	1.0786975736880078
XP_215831	0.9246091191232869
XP_215832	0.9141309056213225
XP_215833	0.9412993175637541
XP_215838	0.7964690036633547
XP_215839	1.2275123587030732
XP_215840	1.0423478662607752
XP_215841	0.8542532178604435
XP_215843	1.326211405100999
XP_215847	1.403291416768054

XP_215848	1.5427195288895117
XP_215851	0.5716556584253035
XP_215857	1.0214092175400593
XP_215858	1.4130999081754334
XP_215862	1.5567772815477934
XP_215872	1.2804223785271285
XP_215875	1.2327911871382224
XP_215876	-1
XP_215880	1.151671392098936
XP_215883	1.3162515293502228
XP_215885	1.1055866192059192
XP_215894	0.9638657588033821
XP_215896	0.7348322398858426
XP_215897	0.9784443515233187
XP_215903	1.425201761724403
XP_215906	1.152887992149993
XP_215908	0.9241656914454905
XP_215919	0.6480050943425898
XP_215920	1.17860346422332
XP_215921	1.1428310101155794
XP_215922	1.1141032237724597
XP_215924	1.5767871759662826
XP_215927	1.0735277515150528
XP_215931	-1
XP_215932	1.164284668131841
XP_215935	0.8953257205711749
XP_215938	-1
XP_215942	1.0484206140479286
XP_215947	1.753950342396195
XP_215949	0.9726307280751642
XP_215951	0.917138839685469
XP_215952	1.1881056248241946
XP_215953	1.5370121531772154
XP_215960	0.9460229589035443
XP_215963	1.6286326889818006
XP_215964	1.111480416017701
XP_215974	1.0812345679078723
XP_215977	-1
XP_215979	1.0157540487940981
XP_215981	0.8853883959097373
XP_215982	-1
XP_215984	1.9884837586998905
XP_215985	1.6074030510055204
XP_215991	0.9492893015030743
XP_215993	0.9104790158291026
XP_215994	0.8452781612609988
XP_216002	0.9752183019888127
XP_216010	1.0142407709433734
XP_216011	0.9962007506941357
XP_216013	0.6995949691789308
XP_216018	2.2107930439701393
XP_216019	1.414480120210237
XP_216020	-1
XP_216024	1.0576390493023642
XP_216025	0.9554848570237505
XP_216026	1.6458921146333967
XP_216034	0.9490151805497741
XP_216035	1.2359134477704143
XP_216040	1.1675336792128228
XP_216041	0.8422018583370171
XP_216042	0.6201376957203879
XP_216044	0.7289839458295967

XP_216050	1.2174824179355193
XP_216056	1.003482019829961
XP_216062	1.0233497135319332
XP_216063	1.292768943750738
XP_216079	1.141971151986062
XP_216081	2.030764259390002
XP_216095	0.8724702131546347
XP_216102	0.9925194141785799
XP_216107	-1
XP_216108	-1
XP_216112	-1
XP_216113	1.1699028462423822
XP_216124	0.9841327926037203
XP_216130	-1
XP_216135	0.7774056084508684
XP_216137	1.3611037905869463
XP_216142	0.4331095148303729
XP_216152	0.8329108463165592
XP_216169	0.8964580668946818
XP_216170	0.31475026771799564
XP_216171	1.219215169650637
XP_216173	1.0016166294285247
XP_216179	0.8953359394858539
XP_216180	0.861748738013007
XP_216181	0.7253517453412089
XP_216188	0.6617260876534033
XP_216191	0.787793776689324
XP_216192	0.6221645832009569
XP_216194	0.7269752745330854
XP_216195	0.7159119879980297
XP_216196	0.6585275585196421
XP_216198	0.8634137593540949
XP_216202	1.0210208591840586
XP_216203	0.9227499435300711
XP_216209	-1
XP_216212	0.7310655046681868
XP_216219	0.9877764372211667
XP_216220	0.7161442697816064
XP_216222	0.8137682888046115
XP_216227	1.2108969500835018
XP_216228	1.3660582233632206
XP_216230	0.9841435966063322
XP_216232	-1
XP_216240	0.8882645108830046
XP_216249	0.9570900367578257
XP_216263	0.9176375549743644
XP_216265	0.9236051555620377
XP_216269	1.07311963642609
XP_216270	1.0084356486938646
XP_216272	0.8088533254859777
XP_216274	1.1261757462138287
XP_216276	0.6567453981214288
XP_216278	0.5706927915064705
XP_216283	1.135394172626529
XP_216287	0.893295457485015
XP_216299	1.2710970020738657
XP_216306	1.10323186944128
XP_216309	0.9114085904391276
XP_216310	-1
XP_216312	2.4833155741381274
XP_216316	1.2744802079388875
XP_216317	0.8903077866883543

XP_216318	1.1652859112410372
XP_216323	1.090369887403609
XP_216331	1.110205383182451
XP_216340	1.4794059016159282
XP_216345	-1
XP_216347	0.9173344018409283
XP_216349	0.6095266587769207
XP_216357	1.2412910790772975
XP_216362	0.7590941563361802
XP_216363	-1
XP_216366	-1
XP_216368	0.9485076732822189
XP_216369	1.27221679184436
XP_216371	1.199591458939416
XP_216372	1.051989687790634
XP_216376	0.6141551227279487
XP_216377	0.8813154654989309
XP_216378	0.876342082629816
XP_216388	-1
XP_216396	0.7004782956053641
XP_216398	0.8349828637443895
XP_216400	0.7555307342356969
XP_216401	-1
XP_216403	-1
XP_216407	1.5888060021053896
XP_216408	0.9339019682483518
XP_216410	0.5202092806789965
XP_216411	1.0024457619232399
XP_216415	0.8319323784032261
XP_216417	-1
XP_216418	0.8073872697816107
XP_216422	1.4272454563370542
XP_216433	1.010421574673817
XP_216438	1.082271184000902
XP_216439	0.7387470838937636
XP_216443	-1
XP_216444	0.8758824412556035
XP_216446	0.9725119032409678
XP_216454	1.4020342812433717
XP_216457	0.7048662068599351
XP_216459	-1
XP_216466	0.9424692929817873
XP_216471	-1
XP_216473	0.8149075426006308
XP_216477	0.9436186093599289
XP_216479	-1
XP_216480	0.7750408641924595
XP_216482	0.9523059793052516
XP_216483	-1
XP_216484	1.035578254765696
XP_216485	0.8734096557857544
XP_216490	1.0515291349090123
XP_216492	0.8994031141755322
XP_216499	0.9471168128664651
XP_216504	1.052012487637805
XP_216505	0.8070039896864807
XP_216509	0.9638576838591639
XP_216510	1.0879818387796127
XP_216515	-1
XP_216516	1.0236879682455315
XP_216517	1.0231387509769736
XP_216518	-1

XP_216521	0.9682475894886052
XP_216524	0.870151352357482
XP_216525	-1
XP_216526	1.1139582583507803
XP_216527	0.9333546216385514
XP_216529	1.0845273025153945
XP_216537	-1
XP_216539	1.0544088487336039
XP_216541	1.0666856229939055
XP_216542	0.9055213857654024
XP_216543	1.0514936906649879
XP_216545	0.8476922475251695
XP_216546	1.2756662678248132
XP_216547	0.7811560673220016
XP_216548	-1
XP_216552	0.7088233365297576
XP_216558	1.054575659159491
XP_216563	0.8176587710044221
XP_216565	1.2435371544457567
XP_216567	0.8098279869011868
XP_216577	1.1748733536486844
XP_216580	-1
XP_216584	-1
XP_216586	1.5817179313258596
XP_216589	0.8272940701708257
XP_216592	-1
XP_216596	0.9564626399500534
XP_216597	1.0143941698776595
XP_216599	0.7067673276686164
XP_216604	1.0912952836773775
XP_216606	0.7367017684796954
XP_216607	-1
XP_216609	0.8116797848328036
XP_216610	0.9238474683517229
XP_216611	0.9906973624377113
XP_216616	0.805336948554242
XP_216632	0.6091127474969938
XP_216633	0.9489984485984513
XP_216634	1.6400470990459866
XP_216635	1.0087311715750145
XP_216638	0.8700392902933501
XP_216643	0.8089727251551245
XP_216644	1.3840155832644065
XP_216648	0.8257696696323628
XP_216650	0.6899815685551832
XP_216651	0.9803500871771998
XP_216656	0.8250544856214829
XP_216661	1.0381510632161366
XP_216665	0.20011316644356467
XP_216666	1.083233660033615
XP_216667	0.9356910973093968
XP_216670	1.0101525960555933
XP_216672	0.8755176735609198
XP_216677	1.6153604034642546
XP_216678	2.0904802294680667
XP_216679	0.7520457743820578
XP_216689	0.7696049773785781
XP_216701	1.0061119987903393
XP_216704	-1
XP_216706	0.910409561928999
XP_216707	-1
XP_216709	0.9336153909929148

XP_216716	1.1742584235846876
XP_216717	0.7627470820842173
XP_216720	-1
XP_216721	1.0248500257966129
XP_216729	0.9147974092759056
XP_216733	0.990542512915678
XP_216738	1.1571622346363966
XP_216739	1.056400284393184
XP_216740	0.8105698810232621
XP_216741	1.1378210255746088
XP_216744	0.9142640401299338
XP_216747	1.0097357349734177
XP_216748	0.9482312945525412
XP_216750	1.0611500498794821
XP_216751	1.0744953262469727
XP_216752	0.7576005412181388
XP_216755	1.054381847369963
XP_216757	1.3450770996026358
XP_216759	1.0012032132099464
XP_216762	1.0980628812849933
XP_216763	0.8123656915582563
XP_216765	0.9163982463736802
XP_216766	1.4017012250866412
XP_216776	2.1961565238713376
XP_216777	-1
XP_216781	1.1480113456176935
XP_216782	-1
XP_216784	1.1373574275663247
XP_216785	0.9182358324601952
XP_216787	0.9441253854040981
XP_216791	1.6690259975008523
XP_216799	0.7847966729025866
XP_216800	0.45146032429985145
XP_216802	-1
XP_216805	1.0440923583649289
XP_216815	0.8349799914892264
XP_216820	1.049819402357904
XP_216827	0.9045180261084559
XP_216828	0.8843610381192647
XP_216835	0.8854973951655971
XP_216836	0.8323192515149552
XP_216837	1.0072210138525752
XP_216839	0.7298120995223812
XP_216841	0.7306471139065168
XP_216844	1.1352476791457902
XP_216845	1.039687528023562
XP_216848	-1
XP_216850	1.3288270490540215
XP_216854	0.6078891819978128
XP_216859	0.852631093203267
XP_216862	1.3857629367353903
XP_216872	0.9055738468079495
XP_216874	-1
XP_216880	0.8756480828541333
XP_216882	0.8674120438204102
XP_216886	0.8209678171380953
XP_216890	1.0392897576829705
XP_216892	1.3494870350718702
XP_216893	1.1250639185072537
XP_216899	1.8762511050942834
XP_216900	1.0712347399368871
XP_216904	0.6292696293207004

XP_216905	1.7254292548187617
XP_216910	1.2368566054777796
XP_216911	1.1731188870278408
XP_216913	0.8790519278762456
XP_216915	1.069189935018583
XP_216921	1.0922371453980417
XP_216922	1.0587525988097812
XP_216927	0.9495224075406664
XP_216928	1.0055566082639518
XP_216929	0.934632381893925
XP_216934	0.9700559320927875
XP_216938	1.2592615939472145
XP_216940	1.438010665192856
XP_216941	-1
XP_216944	0.8328777907451536
XP_216945	1.1561208585603788
XP_216949	0.8019839642835818
XP_216955	0.8923592923491067
XP_216959	2.042248130903182
XP_216960	-1
XP_216962	1.0190436004806969
XP_216963	0.8449206459592862
XP_216964	0.7502888076765037
XP_216965	-1
XP_216966	3.006688102006102
XP_216967	0.8344854503408277
XP_216968	0.7589678430893169
XP_216969	1.1078917055597772
XP_216970	-1
XP_216973	1.3767518384601436
XP_216979	-1
XP_216981	0.891070336182466
XP_216983	1.3488052569527582
XP_216985	1.144974853716504
XP_216989	1.1286332986440488
XP_216991	0.7858682401016357
XP_217013	0.8820681735183165
XP_217014	1.0711496143286012
XP_217016	1.0221461244305379
XP_217017	1.041939510095306
XP_217018	0.9272163662467428
XP_217019	1.0476666299401967
XP_217021	1.4013381691003586
XP_217022	0.8511924358901266
XP_217035	0.5774039565736951
XP_217036	1.2270025459683858
XP_217038	1.2471064115005108
XP_217041	0.7777048860608948
XP_217044	-1
XP_217046	1.1006855914039193
XP_217048	1.0722250853965107
XP_217050	0.9209490030509855
XP_217055	0.9812692551589267
XP_217061	0.8657154501443595
XP_217062	0.7805311711720689
XP_217063	1.151489465915161
XP_217078	-1
XP_217080	1.06376580655204
XP_217086	1.1226396101923488
XP_217089	1.4076168969224296
XP_217092	-1
XP_217093	-1

XP_217094	0.996236232740008
XP_217103	0.9685501105665499
XP_217105	1.1182861101712425
XP_217106	1.3132810562862496
XP_217113	0.7764037133988383
XP_217114	-1
XP_217115	1.0711049216874187
XP_217120	1.0347123392160515
XP_217121	-1
XP_217129	-1
XP_217130	0.8231522700377778
XP_217134	1.1470508207361172
XP_217136	1.127684456289526
XP_217138	-1
XP_217141	1.724778443630119
XP_217144	0.7338246142183872
XP_217146	0.7970380417083041
XP_217148	1.0316966249199002
XP_217149	1.0840454842627303
XP_217152	1.3868119380319845
XP_217155	0.8933902969001705
XP_217157	0.9842848808929906
XP_217161	1.0798054734395366
XP_217165	0.5301315071190523
XP_217167	-1
XP_217170	1.1606440828804019
XP_217177	-1
XP_217180	-1
XP_217181	0.8248305378214225
XP_217185	1.0003115955253297
XP_217189	1.1225657488131624
XP_217193	1.0848670625084598
XP_217194	0.40249104108198597
XP_217195	0.9102398616138907
XP_217197	1.4230608870966532
XP_217198	-1
XP_217200	-1
XP_217206	1.0605221202491906
XP_217208	0.8586657081213137
XP_217209	0.9848323302338867
XP_217210	1.1488116828278019
XP_217219	0.5679767636284544
XP_217224	1.0016928320502452
XP_217235	1.0368483181217574
XP_217239	0.9479358674323723
XP_217245	1.0884168022445362
XP_217246	0.7818739341503737
XP_217250	1.269086788918626
XP_217254	0.9720090947926782
XP_217258	0.8713834229501655
XP_217262	0.9975286402754335
XP_217263	0.9272933712293989
XP_217268	-1
XP_217275	1.120634401447959
XP_217279	0.978122874495781
XP_217283	0.8480387005139258
XP_217284	0.9522305584545312
XP_217285	0.9787052151510972
XP_217293	-1
XP_217294	0.9839684431288195
XP_217295	0.9270545171092773
XP_217296	1.5130414855075964

XP_217297	1.3353348137147618
XP_217300	1.0484313021214544
XP_217304	-1
XP_217313	0.8453615670686214
XP_217314	0.830998605550481
XP_217317	-1
XP_217318	0.9922860231365364
XP_217320	1.245507837437488
XP_217326	-1
XP_217334	0.9244940815968601
XP_217341	-1
XP_217342	-1
XP_217346	1.9680366986971716
XP_217350	0.6217459919308732
XP_217355	0.9321128302580686
XP_217359	1.075730958783741
XP_217363	1.4223459887240975
XP_217372	1.4108575645838712
XP_217373	1.0425628439055308
XP_217375	1.3462841972432775
XP_217378	0.9116955286314835
XP_217381	1.6201749773073413
XP_217393	1.1187479763380763
XP_217397	0.9921973965248415
XP_217400	0.7306265181540298
XP_217405	1.0038827971644408
XP_217409	0.8634728358383903
XP_217410	0.5942058061456338
XP_217414	1.0912745711484464
XP_217421	0.8925770343435896
XP_217431	0.8760853950084724
XP_217432	1.1401340585042326
XP_217436	0.9926813178008166
XP_217437	0.769688015894067
XP_217441	1.050214064039969
XP_217444	0.8239461913291972
XP_217445	0.7843761748153711
XP_217448	1.116669400247561
XP_217450	0.7802443004296388
XP_217454	1.0440917409805004
XP_217458	1.0158971257298222
XP_217464	1.2085956989534854
XP_217465	0.8109463793327962
XP_217470	0.6485700756049538
XP_217474	0.970001041660677
XP_217475	0.764697284887044
XP_217476	1.1899170694487238
XP_217492	0.9447969813351801
XP_217505	1.1269969128975743
XP_217514	1.152259857356081
XP_217519	1.6927799976802163
XP_217535	1.3896459495409366
XP_217550	0.8150369470756743
XP_217560	1.0056438510847625
XP_217563	0.7581086493200558
XP_217568	0.8885932376129756
XP_217570	1.0626518132303195
XP_217572	0.8373232284262769
XP_217585	1.2659043579362665
XP_217586	0.8490370079556532
XP_217587	0.7883071453874013
XP_217592	0.6459162344097357

XP_217594	1.0024744027740502
XP_217595	1.0681341802133533
XP_217596	0.8342849506131516
XP_217599	1.298296428540278
XP_217601	1.095970530618356
XP_217607	-1
XP_217612	-1
XP_217615	1.0680105490575313
XP_217616	1.8036411675179878
XP_217637	-1
XP_217641	1.2749947132229262
XP_217642	0.8590427297750917
XP_217647	-1
XP_217657	1.5981530950145624
XP_217663	1.3564426893630468
XP_217671	1.2928152213740753
XP_217680	0.9318838850854969
XP_217689	-1
XP_217693	1.0961077676869133
XP_217724	-1
XP_217725	-1
XP_217732	0.6273119736243381
XP_217737	1.0853247092862928
XP_217743	-1
XP_217744	-1
XP_217745	1.0512133784634632
XP_217785	1.155356320045626
XP_217791	0.8319477608708478
XP_217792	1.7744465227199104
XP_217804	1.0330162243874208
XP_217806	1.7214853575868077
XP_217807	1.8344035446311526
XP_217819	0.6609260813400503
XP_217825	-1
XP_217827	1.0862024503698033
XP_217838	-1
XP_217848	-1
XP_217910	0.9432005139449599
XP_217944	1.0050239573724424
XP_217963	1.2109056156521059
XP_217975	1.018129823459649
XP_218002	0.9776851314506664
XP_218008	1.2879979628242297
XP_218012	0.8718171998924417
XP_218037	0.9683414630168783
XP_218041	1.269699647667999
XP_218069	1.13194879762107
XP_218070	1.1760266017467926
XP_218081	1.213783273523547
XP_218084	0.620559522154249
XP_218085	1.0094299269130784
XP_218094	0.9844030107202549
XP_218114	1.7524811489775953
XP_218124	1.4962368740881906
XP_218128	1.138326615085902
XP_218148	1.2655351383068492
XP_218157	0.7991352892481194
XP_218158	1.0106190500342664
XP_218162	1.0100931758072622
XP_218181	0.44937875273604133
XP_218185	1.179852811862245
XP_218187	1.0954768431933855

XP_218198	1.1951563442859612
XP_218199	1.156219108026196
XP_218202	-1
XP_218203	0.77545323568834
XP_218204	0.7340766060154773
XP_218209	0.64792806219068
XP_218215	1.2395123856844663
XP_218220	-1
XP_218221	1.1980740004223642
XP_218223	0.37388515756490537
XP_218226	1.1085567029213996
XP_218227	1.1588755319488453
XP_218238	0.9549893375784976
XP_218248	0.7008189301602504
XP_218274	0.8117297882259447
XP_218275	-1
XP_218287	1.054061018636563
XP_218292	1.908135032325194
XP_218336	1.1257996443283416
XP_218337	1.1338389330711676
XP_218342	1.1802479147021112
XP_218343	0.8380267894217244
XP_218345	-1
XP_218346	1.512002698424011
XP_218355	1.044329642864841
XP_218358	0.8652616798851517
XP_218364	1.2024544676100652
XP_218368	0.9626564172279259
XP_218378	0.859634029137809
XP_218382	1.5787990780710999
XP_218384	-1
XP_218395	1.355336837651904
XP_218398	-1
XP_218405	1.1053158622223604
XP_218412	0.9850056956365325
XP_218414	1.0175065510517167
XP_218415	-1
XP_218421	1.6503483671098056
XP_218425	0.9500613194270828
XP_218426	0.8711887902490963
XP_218427	0.5735955493144714
XP_218444	1.07766879238053
XP_218446	-1
XP_218447	1.1078099937400372
XP_218452	0.6802927260697995
XP_218456	1.4627083981748508
XP_218459	1.1753584695062245
XP_218461	0.9894846061286328
XP_218462	1.4732782739664076
XP_218473	1.522014846933648
XP_218479	0.8927377985621023
XP_218483	0.9007965303928843
XP_218487	0.9911889236786838
XP_218502	1.1424187734122675
XP_218522	1.1407390038451242
XP_218546	0.780334002913909
XP_218549	1.2698907108844788
XP_218558	1.0382509002699485
XP_218577	1.2749499314453852
XP_218589	0.6319937895614401
XP_218592	-1
XP_218594	-1

XP_218595	1.3068132786509778
XP_218598	1.249756322483827
XP_218608	1.148483663780045
XP_218609	0.8905048075222792
XP_218615	-1
XP_218619	1.2214625457541064
XP_218620	1.3699676434735608
XP_218625	0.7200889985436069
XP_218630	1.0702002559361745
XP_218633	1.293907953955846
XP_218638	1.363052680425463
XP_218639	1.0246360045929368
XP_218648	-1
XP_218652	0.9605196100548904
XP_218663	0.9246823994399083
XP_218665	1.0781213888989412
XP_218667	3.392728489555878
XP_218676	1.0592850374608347
XP_218703	1.1439431446550232
XP_218704	1.1751392892816361
XP_218706	1.7596956953585252
XP_218717	1.212005022732443
XP_218720	0.9670420581782938
XP_218734	0.8670747532393993
XP_218743	-1
XP_218753	1.350625126182672
XP_218755	0.9738486426883604
XP_218759	0.969194499402236
XP_218760	0.7876299371227731
XP_218762	1.07141045299066
XP_218776	1.16482481492366
XP_218780	1.0143015341589865
XP_218815	1.384633392791466
XP_218816	0.9513463403691748
XP_218817	1.3207535454003145
XP_218818	1.2252045045842213
XP_218819	1.0808163246727784
XP_218821	1.2513237333112903
XP_218824	1.2865182642397213
XP_218829	1.035377173015347
XP_218832	1.7432742416335618
XP_218833	1.0574451387932398
XP_218841	0.9526482242331611
XP_218845	1.290809632596612
XP_218852	-1
XP_218855	-1
XP_218859	-1
XP_218860	0.8766006783931416
XP_218861	-1
XP_218868	1.1636859573748397
XP_218897	1.135473370884345
XP_218915	1.2765078345308984
XP_218927	0.5654599317618074
XP_218929	0.8353309982841627
XP_218937	1.1482138175612275
XP_218949	1.188146661353334
XP_218953	1.257606981471778
XP_218958	1.1173857251969335
XP_218963	1.2657033328602363
XP_218964	0.9216246862081245
XP_218972	1.4281235264820464
XP_218975	-1

XP_218977	1.1724863871963773
XP_218980	1.471000199222967
XP_218995	0.8917313607186691
XP_218997	1.2614132793315698
XP_218999	-1
XP_219002	1.1843651805592357
XP_219006	1.0114052687951745
XP_219016	1.1343622799600324
XP_219039	1.164837035870473
XP_219062	1.1377378235771343
XP_219068	1.1961292218092636
XP_219070	1.0656062914439433
XP_219074	0.9899707771722536
XP_219081	0.8309183761763661
XP_219083	1.0124844835697207
XP_219088	1.3271017381430772
XP_219101	1.0279213147073234
XP_219126	1.1173472347594127
XP_219127	1.1707279021179693
XP_219128	0.8696284716606127
XP_219130	1.1024353800806488
XP_219135	0.9815751380600579
XP_219176	-1
XP_219183	-1
XP_219184	-1
XP_219194	1.19018436086342
XP_219202	1.1970370134786592
XP_219204	1.1195283935764864
XP_219208	1.048429953490668
XP_219221	-1
XP_219264	1.2244665586447185
XP_219265	0.5782125005037485
XP_219276	1.0444668782407327
XP_219279	-1
XP_219281	-1
XP_219290	-1
XP_219292	1.671904196386959
XP_219296	0.8582604858662765
XP_219297	1.087762552590578
XP_219312	1.2293774687700796
XP_219314	1.4341900559406489
XP_219318	0.7529088775832565
XP_219325	1.1707634602445005
XP_219336	1.1996649771273447
XP_219345	1.933687715291665
XP_219352	1.2234002935563935
XP_219354	1.0673197809428647
XP_219356	1.1429798895810634
XP_219359	0.9092693065347776
XP_219363	0.9278311028575311
XP_219364	1.012682634939162
XP_219365	0.8310910587765803
XP_219372	0.8816321396182275
XP_219373	0.8387142532284942
XP_219376	1.2351764505288259
XP_219386	1.4760038402640798
XP_219389	1.3242335188680472
XP_219392	1.6804178621689667
XP_219401	-1
XP_219414	1.0341528140107643
XP_219437	-1
XP_219441	0.9286367490335143

XP_219450	0.8760507890351956
XP_219454	-1
XP_219462	1.073508425373659
XP_219470	1.288368195738082
XP_219477	-1
XP_219490	-1
XP_219494	1.0276712345653733
XP_219498	0.9435421339193292
XP_219503	1.019305580339162
XP_219510	1.0298635664592384
XP_219511	1.3440261775762672
XP_219515	1.1652603000627761
XP_219517	-1
XP_219519	0.7956009153351661
XP_219525	1.1005154647615256
XP_219526	1.0109888264181854
XP_219529	0.7575479632440606
XP_219535	1.2247646164491677
XP_219540	1.9363646276194038
XP_219546	-1
XP_219554	1.1822107053532456
XP_219556	1.047912033008626
XP_219557	1.0433480318521065
XP_219563	0.847878792907651
XP_219564	0.7917100471322422
XP_219575	1.041458798105605
XP_219576	1.1367458976904319
XP_219579	1.22884936700566
XP_219584	1.2450313474662906
XP_219600	1.141359021504856
XP_219602	1.151803557688716
XP_219604	1.6135386155620826
XP_219609	-1
XP_219631	0.8020397672966576
XP_219651	1.404980585060381
XP_219677	-1
XP_219679	0.7204211894488151
XP_219683	0.9109361151431895
XP_219685	1.0375327393971532
XP_219692	-1
XP_219694	1.1176431186231222
XP_219703	-1
XP_219705	1.2198865532429963
XP_219708	1.7215116218034763
XP_219716	1.0277412480293684
XP_219720	-1
XP_219723	1.1388824908569548
XP_219728	-1
XP_219729	1.2026482203205484
XP_219736	1.2448989933997174
XP_219747	1.0036609659102886
XP_219749	-1
XP_219757	0.9998561215033422
XP_219766	1.0980304857908367
XP_219775	0.9859908710575589
XP_219777	-1
XP_219778	1.225566948846629
XP_219785	-1
XP_219793	-1
XP_219819	-1
XP_219820	0.6588452942303744
XP_219825	1.059402510520301

XP_219848	1.533913136072263
XP_219856	1.0611576877219442
XP_219860	0.893724913848399
XP_219866	0.909318896021208
XP_219870	1.215288009998236
XP_219873	0.6471120445722077
XP_219879	-1
XP_219880	1.4011678802027128
XP_219895	1.173746341906375
XP_219896	-1
XP_219902	-1
XP_219905	6.160905743454783
XP_219909	-1
XP_219915	-1
XP_219925	2.769977559347474
XP_219926	-1
XP_219938	1.0909165515210737
XP_219939	1.117360674096365
XP_219944	0.932596110522241
XP_219948	0.954682651032984
XP_219959	0.5366805530038915
XP_219962	1.6172778644035504
XP_219963	-1
XP_219967	1.6282257045932769
XP_219971	0.9044812853845078
XP_219976	0.7568639198910128
XP_219998	1.3282146450166725
XP_220013	2.611038402530941
XP_220019	1.4107564360830807
XP_220024	1.0986453930525937
XP_220031	1.4009684636194806
XP_220043	0.6170346762541314
XP_220044	0.903208263854026
XP_220051	1.3356794054474526
XP_220055	1.468796820834127
XP_220060	-1
XP_220071	1.7655177575322054
XP_220093	0.8717003437850442
XP_220097	1.666034562804288
XP_220099	0.9531476790420418
XP_220122	0.7403126590537412
XP_220152	0.9558057473347241
XP_220155	1.1164795329982358
XP_220159	1.1601759697666487
XP_220161	0.9378028712475158
XP_220167	1.2067403798163443
XP_220169	1.1743572654511152
XP_220180	1.3754261143116349
XP_220185	1.5665067328907094
XP_220197	1.0941671572025438
XP_220198	0.7648679793847166
XP_220199	-1
XP_220206	0.21544485828226864
XP_220207	1.6126184373491714
XP_220220	-1
XP_220221	1.0324212195262779
XP_220222	1.4111979006390634
XP_220229	0.965404809434912
XP_220230	1.09647968131438
XP_220231	0.9647074004512687
XP_220232	1.141693209396456
XP_220236	0.7918552723585407

XP_220240	1.0671827261075604
XP_220243	0.9473738754330127
XP_220256	0.5816956856287301
XP_220257	1.4623665851659509
XP_220262	1.044700800768986
XP_220263	-1
XP_220274	-1
XP_220286	0.9029329465688565
XP_220308	1.2321501216603523
XP_220335	0.9738049300341411
XP_220341	0.978010419018703
XP_220346	1.0951412912800298
XP_220357	1.048703456924684
XP_220360	1.0450954779680892
XP_220362	1.1933818294937837
XP_220368	-1
XP_220373	1.042537658248836
XP_220380	0.7096392966358295
XP_220398	0.9686689587559901
XP_220404	0.8256186562505232
XP_220413	1.0263658690966877
XP_220420	0.8908350388961012
XP_220428	1.049070084622681
XP_220442	1.0624011288706658
XP_220448	1.8856198588400872
XP_220451	1.2151838627999694
XP_220455	0.9318813659151359
XP_220456	1.3926359909944932
XP_220495	1.1241598187966915
XP_220498	-1
XP_220504	0.5713639285138246
XP_220508	1.0768685002420102
XP_220511	0.7865190233416833
XP_220513	1.1437508806581451
XP_220514	0.8273530635339962
XP_220520	1.0011313574412644
XP_220522	0.8966118945983906
XP_220530	0.8985143999027524
XP_220532	0.8201893859816927
XP_220535	2.8099074851333548
XP_220540	-1
XP_220541	1.0127395323576789
XP_220544	0.7428192039607849
XP_220549	1.2245194204364382
XP_220563	0.32186195912141563
XP_220573	-1
XP_220576	0.9696898134936144
XP_220582	0.9987922574268691
XP_220588	1.070277534977542
XP_220592	1.052265568242429
XP_220593	1.4974198967274717
XP_220595	-1
XP_220600	1.3869840416675865
XP_220602	1.5490382471370276
XP_220603	1.2158177015211231
XP_220606	0.9970899174590557
XP_220607	0.883968269775823
XP_220609	0.9304110600130668
XP_220612	1.6662236299694233
XP_220615	1.105218393798292
XP_220618	1.2412876980771836
XP_220624	1.0350405095844624

XP_220627	1.0361757951923802
XP_220632	0.8119056952865168
XP_220636	1.180762407365667
XP_220639	1.1230072799348794
XP_220640	0.6439650486136479
XP_220642	0.6027615758817894
XP_220644	1.1393721022974852
XP_220645	0.9316502228449152
XP_220647	1.087529082030459
XP_220660	1.0964944393740632
XP_220667	0.72710748997498
XP_220692	0.5576644548115847
XP_220694	1.4585236813362104
XP_220695	1.1618747970236492
XP_220698	-1
XP_220699	1.2418972030723505
XP_220700	0.9906623477416909
XP_220705	0.8543297218315736
XP_220706	0.9155574889337004
XP_220712	0.9390540533083741
XP_220717	-1
XP_220719	-1
XP_220725	0.4451630343643553
XP_220736	0.9696528583543991
XP_220750	1.1101680929902749
XP_220753	1.919137623496792
XP_220754	1.0877442286427212
XP_220756	2.2085946567806185
XP_220757	1.0453256661332164
XP_220766	0.9767097213890218
XP_220770	0.8657764255330621
XP_220771	1.996049152885148
XP_220776	0.9946483095187705
XP_220782	-1
XP_220783	0.9753658306483177
XP_220785	2.2115682321743892
XP_220798	1.3042377636712101
XP_220802	0.7319448145115284
XP_220804	1.1605766113884808
XP_220810	-1
XP_220813	1.3574948617420513
XP_220825	1.0505334542470481
XP_220829	0.7433545336673396
XP_220860	0.334082468420944
XP_220882	1.002404989585744
XP_220884	2.1635351099460425
XP_220894	0.9292346200055448
XP_220902	0.9578634063263962
XP_220907	0.9419557613432897
XP_220918	-1
XP_220919	1.040516142995773
XP_220928	1.1223444711559287
XP_220931	1.9523607251084056
XP_220938	1.6342444084542223
XP_220950	1.4683537330822594
XP_220957	1.116429568064867
XP_220964	1.2797013474690833
XP_220971	0.9520383953020182
XP_220978	0.848536401016089
XP_220981	0.9808375556390981
XP_220990	0.9648124365837892
XP_220993	1.4637018106871478

XP_220994	1.2109613661320189
XP_220996	1.057038892193877
XP_221002	2.075385437979227
XP_221004	-1
XP_221008	0.8366679800090829
XP_221015	1.012072570522333
XP_221021	1.247386906779302
XP_221022	0.7993928168064391
XP_221025	1.2641825146158099
XP_221030	1.3182844205528836
XP_221034	1.851231052109104
XP_221038	1.1036966839454263
XP_221047	0.8863972247807318
XP_221048	1.0013959138855744
XP_221050	1.1637026543468647
XP_221063	0.9991310184490808
XP_221083	-1
XP_221087	1.1984822552280192
XP_221091	1.0288934757839527
XP_221100	1.3510199727012326
XP_221102	2.5747865404330974
XP_221103	0.9029878139960673
XP_221108	0.9098301631851159
XP_221110	1.021383491588556
XP_221111	0.7778394885114376
XP_221119	1.1138640746613477
XP_221120	1.0273999626097645
XP_221124	1.0735894460036348
XP_221129	-1
XP_221136	1.1747697274731572
XP_221139	1.1926198867407751
XP_221141	1.2543931136754518
XP_221142	-1
XP_221143	1.7024194230017897
XP_221183	1.0154897340947424
XP_221186	1.0713604986630885
XP_221189	-1
XP_221191	1.0669267284515171
XP_221195	0.7003679559043241
XP_221202	0.7381752772125595
XP_221212	-1
XP_221214	1.0629833934702422
XP_221216	1.0443224841266414
XP_221217	1.0763862568048592
XP_221220	1.4373554549394838
XP_221245	1.3449853508719798
XP_221248	2.1929567225253943
XP_221249	1.3114000344942711
XP_221258	-1
XP_221261	1.0730580311478835
XP_221268	0.8877152944410929
XP_221270	0.7418607005530191
XP_221272	1.398501433007308
XP_221276	1.4514096758485022
XP_221290	1.036072233363166
XP_221302	1.3960855825919767
XP_221307	0.8792423262542155
XP_221309	0.8628505147341153
XP_221310	-1
XP_221319	0.8147441954895719
XP_221321	0.9140629252023906
XP_221337	-1

XP_221343	1.4469065520955644
XP_221345	-1
XP_221354	1.1223903723481745
XP_221357	0.6856604561352727
XP_221358	0.9750597838915219
XP_221374	0.9107267713487704
XP_221376	0.8411567205556404
XP_221379	-1
XP_221382	0.8957606601654998
XP_221384	1.1353452887827993
XP_221387	1.0832272470869933
XP_221392	0.822190189727115
XP_221396	0.8623081038483206
XP_221400	1.2313171238216618
XP_221404	0.8226354030338272
XP_221417	1.108305184901322
XP_221420	0.9134772949815758
XP_221424	-1
XP_221437	1.5017150704518638
XP_221438	0.9740226566196034
XP_221439	-1
XP_221441	0.9730815278694601
XP_221446	1.0345986499967732
XP_221452	-1
XP_221454	1.0239662421723856
XP_221455	0.99229656282052
XP_221464	0.9317160088118523
XP_221465	1.0053036017477797
XP_221479	0.9190377734724835
XP_221489	-1
XP_221497	1.3329704250447252
XP_221512	0.9818177374350094
XP_221521	1.2136356186194779
XP_221527	1.1043165528437704
XP_221535	1.1659556419585098
XP_221536	0.9942823315205582
XP_221539	1.0752917151850194
XP_221545	1.5332067764762203
XP_221560	0.6565637467697176
XP_221562	0.8485313126492331
XP_221566	-1
XP_221568	-1
XP_221595	0.6766344970066488
XP_221622	1.0108558027798078
XP_221627	0.9913189272578853
XP_221639	0.8306436346340575
XP_221640	0.9957523222729124
XP_221641	0.7666870117286885
XP_221655	1.1670152040420132
XP_221656	1.3257171578331042
XP_221670	4.202839995497324
XP_221671	1.4494488321740906
XP_221672	2.9515604572272967
XP_221690	-1
XP_221697	1.1444461810003608
XP_221712	0.9394018697792482
XP_221722	1.18823291316491
XP_221737	0.7886318458431115
XP_221768	0.5598943314437826
XP_221787	1.1147776533116203
XP_221788	1.1959486285364784
XP_221796	1.6399206267607138

XP_221799	1.3903150122770567
XP_221817	1.3098168977461813
XP_221867	-1
XP_221871	0.8771128319636009
XP_221896	1.258245525333953
XP_221910	1.2647084046766006
XP_221913	1.0314657953118382
XP_221915	1.0798345668050564
XP_221916	0.8596866971681716
XP_221920	0.8185208006614771
XP_221922	0.8925951636315929
XP_221925	1.124646377843686
XP_221926	-1
XP_221932	-1
XP_221936	1.2599378613482672
XP_221937	1.3158351618375823
XP_221941	1.2086304943146764
XP_221946	1.0597734158692584
XP_221949	0.869695581102491
XP_221954	0.8371938274077224
XP_221956	1.3415035014681314
XP_221957	0.9252157552289969
XP_221971	-1
XP_221972	0.7858641461031439
XP_221990	1.287848673671055
XP_221996	0.9696870727528627
XP_222000	0.8203469901140457
XP_222002	0.820582417544358
XP_222015	1.0028060010433293
XP_222019	1.026165399595234
XP_222024	1.0773526097161008
XP_222035	1.1110113762416627
XP_222046	1.2146976869592052
XP_222048	1.0860402817570967
XP_222057	0.8255977765862659
XP_222065	1.2533082647093357
XP_222084	1.0933764841775397
XP_222088	0.8755656025171855
XP_222105	0.8783311280823219
XP_222107	1.199382037181312
XP_222140	1.2088327296391235
XP_222145	1.207103041262017
XP_222150	-1
XP_222152	1.7545975605365738
XP_222155	1.170867115241099
XP_222157	1.813452383007322
XP_222159	0.9066497742066789
XP_222171	0.8932960015797852
XP_222178	1.6023727782796104
XP_222179	0.6066963893280481
XP_222180	1.0916025837629368
XP_222184	1.1856364255311387
XP_222187	-1
XP_222190	-1
XP_222198	1.104091582011695
XP_222212	6.125608151489009
XP_222227	0.9457833264982308
XP_222228	0.7081310757682974
XP_222231	7.985574814282482
XP_222242	0.9540588884130162
XP_222251	0.9169357298373613
XP_222252	0.8716846896939092

XP_222253	1.1765184760540093
XP_222254	1.1161507631328336
XP_222255	1.219715710612342
XP_222260	1.5256225060323683
XP_222263	1.1527065904365104
XP_222267	0.9880207795440548
XP_222273	0.6905170054942161
XP_222276	-1
XP_222287	0.8853700776975275
XP_222292	-1
XP_222302	-1
XP_222331	1.1753424561093908
XP_222335	-1
XP_222339	0.9989093580771997
XP_222370	1.573809615223377
XP_222377	-1
XP_222382	0.966021760964033
XP_222384	-1
XP_222392	-1
XP_222421	1.1139458181805595
XP_222428	-1
XP_222442	1.082120930390806
XP_222452	1.1069678274323564
XP_222453	1.0504503025214265
XP_222456	0.5704544950394439
XP_222457	1.2015005566463202
XP_222460	0.9994789622650688
XP_222461	1.3151377329125735
XP_222476	0.8579129302002659
XP_222499	1.104173032635786
XP_222503	0.7686321804212315
XP_222520	1.10425740224504
XP_222534	-1
XP_222538	1.0346114551748247
XP_222551	0.7955345248208879
XP_222557	1.212451497504075
XP_222559	0.9378944880110258
XP_222561	1.1395998487856878
XP_222564	-1
XP_222583	1.0914430005321365
XP_222597	1.2022475131610764
XP_222609	1.274978818647738
XP_222613	1.0126777109936986
XP_222618	-1
XP_222624	1.1253450055005911
XP_222627	0.939785261521607
XP_222637	0.929093138245565
XP_222662	0.8963747927140927
XP_222667	1.388739195748153
XP_222675	1.1926465278051077
XP_222678	1.0722525000700251
XP_222686	0.8571652379110039
XP_222693	1.0121501936791844
XP_222695	0.9904844524101947
XP_222716	1.2433060833396228
XP_222745	1.4442246123633167
XP_222768	1.499037943094353
XP_222769	0.8548071442055485
XP_222780	0.6333537662316816
XP_222801	0.7608653300490724
XP_222804	1.045574798782107
XP_222824	1.1754209819027561

XP_222828	1.4226623115091506
XP_222831	1.1016166280938584
XP_222832	1.232153976197594
XP_222834	1.1268559180702045
XP_222837	-1
XP_222849	0.9282166645883225
XP_222855	1.1016515158493136
XP_222863	3.3889601713533684
XP_222868	1.133009430553927
XP_222887	1.0656280471646107
XP_222897	1.3344874805741824
XP_222899	0.8144438051369772
XP_222900	0.7816184404662025
XP_222902	1.1021607460750493
XP_222913	0.9329902329925069
XP_222918	1.3095046503208783
XP_222920	1.101040869878864
XP_222946	1.2297970287274302
XP_222952	0.7767169297635425
XP_222955	3.648886247535365
XP_222958	-1
XP_222971	-1
XP_222975	1.0807303012951832
XP_222991	1.1236366124219488
XP_222995	1.2910673290848098
XP_223012	1.1430425803420081
XP_223014	1.3065489682728848
XP_223016	-1
XP_223022	1.0103713618117247
XP_223053	0.8561049358919504
XP_223054	1.240660475370286
XP_223057	1.1875796326549972
XP_223060	0.7865171103975365
XP_223074	1.4304404483824211
XP_223075	1.3817160436243647
XP_223076	0.14315777882862907
XP_223083	1.349138374761811
XP_223085	0.8043126735315839
XP_223087	-1
XP_223090	1.3338481970576002
XP_223116	1.01239966742583
XP_223121	-1
XP_223124	1.2078165250909685
XP_223143	1.1582755995566218
XP_223146	0.5782373429959996
XP_223148	0.7940503249130458
XP_223150	1.604268912164549
XP_223152	1.1939816824021068
XP_223159	-1
XP_223174	0.9802966089041499
XP_223180	1.1335694918955028
XP_223190	1.39795276898184
XP_223196	-1
XP_223205	1.1067999952083962
XP_223209	-1
XP_223229	0.9747363219209746
XP_223235	1.0009589640977488
XP_223237	1.1589901953053812
XP_223279	1.168579413905847
XP_223290	0.9366750334206703
XP_223301	1.2978918177820458
XP_223305	1.1798511319420557

XP_223310	1.0458406630832426
XP_223335	1.248213961938333
XP_223338	-1
XP_223340	1.6593090998630349
XP_223341	0.6370349850617019
XP_223350	1.2228061958611058
XP_223353	0.9898630172574647
XP_223365	1.2388758310773853
XP_223378	-1
XP_223394	1.112426139524873
XP_223397	0.8860464225073442
XP_223399	0.6260588393972857
XP_223400	1.0849025440957785
XP_223406	1.0841622210359125
XP_223410	0.9275329638426184
XP_223426	1.1095376561942558
XP_223434	1.3802982508317851
XP_223454	1.1596713952817514
XP_223463	1.2621621441010193
XP_223469	1.1202264713123355
XP_223485	-1
XP_223486	0.9893954597968335
XP_223494	1.3072639485841722
XP_223495	1.3717125198361069
XP_223496	1.0491973470598541
XP_223508	0.9105335047797581
XP_223511	1.2520341430083977
XP_223512	0.8535270630215859
XP_223515	-1
XP_223532	-1
XP_223533	-1
XP_223536	1.250804114095094
XP_223539	0.9133846551409314
XP_223541	0.7453111978680785
XP_223544	1.1518991414106592
XP_223556	-1
XP_223560	4.331785548555712
XP_223566	0.9457554064507642
XP_223580	1.0418669451747844
XP_223583	3.7201906762429267
XP_223590	1.0241090951419742
XP_223591	0.7920826934193093
XP_223592	-1
XP_223597	2.214171496347897
XP_223600	0.978745277812551
XP_223611	1.3343317974795714
XP_223613	1.2342098753607076
XP_223625	1.1218173020379676
XP_223640	0.9839953621521366
XP_223643	1.5013391859328813
XP_223674	-1
XP_223680	0.9979996619100981
XP_223684	1.0701333402795334
XP_223688	1.0170560585522843
XP_223710	-1
XP_223723	0.8598626210690395
XP_223725	-1
XP_223745	1.3647011780605338
XP_223768	1.225402623273867
XP_223775	0.7754355396276451
XP_223779	0.887617794631996
XP_223785	0.7288036362267571

XP_223786	0.8239096023543581
XP_223791	-1
XP_223792	-1
XP_223793	0.8151430653245265
XP_223816	1.1031954333281198
XP_223820	-1
XP_223826	-1
XP_223828	1.0095667117672134
XP_223876	1.119278788089206
XP_223879	1.1249718360981762
XP_223897	-1
XP_223902	1.255049795392343
XP_223905	0.8368730521298009
XP_223916	1.0581909157882725
XP_223918	1.1942573461708241
XP_223930	1.2105816985721123
XP_223937	1.164204205211006
XP_223938	1.045393187489991
XP_223969	-1
XP_223974	-1
XP_223975	0.9614741131278216
XP_223980	1.0528778552872549
XP_223981	1.0917071799826858
XP_223984	1.2439907037672082
XP_223991	1.345891060664709
XP_224000	1.4696370567071373
XP_224011	1.9436691022199313
XP_224014	1.1255262710674667
XP_224023	1.2320163704986142
XP_224084	0.7647757313834501
XP_224103	1.5363574276689658
XP_224131	0.78789623970233
XP_224143	-1
XP_224165	0.9291678684815202
XP_224172	1.2548755776832183
XP_224173	1.8698456404908264
XP_224181	1.0501679546066438
XP_224184	1.0717786679723627
XP_224186	-1
XP_224190	0.7822613630684382
XP_224197	1.4982565544543154
XP_224209	1.1471718779002786
XP_224231	1.1745564056173265
XP_224248	0.9925281766449018
XP_224256	1.4940089179784517
XP_224270	0.7194682545466584
XP_224282	0.6947477899889107
XP_224283	1.0055419285306026
XP_224288	1.6598050956134096
XP_224295	0.7296513211190107
XP_224297	-1
XP_224308	1.499727244516409
XP_224325	1.1536018640611114
XP_224334	-1
XP_224344	0.7945767379764112
XP_224348	1.0957683936176794
XP_224354	0.8677797021187422
XP_224366	1.425952571092228
XP_224400	1.5697902410843243
XP_224409	0.950666721946872
XP_224417	0.8337984825588628
XP_224429	0.9370707948042707

XP_224440	0.9820470020535362
XP_224449	1.3900425936700938
XP_224465	0.9375695140183365
XP_224478	1.1161352896151722
XP_224480	1.7141960740518998
XP_224489	1.0071255081228003
XP_224515	0.8138887216953545
XP_224518	0.3135796118457467
XP_224519	1.1505240072847973
XP_224521	1.128337032209809
XP_224525	2.1946221636567786
XP_224534	1.2211014767428574
XP_224535	1.344972535677838
XP_224538	1.0753820684815458
XP_224551	0.914693121954001
XP_224561	1.052085670721273
XP_224583	12.832548479358676
XP_224584	1.2500524162332098
XP_224604	1.3356877082375787
XP_224614	0.8342754745004415
XP_224618	1.039556939024638
XP_224620	0.874399695327082
XP_224627	-1
XP_224630	0.8030053359894155
XP_224637	0.800237540556696
XP_224639	0.8834918013809001
XP_224643	0.8766754824622446
XP_224646	-1
XP_224672	1.0161938790164862
XP_224673	-1
XP_224680	0.9000006236528448
XP_224706	1.0710403786446616
XP_224707	1.0971681408888412
XP_224708	1.206667583824562
XP_224712	-1
XP_224713	-1
XP_224715	1.2504889768825225
XP_224718	0.8816381113153504
XP_224722	0.8795774411217728
XP_224724	1.345252057859564
XP_224728	0.9264929908212314
XP_224729	1.1661314654049264
XP_224733	0.9750285468199351
XP_224739	-1
XP_224741	1.1399190759656672
XP_224762	0.9560490677664326
XP_224778	1.2626905021793668
XP_224781	1.1394271227658173
XP_224783	1.2053624630276791
XP_224784	0.7528285682588861
XP_224814	1.3059054879498202
XP_224826	1.3865736859193047
XP_224839	1.3634418688298955
XP_224847	0.9744295071027355
XP_224853	0.9897629845303076
XP_224859	1.026166684811684
XP_224861	0.8302585810465367
XP_224872	-1
XP_224892	1.0196634311362134
XP_224894	1.0663349606353982
XP_224908	-1
XP_224910	1.2022432475319762

XP_224923	0.9022293166737736
XP_224926	1.2794399309738291
XP_224929	0.8527706522473113
XP_224930	0.8494213206888416
XP_224934	1.468079306240928
XP_224940	1.109371585895061
XP_224944	1.1943628597618476
XP_224948	1.036519929840132
XP_224952	1.267944180747141
XP_224954	1.0197405393923844
XP_224961	3.848901386751166
XP_224964	1.3866594491825435
XP_224979	-1
XP_225006	0.9663491487108652
XP_225008	1.0357541333727676
XP_225020	2.8684426546631054
XP_225027	1.058409020199477
XP_225039	1.4124678796732575
XP_225043	0.6972880500583583
XP_225044	1.3990777883772907
XP_225052	0.8489580297990303
XP_225063	0.9344840706577046
XP_225068	-1
XP_225077	1.1596013824413713
XP_225078	1.106144830757991
XP_225086	0.8023888589739206
XP_225101	-1
XP_225104	-1
XP_225111	0.925832746925301
XP_225125	0.8916354161666762
XP_225137	2.023292096736752
XP_225138	0.8427651045943197
XP_225142	1.3331097409887889
XP_225143	1.448879479939141
XP_225147	2.4514818822072124
XP_225150	1.215045105095579
XP_225159	0.9474027100770365
XP_225168	1.0672934191144279
XP_225169	-1
XP_225170	1.212441218121146
XP_225176	1.008342183943077
XP_225178	0.5715355727890459
XP_225188	-1
XP_225198	0.11854113368568757
XP_225203	1.2731808255997377
XP_225204	1.0358624860086687
XP_225206	-1
XP_225211	-1
XP_225220	1.2619217401518794
XP_225227	1.1876792786874009
XP_225229	1.0994878672007404
XP_225233	0.8100475397378727
XP_225238	1.2647267977903722
XP_225241	1.2401025605640135
XP_225250	-1
XP_225253	0.9730256191083131
XP_225259	1.0911695631856244
XP_225307	-1
XP_225319	0.6228381215536893
XP_225322	1.2040111393395165
XP_225332	0.7772353016638358
XP_225336	1.16823605477699

XP_225342	0.5128488680651685
XP_225346	0.9212249932552906
XP_225368	1.2501894907882787
XP_225376	0.8462552376753503
XP_225379	1.7986820235478083
XP_225396	0.9097978648581152
XP_225404	0.8549942435843665
XP_225411	1.0629872086867687
XP_225436	1.0690555733808516
XP_225447	0.9595329442498869
XP_225457	2.08141330342928
XP_225472	1.222808163191717
XP_225491	1.3005428289325822
XP_225512	1.0038116277473945
XP_225526	1.1707709818143133
XP_225538	1.219199107265673
XP_225556	-1
XP_225559	-1
XP_225570	0.933569959179387
XP_225585	1.4538190700659879
XP_225586	0.9151335138556645
XP_225599	0.9879564788464744
XP_225600	1.1219535123916329
XP_225603	1.0195837374380805
XP_225610	1.2276533947540513
XP_225625	0.78770834356465
XP_225630	-1
XP_225631	1.1289714526810768
XP_225635	1.0466931330250475
XP_225644	-1
XP_225658	-1
XP_225659	1.3954030999966687
XP_225667	0.9202458455744049
XP_225679	0.9264117703932868
XP_225680	1.2246575413247096
XP_225685	0.7707742657568049
XP_225695	0.9101219562704794
XP_225698	-1
XP_225706	1.1002289954865074
XP_225707	1.3592307648176993
XP_225711	0.830980111468964
XP_225713	2.3680883259658905
XP_225717	1.0667692091034833
XP_225718	-1
XP_225730	0.8098439628295995
XP_225732	0.9983531058707105
XP_225735	-1
XP_225744	1.3276292506035237
XP_225748	0.912589809819071
XP_225755	1.2886132523412772
XP_225760	1.1634798244067037
XP_225768	1.1694228047662667
XP_225770	1.201619088808284
XP_225838	1.3322382192509035
XP_225856	-1
XP_225862	1.1973813221268177
XP_225867	0.6033087681316069
XP_225882	-1
XP_225885	0.9854382283933548
XP_225897	1.4042567635270093
XP_225909	0.5012141597279579
XP_225923	1.1015315819690905

XP_225966	-1
XP_225972	0.9245451435339733
XP_225981	0.9008481399680814
XP_225997	0.9911690747782417
XP_225999	0.6810826593531193
XP_226005	1.002089649691366
XP_226007	0.8716951259468594
XP_226008	1.1314269090518387
XP_226009	0.9653155919006878
XP_226014	1.2531246251267705
XP_226016	1.594883666179511
XP_226027	0.9086565808910567
XP_226032	1.200965084959696
XP_226046	1.535699393798773
XP_226056	2.2569648983966424
XP_226060	1.4908023966982569
XP_226070	1.0953202885893403
XP_226071	1.0825044793626004
XP_226098	0.7892132104721193
XP_226107	1.0205562650122697
XP_226109	-1
XP_226112	1.229602520657382
XP_226118	1.2936300934117329
XP_226119	1.252132045911136
XP_226121	1.363073390849217
XP_226123	-1
XP_226158	0.8131884924073645
XP_226159	0.7675781189300351
XP_226162	0.7382120012509988
XP_226165	-1
XP_226175	1.1096308191542814
XP_226178	-1
XP_226203	-1
XP_226209	1.415899055815393
XP_226210	0.7901689278896619
XP_226211	-1
XP_226233	0.9619607026974053
XP_226237	1.0257773898067726
XP_226238	0.8132827211931898
XP_226315	1.059051744939194
XP_226329	0.8567911571192817
XP_226330	0.938669456689599
XP_226331	1.0197186279564168
XP_226343	1.0545199731474153
XP_226349	1.0638756022613975
XP_226362	1.0407929540272363
XP_226369	1.1846209196255377
XP_226380	0.8558061391456984
XP_226381	1.0043093402996648
XP_226388	1.431630737987924
XP_226390	1.5969676930312051
XP_226402	1.4214337512491557
XP_226408	1.103712572400215
XP_226416	1.213221910311539
XP_226417	-1
XP_226449	0.883972696565932
XP_226462	-1
XP_226464	1.0101444506196016
XP_226489	1.2657782512542466
XP_226493	0.9148952939652473
XP_226517	1.0135474603718435
XP_226521	2.799251876145237

XP_226522	-1
XP_226528	0.7798230815540375
XP_226533	0.9503273098639856
XP_226544	1.0471311684060625
XP_226545	1.2116781323463663
XP_226546	1.1857349024753008
XP_226547	1.1012745703301972
XP_226548	1.2034008633250548
XP_226553	1.9839350802385087
XP_226569	0.9459896341892722
XP_226570	1.109110595572726
XP_226571	0.7663311181113314
XP_226572	2.1662154093593564
XP_226578	1.0372167974226238
XP_226582	0.9095226546144385
XP_226604	0.9918888445030557
XP_226606	1.2484690593733458
XP_226624	1.1293251803371327
XP_226639	1.1301899039133156
XP_226652	1.2500936493522128
XP_226666	1.1224830212732788
XP_226679	1.3297698590188889
XP_226696	1.1035140391339768
XP_226700	1.2349507336768177
XP_226703	1.2721312295289156
XP_226715	0.8075497557908715
XP_226716	0.9957283022057256
XP_226718	1.6191609968404777
XP_226722	1.035517607613003
XP_226731	1.0971490320205557
XP_226732	1.1538533617491271
XP_226733	-1
XP_226738	1.094888996248934
XP_226759	1.9070540139749692
XP_226768	0.9163700291193503
XP_226769	0.9716157278102652
XP_226771	1.0892101895760542
XP_226789	1.0030287823285604
XP_226790	1.203891958066743
XP_226798	0.715001779527865
XP_226803	-1
XP_226804	1.738868120356411
XP_226812	1.2629192754646867
XP_226813	1.1780414636771839
XP_226833	-1
XP_226834	1.1467931389557682
XP_226835	0.8425737519670818
XP_226837	-1
XP_226839	1.2106581398319711
XP_226842	0.8529824980768823
XP_226843	1.1777592763198463
XP_226853	1.3094623748654024
XP_226858	0.9946194452725575
XP_226874	1.133945191638734
XP_226888	0.8228338444017855
XP_226897	0.9666455373588888
XP_226909	0.9508419356412499
XP_226922	-1
XP_226939	0.8384229644354767
XP_226945	1.288350360779494
XP_226952	0.9951924534281122
XP_226953	-1

XP_226957	0.9790710836250297
XP_226964	0.7997426734868909
XP_226973	1.1177282872646186
XP_226976	0.9972235436180955
XP_226980	1.2344483243511932
XP_226987	1.2425961976662347
XP_226988	1.0031775965930056
XP_227009	0.9287746753800868
XP_227021	1.1627093138610387
XP_227030	1.1896914044126055
XP_227050	-1
XP_227060	1.0433917179036623
XP_227064	0.9117350751690486
XP_227066	1.2831047065137144
XP_227072	1.0906473253610207
XP_227074	0.9455376041633952
XP_227080	0.6533780163955535
XP_227081	1.9328474474340263
XP_227084	1.1424211052471676
XP_227088	-1
XP_227089	0.9972860658378444
XP_227117	-1
XP_227131	2.0600531846404304
XP_227132	1.0091520694823544
XP_227134	1.0626483964659348
XP_227140	1.134826854651146
XP_227142	0.096099364833102
XP_227148	1.408318236205828
XP_227155	1.211551715331539
XP_227163	1.3266941925457116
XP_227168	0.5871295725172633
XP_227177	1.193932719512955
XP_227201	2.2418715538133993
XP_227208	0.9653787032642508
XP_227232	1.05632950224092
XP_227234	1.063051404688125
XP_227247	0.8630663831670113
XP_227248	0.7089666896063566
XP_227282	0.9689657601411126
XP_227287	1.0057012300971107
XP_227313	1.1689274064939248
XP_227339	1.7063713520531254
XP_227351	0.8860557265851775
XP_227367	0.896481762676812
XP_227369	0.8030601701734902
XP_227371	1.0655265711928739
XP_227385	0.8833360236215687
XP_227389	1.209247339839077
XP_227396	0.6673239387438774
XP_227399	1.951491834193319
XP_227404	-1
XP_227405	1.179296090704989
XP_227412	1.2950282233368693
XP_227413	0.9475963256507355
XP_227420	-1
XP_227427	0.9997751921768518
XP_227428	1.042271244952793
XP_227429	1.0404601861825131
XP_227440	1.0538715318984297
XP_227443	1.3800817834804988
XP_227446	1.148424565278974
XP_227448	1.0320672474413692

XP_227451	4.768952761840539
XP_227462	-1
XP_227469	0.959161341720345
XP_227480	1.1157194075240675
XP_227483	1.1929417933348616
XP_227484	1.0167115783371206
XP_227489	-1
XP_227490	0.7834131656533584
XP_227491	-1
XP_227510	1.0416958822452436
XP_227516	1.1467983832923907
XP_227525	1.6589913971704926
XP_227535	0.4028671462717524
XP_227538	1.2154441242273941
XP_227540	-1
XP_227543	1.8573801055775523
XP_227547	1.167017373068093
XP_227549	0.9262744318657196
XP_227571	0.9068488226671733
XP_227577	-1
XP_227581	1.1139059126237971
XP_227583	0.9254819476246916
XP_227584	-1
XP_227591	0.545309009047576
XP_227598	1.026968699953161
XP_227605	1.0896089994573923
XP_227620	1.370550502401249
XP_227623	-1
XP_227624	1.3498957953225987
XP_227651	1.3578952799789026
XP_227657	1.5475784985992673
XP_227663	1.1735739043933773
XP_227665	1.1659440970976984
XP_227679	1.0424107760314314
XP_227686	0.8687175497024182
XP_227690	0.848129834118004
XP_227694	1.1958271223671464
XP_227701	1.2266935133630044
XP_227735	1.2312045402789893
XP_227745	1.3457961921826171
XP_227762	-1
XP_227765	0.905321967705791
XP_227786	1.3106699936674049
XP_227795	-1
XP_227828	2.5660666278978606
XP_227870	0.9284242804184782
XP_227873	1.4118548092090577
XP_227877	0.8894172321534898
XP_227882	1.037507672883313
XP_227896	-1
XP_227907	1.0302597142703975
XP_227921	1.1950526693657189
XP_227929	-1
XP_227945	0.9866242416445893
XP_227971	1.448454617853912
XP_227978	0.956571163536156
XP_227983	1.100806759547865
XP_227989	-1
XP_227992	1.228846246479837
XP_228005	0.8287340723331306
XP_228014	1.2620326665924886
XP_228016	1.3848772198582069

XP_228029	1.215872407453309
XP_228030	1.1203133582545555
XP_228032	1.2940569950953311
XP_228038	0.8815342778203187
XP_228043	0.8360878939029713
XP_228046	0.9055943892024093
XP_228059	0.5622731820478813
XP_228063	0.9737990136003961
XP_228068	-1
XP_228072	0.8678115629733125
XP_228073	0.7255487701676716
XP_228082	0.9973642978484072
XP_228091	2.667574835395521
XP_228122	1.0624146698487351
XP_228137	-1
XP_228147	1.1834657506243744
XP_228149	1.6656168862181735
XP_228158	1.1700784361870225
XP_228159	1.0664576546381868
XP_228164	0.7633951455279228
XP_228196	1.4066602653903668
XP_228197	1.5524452300208098
XP_228202	2.3014412114516714
XP_228209	6.409673489536046
XP_228242	1.8045135310823497
XP_228273	1.4463892004410492
XP_228301	1.045368160852754
XP_228305	1.022817949082385
XP_228320	0.42438561793859136
XP_228329	0.6292257678499271
XP_228360	0.35065563193209565
XP_228363	0.9593161208453495
XP_228392	1.2175016942496282
XP_228394	-1
XP_228424	2.1362558191731456
XP_228426	1.0432343989214183
XP_228432	-1
XP_228441	1.008055520541048
XP_228444	0.2803730145603655
XP_228447	0.7775440828638128
XP_228448	0.9875432099176153
XP_228455	1.0972495418361885
XP_228457	1.1143548431490224
XP_228475	1.0331148927498548
XP_228482	-1
XP_228493	0.9707330655108751
XP_228499	0.9349497951094817
XP_228500	1.014086614177573
XP_228501	1.314236819681731
XP_228518	0.12472240412611264
XP_228520	0.9423721036758836
XP_228522	1.125329405151516
XP_228536	0.9803762043016677
XP_228540	0.611330239943479
XP_228541	0.792525444456918
XP_228548	1.0060765214622687
XP_228551	-1
XP_228553	-1
XP_228556	1.086323335239305
XP_228590	-1
XP_228607	1.1973609928092561
XP_228615	0.9232221192381517

XP_228623	1.2159199598321158
XP_228644	1.1877340211102958
XP_228661	1.177695247757436
XP_228670	-1
XP_228683	0.8758346293317003
XP_228696	1.2355742134958743
XP_228708	-1
XP_228733	0.7006500605870252
XP_228753	0.4657029382380501
XP_228758	0.848357391924064
XP_228759	0.7153294143158115
XP_228760	1.1396479941458215
XP_228761	1.019798840247007
XP_228769	0.9497756073211033
XP_228770	1.1246027348098089
XP_228771	1.1658566253657627
XP_228776	1.165787358289476
XP_228777	0.9731670269818871
XP_228778	2.1042246628350814
XP_228781	0.7077010312806776
XP_228786	1.284609372100319
XP_228797	1.1867137950358715
XP_228801	0.7101712619741395
XP_228809	0.7317897605362891
XP_228810	0.9483818965623358
XP_228818	0.9783275883168674
XP_228841	-1
XP_228848	0.8713143992611379
XP_228858	0.9249781940603987
XP_228861	0.7994637757993175
XP_228862	1.1001318443374368
XP_228865	0.9345928401432273
XP_228867	-1
XP_228876	1.17928681961268
XP_228906	0.9103089421613259
XP_228909	1.0516902858004864
XP_228914	1.3348933123423545
XP_228942	1.0979411738134024
XP_228943	0.8480875572293348
XP_228973	1.0895001135325415
XP_229025	0.7683766274830249
XP_229026	-1
XP_229030	1.0159158835555444
XP_229036	1.0593320127158692
XP_229038	1.1771079946428211
XP_229065	0.8045439536217939
XP_229087	0.821444344863
XP_229096	1.0076312247555534
XP_229106	0.8318138466055052
XP_229110	1.0170638384795683
XP_229114	0.48273816367882394
XP_229122	1.8391889706152744
XP_229131	-1
XP_229140	1.3248316503080315
XP_229146	1.7657548415058142
XP_229152	0.9457147467806298
XP_229162	2.789825409635617
XP_229173	0.9991007677671208
XP_229192	-1
XP_229201	0.7408866928578698
XP_229225	1.4714476467189486
XP_229227	0.3462117581809225

XP_229231	1.1177965975884538
XP_229267	1.167657933418062
XP_229276	1.232571141506736
XP_229336	0.9801089493279899
XP_229338	1.1131538966636034
XP_229347	-1
XP_229361	0.6943323320128937
XP_229383	-1
XP_229399	1.5000892649388882
XP_229475	0.8599052866313557
XP_229481	1.1858204183618128
XP_229490	0.8427155480786533
XP_229548	1.3455649905798148
XP_229759	1.4231537326303592
XP_229770	-1
XP_229918	0.9805176444823558
XP_229925	0.5230615331495683
XP_229935	-1
XP_229936	1.4897325097261989
XP_229944	1.3724834036485731
XP_229959	-1
XP_229977	0.9622741777384561
XP_229979	1.3470186402821118
XP_229981	-1
XP_229988	-1
XP_230003	1.287314358076656
XP_230007	1.4129443175742304
XP_230015	0.8276622138455724
XP_230029	-1
XP_230033	0.5415058552135853
XP_230038	1.439447133082551
XP_230039	0.8630130722361575
XP_230047	0.8004093046229913
XP_230055	0.7203950920057548
XP_230070	0.8001009680974059
XP_230077	1.241729958423709
XP_230084	0.9671131252799446
XP_230093	1.365038713447215
XP_230118	1.2168838283355536
XP_230140	1.1228004724424745
XP_230146	1.727582156222131
XP_230169	0.9917949871933617
XP_230183	1.1405257103116775
XP_230213	1.1907225529329994
XP_230214	1.4667855061004176
XP_230225	1.3262785296018598
XP_230226	0.6363652315519971
XP_230228	0.7187529180250433
XP_230248	-1
XP_230269	2.1229182841055043
XP_230275	1.5596107479354575
XP_230276	1.221860931277605
XP_230278	1.337398629272096
XP_230279	1.1160692887110517
XP_230282	0.9666725027099465
XP_230283	-1
XP_230284	1.1263631617224905
XP_230297	-1
XP_230303	0.5998407900391864
XP_230306	-1
XP_230307	1.1218669745514847
XP_230327	1.067601002094605

XP_230346	1.4121072678414974
XP_230348	1.0311310452340587
XP_230356	0.9001397808813213
XP_230377	-1
XP_230389	0.9643078178936378
XP_230399	0.8577399664191617
XP_230402	1.09704324386951
XP_230457	0.9479552955501948
XP_230462	0.9169777035294695
XP_230464	-1
XP_230465	0.8666923539659364
XP_230467	1.0504758384946054
XP_230468	1.4039599448253883
XP_230471	-1
XP_230472	0.6499744610093965
XP_230473	2.753033948160692
XP_230478	2.0053762021161625
XP_230479	-1
XP_230480	1.0805133093453791
XP_230486	1.2270585797812428
XP_230488	-1
XP_230491	0.9758778717888895
XP_230493	1.009622695284416
XP_230494	-1
XP_230495	0.7154510028326079
XP_230497	0.9736642166552995
XP_230500	0.8262799414415193
XP_230519	0.369032631473555
XP_230520	-1
XP_230531	0.9939411159139346
XP_230532	0.9393683826161335
XP_230537	-1
XP_230548	1.1773193263001998
XP_230550	0.9152861185681216
XP_230555	1.4889368364941225
XP_230557	0.8602519591626319
XP_230561	1.322194821741158
XP_230564	0.2751799974907924
XP_230567	0.6794358583321943
XP_230568	-1
XP_230578	-1
XP_230583	1.1201939238972245
XP_230584	1.63807649550072
XP_230589	0.8785057645462592
XP_230592	-1
XP_230598	1.4577277404228886
XP_230604	1.279075362068211
XP_230608	1.1855234156863328
XP_230616	0.838678998958783
XP_230625	0.9267816252562274
XP_230634	1.089492274696111
XP_230635	-1
XP_230637	1.0180464142919816
XP_230638	0.8784827818007684
XP_230641	1.0308568943552632
XP_230644	1.0331157088013123
XP_230647	2.2396574926065127
XP_230654	1.1491735921420616
XP_230663	-1
XP_230701	1.0528511845101798
XP_230715	-1
XP_230716	1.2442533622049101

XP_230719	0.8509125962185807
XP_230723	1.0463545539213928
XP_230734	1.625681576955585
XP_230740	3.716765847712579
XP_230750	1.1271569242144175
XP_230758	1.1503272899468842
XP_230765	1.2314237077570278
XP_230770	1.2017297551230797
XP_230774	1.0827798623267795
XP_230778	1.0636559752795576
XP_230784	-1
XP_230785	1.2728280065544981
XP_230793	1.16441363370516
XP_230795	1.2957980470785653
XP_230798	0.9347099737834048
XP_230799	1.0250364458118573
XP_230802	1.402761370501904
XP_230814	0.8854830084905918
XP_230837	2.0322118166832945
XP_230844	0.9369464976794019
XP_230845	1.1177864613634936
XP_230846	1.1645282525620355
XP_230848	-1
XP_230849	0.9626730330275023
XP_230854	-1
XP_230856	1.2386885316892395
XP_230860	0.8894671300329134
XP_230861	1.3604710362980483
XP_230869	1.1253871386214138
XP_230874	-1
XP_230877	1.2227717755729655
XP_230878	0.9008319245674462
XP_230880	1.223585591035132
XP_230881	0.7298504861496905
XP_230890	1.217258538420211
XP_230892	0.7566930734879477
XP_230899	2.228376670406386
XP_230900	1.0880465136008375
XP_230940	1.1517985734857787
XP_230952	1.290528194131779
XP_230955	1.2085319002313402
XP_230961	1.2092863375559868
XP_230967	0.8297444264007074
XP_230968	0.8672104906154502
XP_230973	1.087909960423878
XP_230982	1.024121461518882
XP_230983	1.1849581999749692
XP_230986	1.0623422940610148
XP_230988	1.0234392127913867
XP_230990	0.9456924124746829
XP_231003	0.7609749651747705
XP_231032	0.941649483677632
XP_231041	-1
XP_231046	0.9477578555693628
XP_231047	1.2525556424110258
XP_231055	-1
XP_231069	-1
XP_231083	1.0551895789839933
XP_231085	3.695549735898633
XP_231090	1.1469340061420428
XP_231115	1.49430379366354
XP_231118	-1

XP_231121	0.7998528988594797
XP_231122	0.8414436640336742
XP_231133	-1
XP_231135	1.1238541929451193
XP_231139	1.1333064151275294
XP_231144	1.0993421641205472
XP_231147	1.163887209228363
XP_231148	0.6276944451338907
XP_231153	1.3617396781506905
XP_231161	0.988274519982396
XP_231165	1.7321993819797326
XP_231168	1.1354718801169301
XP_231172	1.1951484718908565
XP_231184	-1
XP_231192	0.7305850331024687
XP_231193	1.2345623392953051
XP_231214	1.1030585082829305
XP_231220	1.0729332539244296
XP_231230	0.7601775152798308
XP_231245	-1
XP_231251	1.417320028984294
XP_231271	1.475559020158433
XP_231276	-1
XP_231279	1.1024084744986202
XP_231287	1.0357348772103796
XP_231295	1.0796896705546126
XP_231302	0.8222920280457569
XP_231307	1.1617663216016945
XP_231354	1.4573031984734892
XP_231361	1.1056254493414115
XP_231505	0.7330390450673109
XP_231552	1.2131637175130445
XP_231561	1.0580667840469338
XP_231594	1.336248790096361
XP_231599	0.9566601590396377
XP_231616	1.0677306670499425
XP_231617	1.007155957127714
XP_231625	-1
XP_231655	0.9658598700228717
XP_231658	1.1654603853912398
XP_231707	1.173886431619468
XP_231724	0.9367798131888233
XP_231725	-1
XP_231728	1.0766851548878944
XP_231739	1.3457734306260747
XP_231749	1.045117826291473
XP_231763	1.4062337195186199
XP_231785	1.1057313990110502
XP_231803	1.1832017221014215
XP_231860	1.3397378770251862
XP_231873	1.1251394213272226
XP_231925	0.9098236800246023
XP_231999	0.8035467072467822
XP_232055	0.8993230842528758
XP_232064	1.1054865080428562
XP_232102	1.0184018762974452
XP_232111	8.824826609176588
XP_232114	0.7766277223839445
XP_232123	1.206333571550619
XP_232134	0.6212878194839653
XP_232139	1.0738779908974467
XP_232140	1.068274566235975

XP_232170	1.1963147806083223
XP_232194	0.8427867534056238
XP_232195	0.993069380568674
XP_232197	1.2885574988451962
XP_232200	1.0305065651654606
XP_232237	0.8587811446595438
XP_232246	0.8658284302678543
XP_232252	1.2489246709977135
XP_232253	-1
XP_232270	0.6156852193964643
XP_232273	1.30938435792344
XP_232276	0.7701543647850163
XP_232281	1.3121713047290304
XP_232283	-1
XP_232287	1.119279483454792
XP_232293	1.0781031033024966
XP_232323	1.1222296545146446
XP_232335	0.9667056678893227
XP_232336	0.846799311388384
XP_232343	0.9870439040004073
XP_232347	-1
XP_232350	0.9174665238802039
XP_232351	1.0106127326532826
XP_232354	1.0655691321081115
XP_232363	-1
XP_232370	1.137124487638665
XP_232400	-1
XP_232418	1.367017816015809
XP_232463	1.1289339149110547
XP_232466	0.9098621926139616
XP_232475	1.5487896372897108
XP_232477	1.2947258791247886
XP_232488	1.0346961589552903
XP_232531	0.6794484686448857
XP_232536	0.8564640888571898
XP_232578	1.0634651673236644
XP_232586	0.9061666166416827
XP_232587	1.4928591672701101
XP_232608	-1
XP_232614	1.0126435926202717
XP_232615	1.0014513050534106
XP_232620	-1
XP_232640	-1
XP_232641	1.0196782559644137
XP_232646	1.267236971258267
XP_232648	0.9289642666235786
XP_232651	1.0069578851130454
XP_232671	1.2194149056775068
XP_232675	0.6112997516534155
XP_232701	0.6817717857164212
XP_232709	-1
XP_232716	1.1248461886435825
XP_232732	1.386853518578736
XP_232742	1.014287727741651
XP_232745	1.8817266441834721
XP_232747	-1
XP_232757	0.9875636210342225
XP_232763	1.1345068354224785
XP_232766	1.3868032936628396
XP_232769	0.7351439105732296
XP_232775	0.9701917817424666
XP_232778	0.8561807871043817

XP_232779	1.1208997682119732
XP_232784	1.3866184934050034
XP_232785	0.9039321359400493
XP_232797	0.9645577549055421
XP_232819	1.078739454046589
XP_232828	1.5643789617438213
XP_232837	1.0812784539495557
XP_232847	1.489673971675272
XP_232859	1.1638322203209854
XP_232860	1.2156178799121757
XP_232879	0.9360601968124893
XP_232884	1.0650799256349457
XP_232889	0.9257289740589189
XP_232897	1.0738435791638827
XP_232898	2.4145024647387916
XP_232901	0.9701024740898638
XP_232902	1.0735599184716131
XP_232918	-1
XP_232919	-1
XP_232929	1.0638334623073968
XP_232937	1.0694774511246081
XP_232941	1.0080551977586307
XP_232950	1.1184391276801764
XP_232963	1.0953928490427
XP_232972	0.9583991616798077
XP_232983	1.3072998172572792
XP_232987	1.226387541675045
XP_232988	1.1794544793204238
XP_232990	-1
XP_233004	0.9472076933380591
XP_233017	-1
XP_233031	0.9263827763784249
XP_233037	0.9361491926814276
XP_233039	0.9391875053825389
XP_233065	0.39478181206978186
XP_233080	1.6575549860057794
XP_233081	1.308588010464575
XP_233093	0.9660947591669112
XP_233103	1.137312493398054
XP_233106	0.48973531810882265
XP_233112	0.7267698325498153
XP_233122	1.9868949344411424
XP_233123	1.0579575186055827
XP_233132	0.957046910134684
XP_233137	0.9045282995594924
XP_233139	0.8828406463179955
XP_233141	-1
XP_233143	1.3598184215383775
XP_233144	0.9619799564176119
XP_233182	-1
XP_233212	1.071513483875202
XP_233215	1.4648084675243116
XP_233216	-1
XP_233217	1.3526503640695218
XP_233225	0.9785574898828883
XP_233227	0.8937118912085257
XP_233231	0.9142335327620194
XP_233240	1.1078764779463564
XP_233244	1.5802637680226583
XP_233250	1.1782981349090151
XP_233266	1.1693892794812526
XP_233268	1.3711075958104093

XP_233269	2.3644254251449515
XP_233272	1.1187447215103987
XP_233277	0.9836085170471213
XP_233280	-1
XP_233288	1.045505200299422
XP_233305	0.9782658464886556
XP_233308	1.1802104017412804
XP_233313	0.7009469048890647
XP_233316	1.1240788231867649
XP_233325	-1
XP_233326	1.0664237145547644
XP_233335	0.8789306975531399
XP_233337	-1
XP_233342	1.0102518400615377
XP_233356	1.0837580026063633
XP_233371	1.0244052802878902
XP_233380	-1
XP_233382	0.9503724279141955
XP_233413	-1
XP_233417	1.0400549885765145
XP_233421	1.0062165847111952
XP_233422	-1
XP_233424	0.49695620305074195
XP_233428	-1
XP_233431	0.8383285888581279
XP_233435	1.427865352715603
XP_233437	-1
XP_233439	0.647007745948623
XP_233446	-1
XP_233448	1.2140820944063409
XP_233452	-1
XP_233453	0.868586944983397
XP_233462	1.0692402824065634
XP_233464	1.170388010182493
XP_233467	1.0779198677807047
XP_233475	1.1950255659866957
XP_233485	0.7044117475629781
XP_233493	1.043325652522422
XP_233498	-1
XP_233499	-1
XP_233500	0.9052168583784815
XP_233506	1.3976151265828207
XP_233508	1.2626775581785343
XP_233517	1.2260764350465216
XP_233522	5.810508347698616
XP_233523	1.1801128009109527
XP_233529	1.5468404693179814
XP_233535	1.2884528197133587
XP_233542	0.8127626224162064
XP_233555	0.9742564519437718
XP_233556	0.9882131108940874
XP_233567	1.4793720246825544
XP_233581	0.7020338248363358
XP_233595	0.8590081581586251
XP_233601	0.9455910594800172
XP_233602	-1
XP_233603	-1
XP_233606	1.7222554507030499
XP_233611	0.8475749341046773
XP_233616	-1
XP_233620	0.8910144817323603
XP_233629	0.7303130666117411

XP_233636	1.0074943220999983
XP_233642	1.254840992469964
XP_233669	1.085359985888725
XP_233673	2.204126464260357
XP_233676	1.1690980333243455
XP_233679	1.861737103475644
XP_233684	-1
XP_233692	0.5875494833021454
XP_233694	1.019821394234392
XP_233698	-1
XP_233700	1.2753429500660827
XP_233702	0.4585570305650258
XP_233713	1.15758669874071
XP_233719	-1
XP_233721	0.04967535737024465
XP_233726	0.9595752898222912
XP_233728	1.4994825024493463
XP_233730	0.88258516461307
XP_233737	-1
XP_233740	1.0021427814123423
XP_233741	1.4071291242480106
XP_233767	-1
XP_233782	1.140098681875372
XP_233792	0.9584020057493977
XP_233793	0.8104205334611237
XP_233798	0.8661491710825827
XP_233805	1.1626272040510697
XP_233806	0.964833893180361
XP_233812	1.8608454433433839
XP_233815	1.411770735002678
XP_233824	0.9464015627147762
XP_233830	0.5955148749205347
XP_233839	1.2830303427394267
XP_233842	1.2656442385459332
XP_233856	-1
XP_233868	0.9774964105691931
XP_233872	0.7617153381527708
XP_233883	0.9682837013642739
XP_233884	1.3718517258963132
XP_233885	1.0087285946252547
XP_233923	0.8073723232455009
XP_233931	1.1252533641478504
XP_233937	1.1813755753829152
XP_233942	1.4346394860625227
XP_233944	1.2016564051103384
XP_233945	1.310681823321361
XP_233960	1.7041874171784546
XP_233962	0.6095401776544999
XP_233964	1.8486718553895611
XP_233966	1.1001976798678934
XP_233968	-1
XP_233980	1.086757756292296
XP_233988	0.7449492366728063
XP_234008	1.1170840475911006
XP_234011	1.9189375398209592
XP_234023	1.3819147239124676
XP_234029	0.8407653282656621
XP_234038	0.5772757270683795
XP_234039	1.267889060647902
XP_234056	0.9710749662522288
XP_234077	-1
XP_234081	0.9517042300858489

XP_234082	0.7565737871339788
XP_234092	1.1036386750088656
XP_234116	0.9885689022303294
XP_234130	0.8402892933735013
XP_234139	-1
XP_234179	0.5471171437782716
XP_234186	0.94392060109587
XP_234219	0.8818563499161862
XP_234236	0.9828591138049642
XP_234255	-1
XP_234263	1.150180488150718
XP_234264	2.2083267753108813
XP_234272	0.8850869224898231
XP_234277	-1
XP_234281	1.1386494325117302
XP_234295	0.8968783998805119
XP_234300	-1
XP_234303	1.099161839834484
XP_234317	-1
XP_234320	2.5496198458399078
XP_234332	1.1023823829914594
XP_234335	0.9779675582944019
XP_234338	1.2819120483182114
XP_234345	1.021732517906428
XP_234361	0.7980160258740908
XP_234368	0.8833339416952051
XP_234377	1.1730100379644524
XP_234385	-1
XP_234393	1.1385417729520027
XP_234394	0.8983692358822676
XP_234398	-1
XP_234409	-1
XP_234412	0.9489351432819211
XP_234415	1.0436247399693646
XP_234416	1.1100550340025748
XP_234420	1.0925066029098642
XP_234422	-1
XP_234426	0.807770581649931
XP_234428	0.8944553142910681
XP_234429	1.8456339830053785
XP_234434	-1
XP_234441	1.467771042354597
XP_234442	1.3414024789180552
XP_234471	-1
XP_234483	1.1997005982417053
XP_234484	-1
XP_234491	1.1644674924402736
XP_234508	1.054027978164734
XP_234520	0.8892894533675039
XP_234523	-1
XP_234532	-1
XP_234543	-1
XP_234544	0.8036724974619921
XP_234547	1.2729311090364812
XP_234555	0.9393404974872988
XP_234558	-1
XP_234565	-1
XP_234568	1.1260175499016627
XP_234572	1.354695591652223
XP_234579	0.9464765567358445
XP_234581	0.6079677981890727
XP_234583	0.5832928204290666

XP_234588	-1
XP_234646	1.139710622002079
XP_234693	0.9456841779503581
XP_234695	0.7028700189968952
XP_234706	1.3704690654009637
XP_234709	1.057004862371486
XP_234715	1.1995094781318618
XP_234720	1.3244643326449723
XP_234725	1.0370188012907524
XP_234768	1.1177894283641154
XP_234776	1.1424426668689425
XP_234787	1.1972379529810095
XP_234801	1.2203137757851146
XP_234837	-1
XP_234839	1.2473381796395466
XP_234841	0.7936871630194212
XP_234843	0.949957753295991
XP_234877	0.7997842329483326
XP_234880	0.6888528865920128
XP_234885	0.9540938892856561
XP_234896	1.2475743762178035
XP_234900	1.0438311737341117
XP_234902	0.939102181855356
XP_234908	1.3130269149386582
XP_234909	0.8086036251668042
XP_234910	2.3893091426496524
XP_234914	-1
XP_234920	0.8953544751576928
XP_234921	0.9795755741950812
XP_234923	-1
XP_234926	0.848948387822638
XP_234928	0.6422820753904622
XP_234939	0.6572593883837241
XP_234942	-1
XP_234961	-1
XP_234965	0.9343149192560208
XP_234966	1.2406073248520302
XP_234988	0.9773749793123656
XP_234993	0.26282826644166474
XP_235003	-1
XP_235005	-1
XP_235012	-1
XP_235051	0.9156553061100986
XP_235055	1.4598507961642773
XP_235057	0.9119940991315301
XP_235069	-1
XP_235070	1.7362735521481634
XP_235083	0.9945951516339084
XP_235088	-1
XP_235093	1.0277197641356441
XP_235095	1.2106735055011246
XP_235113	1.2420294864970765
XP_235115	1.1476512017152274
XP_235128	0.9784500668149286
XP_235146	0.9134074230799569
XP_235156	1.4599091266952646
XP_235157	1.395250729443145
XP_235162	-1
XP_235168	0.9226420053092662
XP_235169	1.9718345190260302
XP_235172	-1
XP_235176	0.9507786577193472

XP_235179	1.0783555040018171
XP_235183	0.6159751822089198
XP_235198	1.7570223311712836
XP_235200	-1
XP_235207	1.098491166119787
XP_235213	1.2201167992209736
XP_235224	1.0554759275563916
XP_235248	-1
XP_235261	1.2795826886929287
XP_235288	1.2460134470566817
XP_235289	1.1480834957673056
XP_235296	1.5095003332247034
XP_235308	1.6336457787264826
XP_235318	1.408934348680547
XP_235326	-1
XP_235338	1.070837191334273
XP_235347	1.4953455271518896
XP_235349	-1
XP_235372	0.8702857017753516
XP_235375	1.3911690125553287
XP_235391	1.2489633749776898
XP_235393	0.9962354015239305
XP_235395	0.9420875868787443
XP_235398	1.0151122117524023
XP_235400	0.7796373752653828
XP_235410	0.8519793768729702
XP_235420	2.1749771998279126
XP_235426	1.385200787344742
XP_235436	0.9147577492154456
XP_235439	0.7543584730237954
XP_235445	1.0586184669244172
XP_235448	1.011398937149658
XP_235449	0.8184275867452321
XP_235452	0.9066960443048043
XP_235454	0.8679397016886721
XP_235461	0.9333753075879478
XP_235473	0.9742369353769972
XP_235474	1.2183660567807244
XP_235476	1.0513251290250503
XP_235478	0.9919662621484635
XP_235493	-1
XP_235496	0.9628766244240777
XP_235497	0.8600064470308787
XP_235500	-1
XP_235509	0.9032715425124259
XP_235511	1.032428758280895
XP_235513	1.140967734923555
XP_235515	0.6517341122997357
XP_235517	0.8033617080557862
XP_235518	0.8815578996152583
XP_235527	-1
XP_235529	0.38913992668903424
XP_235548	1.116997479151049
XP_235552	-1
XP_235555	0.9991812617640635
XP_235559	1.1544990827135362
XP_235561	1.0741756706408858
XP_235562	0.9835262574935227
XP_235563	-1
XP_235565	-1
XP_235571	-1
XP_235581	1.3133253062595887

XP_235593	1.0995495374501334
XP_235598	1.018028053132977
XP_235610	1.1930467844963477
XP_235625	0.7366395739578411
XP_235633	-1
XP_235639	1.3623452773266476
XP_235640	1.17781816889271
XP_235645	3.277529056779728
XP_235649	-1
XP_235650	0.9347818153035493
XP_235656	2.3366874664517057
XP_235661	1.289156623631223
XP_235668	1.8672408211920437
XP_235670	-1
XP_235686	2.0075802144190735
XP_235687	1.6710829158319591
XP_235689	0.9646923179879199
XP_235691	1.0143351753806606
XP_235705	1.068442643416223
XP_235710	1.172566556573679
XP_235711	1.2627074052262255
XP_235717	1.0839384362201279
XP_235722	0.9520545504589464
XP_235761	0.8546720679804257
XP_235768	-1
XP_235782	1.2208427577089775
XP_235792	1.2392667284911154
XP_235805	-1
XP_235807	0.9082589998992262
XP_235808	0.890862401771054
XP_235821	1.1367388294119605
XP_235822	0.994689219909965
XP_235826	1.9004465957550343
XP_235831	1.1811145473244482
XP_235840	-1
XP_235865	0.9270580284461772
XP_235886	0.9192904910699007
XP_235905	1.5354612794557712
XP_235922	1.0355620316237049
XP_235942	1.2810930816165405
XP_235947	1.2778572944250468
XP_235955	0.8862301743110664
XP_235959	0.6446823402443879
XP_235972	0.6985068338977082
XP_235979	-1
XP_235988	1.1182331996987438
XP_235989	0.9120257104767036
XP_235993	-1
XP_236010	1.002045676348428
XP_236020	1.0401779248323895
XP_236042	1.1386039287659167
XP_236056	1.5050523079503582
XP_236077	2.226593982855279
XP_236086	1.331262629808622
XP_236099	1.2474186614613076
XP_236102	1.2269948162865896
XP_236106	0.8690124829758258
XP_236124	1.1291246304875822
XP_236128	1.0222786779985986
XP_236134	1.2015130513807588
XP_236145	0.6340173216105203
XP_236150	1.558391664143473

XP_236151	0.930343597458004
XP_236158	0.7781741211642992
XP_236174	1.5092209287334146
XP_236179	0.8356288237861904
XP_236180	0.37478896622422636
XP_236189	0.8662638951103951
XP_236194	-1
XP_236196	1.0541750196888453
XP_236201	1.8896831468501718
XP_236203	1.3536281536124066
XP_236206	1.0827461763268504
XP_236210	0.49240471186954177
XP_236212	1.0945094572507048
XP_236221	-1
XP_236230	1.15187469974312
XP_236231	1.3650530325430401
XP_236242	-1
XP_236249	0.7938929986529449
XP_236253	-1
XP_236263	1.1554186181845698
XP_236270	2.7779711051812725
XP_236272	1.0405279802200882
XP_236274	1.186927442284228
XP_236275	-1
XP_236276	1.0343593516940752
XP_236283	1.4582557153889035
XP_236286	-1
XP_236292	-1
XP_236296	1.328642975027564
XP_236298	1.0402273031526688
XP_236306	-1
XP_236307	1.175159264327468
XP_236312	1.065784744453606
XP_236313	-1
XP_236314	1.002330416572691
XP_236320	1.1525562891105257
XP_236321	1.244392720334352
XP_236325	0.24325361999262962
XP_236329	1.1756684853451427
XP_236331	1.077242100530974
XP_236333	1.6608757082824355
XP_236338	1.2564832268959287
XP_236350	1.161053581971537
XP_236352	0.9780962476710854
XP_236353	1.0306374139958305
XP_236355	1.0304537373662201
XP_236358	-1
XP_236362	1.0110461483646251
XP_236367	-1
XP_236370	-1
XP_236381	0.9942822251792688
XP_236392	-1
XP_236397	1.2377677401656746
XP_236408	-1
XP_236412	1.249573481839152
XP_236420	1.2786471051042847
XP_236428	-1
XP_236444	1.217106377715998
XP_236449	1.1533770009725093
XP_236458	1.1581717628847115
XP_236461	0.6257628240085426
XP_236468	-1

XP_236471	1.1549676019593669
XP_236493	-1
XP_236501	0.7462622637893451
XP_236515	0.9613018769327324
XP_236524	1.2874731692821293
XP_236553	0.9582458034092497
XP_236571	0.5228165701215339
XP_236579	1.402189039920458
XP_236595	-1
XP_236602	1.7322285162192128
XP_236606	0.8731789924788994
XP_236613	1.0865000755565664
XP_236614	1.0807424333246638
XP_236616	1.127067575860756
XP_236624	1.1040290933593495
XP_236627	1.004846291389817
XP_236628	-1
XP_236629	-1
XP_236630	0.4528729855408629
XP_236639	1.0294577239472167
XP_236644	0.7767244898268455
XP_236646	0.7164447477150505
XP_236649	1.0768382892124118
XP_236654	1.367676703003825
XP_236656	0.746538820067861
XP_236658	0.8178573249830222
XP_236659	1.4362498048166101
XP_236665	1.2143437001391761
XP_236675	1.1003507977902691
XP_236676	0.2285536976529797
XP_236685	0.8844973215723683
XP_236698	1.0336219619664748
XP_236702	1.0671943293219737
XP_236703	0.986584776860139
XP_236715	1.1405019675954229
XP_236718	1.2714289057249635
XP_236722	1.1757742461413547
XP_236723	1.7716147193244365
XP_236725	0.83731544822543
XP_236726	0.7567907936527841
XP_236730	1.0027152323042212
XP_236734	1.433441285967889
XP_236735	0.9581462818124448
XP_236745	0.9580181499788339
XP_236746	-1
XP_236755	-1
XP_236768	1.8742067809726888
XP_236769	1.1004554602326504
XP_236774	1.2938866881120414
XP_236783	0.8934906636329454
XP_236784	1.1687793834478002
XP_236798	0.9630999827502926
XP_236813	1.38574872436672
XP_236820	1.4949940910059674
XP_236822	1.3060899226664728
XP_236825	1.2291822606674472
XP_236870	1.1104347502633236
XP_236909	0.9756865271151339
XP_236911	0.965345123353122
XP_236914	-1
XP_236916	1.1503443167947838
XP_236917	1.871282823389257

XP_236920	1.4882279722415241
XP_236926	0.5805082623866543
XP_236927	0.9751845015713316
XP_236929	0.8715940466279031
XP_236930	1.0173075360532076
XP_236932	1.0730447157615814
XP_236934	-1
XP_236937	0.8089894823810768
XP_236953	1.213318660001526
XP_236955	0.8764637431499738
XP_236956	1.039595164453093
XP_236979	0.7578883717848792
XP_236984	0.7955093761085339
XP_236988	1.1304980510640779
XP_236990	-1
XP_237009	-1
XP_237019	1.292146284002406
XP_237039	0.841332069971976
XP_237042	1.2854326048361309
XP_237046	1.1016301118166967
XP_237056	1.0724961737806862
XP_237064	1.0701490391113115
XP_237071	0.9847707350300414
XP_237073	1.3454707281047693
XP_237079	1.0244316643876865
XP_237084	1.0831710907912555
XP_237086	-1
XP_237087	1.0716527942873313
XP_237088	0.7640372813536128
XP_237091	1.5357811498872613
XP_237093	0.9023785533557079
XP_237102	0.6872641048075053
XP_237111	0.816020954096247
XP_237112	1.8845535043790491
XP_237113	-1
XP_237115	0.98607586064201
XP_237116	0.9664899815180675
XP_237147	-1
XP_237151	0.9951050391784905
XP_237166	1.0299861922171567
XP_237174	1.102933841395639
XP_237177	1.1595909920324834
XP_237180	1.1523278446482241
XP_237184	1.2232121699678413
XP_237187	1.382797019915915
XP_237191	0.7942011817884023
XP_237195	0.627058555046344
XP_237215	-1
XP_237217	1.6134004362566798
XP_237232	1.1222550435157592
XP_237238	1.4073590110319818
XP_237241	1.0720713557718748
XP_237242	1.048149384077183
XP_237246	1.3078614259871608
XP_237255	-1
XP_237287	1.1179715095616967
XP_237291	0.849229872915816
XP_237293	1.0110421158170604
XP_237295	1.3771631083894826
XP_237311	0.8466721128268332
XP_237313	0.9295454089669274
XP_237316	0.7555282180025954

XP_237323	0.71358546676663
XP_237347	0.9008272874209476
XP_237359	-1
XP_237363	1.256403339023341
XP_237365	1.6297961282419327
XP_237369	1.1596191074668136
XP_237375	1.446033886632578
XP_237386	1.127322326117124
XP_237388	1.2267959881653845
XP_237395	0.8637323550085007
XP_237416	0.8857151284643239
XP_237421	-1
XP_237444	1.0291991129561082
XP_237454	1.7963126535804446
XP_237464	1.3816014614722296
XP_237474	1.1355241743200335
XP_237497	-1
XP_237509	1.0647049638075077
XP_237523	0.946689154673993
XP_237524	1.3625029802912423
XP_237535	0.11926255016554747
XP_237536	2.8491992808486377
XP_237548	1.2666082453343044
XP_237588	0.909147170218275
XP_237614	1.0021652606191715
XP_237701	1.859999574270324
XP_237718	1.3848339154874163
XP_237746	0.9882410899047184
XP_237754	-1
XP_237758	-1
XP_237764	0.9903826642005953
XP_237773	0.9241307931156586
XP_237782	0.8191799880397616
XP_237786	1.1916061140396643
XP_237787	-1
XP_237790	0.7479791813370956
XP_237792	0.050925496206180774
XP_237807	0.896969197508611
XP_237808	1.0221286196421837
XP_237817	1.2312543522299455
XP_237825	0.893305705975223
XP_237828	0.9744585387731023
XP_237842	1.0469891630738546
XP_237865	0.9137002876308424
XP_237868	1.1660157576721517
XP_237878	0.9175580310262124
XP_237883	0.650535768809421
XP_237884	-1
XP_237903	0.8816492208834203
XP_237905	0.953050606064726
XP_237911	-1
XP_237924	1.4125507928412109
XP_237930	1.0779643774210417
XP_237944	0.5872780895007309
XP_237957	0.7980220918773737
XP_237959	-1
XP_237965	0.8722765294200726
XP_237967	0.04536699267941055
XP_237971	3.0061370462053567
XP_237984	0.9265742426696206
XP_237992	1.0228815100429132
XP_237998	1.2111030810554488

XP_237999	0.8576770123121789
XP_238004	1.1471213363128039
XP_238005	1.120547024134397
XP_238016	1.3676712481361353
XP_238019	1.0576074728265477
XP_238028	-1
XP_238042	-1
XP_238048	1.2091717388724237
XP_238057	0.9648519929586757
XP_238063	1.107668902573774
XP_238072	-1
XP_238078	1.0539960054781203
XP_238082	1.2996788679406028
XP_238088	1.0987743309591207
XP_238093	1.2876871335652778
XP_238097	1.0109305409162637
XP_238103	0.9374158379340815
XP_238104	0.8454041300688875
XP_238106	-1
XP_238109	-1
XP_238119	1.993212397517153
XP_238138	1.0489498076092783
XP_238141	1.0274846320937538
XP_238146	0.9417968410138627
XP_238151	0.3086922656759667
XP_238154	1.0099429129071213
XP_238155	0.8629512896250645
XP_238163	0.5964302724182832
XP_238166	1.1650479926482764
XP_238167	1.2150159017918474
XP_238190	0.7726791444493801
XP_238193	1.1781226522924355
XP_238205	-1
XP_238208	1.0817303049934748
XP_238213	1.1236841185976973
XP_238215	0.6240503842104307
XP_238235	1.1057057816165725
XP_238238	1.1190945129201313
XP_238243	0.8969577312954436
XP_238245	1.8502139162052507
XP_238247	0.9380729467348196
XP_238278	1.0256221081930879
XP_238280	0.9602300299717381
XP_238285	1.9814369349827647
XP_238286	1.289081729006272
XP_238302	-1
XP_238312	-1
XP_238313	1.0100683500210075
XP_238320	0.8910010201764926
XP_238321	1.841762202233772
XP_238325	1.5223764012283088
XP_238327	0.8872634828543696
XP_238330	0.9513283905961823
XP_238333	0.9339411869399922
XP_238336	1.324184800551594
XP_238346	0.9691783097463943
XP_238366	-1
XP_238368	1.1485017550249113
XP_238369	1.11938947708645
XP_238380	0.9339079938384803
XP_238382	0.9625926830410327
XP_238384	1.2264999615155103

XP_238392	-1
XP_238393	0.8878103088828682
XP_238396	1.0428033942915307
XP_238407	0.7074278638058688
XP_238408	-1
XP_238415	-1
XP_238425	0.5265478213753556
XP_238442	1.277983450800925
XP_238444	1.1184835613796833
XP_238447	0.9401582533174201
XP_238462	1.2587661305351443
XP_238465	0.9967369442583806
XP_238467	2.1865364481034755
XP_238474	1.2201657863953919
XP_238480	1.2401692042682726
XP_238508	1.1179081705119092
XP_238509	0.560776621461277
XP_238518	0.9600797926493406
XP_238523	1.940219254766805
XP_238534	0.8117465157523603
XP_238535	0.48144883771222063
XP_238540	0.9690560273150886
XP_238543	1.0420506699145629
XP_238554	1.0519462983081542
XP_238561	0.9202380580842137
XP_238569	0.924552841146578
XP_238570	0.6692863613377887
XP_238571	1.0160609313233515
XP_238588	0.824362135757907
XP_238596	1.1920589285481873
XP_238622	1.326084899435202
XP_238649	1.2507175647030082
XP_238764	-1
XP_238770	0.9856906636402738
XP_238783	0.7351158204026221
XP_238806	0.785372853422755
XP_238899	1.9787015962494932
XP_238901	1.1523921595076294
XP_238988	1.0517003798313607
XP_239014	1.054994402969786
XP_239062	1.0351484724292834
XP_239083	1.110065273329282
XP_239095	-1
XP_239135	0.9247976152939684
XP_239171	1.2837083627794754
XP_239247	0.6886385189040288
XP_239254	1.1147395816153016
XP_239258	0.9887964160520061
XP_239260	1.394475731505426
XP_239269	1.2198903318751895
XP_239329	1.180724821574266
XP_239335	0.9910269850902064
XP_239336	0.992693655474461
XP_239340	0.7596612051604638
XP_239346	0.9302668252170756
XP_239373	0.9327214897055475
XP_239438	1.5756530895601741
XP_239603	1.4347948675415105
XP_239606	-1
XP_239684	0.8959251497370745
XP_239761	-1
XP_239866	1.8042051808643222

XP_239920	1.0671143009588828
XP_240072	0.987227799821849
XP_240184	0.8129752985149606
XP_240311	-1
XP_240330	2.2244511357159764
XP_240417	1.0672963555947517
XP_240446	1.4075215487281043
XP_240464	1.131930876619947
XP_240482	0.8559869106885571
XP_240734	0.9676874131235695
XP_240978	1.109780515886654
XP_241053	1.2195129958397406
XP_241375	1.1058394827093128
XP_241458	1.258356726694734
XP_241475	-1
XP_241525	1.9332198354620391
XP_241623	-1
XP_241632	1.2104632137746054
XP_241671	1.1803022300939106
XP_241691	1.3635721945028678
XP_241769	0.8982647431291362
XP_241770	1.1375209757555294
XP_241848	-1
XP_242005	1.280766364720866
XP_242065	0.6530649534882886
XP_242556	0.9132729478165396
XP_242644	0.7175766340349493
XP_242940	0.9667325046699249
XP_242960	0.8441539847726713
XP_242982	0.968791752100387
XP_242992	0.6502939672364361
XP_243032	1.000329374526422
XP_243038	0.895604823203822
XP_243040	1.0357820862299445
XP_243049	0.9548887232126134
XP_243122	1.130902885707924
XP_243142	0.9671066051266153
XP_243280	1.1196743773838371
XP_243307	-1
XP_243465	1.0710601292574422
XP_243478	-1
XP_243524	1.1492927001381439
XP_243588	1.2480043792491615
XP_243637	2.254538726946833
XP_243652	0.9385389348817226
XP_243749	1.1190507963620964
XP_243863	0.6960708075020821
XP_243912	0.8954010088764782
XP_243980	1.0099807582627882
XP_244043	1.074160578364082
XP_244124	-1
XP_244148	1.0717012265294883
XP_244282	-1
XP_340739	1.012439226516605
XP_340740	-1
XP_340741	0.9427884174387184
XP_340743	0.9438655699387343
XP_340744	2.2760044966601956
XP_340748	2.004593203420918
XP_340749	-1
XP_340750	1.0363417103219694
XP_340756	1.090665492520436

XP_340761	0.7696521415883602
XP_340762	0.910812227404468
XP_340764	0.3488127079419616
XP_340765	1.0608341254479052
XP_340766	1.0234164756738806
XP_340769	0.9504880429947081
XP_340770	1.1228139882241275
XP_340772	1.0130463712192312
XP_340774	-1
XP_340775	0.9266566206237451
XP_340776	0.8293472602650721
XP_340784	1.118031840738737
XP_340790	1.0199347344632212
XP_340793	1.227252074618191
XP_340794	0.7309091079073547
XP_340797	1.0442728062695878
XP_340799	0.5837635484419647
XP_340800	-1
XP_340802	-1
XP_340803	0.9303332404180843
XP_340804	1.0842308833161234
XP_340806	0.8879036691157127
XP_340807	1.20563914701935
XP_340808	1.5081188813562336
XP_340810	0.6739948373549319
XP_340814	1.1746601836367767
XP_340817	1.0964574247035366
XP_340818	8.273865583487854
XP_340820	-1
XP_340821	-1
XP_340823	1.421939990954565
XP_340825	1.234466732536372
XP_340826	-1
XP_340828	0.8421253248615548
XP_340829	-1
XP_340832	1.0888809652143572
XP_340836	1.033838616507236
XP_340839	1.5709575014227506
XP_340842	0.7642140377901461
XP_340847	-1
XP_340849	1.3760452453826302
XP_340850	0.8415449148129399
XP_340855	1.2852523146933879
XP_340857	0.8763827328479855
XP_340858	0.9774094027963893
XP_340865	-1
XP_340867	-1
XP_340869	-1
XP_340870	1.1794436039144123
XP_340871	1.5699949251447232
XP_340875	1.0476760966095002
XP_340876	1.2812052735848078
XP_340881	0.983394353482927
XP_340882	-1
XP_340887	1.1123652981581165
XP_340889	1.0210770100169997
XP_340890	1.036088123083593
XP_340892	-1
XP_340893	1.255536762498492
XP_340894	0.8641950907540976
XP_340896	-1
XP_340897	-1

XP_340898	1.0240445551465378
XP_340905	1.0967496860547152
XP_340906	1.1482654233966894
XP_340910	0.8506456387221708
XP_340911	0.85248874287333
XP_340912	-1
XP_340913	-1
XP_340914	1.17124005060654
XP_340915	0.9808095482834155
XP_340918	1.1674695918950937
XP_340919	1.111708522736545
XP_340922	-1
XP_340929	1.006252464213711
XP_340930	0.8564358783339835
XP_340931	3.046111438444786
XP_340937	1.3416684628769626
XP_340938	1.0709383815170752
XP_340939	1.0920221277171838
XP_340940	0.938646249743229
XP_340942	1.1098894052035992
XP_340947	1.1737920031995728
XP_340948	1.5863120357930909
XP_340949	0.8759325905411327
XP_340962	1.1772489221621367
XP_340967	-1
XP_340968	0.9661919404267174
XP_340971	1.0921897984615778
XP_340974	1.335968757786782
XP_340978	0.9935537560428226
XP_340981	1.2296782648904148
XP_340985	1.0572907802274456
XP_340987	1.4432414568861311
XP_340988	1.135607955469178
XP_340989	1.1786146361514718
XP_340994	0.9614757543632385
XP_340995	0.5508366742607586
XP_340997	1.0488939355058902
XP_340998	1.0111894123329142
XP_341000	0.6387911740862883
XP_341005	0.8692337535833444
XP_341008	0.9824580682435772
XP_341010	-1
XP_341011	1.1299768336618674
XP_341012	1.004503054662781
XP_341016	2.352726242369905
XP_341018	0.8754396383011173
XP_341020	-1
XP_341021	0.8161562522329446
XP_341034	0.9978359739357511
XP_341038	0.9279410000506007
XP_341040	1.1116537274558058
XP_341041	1.0526500366136216
XP_341042	1.6058789792271997
XP_341044	0.5467935280939958
XP_341045	0.7753524250949949
XP_341049	1.194150385747559
XP_341052	0.842377685111638
XP_341056	-1
XP_341059	0.9620297564400355
XP_341064	0.8434609992244787
XP_341072	-1
XP_341077	0.8901077557790306

XP_341079	0.7663943562833421
XP_341080	1.8872202517069885
XP_341091	1.377763492359137
XP_341093	1.6123449025968295
XP_341094	1.1386514113260222
XP_341095	0.9009145220424206
XP_341101	0.9768283274432236
XP_341103	-1
XP_341111	0.7271639236447623
XP_341112	-1
XP_341113	1.3078736948723777
XP_341115	-1
XP_341116	0.9876304198859835
XP_341119	1.0259931790992043
XP_341121	0.8493058347177037
XP_341122	0.9798868630450412
XP_341125	1.449679391046421
XP_341133	-1
XP_341134	1.1298192194922472
XP_341137	0.9571231289514083
XP_341138	1.0446515408367012
XP_341141	1.1811930982569925
XP_341142	0.7424998527846527
XP_341148	1.0938989977051359
XP_341149	1.567421569454259
XP_341152	1.0142152866265652
XP_341153	1.1262102643202565
XP_341156	1.2753217927279705
XP_341160	1.091325317025231
XP_341162	1.2997957648995808
XP_341163	0.9316619367804964
XP_341166	1.1770133497941402
XP_341170	0.8950785750994533
XP_341172	1.0905582838837309
XP_341176	0.7227370787293382
XP_341181	0.8171071896230586
XP_341184	1.2331785491066392
XP_341189	0.6287000942936692
XP_341197	-1
XP_341202	1.2071095068994504
XP_341205	8.574174245437133
XP_341208	1.127623693722394
XP_341211	0.9435537913025045
XP_341212	1.307272760571233
XP_341220	-1
XP_341225	1.2978416519197566
XP_341226	-1
XP_341228	-1
XP_341231	1.0793226882336449
XP_341232	0.9078992072453951
XP_341233	-1
XP_341234	5.237397326634471
XP_341237	0.9804050905480421
XP_341238	-1
XP_341240	0.7973539473612558
XP_341241	-1
XP_341242	0.7649887136209997
XP_341245	-1
XP_341246	0.7815712763665328
XP_341249	1.3981120830615523
XP_341250	0.4813242524650107
XP_341251	0.9084935439829539

XP_341254	0.9329244963076769
XP_341257	1.2445075714697234
XP_341259	-1
XP_341260	1.0806552311570543
XP_341263	0.9373330915720096
XP_341265	1.15236236688828
XP_341267	1.0685884179763043
XP_341273	0.8569650600177369
XP_341280	1.0525343263349811
XP_341281	0.7247623267968463
XP_341282	0.98249745963837
XP_341289	1.2340100756556125
XP_341290	0.28119420163598713
XP_341305	0.914319222465601
XP_341306	1.1968153934736656
XP_341307	1.3461896569512932
XP_341310	1.695605004561731
XP_341311	1.1245449887168795
XP_341313	0.9670161355665869
XP_341315	0.8444266163363002
XP_341318	1.054282264794369
XP_341319	1.1669269938061153
XP_341320	0.8123232933942134
XP_341321	1.2532767532737468
XP_341322	1.027451116234895
XP_341323	0.726429755929078
XP_341326	157.26728342591457
XP_341332	1.035812114197352
XP_341333	0.7729027356406339
XP_341336	0.8754837818587231
XP_341338	1.0158296127885171
XP_341340	0.8432880303966893
XP_341342	-1
XP_341347	1.0563266098519444
XP_341348	1.0590248113833935
XP_341350	0.9900389501793306
XP_341352	0.8589729941289174
XP_341353	-1
XP_341354	1.02424927050798
XP_341355	0.8044823314098405
XP_341359	1.295204995124669
XP_341361	0.9610706766781295
XP_341367	0.9248255181987739
XP_341369	0.9649825402326124
XP_341375	1.1470702129522021
XP_341377	0.9410454594779104
XP_341378	1.3332373707422138
XP_341382	-1
XP_341384	0.8632695632610544
XP_341387	0.7724126821314462
XP_341390	0.24227739633938242
XP_341394	0.9595226212286079
XP_341395	-1
XP_341398	0.6338528795656219
XP_341399	1.1291802128505253
XP_341409	1.043047276310748
XP_341413	-1
XP_341414	0.9304070455298357
XP_341415	0.7498120739883736
XP_341416	0.906593915734487
XP_341417	-1
XP_341420	1.3464497306183925

XP_341424	0.8463536738942397
XP_341425	-1
XP_341429	1.9558958209648123
XP_341435	0.8133001539439482
XP_341436	0.8798986217524325
XP_341437	0.9486316412220583
XP_341439	1.4275734683065828
XP_341441	1.169143082245193
XP_341445	1.6612454530698642
XP_341448	-1
XP_341451	0.47313919139113336
XP_341454	1.1784093885148905
XP_341455	-1
XP_341456	1.2228950102514364
XP_341457	0.8461222015405849
XP_341463	1.104525299773708
XP_341467	1.581807519201537
XP_341468	1.3126955468863164
XP_341469	0.9020683189990667
XP_341471	-1
XP_341475	0.7057378430943394
XP_341476	1.1327166417225885
XP_341478	1.4661760775208943
XP_341480	1.09583196971222
XP_341482	1.5477516886202105
XP_341485	1.795747456464813
XP_341486	1.3103567823587985
XP_341491	1.1839526497887105
XP_341495	1.5919253954888533
XP_341498	1.231525003714786
XP_341499	0.9656297393124542
XP_341500	-1
XP_341501	1.487096165776899
XP_341504	1.1299036852930158
XP_341507	1.269831939492405
XP_341509	1.2285287363275972
XP_341510	0.9617506754584877
XP_341511	0.9997962174392723
XP_341517	1.0397858705535077
XP_341518	-1
XP_341520	1.2191164055214945
XP_341521	-1
XP_341523	-1
XP_341524	1.031328785364941
XP_341526	0.9696805441940946
XP_341528	1.2437924978557724
XP_341531	-1
XP_341534	1.0703724921619844
XP_341535	1.2197011933233104
XP_341538	1.0285156015689338
XP_341539	1.0452472358063436
XP_341540	1.3413780343330763
XP_341541	-1
XP_341543	0.9952830301884091
XP_341546	0.9049806599214741
XP_341547	0.9822319792903624
XP_341549	-1
XP_341550	0.8327444902530617
XP_341553	0.9456969539163111
XP_341554	0.9210648644699972
XP_341559	1.055504380927928
XP_341568	-1

XP_341569	1.1229821572280572
XP_341571	0.9901906148498814
XP_341572	0.9443297379796247
XP_341574	0.8622366665816389
XP_341575	1.0796488591235907
XP_341580	1.0176204765343106
XP_341581	0.9915984403809673
XP_341583	-1
XP_341585	0.9317866707036437
XP_341586	0.9981761820526215
XP_341588	0.9033117813469635
XP_341589	0.9808103141754441
XP_341593	1.0413882991448924
XP_341594	-1
XP_341597	0.6913683124119389
XP_341601	0.9196431453879339
XP_341606	1.0868475741642973
XP_341609	1.0295351341762893
XP_341611	1.061120070929149
XP_341616	1.3729018636245123
XP_341619	1.731864539427833
XP_341620	1.0663557000235153
XP_341622	-1
XP_341623	0.7692956965021368
XP_341624	-1
XP_341630	0.9956217394514245
XP_341631	1.0963643821714895
XP_341637	0.9109775691792441
XP_341642	-1
XP_341644	0.8880789037309637
XP_341650	2.960007188321937
XP_341653	0.9702476483365264
XP_341654	0.7389980134271319
XP_341658	0.4559728940987146
XP_341660	-1
XP_341662	0.7842192749954219
XP_341663	2.8321533637381973
XP_341664	1.1610955697124445
XP_341665	0.896365724544848
XP_341667	1.3843316843924929
XP_341668	1.209039374920686
XP_341672	1.1040139975276193
XP_341676	-1
XP_341677	1.1752833059134125
XP_341680	1.014002619922112
XP_341684	1.2410920144518334
XP_341689	0.9168726904792625
XP_341690	1.3670809201852379
XP_341692	1.8602819640623356
XP_341694	0.6050522187641586
XP_341703	-1
XP_341704	0.9064119832386414
XP_341705	1.1616163166493167
XP_341709	1.3556802192339612
XP_341710	1.6071810076992505
XP_341715	0.8680157536744106
XP_341716	-1
XP_341717	1.0674879897678968
XP_341720	-1
XP_341722	1.3772501423678927
XP_341723	0.8947889036103697
XP_341725	-1

XP_341727	0.8694338269743339
XP_341728	1.2245170656787587
XP_341730	0.8617375723697105
XP_341731	0.6862025926083363
XP_341733	0.8177432738158502
XP_341734	1.122995232496536
XP_341738	-1
XP_341743	0.5986535132830424
XP_341746	0.7126645154304126
XP_341751	1.544977791295081
XP_341756	1.4220540751105932
XP_341758	-1
XP_341760	1.1551346776774523
XP_341763	1.4589611211779074
XP_341764	1.1054274811965368
XP_341765	0.6189671434531745
XP_341773	1.1328157539834076
XP_341774	0.7974827315697617
XP_341780	-1
XP_341782	-1
XP_341785	0.7023820076979906
XP_341786	-1
XP_341789	1.135335173782847
XP_341791	1.095706080325759
XP_341796	1.0678230772961221
XP_341797	0.7256521667770145
XP_341800	1.0523846338516947
XP_341801	1.1145512644769215
XP_341802	0.9040906845834162
XP_341804	1.0659191717294314
XP_341805	1.1699973632823308
XP_341806	0.9275190858418317
XP_341809	1.2613730019988065
XP_341810	1.0500916555328454
XP_341812	0.7514351430928928
XP_341813	-1
XP_341816	1.1011209415382885
XP_341818	1.3054438565967874
XP_341819	1.1514027807422236
XP_341823	-1
XP_341825	0.9402643866524474
XP_341826	-1
XP_341829	1.2802691371654784
XP_341830	1.0274163380281909
XP_341832	0.9683344836497512
XP_341833	0.9657096405218075
XP_341835	1.362699017251282
XP_341836	1.1795461039564343
XP_341838	-1
XP_341843	1.1312757264950215
XP_341848	0.768546915949968
XP_341849	1.4511135170877678
XP_341851	0.7297057506708189
XP_341852	1.1305287002023323
XP_341853	0.9913836042589622
XP_341855	0.6971120943828746
XP_341858	1.1252235721225674
XP_341861	0.8640284183595762
XP_341862	1.123375843653126
XP_341864	0.7634510725372438
XP_341868	1.3313049816419922
XP_341870	1.2239186759619605

XP_341871	1.0706392961846969
XP_341875	0.9436299434534596
XP_341878	1.1054917131162199
XP_341881	-1
XP_341883	-1
XP_341893	1.00716099013023
XP_341894	1.0841557047427086
XP_341895	1.5286551280059666
XP_341896	1.1399058025408406
XP_341897	1.4639453270242546
XP_341902	1.7752139288807425
XP_341903	1.1213394459311659
XP_341907	1.1848193433419016
XP_341912	-1
XP_341913	1.2167517967261867
XP_341916	-1
XP_341917	1.0924764088878043
XP_341922	-1
XP_341925	1.220190728134724
XP_341928	0.9775717316451872
XP_341929	1.1226764181661544
XP_341930	0.7985631636244874
XP_341931	1.1932450868903792
XP_341932	-1
XP_341933	1.052081242889525
XP_341935	1.2152778988381872
XP_341937	0.6013574128328955
XP_341938	1.0691701152198578
XP_341941	1.2096664733797569
XP_341942	-1
XP_341944	1.1685016427085964
XP_341948	0.9313108136272392
XP_341950	-1
XP_341951	1.2260165598165624
XP_341953	1.9425840867129798
XP_341954	-1
XP_341956	1.1434940644578406
XP_341958	0.6636995684043744
XP_341959	2.1716791788763796
XP_341966	1.0843211312445546
XP_341967	3.137167457160256
XP_341976	-1
XP_341977	1.0445761043298383
XP_341982	1.0221059866060633
XP_341984	3.52996070145152
XP_341993	1.0726917611494502
XP_341995	-1
XP_341996	0.9525214873109816
XP_341997	1.2169307763181343
XP_341998	0.9879948668826583
XP_342001	1.052600362136751
XP_342002	1.1674953963579413
XP_342003	0.9664932503240545
XP_342004	0.7158049329496623
XP_342005	1.2682424842196214
XP_342006	0.7707157949842949
XP_342007	0.913553180703259
XP_342008	1.3545370351370607
XP_342012	-1
XP_342014	0.8075437039745371
XP_342016	1.2245898797497443
XP_342021	0.9612390575193951

XP_342023	0.7776735059142653
XP_342024	0.9229418514392855
XP_342025	0.8300778179755347
XP_342026	0.9774538684538537
XP_342027	0.9944418122152768
XP_342029	1.0295795137127035
XP_342031	0.17527095157870723
XP_342032	0.9312321258209292
XP_342033	1.265103892542901
XP_342034	1.2524571938262352
XP_342035	-1
XP_342036	0.6198039265612744
XP_342038	2.018616987228514
XP_342040	1.0709349661559453
XP_342043	0.8143815145096189
XP_342045	0.8597046895388399
XP_342052	0.9482548920834105
XP_342058	1.0255158413131888
XP_342066	-1
XP_342069	1.2013927807853857
XP_342074	8.774317757547077
XP_342076	1.0860743477260535
XP_342077	0.9746343373371419
XP_342083	1.7707589576668372
XP_342084	1.246391590060826
XP_342086	1.4326743318856932
XP_342087	1.2985918038205924
XP_342092	-1
XP_342093	1.0399741848976465
XP_342094	1.33899466900429
XP_342095	0.9860685147767696
XP_342100	1.0943476083210382
XP_342101	1.066791132836963
XP_342102	0.9351932258355308
XP_342103	-1
XP_342104	0.9973028041868931
XP_342105	1.4401801970367212
XP_342108	1.073830831772496
XP_342110	1.0058886296185907
XP_342112	-1
XP_342116	1.0558760707199388
XP_342118	1.294566896823055
XP_342119	1.305255581467794
XP_342120	0.9993088089667747
XP_342122	-1
XP_342123	1.1926576222842902
XP_342125	-1
XP_342132	1.280592052092416
XP_342143	1.2794930607934347
XP_342144	1.2438834176174263
XP_342150	1.2010914542753828
XP_342161	1.7392983195885476
XP_342169	1.2225585486099824
XP_342173	1.0371133423682832
XP_342175	1.0595011401363745
XP_342180	0.9953308839863411
XP_342182	0.5458999981649925
XP_342184	1.1214855071081222
XP_342186	1.034122469296185
XP_342187	1.9923325173467366
XP_342190	1.2801605255244208
XP_342201	0.7660996988342929

XP_342202	0.6426240043968529
XP_342210	1.1747545986859196
XP_342211	1.3305070779436998
XP_342216	0.8415595135675343
XP_342218	1.687232782323009
XP_342219	-1
XP_342220	1.0477569171125583
XP_342221	2.34104868568606
XP_342224	1.199321395839984
XP_342226	0.8023220826211092
XP_342232	0.7463753277627218
XP_342233	1.1148730727483926
XP_342239	1.277830997835366
XP_342244	0.7191528782391307
XP_342245	0.5366264385725662
XP_342247	0.9934765882123867
XP_342250	0.8444436214052923
XP_342252	-1
XP_342253	0.7910935148117924
XP_342257	-1
XP_342258	1.2167805798516698
XP_342263	1.032444387762707
XP_342264	1.1001762039054328
XP_342269	1.0327025824516327
XP_342275	-1
XP_342279	1.1651708386172333
XP_342280	0.89686009106204
XP_342282	-1
XP_342283	1.018604031011018
XP_342289	1.0220689475049844
XP_342292	-1
XP_342294	1.0648219213034593
XP_342296	1.179929452067438
XP_342297	1.5003012556330262
XP_342299	1.0281447589900277
XP_342302	0.897569328643939
XP_342304	1.2886446390971578
XP_342307	1.171724155573358
XP_342308	1.0909550650409507
XP_342309	1.2201725360250453
XP_342310	1.285430254490113
XP_342311	0.7410186814843591
XP_342318	0.9208893754688393
XP_342319	1.065493978877131
XP_342320	0.7149289475792724
XP_342321	-1
XP_342322	1.2898144575458574
XP_342326	-1
XP_342332	1.7082883316513808
XP_342333	0.9994963862092064
XP_342338	14.35243744938791
XP_342341	1.0758303044705446
XP_342342	0.7819618914160418
XP_342343	1.1345044725530986
XP_342345	1.0660913817750852
XP_342346	0.923711494488178
XP_342347	1.0711618572148018
XP_342348	0.9374537182182429
XP_342354	1.1136420060653327
XP_342360	0.9772890957755251
XP_342364	1.262572148029423
XP_342365	1.1217674798220483

XP_342369	1.0831854853418894
XP_342370	0.6345346179823413
XP_342375	1.1589181281484746
XP_342383	0.5566583557368577
XP_342385	1.183065812478992
XP_342390	0.9992945887710052
XP_342391	1.0506843776636563
XP_342392	1.0666493611241088
XP_342393	1.1992351587848122
XP_342394	0.4685689789247626
XP_342396	2.0466427319418408
XP_342397	1.2046492014029608
XP_342398	0.8483611234819103
XP_342399	1.1244389809314443
XP_342401	1.0789545318471208
XP_342402	1.2404488638477338
XP_342405	0.947646015689277
XP_342409	1.5498623790958985
XP_342410	0.7989728965454648
XP_342413	-1
XP_342416	-1
XP_342418	-1
XP_342421	1.0460022621523002
XP_342431	1.0143227940124575
XP_342433	-1
XP_342439	1.3336890429748793
XP_342441	-1
XP_342444	-1
XP_342451	1.0688467324914686
XP_342453	0.7940212010535218
XP_342455	1.037798815058337
XP_342456	-1
XP_342459	0.7039565773645866
XP_342465	-1
XP_342468	0.8229314178999299
XP_342470	0.9455851586491069
XP_342471	-1
XP_342479	1.7734335869748994
XP_342482	-1
XP_342484	0.7716636561590225
XP_342486	1.184687475905478
XP_342489	0.7515453350115396
XP_342492	0.9419622430398239
XP_342494	2.7957887988479264
XP_342495	1.007444886842277
XP_342497	0.9701316606220775
XP_342499	1.0236338578968753
XP_342501	1.056617103005267
XP_342503	0.7844986204798415
XP_342513	0.9421504199784438
XP_342517	1.2662763908957684
XP_342518	0.9681600717595664
XP_342521	0.7789853195404229
XP_342522	0.6946801830232237
XP_342523	1.1836689036056927
XP_342527	1.1170525244641099
XP_342529	1.4114265746699821
XP_342530	1.2018888063693391
XP_342531	0.9559478780221626
XP_342532	1.2586547896997793
XP_342534	1.1162845921764275
XP_342535	1.0751124228976907

XP_342536	1.7973150214666198
XP_342543	1.0697914821259682
XP_342553	1.1939523081299566
XP_342554	-1
XP_342556	-1
XP_342558	1.4009788315968497
XP_342559	0.8682979049779419
XP_342561	1.2240171154596842
XP_342563	1.0339163655626329
XP_342564	1.2243617589538183
XP_342568	0.8760286388143027
XP_342571	1.190916170990945
XP_342572	1.0609730878593904
XP_342574	1.0706607250883184
XP_342576	-1
XP_342577	-1
XP_342578	1.2045124373472074
XP_342579	1.6182242646082772
XP_342582	1.1433035628910644
XP_342589	1.4489541912494015
XP_342592	1.23941158430904
XP_342595	1.6421441750605426
XP_342602	1.3873528580237016
XP_342608	-1
XP_342609	1.7477953693355175
XP_342613	1.246230320557704
XP_342619	1.3044802162336033
XP_342627	-1
XP_342630	1.040637806545152
XP_342631	1.2388406663382794
XP_342632	1.5294493624093086
XP_342633	1.2288668498294313
XP_342637	1.4881341728792308
XP_342639	1.0785183238285587
XP_342641	1.221356888324163
XP_342642	1.263058312942936
XP_342643	0.9057481393372475
XP_342644	1.7259852924507866
XP_342645	-1
XP_342647	1.0852025282205329
XP_342648	1.14908321162671
XP_342654	1.0239025467552623
XP_342656	1.0819914797701027
XP_342658	0.861907401548352
XP_342662	-1
XP_342663	1.149448326713417
XP_342665	0.6801400868293174
XP_342673	1.0147178596810258
XP_342674	0.9015826666861683
XP_342676	0.8079307319371936
XP_342677	1.1058973441079958
XP_342680	1.1290216664126749
XP_342681	1.2981780364197089
XP_342682	2.1906084735621225
XP_342684	1.0397495169234714
XP_342687	0.8071181381111687
XP_342691	1.2668810256133853
XP_342693	1.0585431585931524
XP_342701	0.9681070788300213
XP_342703	1.0219327523027355
XP_342706	0.9255777600309892
XP_342707	-1

XP_342708	-1
XP_342711	1.0558112703967273
XP_342712	1.025519524852838
XP_342713	1.0577513891497814
XP_342715	0.7282018107368603
XP_342718	0.9116015726475754
XP_342722	-1
XP_342730	0.5051473355446462
XP_342732	-1
XP_342736	0.7985049088000462
XP_342737	2.004898332063711
XP_342739	0.7793579348111032
XP_342740	-1
XP_342742	1.1248809199503713
XP_342746	0.9096737865418691
XP_342747	0.5356167764679687
XP_342749	0.9893133525918689
XP_342751	0.6532307814613746
XP_342755	0.697751391400072
XP_342756	0.7644007989092786
XP_342758	-1
XP_342759	1.24624331700079
XP_342760	0.9208411831616783
XP_342761	3.5676498273576764
XP_342762	0.769386946295104
XP_342763	-1
XP_342764	0.94348425297765
XP_342766	1.7863918065602236
XP_342771	-1
XP_342776	0.9460995501484738
XP_342778	1.2017905417422805
XP_342779	1.081676447255756
XP_342780	1.502219229643363
XP_342781	-1
XP_342785	0.7559917033460885
XP_342793	1.2923640331329325
XP_342794	1.1781262970229007
XP_342795	-1
XP_342802	0.8639725505756697
XP_342807	1.05002340103112
XP_342813	1.0934273584508898
XP_342816	1.2314147661493948
XP_342817	1.0478792941234296
XP_342821	1.0116516605804513
XP_342823	1.0115257205089219
XP_342824	4.968392723944186
XP_342829	1.2115751736456468
XP_342830	0.7007619294030194
XP_342832	-1
XP_342833	1.115923731622414
XP_342834	1.0679291421845696
XP_342835	-1
XP_342836	0.8679288999326847
XP_342837	1.2568539654648208
XP_342841	1.159562012764177
XP_342847	0.863479006312732
XP_342849	1.1275467905787506
XP_342851	0.648357476818311
XP_342852	1.1305050842047195
XP_342854	1.4406551978493398
XP_342855	1.01302623331896
XP_342856	1.1420986863362934

XP_342857	-1
XP_342858	-1
XP_342859	1.2873405853491624
XP_342867	1.016247131709988
XP_342870	1.0189637518389703
XP_342878	1.272587610533605
XP_342885	0.7887285309649585
XP_342886	1.25534760254605
XP_342888	0.874116320949828
XP_342889	0.6688764849293477
XP_342894	1.0664916972258194
XP_342895	0.8477238878657131
XP_342896	-1
XP_342897	0.9896601443422551
XP_342898	0.7751629801695897
XP_342904	-1
XP_342906	1.4236705925875055
XP_342908	0.7487937704353466
XP_342909	1.4812341386117804
XP_342910	1.3041878232577249
XP_342912	1.130085032156346
XP_342916	0.4489928889480532
XP_342917	1.1359031482570414
XP_342921	1.1110993661295163
XP_342924	1.2080530359639068
XP_342928	0.9620840920201824
XP_342931	-1
XP_342933	0.997586205892263
XP_342937	-1
XP_342940	0.4224426549451168
XP_342942	-1
XP_342949	1.1517142428722444
XP_342950	1.0884369551883477
XP_342952	1.1490691803098525
XP_342953	-1
XP_342958	0.8108821716101099
XP_342959	0.821333860728756
XP_342961	1.486090491255685
XP_342962	1.1262618513923575
XP_342963	1.2087223481577334
XP_342964	0.898320490585188
XP_342971	1.2870751407625913
XP_342976	-1
XP_342978	-1
XP_342979	1.335228857351376
XP_342980	1.4868322580258484
XP_342981	1.1521525038484228
XP_342983	1.1938069086148062
XP_342985	-1
XP_342986	1.1356716077216424
XP_342989	1.2334978577950495
XP_342995	0.8867269483987674
XP_342996	1.1702962958981327
XP_343001	1.3553643608402073
XP_343003	0.849512025603082
XP_343004	0.6559773144471653
XP_343007	1.1885896121442476
XP_343008	1.5885538121483842
XP_343010	1.0401007756347964
XP_343015	1.2873302044379296
XP_343019	0.9445707256279668
XP_343020	1.7657441412502488

XP_343026	1.0020656781563377
XP_343027	-1
XP_343028	0.9238854793986984
XP_343029	0.7712771311878708
XP_343034	-1
XP_343036	-1
XP_343038	0.843818790300633
XP_343040	1.4709436908522484
XP_343045	1.0038866697344007
XP_343046	2.7605482159082855
XP_343047	0.7364437349408447
XP_343049	1.0983059100950603
XP_343058	1.0326255499124277
XP_343060	0.968944628983264
XP_343063	0.8628157334483777
XP_343066	2.2399958965995825
XP_343068	1.0415985585148955
XP_343073	1.0680779309450779
XP_343081	0.9532492383283886
XP_343085	0.9781875878182795
XP_343087	0.7813326609843284
XP_343089	1.1521021043187245
XP_343097	1.309511358358815
XP_343099	-1
XP_343100	1.0568403390041725
XP_343104	0.6850442861003229
XP_343108	0.9742458318425489
XP_343113	0.8934754909394096
XP_343114	1.917903454384754
XP_343115	1.277418040362885
XP_343116	0.9710391297514973
XP_343119	1.0471246929509224
XP_343120	1.1140847837731072
XP_343122	-1
XP_343123	-1
XP_343128	1.4172272520547249
XP_343130	-1
XP_343131	1.904650028862567
XP_343132	2.5057026976108765
XP_343136	1.111867953084012
XP_343138	-1
XP_343139	1.007229346714261
XP_343141	0.8427261563430483
XP_343142	0.8686614851654277
XP_343144	1.205568729413238
XP_343145	0.941748832912038
XP_343148	0.8153705833256439
XP_343149	-1
XP_343153	1.0201010450056414
XP_343154	0.7707965516443582
XP_343156	1.0523250684693506
XP_343158	0.7485398883887343
XP_343159	1.0425612145379024
XP_343160	1.3693687007825768
XP_343163	0.8061593953796615
XP_343165	1.2801006922532636
XP_343167	1.3136143438118255
XP_343170	0.7709931360033297
XP_343172	-1
XP_343174	1.1317512722592644
XP_343175	0.7902703795926943
XP_343176	1.2919480272266413

XP_343177	1.2426063230191522
XP_343179	0.9639226948557269
XP_343180	0.9567758603406719
XP_343182	-1
XP_343184	0.7531075528687606
XP_343189	0.8169165143592432
XP_343190	0.7273239377859952
XP_343192	0.9053998980262448
XP_343193	0.8347774366030585
XP_343195	1.4495660322514528
XP_343197	-1
XP_343198	-1
XP_343203	-1
XP_343205	1.0579392933640412
XP_343208	1.1379721106767617
XP_343209	0.9850321311459855
XP_343219	-1
XP_343220	1.0105815754108398
XP_343222	1.0474624730644617
XP_343224	0.9824585983595432
XP_343226	0.9833961952424662
XP_343227	3.1431925569778922
XP_343230	1.1045209158402671
XP_343235	1.086221110398624
XP_343243	0.8311368244446867
XP_343245	0.6606262877409458
XP_343249	1.2009357303170156
XP_343250	1.1768478150481272
XP_343251	1.5900687046674475
XP_343252	0.9458715882988052
XP_343256	1.0275889091410775
XP_343257	-1
XP_343260	0.835577340836891
XP_343263	0.9177872838205414
XP_343267	1.0012453885295707
XP_343269	0.9810197911066234
XP_343271	0.9095080349778168
XP_343272	1.698701133031243
XP_343273	0.968856391361447
XP_343274	0.967913395546277
XP_343275	0.910617421416553
XP_343279	1.0778156142550244
XP_343281	0.8197059118950553
XP_343282	0.9880973397827916
XP_343285	-1
XP_343286	0.8875187730236132
XP_343287	1.2011653005193896
XP_343289	1.1461771747628295
XP_343296	1.520247160400549
XP_343297	0.880200263844506
XP_343299	1.1730511287302754
XP_343304	0.9692996182220992
XP_343306	1.0988242262296082
XP_343307	-1
XP_343309	1.003958181052018
XP_343314	-1
XP_343315	-1
XP_343320	1.003119321134653
XP_343326	-1
XP_343327	1.0126315213266754
XP_343328	6.937465375544986
XP_343330	0.31934150944530765

XP_343332	1.2533717754284182
XP_343333	1.0795975962780509
XP_343334	1.7745367674071109
XP_343335	-1
XP_343337	1.4753138599614128
XP_343338	1.1790674904378657
XP_343339	1.064169534423076
XP_343340	0.7664956836034503
XP_343343	0.6970701469719374
XP_343346	3.251902385152993
XP_343347	0.8354331741713329
XP_343352	1.1136294912126676
XP_343355	0.9939048744953389
XP_343356	-1
XP_343357	1.1018173132057383
XP_343359	1.0171131623318934
XP_343360	1.0183896049441328
XP_343364	-1
XP_343366	1.070081609377228
XP_343379	1.0822841983878255
XP_343381	-1
XP_343384	1.1942742816224778
XP_343385	-1
XP_343386	0.9099779884432571
XP_343387	0.9851927910987884
XP_343390	1.0871997445530284
XP_343393	0.7981231019802989
XP_343395	-1
XP_343396	0.7603945295691918
XP_343397	1.0163676618169386
XP_343401	-1
XP_343402	1.1445445816459692
XP_343403	0.16520778399034927
XP_343406	1.0483653439244618
XP_343409	0.6577398873446545
XP_343410	1.0099216131134205
XP_343411	1.2784524029068929
XP_343413	0.7977957917131282
XP_343415	-1
XP_343416	0.8500194106380019
XP_343419	0.9450790137393468
XP_343420	1.0024305743534774
XP_343421	0.7450763035020087
XP_343427	1.487822312454244
XP_343428	1.4406418715357148
XP_343435	-1
XP_343439	1.8765133929862454
XP_343440	1.130141886898282
XP_343443	1.1830756568724603
XP_343447	2.3419244104089643
XP_343451	2.2679574711741237
XP_343459	1.2346457072580772
XP_343463	-1
XP_343467	1.1291055688282947
XP_343470	0.9397607022579219
XP_343471	-1
XP_343472	-1
XP_343473	0.5187102940279779
XP_343474	1.2335982092435214
XP_343476	1.014808524623574
XP_343479	-1
XP_343480	10.112516590347807

XP_343483	0.7539589972332553
XP_343484	1.3752529284295092
XP_343486	-1
XP_343488	1.3657750067305985
XP_343489	1.1584861180948367
XP_343490	0.6957974013420415
XP_343491	0.7462971625881546
XP_343493	-1
XP_343494	-1
XP_343495	1.2456147105470228
XP_343497	1.1664788610752683
XP_343498	-1
XP_343502	0.9240948997843814
XP_343504	1.0805361042496615
XP_343509	1.3721113093024477
XP_343511	0.9108814538700879
XP_343512	1.0281376162593832
XP_343514	0.7065630933690605
XP_343525	1.1829431544364044
XP_343529	1.0788977246978153
XP_343532	0.700508995565552
XP_343538	1.0699159335648698
XP_343554	1.0189856849855654
XP_343556	1.363661438369619
XP_343558	-1
XP_343561	1.1826999271882792
XP_343562	1.1771333438378144
XP_343563	-1
XP_343564	1.1123626697502722
XP_343565	1.2931441660576153
XP_343568	-1
XP_343570	1.1851693244267663
XP_343571	0.9030583291319751
XP_343576	-1
XP_343577	1.4823637322999048
XP_343580	1.8631394251704911
XP_343581	0.8885678174879453
XP_343583	-1
XP_343584	1.4603718700490826
XP_343591	1.1047721968458644
XP_343593	0.9763071849202936
XP_343594	1.1659061316082335
XP_343600	0.4767241663364688
XP_343608	1.001459669310793
XP_343610	-1
XP_343614	1.1151837207543396
XP_343617	0.7052030951675897
XP_343626	1.7111403643236738
XP_343629	0.9789394912060206
XP_343630	1.0281687722446387
XP_343631	0.9808906134748812
XP_343632	0.9991713614493388
XP_343640	1.0968116372299612
XP_343644	0.965157586617896
XP_343650	0.8973321631290698
XP_343651	0.46888513215281846
XP_343668	1.1203443558197559
XP_343671	1.0035238717124964
XP_343672	1.1998693617759915
XP_343673	1.1599445813371834
XP_343683	1.440061569576353
XP_343710	-1

XP_343712	1.4984817553350809
XP_343733	0.792874571455663
XP_343740	-1
XP_343761	0.9456740143727727
XP_343765	1.2694585105838598
XP_343766	1.9364655807260907
XP_343767	0.9902712595282632
XP_343768	-1
XP_343770	0.9919346304029646
XP_343771	-1
XP_343777	1.713151729685183
XP_343779	-1
XP_343780	0.9090375839058001
XP_343790	1.9922821548861354
XP_343791	-1
XP_343794	0.9508413320988142
XP_343796	1.1499695051342425
XP_343797	-1
XP_343798	1.0918980406932552
XP_343800	0.867847702760428
XP_343801	0.9837043980083324
XP_343808	-1
XP_343809	0.7717473132773205
XP_343813	1.2678531853433277
XP_343819	1.0363892982249967
XP_343823	-1
XP_343824	1.1034063111512051
XP_343832	1.5229955979506935
XP_343834	0.9294216742220853
XP_343836	0.9249172252438099
XP_343840	-1
XP_343843	1.0126345410573425
XP_343844	0.6672968151486947
XP_343845	0.6682786510676145
XP_343857	1.0238039731786974
XP_343859	1.12455396916109
XP_343861	1.143056749389441
XP_343862	-1
XP_343868	0.9758073673507551
XP_343869	0.6699190412211902
XP_343870	0.7954537934933507
XP_343871	0.8449662755735433
XP_343873	1.0310499772637138
XP_343891	1.0199792151161908
XP_343900	1.0543775240601183
XP_343903	-1
XP_343912	-1
XP_343920	1.3535305823879467
XP_343923	1.0111169264302846
XP_343936	0.8391603035316463
XP_343939	1.3371616070250634
XP_343946	0.8792408208436768
XP_343949	1.3325020846389077
XP_343953	1.0604903021811245
XP_343955	1.4855972440692713
XP_343956	0.840585124646232
XP_343957	0.9849473079189978
XP_343961	0.7088991786344859
XP_343963	-1
XP_343967	0.9325134596714967
XP_343983	-1
XP_343985	0.9916633564389962

XP_343987	0.9316567497513096
XP_343989	0.8851049191676985
XP_343990	0.7427254605475406
XP_343992	-1
XP_343999	1.0232151505868217
XP_344000	1.4179429722761112
XP_344006	0.6227383868519673
XP_344010	1.4924381647363385
XP_344017	0.8302853560629218
XP_344034	0.9721755869164923
XP_344036	1.9791202402545582
XP_344040	1.051226442824232
XP_344041	1.3140068677039711
XP_344043	0.9121991077803627
XP_344045	0.9922522795137041
XP_344047	0.9101011823786916
XP_344048	1.0661575022036744
XP_344057	1.0291895131487319
XP_344058	1.1799295052415386
XP_344061	-1
XP_344067	0.8087642616869651
XP_344071	-1
XP_344077	-1
XP_344078	0.8371579695469151
XP_344083	1.1510390888248914
XP_344086	0.7461800103084423
XP_344091	0.8793505373976135
XP_344092	0.290360124578007
XP_344098	-1
XP_344106	0.682498590558917
XP_344119	1.2521626177769603
XP_344131	1.0143039145370176
XP_344135	0.9217821327627411
XP_344136	1.043224924451525
XP_344147	0.3094486435496945
XP_344151	1.5240238175713785
XP_344168	0.8858075686234149
XP_344181	0.6647051257370941
XP_344183	0.8248132294900451
XP_344190	0.3336982031247153
XP_344192	1.9865323700547777
XP_344195	2.825638512608086
XP_344196	0.69830622525834
XP_344199	1.0738729349737404
XP_344205	1.2849541024462334
XP_344206	1.414787246342019
XP_344208	0.9774378116040694
XP_344231	1.6419448265569683
XP_344232	1.3153530503529567
XP_344236	-1
XP_344239	-1
XP_344253	-1
XP_344256	0.8680062216850182
XP_344258	0.9665197009633273
XP_344259	0.7479238301500754
XP_344260	1.380132086351312
XP_344261	1.0668424902962084
XP_344262	1.5487335330769
XP_344268	1.5661798224944359
XP_344269	1.0810759316488212
XP_344272	1.127275780823661
XP_344276	1.0278944032088908

XP_344278	1.1186869700411266
XP_344280	1.3819440963831768
XP_344285	1.1618590081698512
XP_344297	1.1405878122935695
XP_344311	1.0927593529446111
XP_344312	1.326043474329589
XP_344323	1.1552663312950575
XP_344329	1.0787070766697966
XP_344373	0.7593183853217278
XP_344389	1.0638246092224843
XP_344392	-1
XP_344403	1.0527917004216483
XP_344405	0.8108703660608518
XP_344406	0.8353940766878033
XP_344407	-1
XP_344410	1.1732457818450273
XP_344415	0.9282737954657311
XP_344426	0.899045707990584
XP_344435	1.444446992848654
XP_344436	1.5696003460783137
XP_344441	-1
XP_344444	1.1386675543086289
XP_344456	0.8538831180116646
XP_344459	0.9662511368215937
XP_344463	0.8349752948752845
XP_344470	1.1109395327631302
XP_344473	1.263551876369837
XP_344480	0.8571687183197056
XP_344483	1.2251999613375741
XP_344490	0.747965792917857
XP_344491	0.7271525521954011
XP_344492	0.8587628744124114
XP_344494	0.8606716079070612
XP_344495	1.058532451793156
XP_344496	0.8438650419438778
XP_344500	-1
XP_344503	0.9864454989443252
XP_344506	0.6340510622567933
XP_344524	1.0955424371976394
XP_344525	1.32352846966794
XP_344530	1.330628911079784
XP_344531	1.0109893810849178
XP_344538	1.0895490164147286
XP_344539	1.1231340623263721
XP_344543	1.105996223593338
XP_344545	0.7043200733683254
XP_344551	1.082820255596663
XP_344554	0.9345493220400454
XP_344557	-1
XP_344568	0.8921077085692901
XP_344569	0.9329964356409294
XP_344571	0.9185769996769159
XP_344572	1.1072735443427344
XP_344575	0.8363430143129497
XP_344581	-1
XP_344590	0.9698700199542373
XP_344595	1.1779000777402908
XP_344596	0.5210543752171618
XP_344600	0.9314226643996935
XP_344603	-1
XP_344612	1.4302452951837432
XP_344617	1.4428857122171959

XP_344619	0.9814718556010003
XP_344626	-1
XP_344628	-1
XP_344629	1.4076240842853398
XP_344634	-1
XP_344639	1.017563946983107
XP_344649	1.0796687950959558
XP_344650	0.020713723932974024
XP_344657	-1
XP_344662	0.9290324330115758
XP_344668	-1
XP_344669	1.70976832985916
XP_344670	0.8720541305457554
XP_344674	0.8567904699534402
XP_344686	1.2350376039905862
XP_344692	1.025952030065934
XP_344695	0.8932321685694906
XP_344696	1.2451922987150217
XP_344697	-1
XP_344704	1.7895147738912502
XP_344708	1.2729479074438277
XP_344711	0.9280252618397895
XP_344715	1.066859514974895
XP_344719	1.2586112768088493
XP_344723	0.7793583207738088
XP_344724	1.0255838539793067
XP_344729	-1
XP_344738	-1
XP_344743	-1
XP_344744	1.4699820581371608
XP_344745	1.0550640238577373
XP_344751	1.4644257946799755
XP_344755	1.38995116807207
XP_344757	0.713670074348905
XP_344763	0.8022915778917675
XP_344764	-1
XP_344768	-1
XP_344777	-1
XP_344783	0.9324702242349758
XP_344784	1.187858270989933
XP_344786	1.287946886403831
XP_344788	1.1470294370364613
XP_344792	1.1332703051225381
XP_344796	-1
XP_344799	1.1753385061274393
XP_344805	-1
XP_344806	1.1876619031059155
XP_344808	1.2481973327677283
XP_344811	-1
XP_344819	0.7070488322609348
XP_344828	1.6921838573539991
XP_344831	1.1667164999074853
XP_344838	1.1500803085066
XP_344845	0.893552845365763
XP_344848	1.091494519023083
XP_344856	-1
XP_344857	-1
XP_344859	1.0832382576958495
XP_344861	1.0072001596061737
XP_344862	1.1770993370057123
XP_344863	1.3719008804994024
XP_344864	1.564404701899722

XP_344870	0.783370579808019
XP_344875	1.1274202795108177
XP_344880	0.8744729209890214
XP_344888	0.8306376308183404
XP_344889	1.323669429044452
XP_344890	1.2725618020296896
XP_344891	1.0899782674130967
XP_344896	0.9852959118815594
XP_344902	0.9633056596937825
XP_344904	0.9914806716896528
XP_344909	1.068619682852724
XP_344911	1.064911589074481
XP_344916	1.1259666489258067
XP_344917	-1
XP_344924	0.9017414229786987
XP_344926	1.1613517300531708
XP_344935	0.9675762311227004
XP_344943	-1
XP_344944	1.0216749993009477
XP_344948	-1
XP_344953	-1
XP_344958	0.775863076185515
XP_344962	0.7468916311872015
XP_344964	2.552265861403935
XP_344965	26.38274736244219
XP_344970	1.022767791284697
XP_344972	1.206007193316856
XP_344973	1.0038674659848887
XP_344977	1.0132805804746665
XP_344978	-1
XP_344980	1.4141788775145223
XP_344982	0.9966728675205679
XP_344984	0.7490287433773158
XP_344985	1.3377597946349196
XP_344987	-1
XP_344993	1.2255018441249732
XP_345002	1.1597978119825851
XP_345010	0.9128146278314425
XP_345013	1.012701900127379
XP_345030	1.172698346675079
XP_345032	0.9229657958151467
XP_345033	0.9732946051920415
XP_345034	0.9523641002664406
XP_345039	1.6480385146332577
XP_345041	0.9424014637869886
XP_345042	1.0281549655975735
XP_345047	0.8366633358888654
XP_345051	0.9007854634451501
XP_345061	0.7547798774955171
XP_345064	1.4291534958658714
XP_345065	1.195038915488689
XP_345068	-1
XP_345076	1.0102652845816145
XP_345077	2.222304066205148
XP_345080	1.0420584900185241
XP_345083	0.8328886563034753
XP_345088	0.9143485463061639
XP_345092	0.7328647205086632
XP_345093	-1
XP_345106	1.2341068576018392
XP_345107	1.0666200621413673
XP_345110	1.2208573100471911

XP_345112	-1
XP_345113	1.2133704946467037
XP_345114	1.1695751496863358
XP_345115	2.210719234062027
XP_345118	1.1328540359330124
XP_345138	1.1711157294360417
XP_345139	-1
XP_345140	0.7682061926716491
XP_345141	1.0782756222567056
XP_345144	1.0091528002067875
XP_345145	-1
XP_345148	1.19764590137379
XP_345151	1.1912014970019948
XP_345152	1.0761606988660029
XP_345154	1.2088163798588423
XP_345160	1.1789713772527082
XP_345161	1.0978668111232137
XP_345162	0.8059904039076428
XP_345176	0.9479020263482812
XP_345178	-1
XP_345179	1.0028049239006416
XP_345185	-1
XP_345186	1.1617568749989944
XP_345191	1.8836023688334957
XP_345201	1.2297378406370183
XP_345212	0.9400674438271649
XP_345216	0.8532326928565491
XP_345225	1.1169166607876906
XP_345230	1.0841152161798322
XP_345233	1.1734996372806983
XP_345236	0.5107001793958607
XP_345237	1.449873772567397
XP_345238	1.2762134677005548
XP_345247	0.9548100343284243
XP_345250	-1
XP_345254	0.5691378514964529
XP_345256	0.7435475858049001
XP_345260	0.9187441537823272
XP_345271	0.7084318421130266
XP_345273	1.167128173715157
XP_345289	1.0329431112782905
XP_345300	0.46783471315211145
XP_345302	1.206878519023572
XP_345305	-1
XP_345319	1.4674879772617102
XP_345320	1.6904798438919244
XP_345324	1.112245925848335
XP_345325	1.1755620025550406
XP_345333	0.8355377271012204
XP_345336	0.9244654885805668
XP_345340	0.3825183009262953
XP_345341	0.628351918389144
XP_345348	1.0273320847888727
XP_345349	-1
XP_345358	1.283536905845055
XP_345382	1.0384909732593177
XP_345393	-1
XP_345402	1.1790999634762371
XP_345403	-1
XP_345404	-1
XP_345410	1.2767105661995881
XP_345411	0.7907630943156678

XP_345414	0.9116721793089653
XP_345418	0.9676656678728351
XP_345419	0.9638930272205183
XP_345420	-1
XP_345427	1.073484577742774
XP_345432	0.9636960309793902
XP_345439	1.5719548464895645
XP_345442	1.5388635504848989
XP_345445	-1
XP_345446	0.8584703889000463
XP_345450	-1
XP_345455	-1
XP_345457	0.5315537918709855
XP_345462	1.485505480063039
XP_345463	0.8009589815124658
XP_345469	0.7063452210460922
XP_345481	1.1547976137555702
XP_345483	0.9096104100595032
XP_345486	1.003304413387799
XP_345487	1.276545513646618
XP_345488	0.883568854105729
XP_345495	1.0141499384518833
XP_345509	2.384986670902421
XP_345510	0.8513801124695366
XP_345511	-1
XP_345512	1.5480177984534496
XP_345518	0.813996640111195
XP_345519	1.1690135045562753
XP_345523	-1
XP_345525	1.0714075591549097
XP_345526	0.8329885035327135
XP_345529	1.1325848042249251
XP_345530	2.2165841955833128
XP_345536	0.968747766068509
XP_345538	0.8489166629478163
XP_345541	0.8760915108213798
XP_345543	0.7388869088815468
XP_345562	0.9576237603327987
XP_345568	1.6612924404988745
XP_345570	-1
XP_345571	0.8172346192567886
XP_345573	1.2074368198625554
XP_345578	0.9557363587198102
XP_345582	0.8872157446228006
XP_345584	1.2105880588846902
XP_345585	0.9555189308574412
XP_345590	1.0917096497971708
XP_345592	-1
XP_345596	1.0544830616166285
XP_345601	0.7980332922820357
XP_345604	-1
XP_345607	1.0215965813750068
XP_345616	1.0688773429460012
XP_345626	0.9518303355045363
XP_345628	1.2248073680094111
XP_345629	1.2012925197731161
XP_345631	1.3045700504763384
XP_345634	2.2946109052781467
XP_345640	-1
XP_345642	0.8494093316580432
XP_345643	1.0680019128875808
XP_345644	0.8033937703725629

XP_345647	2.774910091925083
XP_345652	1.056100806699982
XP_345670	1.1749416258932803
XP_345672	0.7775518409258404
XP_345675	1.2730855836849553
XP_345690	1.791511610111279
XP_345698	1.0590510526288637
XP_345704	1.1307867556318452
XP_345708	1.0039659429758057
XP_345710	1.044107938538194
XP_345726	1.1354084053402738
XP_345735	1.1616838434069836
XP_345739	0.8814811391438934
XP_345741	0.9230107241048445
XP_345764	1.1110886039964731
XP_345765	1.7039414380796003
XP_345786	-1
XP_345790	-1
XP_345791	-1
XP_345800	1.113410631762285
XP_345801	1.5246193378203377
XP_345802	1.0558040155992359
XP_345804	-1
XP_345805	-1
XP_345807	0.9938993161453343
XP_345814	1.2368256961528388
XP_345833	1.1857200812111282
XP_345836	0.9921903560705255
XP_345842	1.338113473732531
XP_345845	1.1346249340402037
XP_345849	-1
XP_345850	1.0394816353697784
XP_345851	0.983300035804212
XP_345857	0.6880648415143952
XP_345861	-1
XP_345862	0.9629168615372549
XP_345863	0.9678937120420121
XP_345864	0.9693469162293457
XP_345866	1.2178784708447277
XP_345867	0.9603630807300259
XP_345869	0.6824596273743911
XP_345871	0.7973085434771425
XP_345875	1.828254244700834
XP_345876	1.3456709522858492
XP_345877	1.202490080950117
XP_345880	-1
XP_345881	1.0477338458038175
XP_345896	-1
XP_345908	0.9736544787524719
XP_345910	0.8529180765595004
XP_345917	0.9846818681321018
XP_345919	2.095863487504786
XP_345933	0.9916020211093407
XP_345937	0.9605127647996694
XP_345939	1.124968121462045
XP_345940	-1
XP_345941	-1
XP_345943	0.838310241972589
XP_345947	-1
XP_345948	1.0574156665539578
XP_345958	0.9417891962123138
XP_345972	0.9661997435618326

XP_345974	-1
XP_345976	-1
XP_345981	0.9228101867987846
XP_345984	-1
XP_345985	1.3977474834524493
XP_345986	1.1285445863967964
XP_345995	1.7319029972534583
XP_346008	1.156399047797566
XP_346013	1.0668323439742302
XP_346018	1.4965826882992783
XP_346021	1.0960069262408016
XP_346028	1.2829371005174128
XP_346029	1.0560121701710747
XP_346040	-1
XP_346041	0.9317532144522657
XP_346047	-1
XP_346054	1.0015267392149645
XP_346061	0.828781978506882
XP_346062	1.0426342376503277
XP_346069	1.0085605701826366
XP_346070	0.9252708573107338
XP_346071	1.1670354872382642
XP_346073	1.0469318120969193
XP_346074	0.8442372213963395
XP_346075	1.6227286632931381
XP_346078	0.8926010583480992
XP_346079	-1
XP_346084	1.1968120213412414
XP_346086	0.9237511625174863
XP_346090	1.210797709884966
XP_346093	1.0769374681072932
XP_346097	1.0907083846400591
XP_346101	1.066558082112495
XP_346111	-1
XP_346120	1.3141210133475538
XP_346123	0.8921356410777577
XP_346124	1.0297946995405256
XP_346140	1.6251780202966575
XP_346144	0.9803590562551637
XP_346159	1.2577809512136133
XP_346166	0.7550611899077143
XP_346174	-1
XP_346207	4.490291420829757
XP_346231	0.7720431240325324
XP_346236	-1
XP_346238	1.0952250310910012
XP_346248	3.0817006759624435
XP_346254	1.150210222064984
XP_346258	0.9095154327588375
XP_346259	1.2426964927858803
XP_346280	1.6917338377562299
XP_346281	1.1899567056813478
XP_346282	-1
XP_346302	-1
XP_346304	0.7844727085709591
XP_346308	1.1296993380175129
XP_346313	0.9534324641292072
XP_346317	1.191401586841281
XP_346329	1.0639653387066923
XP_346330	1.0639456832190421
XP_346331	-1
XP_346346	0.3127897333251646

XP_346347	1.1532008562414817
XP_346349	1.1382262300427728
XP_346350	0.8910514077239696
XP_346363	1.060305456914926
XP_346367	1.0158003348087186
XP_346368	1.1093536122084642
XP_346373	1.148016766637437
XP_346377	1.1720408225492278
XP_346381	-1
XP_346392	1.3336064377802348
XP_346394	-1
XP_346398	1.1260342441936737
XP_346403	0.7484873636384105
XP_346410	-1
XP_346448	1.2072300536280984
XP_346453	0.8484035718625424
XP_346474	-1
XP_346482	0.7235330409385957
XP_346492	-1
XP_346509	-1
XP_346524	0.7729284621722542
XP_346535	0.8394640520572215
XP_346565	1.095328358471708
XP_346567	1.4465902919520457
XP_346583	0.649445887820026
XP_346590	0.9912641262223314
XP_346600	1.0955797762771287
XP_346603	0.8904131384708828
XP_346612	1.0603916314231048
XP_346623	4.62391313442231
XP_346624	0.9845491925891107
XP_346635	1.0527250593225388
XP_346638	2.149441341074916
XP_346646	1.0900027353425232
XP_346650	1.4693984686528359
XP_346673	1.251255498508817
XP_346680	0.9134351876413332
XP_346694	1.1488010696065376
XP_346702	-1
XP_346706	1.407455401874375
XP_346708	0.593983758820127
XP_346711	1.331039560628874
XP_346732	-1
XP_346740	0.9209648384112906
XP_346749	0.9082373867982918
XP_346754	1.6202482558325866
XP_346794	0.35830196680847176
XP_346797	-1
XP_346804	0.8648711858551815
XP_346811	0.8528394713561426
XP_346813	1.0570471620795705
XP_346818	-1
XP_346836	1.1550521406119587
XP_346849	-1
XP_346854	-1
XP_346872	0.8902499649797208
XP_346882	0.8652574793094319
XP_346885	-1
XP_346886	-1
XP_346889	1.6456883814128103
XP_346890	1.0035334827135773
XP_346893	0.978688169572095

XP_346901	0.86955972007102
XP_346904	0.905207996224933
XP_346912	1.1749365583245204
XP_346915	1.3646714165095164
XP_346916	1.0240632272065826
XP_346920	1.003368453916357
XP_346921	-1
XP_346928	0.9738235069936371
XP_346934	1.4885951421511125
XP_346951	1.0011450142417664
XP_346952	1.2388460332755866
XP_346955	0.2093893676737657
XP_346957	1.8093110478068122
XP_346962	-1
XP_346964	0.5743124256361627
XP_346966	-1
XP_346967	1.3823964505900292
XP_346970	1.1419202438952596
XP_346971	-1
XP_346991	1.3862520114886978
XP_346994	0.9067470034628147
XP_346997	1.0537810834765924
XP_346998	1.3424658947176944
XP_346999	0.9370492967178565
XP_347001	-1
XP_347003	1.2030456615881502
XP_347004	1.2970345727338564
XP_347009	1.11079303952082
XP_347011	-1
XP_347017	0.7274766292755291
XP_347021	1.0692079568055217
XP_347025	1.0135924454292624
XP_347039	0.9922464161484547
XP_347041	1.160483271909822
XP_347046	-1
XP_347049	-1
XP_347055	1.3297709637253663
XP_347056	1.1140070675475857
XP_347057	0.8503709591866717
XP_347060	0.9567468143698673
XP_347061	1.16681334456917
XP_347063	0.9018929024641471
XP_347070	-1
XP_347072	0.9652599179594015
XP_347075	1.0647407417889043
XP_347078	-1
XP_347084	-1
XP_347091	-1
XP_347093	-1
XP_347101	1.1325775735459673
XP_347106	-1
XP_347109	1.282248998817373
XP_347111	1.5191805290157945
XP_347125	1.344007062939056
XP_347127	0.7734810461374724
XP_347147	0.9235028677484753
XP_347148	1.0428776473307297
XP_347149	-1
XP_347150	1.0940865929177153
XP_347151	1.05329475878863
XP_347156	0.9852629573382905
XP_347157	0.9252415929561467

XP_347164	2.632857307083655
XP_347167	-1
XP_347168	0.9518303823528322
XP_347169	1.8511138431422862
XP_347170	0.9178257082952515
XP_347171	1.1961944515474825
XP_347172	0.5284907105689577
XP_347175	0.9492224625784371
XP_347177	1.0532246917960915
XP_347180	1.2836964952580383
XP_347182	1.2365043595748542
XP_347183	4.307548273312038
XP_347184	2.344734463938124
XP_347189	0.8217167933107983
XP_347193	2.6139646617582426
XP_347194	2.893047782279892
XP_347195	0.5344122997821151
XP_347197	1.196992437363948
XP_347199	2.8371001441974415
XP_347203	-1
XP_347210	1.8799959777566402
XP_347211	1.2545582978327756
XP_347213	-1
XP_347215	0.7887102134299218
XP_347216	0.7998256077863024
XP_347217	-1
XP_347218	0.45125770072699806
XP_347223	1.2994074651594587
XP_347224	1.1148438705874437
XP_347231	-1
XP_347234	0.6497125502416731
XP_347245	0.6526535594971609
XP_347246	-1
XP_347254	1.0259650300025902
XP_347264	1.055534685714824
XP_347265	1.1050661859509119
XP_347268	0.9616122241178231
XP_347270	0.7612146288901677
XP_347276	1.005155459450785
XP_347278	1.080142026781113
XP_347281	-1
XP_347283	1.1198652202546493
XP_347289	0.5736114079921731
XP_347290	-1
XP_347291	-1
XP_347292	1.1354946066411638
XP_347296	0.8988922221243274
XP_347305	0.856105044585011
XP_347306	1.0126498420739793
XP_347310	1.0566468779799867
XP_347313	1.7518318246832305
XP_347317	0.8713476998221653
XP_347326	0.76583476143884
XP_347327	0.9275354377437434
XP_347328	-1
XP_347330	-1
XP_347333	0.6062561145701256
XP_347335	0.8889361254644426
XP_347336	1.478057366408868
XP_347338	-1
XP_347339	1.056251772442779
XP_347340	1.8996499525495423

XP_347343	0.9860898517241122
XP_347381	1.1806267204247811
XP_347384	1.0628567446714932
